# A Convenient, Safe, and Atom‐Economical Route to a Large Portfolio of Grubbs‐Type Catalysts for Olefin Metathesis via Four‐Coordinate Ruthenium Alkylidynes

**DOI:** 10.1002/anie.6803845

**Published:** 2026-03-04

**Authors:** Mingxu Cui, Alois Fürstner

**Affiliations:** ^1^ Max‐Planck‐Institut für Kohlenforschung Mülheim/Ruhr Germany

**Keywords:** atom economy, diazo derivatives, metathesis, ruthenium alkylidynes, ruthenium carbenes

## Abstract

*p*‐Tolyl(trimethylsilyl)diazomethane is a readily accessible and easy‐to‐handle net carbyne donor reagent. It reacts with [(*p*‐cymene)RuCl_2_]_2_ in MeCN to give the phosphine‐free chloride‐bridged dinuclear complex [(MeCN)_2_RuCl_2_( = C(*p*‐tolyl)(SiMe_3_))]_2_ (**2**) carrying a silyl group on each of the carbene ligands. Treatment of **2** with either PCy_3_ or an N‐heterocyclic carbene (NHC) causes elimination of TMSCl with formation of the corresponding four‐coordinate ruthenium alkylidynes. The non‐bonding electron lone pair of significant d_z2_ character at their Ru center is prone to protonation; in this way, the alkylidyne unit is converted into a carbene ligand under very mild conditions. The sequence of alkylidyne formation/protonation opens a novel entry into all relevant “generations” of Grubbs and Grubbs‐Hoveyda type catalysts, which is distinguished by a superior ligand‐ and atom economy, a good safety profile, and high overall yields. Moreover, the method is inherently flexible and arguably suitable for parallel screening and reaction optimization purposes.

## Graphical abstract

It may seem counterintuitive to first target a ruthenium alkylidyne when Grubbs‐type ruthenium carbene complexes are the actual goal. In the end, however, this tactic pays valuable dividends in terms of practicality, atom economy, flexibility as well as safety: the new route provides ready access to a large assortment of catalysts for alkene metathesis via a universal precatalyst that is easy to make and handle.

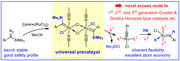

## 1 Introduction

**SCHEME 1:**
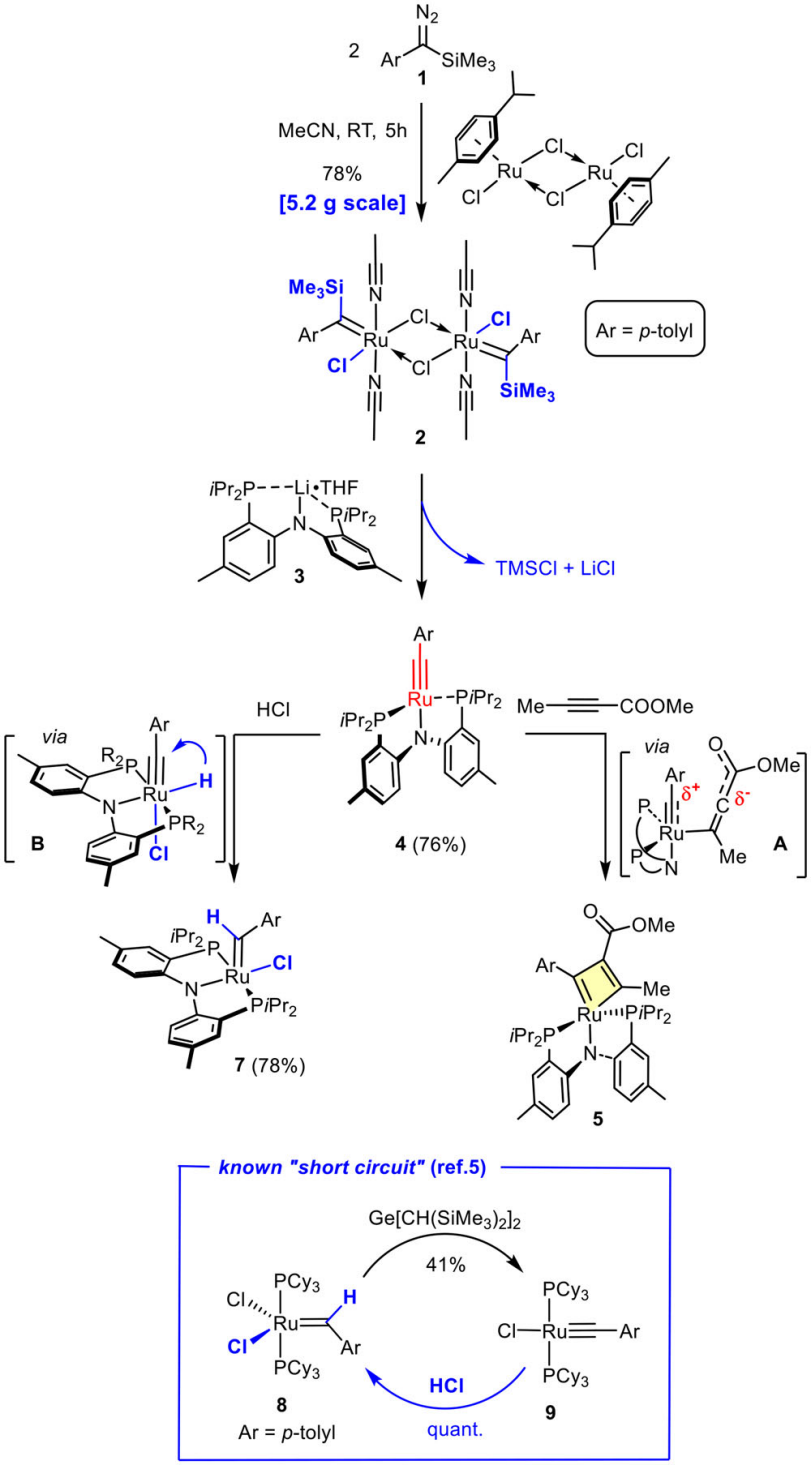
Use of a silylated aryldiazo derivative as carbyne donor reagent (ref [1]) and prototype reactivity of formally d^4^ configured ruthenium alkylidynes.

In a recent Communication, we reported that *p*‐tolyl(trimethylsilyl)diazomethane (**1**) reacts with [(*p*‐cymene)RuCl_2_]_2_ in acetonitrile to give the chloride‐bridged dinuclear complex **2** with a silyl group on each of the carbene ligands (Scheme 1) [1, 2]. Subsequent exposure to the lithium salt **3** entailed rapid elimination of TMSCl with formation of the ruthenium alkylidyne **4** endowed with a PNP‐pincer ligand. The reactivity of this *formally* d^4^‐configured complex is dominated by the non‐bonding lone pair of significant d_z2_ character, which constitutes the largely metal‐centered HOMO. As a result, **4** engages polarized alkynes such as methyl 2‐butynoate in a stepwise rather than concerted [2+2] cycloaddition to give the metallacyclobutadiene **5**; this transformation resembles an inorganic Michael‐type reaction triggered by attack of the lone pair onto the acceptor substrate, followed by collapse of the resulting dipolar intermediate **A** [1].

Although such a stepwise process is a handicap for cycloreversion and prevents catalytic alkyne metathesis from occurring [1], we saw another opportunity for taking advantage of the peculiar chemical character of ruthenium alkylidynes of this type. Specifically, the high‐lying lone pair should render such complexes susceptible to protonation or regular oxidative addition to the H−Cl bond; the resulting hydride species **B** is expected to undergo facile 1,2‐H shift with formation of a carbene as the final product. Indeed, the alkylidyne complex **4** reacts instantly with ethereal HCl to furnish the corresponding carbene complex **7** in high yield, the constitution of which was confirmed by X‐ray diffraction analysis (the structure is contained in the Supporting Information) [3]. It had been known in the literature that ruthenium alkylidynes such as **9** can be protonated, but the reported example was no more than a “short circuit” of little preparative value, as starting material and product were identical (see the Insert in Scheme 1) [4, 5].

**SCHEME 2:**
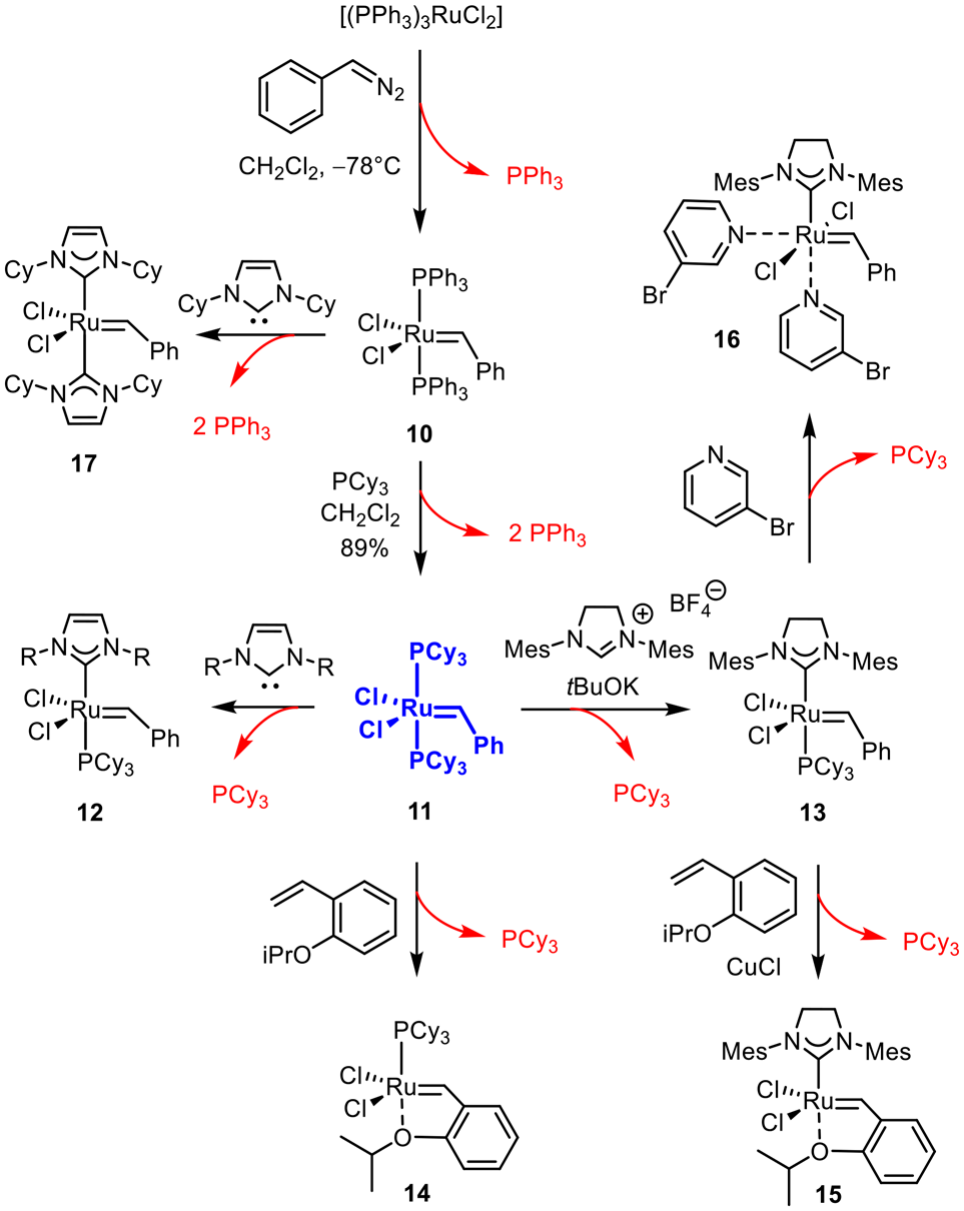
The genesis of the different generations of classical Grubbs‐type catalysts exhibits poor “ligand economy”.

With a new and facile access route to ruthenium alkylidynes in place, however, the situation might change and reward could be drawn from the protonation bias. Provided that the reactivity manifested in the formation of **4** and **7** can be generalized and monodentate phosphines or NHC's be used instead of the PNP pincer ligand, venerable Grubbs‐type catalysts for olefin metathesis might come into reach [6, 7, 8, 9, 10, 11, 12, 13, 14, 15, 16, 17, 18, 19, 20]. The envisaged sequence of alkylidyne formation/protonation should offer notable advantages, which become apparent when considering the genesis of the iconic Grubbs catalysts (Scheme 2). All of them derive from complex **10**, which in turn is obtained by reacting hazardous phenyldiazomethane [21] with [(PPh_3_)_3_RuCl_2_] under cryogenic conditions [22]. Ligand exchange then affords the prototypical first‐generation Grubbs catalyst **11** [22, 23]; already at this stage, all three PPh_3_ ligands of [(PPh_3_)_3_RuCl_2_] have been wasted. Upon conversion of **11** into any of the later generation catalysts exemplified by **12** (R = 2,4,6‐trimethylphenyl) [24, 25, 26], **12** (R = CHMePh) [27], **13** [28, 29], **14** [30, 31], **15** [32, 33], **16** [34], or **17** [35], the “phosphine economy” gets even worse. It is somewhat paradox and arguably disturbing that five equivalents of valuable phosphines must be sacrificed in order to make entirely phosphine‐free catalysts such as **15** or **16**. Moreover, a phosphine scavenger such as stoichiometric CuCl is needed in certain cases. It should be remembered that meticulous purification is imperative since any residual phosphine in a sample of a Grubbs‐type catalyst entails greatly diminished catalytic activity as it will compete with the substrate for the binding to the metal center [7].

## 2 Results and Discussion

Provided that our new phosphine‐free silylated carbene dimer **2** (or analogs thereof) can be used as an alternative entry point, these problems might get solved [36, 37, 38, 39, 40, 41, 42, 43, 44, 45, 46, 47]. The fact that we had already managed in our previous study to prepare multigram quantities of complex **2** made us confident that scalability is a realistic outlook [1]. Actually, **2** is very easy to make: it precipitates from the mixture when [(*p*‐cymene)RuCl_2_]_2_ is reacted with the silylated diazo derivative **1** in MeCN at ambient temperature and can therefore be isolated by simple filtration; moreover, this complex is bench‐stable when kept dry and can be weighed and handled in air. Likewise, the required carbyne donor **1** is available in only three high‐yielding operations on multi‐gram scale starting from 4‐methylbenzoic acid chloride (for details, see the Supporting Information) [48, 49]. Arguably most important, however, is the fact that this particular diazo derivative and its analogues are safe to use due to the massive stabilizing effect of the silyl group. While we found the crude product **1** (Ar = *p*‐tolyl) pure enough for direct use, it can be distilled in vacuo if one desires so (58°C–61°C, 0.2 mm) [49]; its sibling (Ar = Ph) is reported to survive even at temperatures of up to 135°C (0.3 mm) [2, 50]. This remarkable thermal stability distinguishes **1** from phenyldiazomethane used en route to the classical Grubbs carbenes, which has to be handled with the greatest care and must be reacted at cryogenic conditions (Scheme 2) [22, 51]. Moreover, and again in stark contrast to phenyldiazomethane, compound **1** can be stored for months in a refrigerator without notable decomposition.

**SCHEME 3:**
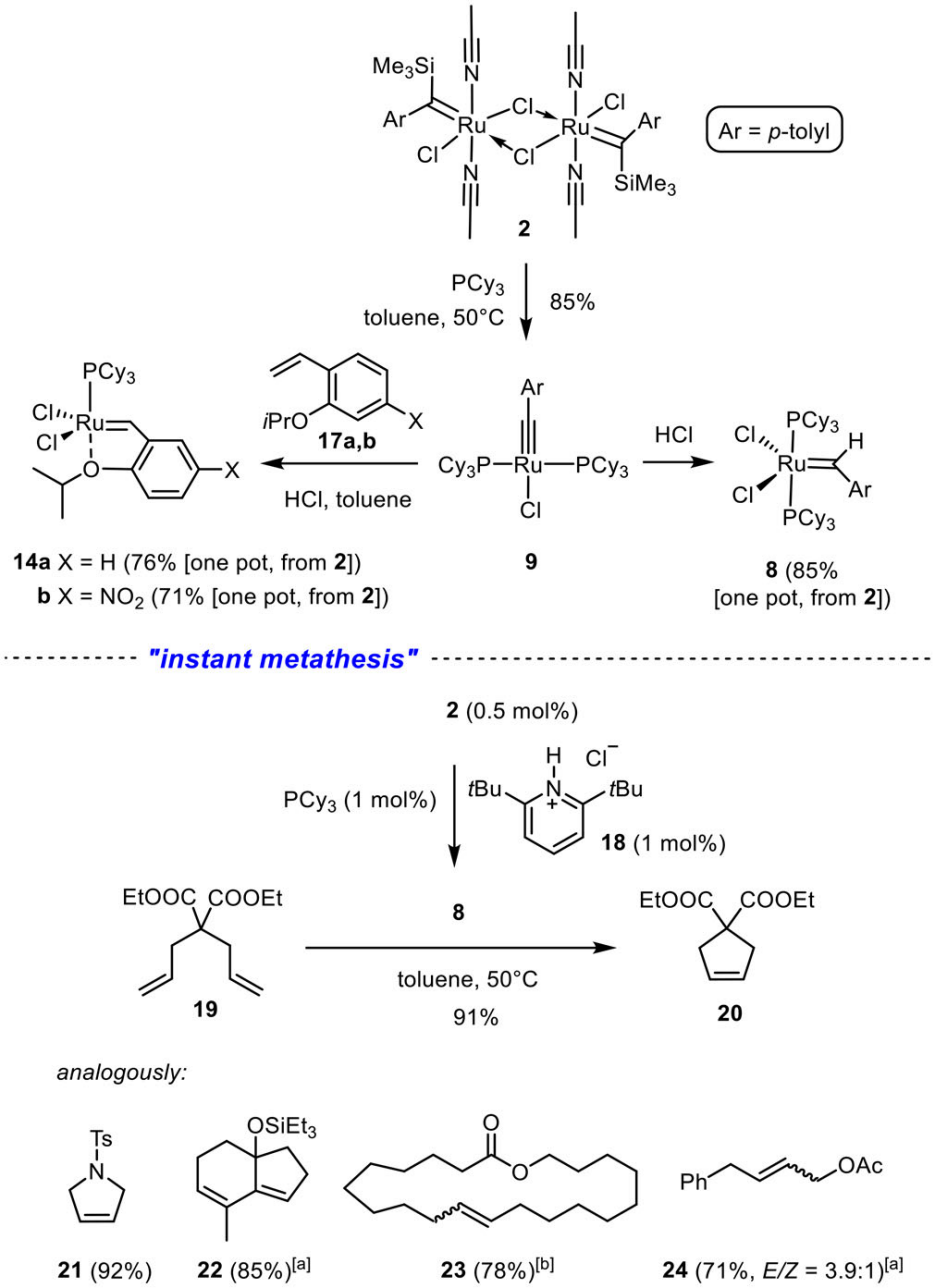
New route to “first generation” Grubbs‐ and Grubbs/Hoveyda‐type catalysts and an application to “instant” metathesis reactions; ^[a]^ with 2.5 mol% of **2**; ^[b]^ with 5 mol% of **2**.

**FIGURE 1:**
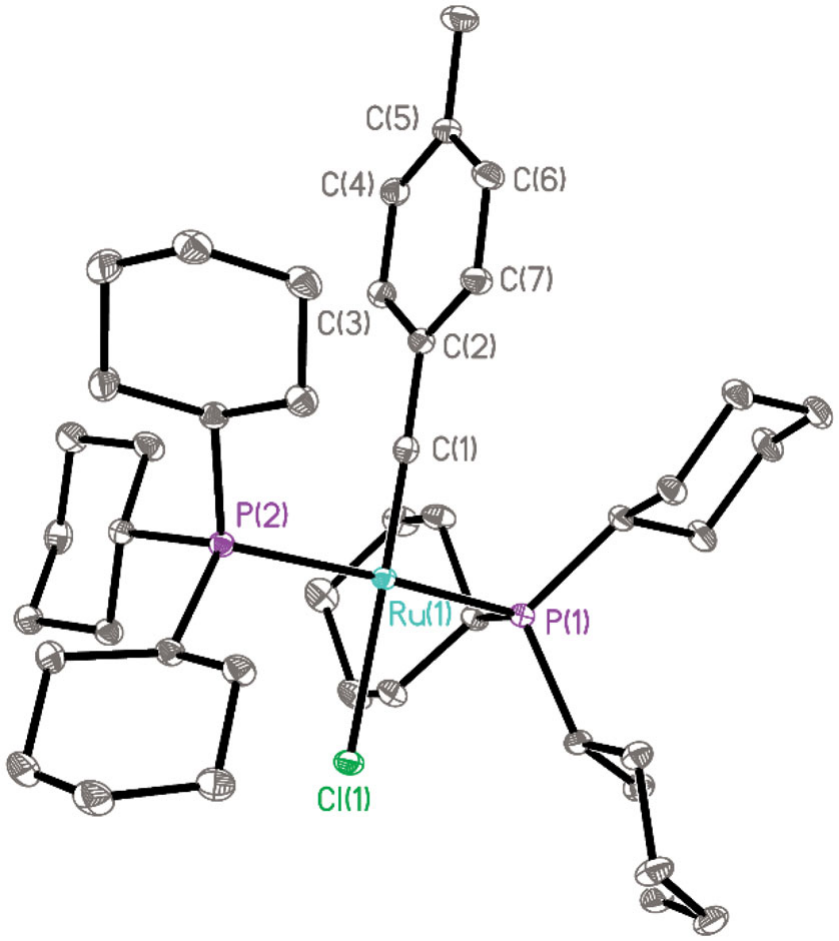
Structure of the four‐coordinate ruthenium alkylidyne **9** in the solid state (thermal ellipsoids at the 40 % probability level); hydrogen atoms and solute toluene in the unit cell omitted for clarity (the full structure is contained in the Supporting Information). Selected bond lengths (Å) and angles (°): Ru1‐Cl1 2.4357(4), Ru1‐P1 2.3845(4), Ru1‐P2 2.3878(4), Ru1‐C1 1.7103(16), C1‐C2 1.445(2); C1‐Ru1‐Cl1 178.12(5); Ru1‐C1‐C2 178.20(13).

Just like in the case of the pincer ligand leading to **4** [1], addition of PCy_3_ to a suspension of **2** in toluene causes elimination of TMSCl to give the corresponding four‐coordinate ruthenium alkylidyne complex **9** in good yield (Scheme 3). This compound is known in the literature, but had been prepared by deprotonation of the Grubbs catalyst **8** with a particular germylene as the optimal base (see Insert in Scheme 1) [4, 5]. The spectroscopic data of our sample were fully matching, with the deshielded signal of the alkylidyne C‐atom being most characteristic (δ_C_ = 237.8 ppm, *t*, *J*_C,P_ = 18.4 Hz). However, crystals suitable for X‐ray diffraction analysis had not been attained in the past. Figure 1 shows the structure of **9** in the solid state [3]. As expected, the two phosphines are *trans* to each other within the square‐planar coordination sphere of the Ru‐center. The Ru(1)−C(1) bond length of 1.7103(16) Å compares well to that of the few other known (mostly higher valent) ruthenium alkylidynes [1, 5, 52, 53, 54, 55, 56], and the Ru(1)−Cl(1) bond (2.4357(4) Å) is slightly elongated as one might expect due to the strong *trans*‐effect of the alkylidyne ligand. In close analogy to complex **26** discussed below, the HOMO of the complex is a high‐lying metal‐centered lone pair of significant d_z2_ character (for details, see the Supporting Information).

Addition of ethereal HCl to a solution of **9** in toluene resulted in the virtually quantitative formation of the “first generation” Grubbs‐type catalyst **8**. Its preparation can be further streamlined in that the ruthenium alkylidyne **9** does not even have to be isolated. Rather, it suffices to stir a mixture of **2** and PCy_3_ in toluene at 50°C for 30 min and then add, for example, 2,6‐di‐*tert*‐butylpyridinium chloride (**18**) as a convenient, crystalline source of HCl to the mixture [57]. Under these conditions, complex **8** was cleanly formed and isolated in 85% yield by filtration, evaporation of the solvent, and washing of the crude product with MeOH. An extension to the formation of the “first generation” Grubbs‐Hoveyda type catalyst **14a** or the corresponding nitro‐substituted variant **14b** is equally straightforward: [30, 58] all it takes is to stir a mixture of **2**, PCy_3_(1 equiv.), the corresponding styrene **17a**,**b** and HCl in toluene to obtain these popular catalysts in good yield each. It is important to note that no phosphine is wasted in either case, which is in marked contrast to the literature routes.

Moreover, **8** can be generated in the presence of an olefinic substrate. As shown by the examples compiled in Scheme 3 (bottom), the yields obtained by this “instant metathesis” procedure are perfectly comparable to those reported in the literature for the exact same products **20–24** [59, 60, 61, 62, 63, 64] using authentic first‐generation Grubbs catalyst.

**SCHEME 4:**
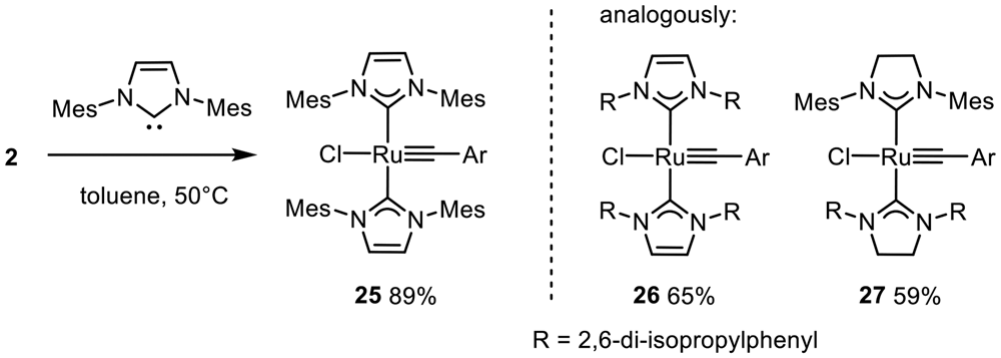
Ruthenium alkylidyne complexes endowed with two NHC ligands.

**FIGURE 2:**
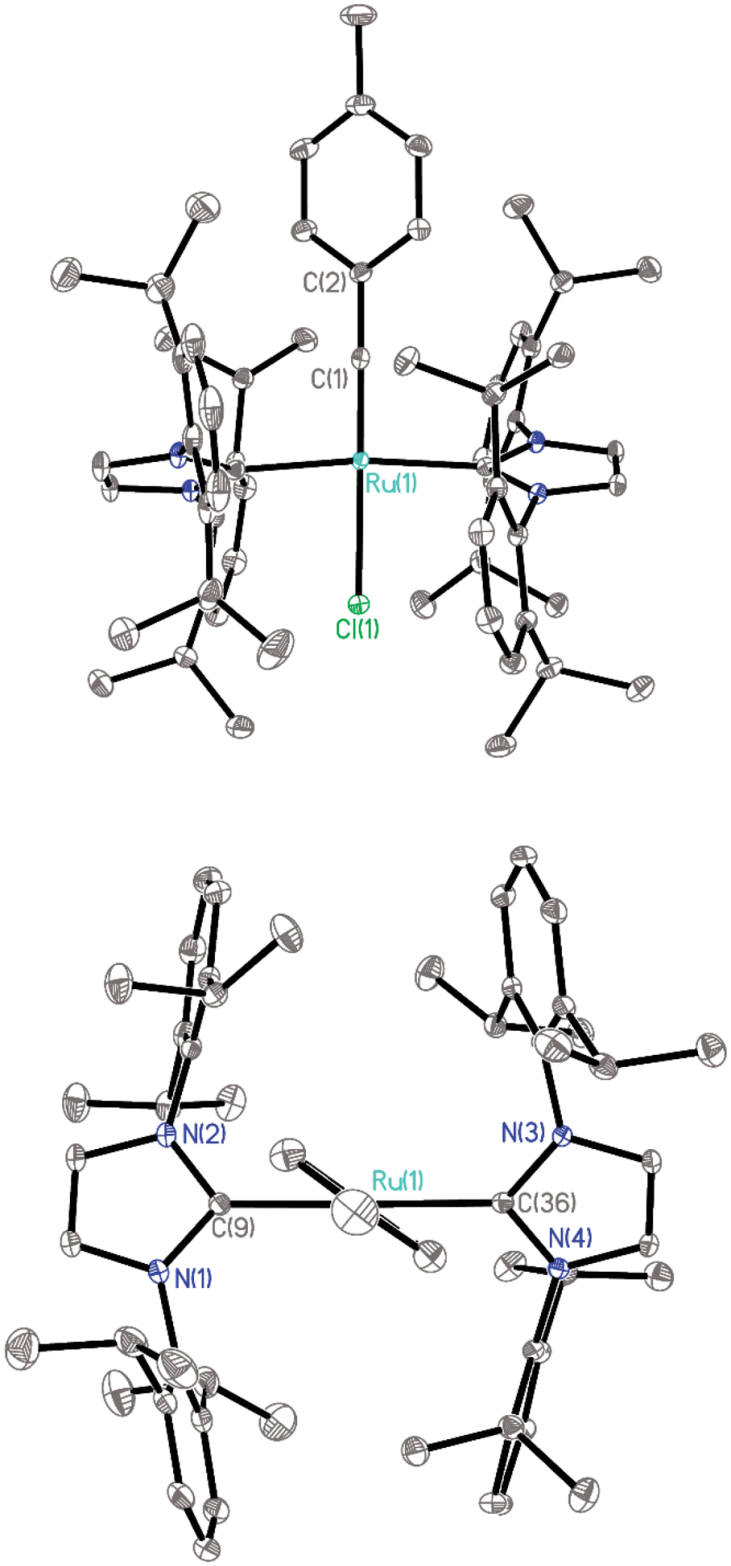
Structure of complex **26** in the solid state (thermal ellipsoids at the 40 % probability level) shown in front‐ and top view to illustrate the congested coordination sphere; H‐atoms and solute *n*‐pentane in the unit cell omitted for clarity. Selected bond lengths (Å) and angles (°): Ru1‐C1 1.7178(13), Ru1‐Cl1 2.4199(3), C1‐C2 1.4456(19), Ru1‐C9 2.1076(13), Ru1‐C36 2.1106(13), Cl1‐Ru1‐C1 178.67(5), C9‐Ru1‐C36 172.76(5), Ru1‐C1‐C2 178.32(11); plane C9‐N1‐N2 to plane C36‐N3‐N4 twist angle: 40.18(6).

**FIGURE 3:**
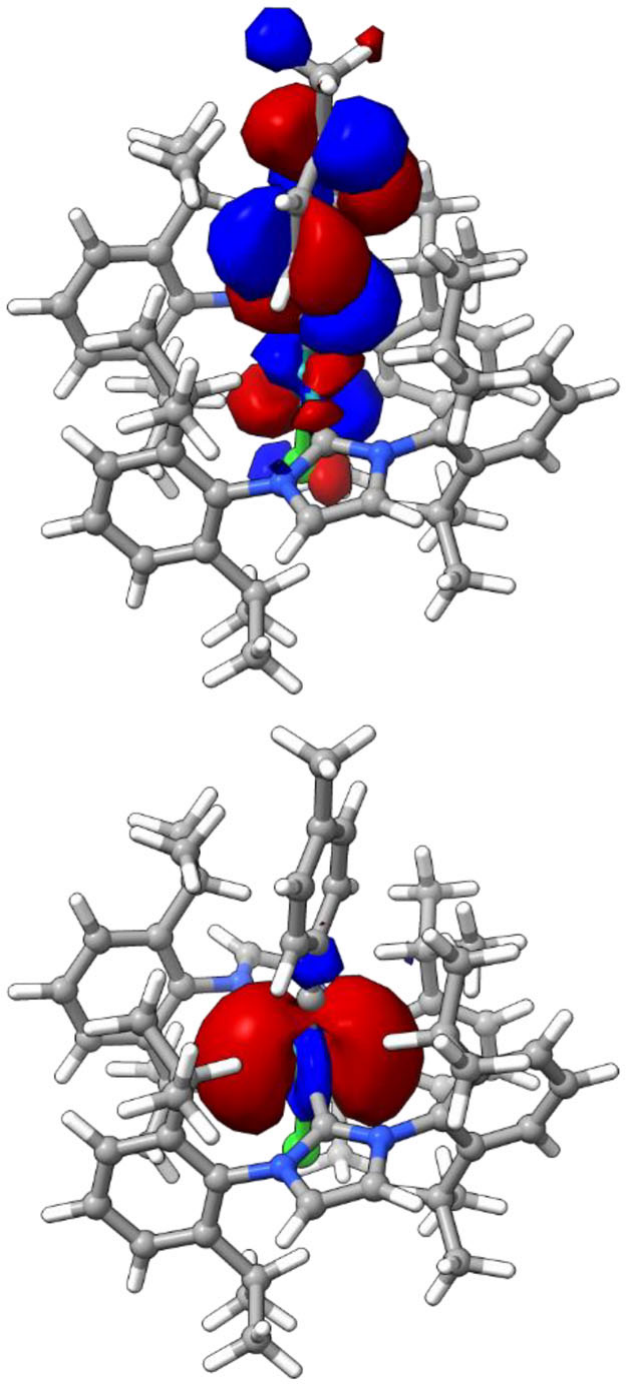
LUMO (top) and HOMO (bottom) of the four‐coordinate ruthenium alkylidyne complex **26** endowed with two NHC ligands; the full frontier MO diagram is contained in the Supporting Information.

Next, it was shown that the reaction of **2** with various N‐heterocyclic carbenes (NHC) instead of PCy_3_ also entails the formation of the corresponding ruthenium alkylidynes [65], as evident from the characteristic signals of their alkylidyne C‐atoms (**25**: δ_C_ = 229.3 ppm; **26**: δ_C_ = 222.9 ppm; **27**: 229.3 ppm) (Scheme 4). In case of **26**, the assignment was confirmed by X‐ray diffraction analysis, which proved the regular square planar coordination geometry of this complex (Figure 2) [3]. At the same time, however, the very congested environment about the ruthenium center becomes apparent, in that the planes of the two imidazol‐2‐ylidene rings are notably tilted against each other, and the alkylidyne “intercalated” in between them is also twisted in order to find enough space. DFT studies at the B3LYP‐D4/def2‐tzvp CPCM(toluene) level of theory [66, 67, 68, 69, 70, 71] using the ORCA 6.0 program package [72] confirmed that **26** has a high‐lying, largely metal‐centered lone pair of significant d_z2_ character representing the HOMO of the complex (Figure 3). As expected, the strongly σ‐donating NHC ligands evidently render it highly electron‐rich and hence protonation‐prone.

**SCHEME 5:**
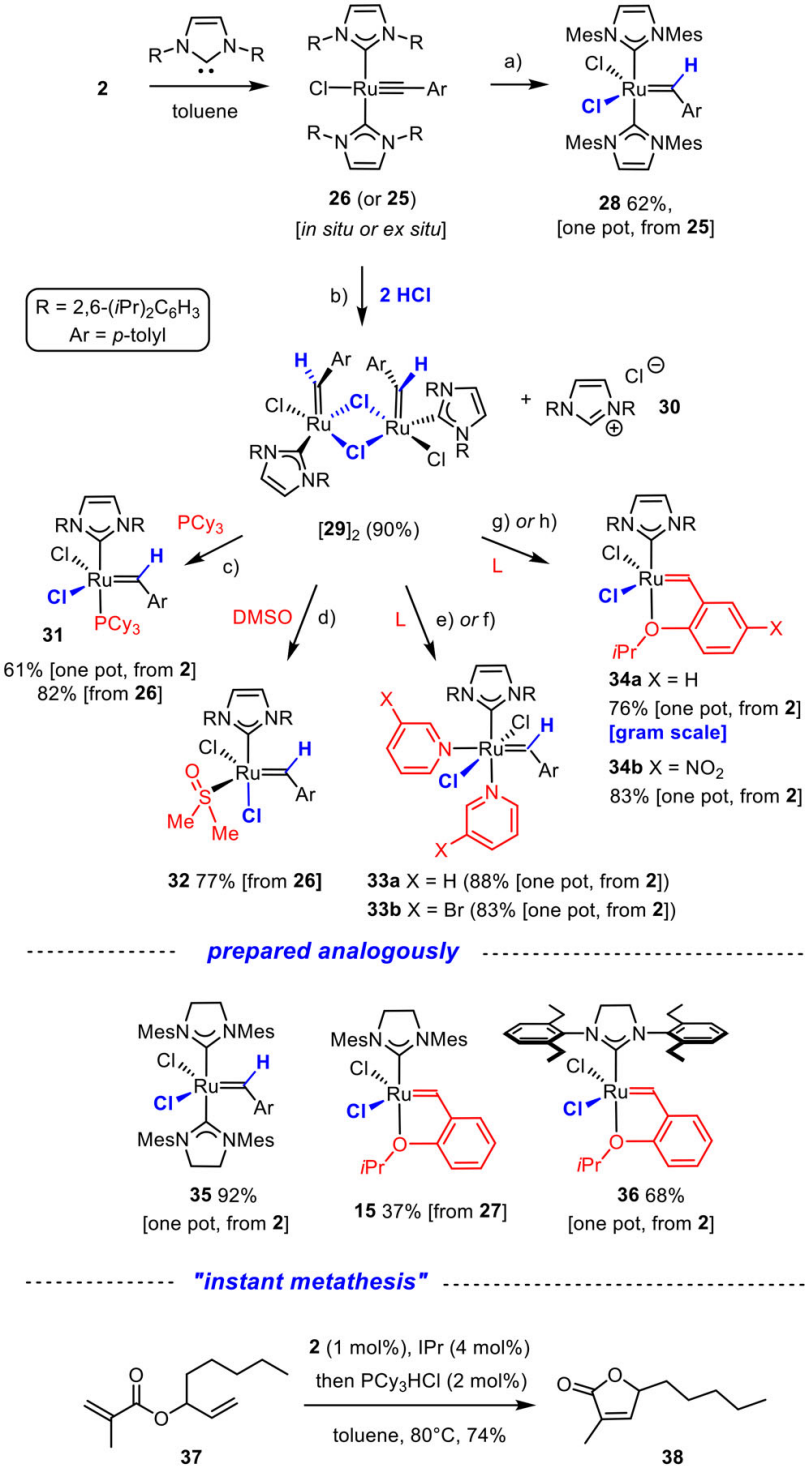
A portfolio of Grubbs‐type catalysts, all derived from ruthenium alkylidynes endowed with two NHC ligands: (a) 2,6‐lutidinium chloride, toluene; (b) HCl in 1,4‐dioxane (4 M), toluene; (c) HCl in Et_2_O (0.5 M), PCy_3_, toluene; (d) HCl in Et_2_O (0.5 M), DMSO, toluene; (e) IPr, toluene, 50°C, then pyridinium hydrochloride; (f) IPr, toluene, 50°C, then HCl in 1,4‐dioxane (4 M), 3‐bromopyridine; (g) HCl in Et_2_O (0.5 M), 1‐isopropoxy‐2‐vinylbenzene (**17a**), toluene, 50°C; h) IPr, toluene, 50°C, then 1‐isopropoxy‐4‐nitro‐2‐vinylbenzene, HCl in 1,4‐dioxane (4 M); IPr = 1,3‐bis(2,6‐diisopropylphenyl)imidazol‐2‐ylidene.

**FIGURE 4:**
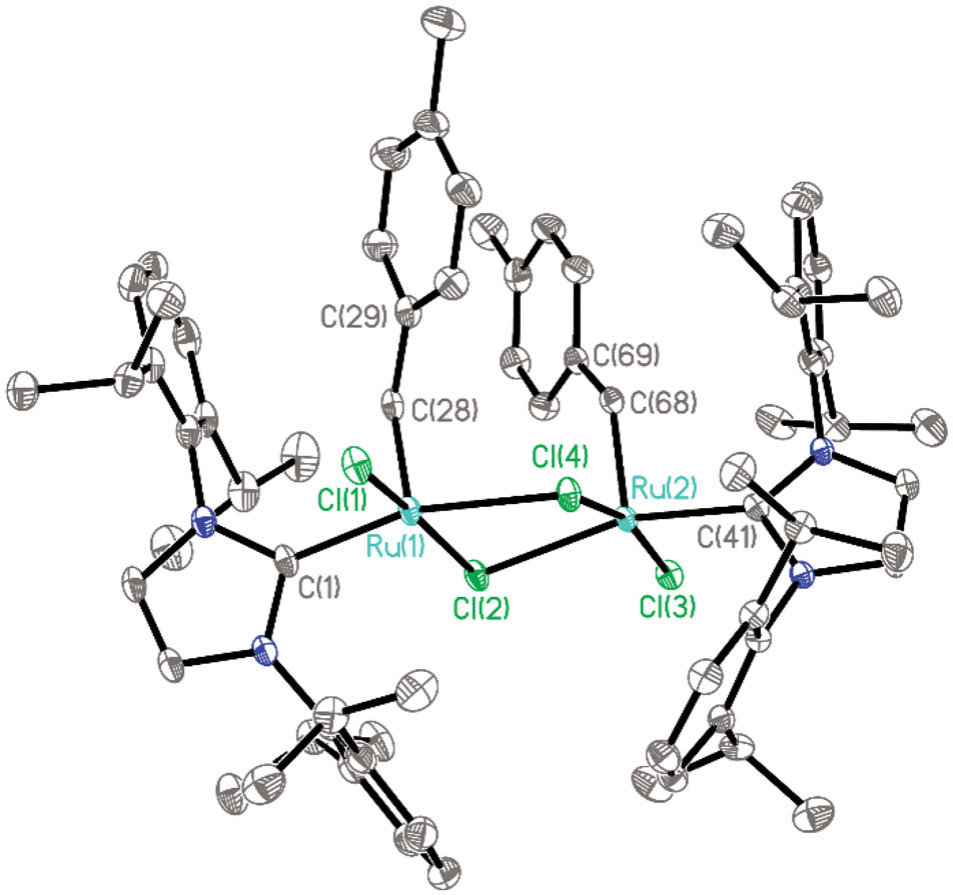
Structure of complex [**29**]_2_ in the solid state (thermal ellipsoids at the 40% probability level); H‐atoms and solute CH_2_Cl_2_ and *n*‐hexane in the unit cell omitted for clarity. Selected bond lengths (Å) and angles (°): Ru1‐C28 1.829(3), Ru1‐C1 2.004(3), Ru2‐C68 1.833(3), Ru2‐C41 2.004(3), C28‐C68 6.17(7); Ru1‐C28‐C29 129.8(2), Ru2‐C68‐C69 130.5(2); C28‐Ru1‐Ru2‐C68 16.54(14).

In line with this notion, addition of one equivalent of HCl selectively hits this lone pair; the transient hydride species instantly converts into the corresponding “latent” metathesis catalysts, as demonstrated by the preparation of **28** and **35** (Scheme 5) [35]. Addition of a second equivalent of HCl then protonates one of the NHC ligands off, causing the precipitation of the corresponding imidazolium salt **30**, which can be collected by filtration and re‐converted into the NHC upon treatment with an appropriate base. The formally four‐coordinate ruthenium carbene concomitantly formed (if one disregards the weakly coordinating solvent) is dimerization‐prone; in any case, the DOSY data speak for a dimer in solution (see the Supporting Information) and the X‐ray diffraction analysis confirmed the presence of the chloride‐bridged dinuclear species [**29**]_2_ in the solid state (Figure 4) 3. Somewhat surprisingly, this complex differs from related dimers known in the literature [73, 74, 75] in that the two 4‐methylbenzylidene units are oriented *cis* rather than *trans* to each other. This peculiar spatial arrangement may explain why [**29**]_2_ is primed for decomposition by bimolecular coupling of the carbene units in solution [76, 77, 78]. In the solid state, however, the complex can be kept even at ambient temperature for ≥ 1 month without significant decomposition; it may therefore serve as a valuable fast‐initiating catalyst [75].

When PCy_3_ is present in the reaction mixture while forming [**29**]_2_ in this way, the “second generation” Grubbs catalyst **31** is directly obtained in good yield [26]. Once again, **31** can be generated in situ in the presence of an olefin to be metathesized, thus extending the scope of the “instant procedure” to reactions requiring such “second generation” catalysts. The RCM reaction leading to the trisubstituted butenolide **38** shown in Scheme 5 is representative (for an additional example, see the Supporting Information) [79].

The ability to form heteroleptic complexes is by no means limited to the use of phosphines. Rather, dimethylsulfoxide can also be introduced to give complex **32**, which is *cis*‐configured and has precedent in the olefin metathesis literature [80]. Likewise, “third generation” Grubbs type complexes such as **33a**,**b** are easily reached by performing the protonation of **26** with the appropriate pyridinium hydrochloride as source of HCl and the donor ligand alike. Complexes of this type are able to metathesize acrylonitrile [34]; as particularly fast‐initiating catalysts [81], they are also enabling tools for material science [82, 83]. Protonation of **26** in the presence of stoichiometric 2‐isopropoxystyrene triggers a cross metathesis reaction to furnish the corresponding “second generation” Grubbs‐Hoveyda type catalyst **34a** in high yield on gram scale; of course, the analogous nitro‐Grela catalyst **34b** is equally within reach as are the analogous complexes **15** and **36** bearing saturated NHC ligands. In no case is any phosphine wasted, the use of phosphine scavengers is unnecessary, and all catalyst samples prepared by this route are hence free of residual PR_3_ that might interfere with their activity. Moreover, the ability to quickly and cleanly generate different ruthenium carbene complexes starting directly from **2** in combination with the “instant metathesis” procedure that allows their catalytic performance to be assessed in situ should lend itself to parallel screening and reaction optimization purposes [84].

**FIGURE 5:**
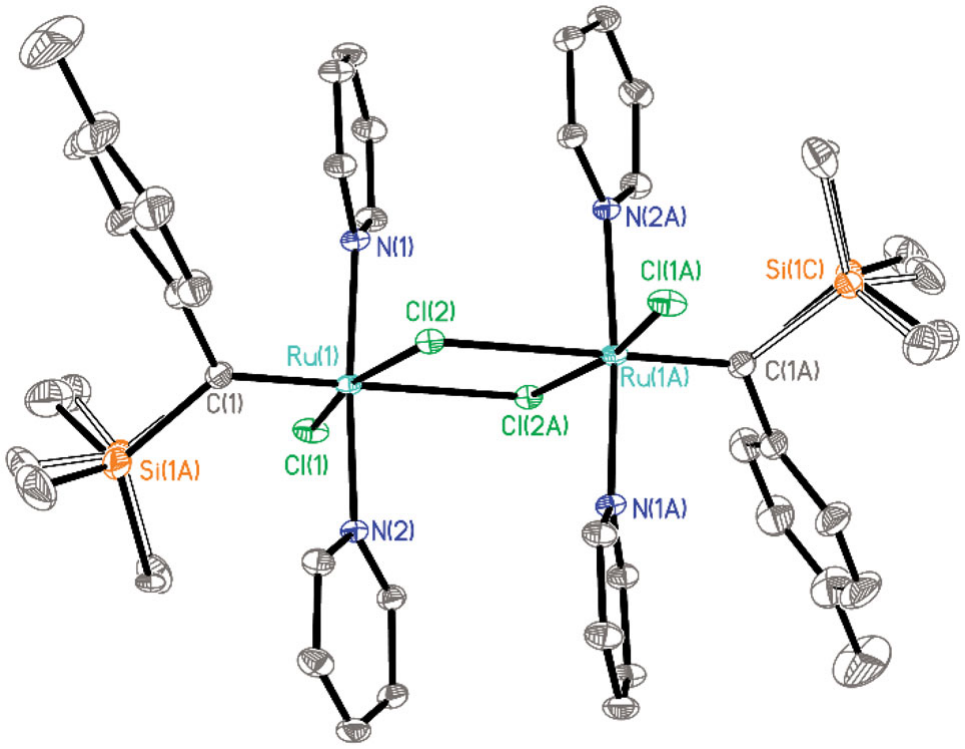
Structure of complex 40 in the solid state (thermal ellipsoids at the 40% probability level); H‐atoms omitted for clarity. Selected bond lengths (Å): Ru1‐C1 1.8678(17), C1‐Si1A 1.940(10), Ru1‐Cl2A 2.6530(4).

**SCHEME 6:**
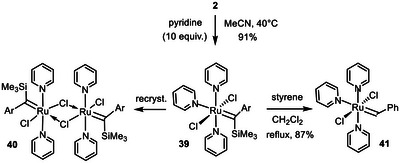
Ligand exchange with formation of the Ru benzylidene complex **41** exclusively ligated to pyridine.

**FIGURE 6:**
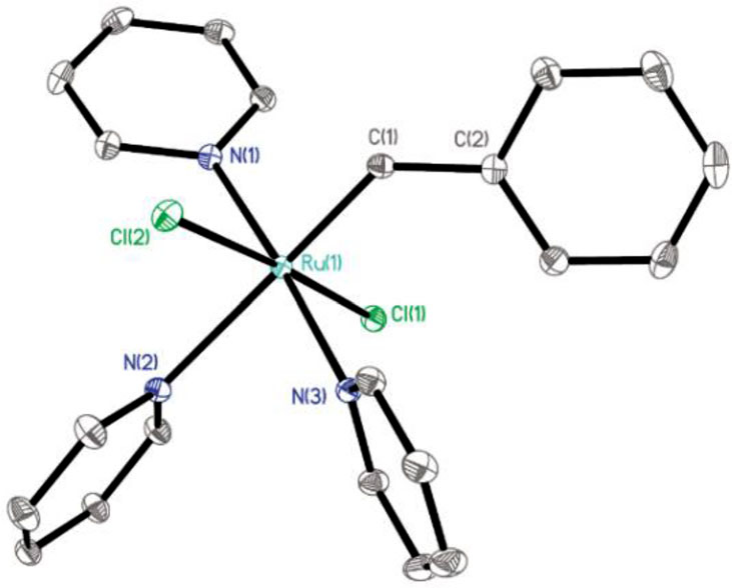
Structure of complex trans‐**41** in the solid state; H‐atoms omitted for clarity. Selected bond lengths (Å): Ru1‐C1 1.868(2), Ru1‐N2 2.3268(19), Ru1‐N1 2.0891(19), Ru1‐N3 2.0931(19).

Finally, it is worth noting that exchange of the labile acetonitrile ligands of **2** for other ligands is also possible without concomitant formation of an alkylidyne unit by elimination of TMSCl. This provides opportunities to access entirely new types of ruthenium carbene complexes. Thus, addition of excess pyridine to a solution of **2** in MeCN left the silylated carbene unit intact. In contrast to the dinuclear acetonitrile adduct **2**, the resulting adduct **39** is well soluble in common organic solvents such as toluene and dichloromethane. In solution, it consists of a mixture of two diastereomers (*trans*:*cis* ≈ 95:5, NOE); the exceptionally deshielded carbene signal of the major isomer at δ_C_ = 403 ppm is noteworthy. Recrystallization of the crude product from CH_2_Cl_2_/*n*‐hexane did not cause TMSCl‐elimination either but gave single crystals suitable for X ‐ray diffraction of the *cis*‐isomer (see the Supporting Information) [85] as well as the chloride‐bridged dimer **40** (Figure 5) [3]. Interestingly, complex **39** is capable of entertaining a high yielding stoichiometric metathesis reaction with styrene to give the benzylidene complex **41**, which exists as a *cis/trans*‐mixture in CD_2_Cl_2_ solution (Scheme 6); single crystals of the *trans*‐isomer suitable for X‐ray diffraction analysis were obtained (Figure 6) [3]. Attempts at using complexes **39** or **40** as catalysts were so far largely met with failure as the labile and unencumbered pyridine ligands are apparently incapable of stabilizing the reactive carbene unit (or the derived methylidene species) to the necessary extent [86].

## 3 Conclusions

In summary, we outline a novel, practical, flexible and high‐yielding entry into a large portfolio of Grubbs‐type ruthenium carbene complexes for use in olefin metathesis. From the conceptual viewpoint, the new route is distinguished by the fact that it passes through ruthenium alkylidynes in the first place and takes advantage of their propensity to undergo protonation, which is rooted in the high‐lying lone pair at the formally d^4^‐configured ruthenium center. In practical terms, the new procedure excels with regard to its flexibility as well as an unmatched “ligand economy”; this virtue stands in marked contrast to the classical literature routes, where up to five equivalents of valuable phosphines have to be sacrificed for the preparation of what are ultimately phosphine‐free catalysts. Since the required ruthenium alkylidynes themselves are readily accessible with the aid of silylated aryldiazomethane derivatives as stable, safe and readily accessible carbyne donor reagents, the new method is deemed to mark an important advance in the field. Further studies in this laboratory are underway to fully exploit the opportunities that it provides.

## Conflicts of Interest

Patent applied for.

## Supporting information




**Supporting File 1**: anie71722‐sup‐0001‐SuppMat.pdf.

## Data Availability

The data that supports the findings of this study are available in the supplementary material of this article.
