# Supplementary material for: A Convenient, Safe, and Atom‐Economical Route to a Large Portfolio of Grubbs‐Type Catalysts for Olefin Metathesis via Four‐Coordinate Ruthenium Alkylidynes
Source: Angew Chem Int Ed Engl. 2026 Mar 4;65(16):e6803845. doi: 10.1002/anie.6803845 (PMC13080428; doi:10.1002/anie.6803845)
Supplement: Supplementary file 1 — Supporting File 1: anie71722‐sup‐0001‐SuppMat.pdf. [file ANIE-65-e6803845-s001.pdf]

SUPPORTING INFORMATION

A Convenient, Safe and Atom-Economical Route to a Large Portfolio of Grubbs-type Catalysts for Olefin Metathesis via Four-Coordinate Ruthenium Alkylidynes

Mingxu Cui, and Alois Fürstner*

Max-Planck-Institut für Kohlenforschung, 45470 Mülheim/Ruhr (Germany)

Email: fuerstner@kofo.mpg.de

Table of Content

Experimental Part	S2
General	S2
NHC Ligands and Pyridinium Salts	S3
Preparation of the Carbyne Synthon	S3
Ruthenium Carbene and Alkylidyne Complexes	S5
„Instant Procedure“ for Catalytic Olefin Metathesis	S16
Supporting Crystallographic Information	S19
Computational Study	S57
xyz Coordinates of Computed Structures	S59
NMR and IR Spectra	S64
References	S107

Experimental Part

General. All reactions were carried out under argon in flame-dried glassware using standard Schlenk techniques unless stated otherwise.[1] Air-sensitive compounds were stored and transferred under Ar. The solvents were purified by distillation over the drying agents indicated and were transferred under Ar: tetrahydrofuran (magnesium/anthracene), toluene ($NaAlEt_4$), benzene (CaH_2), dichloromethane (CaH_2), acetonitrile (CaH_2), diethyl ether (Na/K), *n*-hexane (Na/K), *n*-pentane (Na/K) and methanol (Mg/I_2). Deuterated solvents were degassed by three freeze-pump-thaw cycles and were then stored over molecular sieves (3 Å). The molecular sieves were dried for 24 h at 150°C (sand bath) under vacuum (10^{-3} mbar) prior to use and were stored and transferred under Ar. All commercially available compounds (abcr, BLD, Aldrich, TCI) were used as received, unless stated otherwise.

NMR spectra were acquired on a Bruker Avance III 400 MHz, 500 MHz, or an Avance Neo 600 MHz NMR (equipped with a Bruker BBO CryoProbe) spectrometer in the solvent indicated. Chemical shifts (δ) are given in ppm relative to peaks of residual undeuterated solvent ($CDCl_3$: δ_H = 7.26 ppm; δ_C = 77.16 ppm; CD_2Cl_2: δ_H = 5.32 ppm; δ_C = 53.84 ppm; C_6D_6: δ_H = 7.16 ppm; δ_C = 128.06 ppm; [D_8]-toluene: δ_H = 2.09 ppm; δ_C = 20.43 ppm; [D_3]-MeCN: δ_H = 1.94 ppm; δ_C = 1.32 ppm).[2] $^{31}P\{^1H\}$ chemical shifts are reported relative to 85% H_3PO_4 (δ = 0 ppm; Ξ = 40.480742%). 1D ^{29}Si NMR spectra were acquired with a polarization transfer pulse sequence (refocused INEPT) and broadband proton decoupling. ^{29}Si chemical shifts were referenced indirectly to the ^1H chemical shift of the solvent according to IUPAC recommendations using the xiref macro in Bruker Topspin. ^{29}Si chemical shifts are reported relative to TMS (δ = 0 ppm; Ξ = 19.867187%). Coupling constants (*J*) are given in Hz.

IR spectra were recorded on an ALPHA (Bruker) FT-IR spectrometer with an ATR unit; the signals are given in wavenumbers (ṽ) in cm^{-1}.

HRMS data were recorded on a Bruker APEX III FT-MS (7T magnet), MAT 95 (Finnigan), Thermo Scientific LTQ-FT, or Thermo Scientific Exactive Spectrometer.

Elemental analyses were carried out by Mikroanalytisches Laboratorium Kolbe, Oberhausen, Germany.

NHC Ligands, Substituted Styrene Derivatives, and Pyridinium Salt

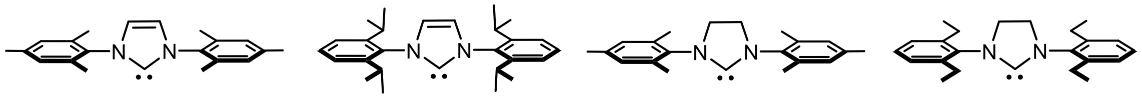

IMes IPr SIMes SIDep

1,3-Di(2′,6′-diisoproplyphenyl)-imidazol-2-ylidene (IPr),[3] 1,3-di(2′,4′,6′-trimethylphenyl)-imidazol-2-ylidene (IMes),[4] 1,3-di(2′,4′,6′-trimethylphenyl)-imidazolin-2-ylidene (SIMes),[4] 1,3-di(2′,6′-diethylphenyl)-imidazolin-2-ylidene (SIDep),[5] 1-isopropoxy-2-vinylbenzene,[4] and 1-isopropoxy-4-nitro-2-vinylbenzene[6] were prepared according to literature procedures.

Pyridinium chloride, 2,6-lutidinium chloride, 2,6-di-*tert*-butylpyridinium chloride and tricyclohexylphosphonium chloride were prepared from HCl solution (4 M in 1,4-dioxane) and the corresponding pyridine (derivative) or phosphine by modified literature procedures.[7,8]

Preparation of the Carbyne Synthon

***p*-Tolyl(trimethylsilyl)methanone (S1)**. A Schlenk flask equipped with a stir bar was charged with $[Pd(C_3H_5)Cl]_2$ (229 mg, 0.62 mmol), triethyl phosphite (0.415 g, 2.5 mmol) and Si_2Me_6 (3.95 g, 27.0 mmol). The resulting mixture was stirred for 5 min to give a pale-yellow suspension. Freshly distilled *p*-methylbenzoyl chloride (3.87 g, 25.0 mmol) was then added to the suspension, forming a clear yellow solution. The mixture was stirred at 110 °C (bath temperature) for 14 h. The mixture was filtered through a plug of Celite to remove Pd black, which was rinsed with pentane. The combined filtrates were evaporated using a rotary evaporator. The residue was purified by short-path vacuum distillation (b.p. 53-57°C at 10^{-3} atm; oil bath temperature ≈90 °C) to yield 2.64 g of *p*-tolyl(trimethylsilyl)methanone as a yellow oil. Pentane was added to the residue, precipitating a pale gray solid from an orange solution. The orange solution was separated from the precipitate by filtration. The filtrate was evaporated and the residue subjected again to Kugelrohr distillation (110°C at 10^{-3} bar; the temperature must not exceed 125°C to avoid decomposition), yielding another 1.55 g of the product. Total yield: 4.19 g, 87%. ^{1}H NMR (400 MHz, $CDCl_3$) δ 7.75 (app d, J = 8.1 Hz, 2H), 7.27 (app d, J = 7.9 Hz, 2H), 2.41 (s, 3H), 0.37 (s, 9H). $^{13}C\{^1H\}$ NMR (101 MHz, $CDCl_3$) δ 235.0, 143.6, 139.3, 129.5, 127.8, 21.8, −1.2.

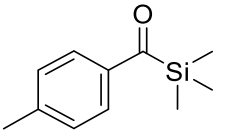

The spectroscopic data match the literature.[9]

{Pd[C(O)-*p*-tolyl]P(OEt)$_3$}$_2$(*μ*-Cl)$_2$ (S2). The pale gray solid was dissolved in THF, insoluble residues were filtered off, and the filtrate was layered with hexane. Colorless crystals were deposited after one week at ambient temperature, which were collected and dried under vacuum to give the title complex as pale yellow crystals (0.129 g, 24% (based on Pd)). ^{1}H NMR (400 MHz, $CDCl_3$) δ 8.04 (app d, *J* = 7.9 Hz, 4H), 7.19 (app d, *J* = 7.8 Hz, 4H), 4.27 – 3.98 (m, 12H), 2.37 (s, 6H), 1.18 (t, *J* = 7.1 Hz, 18H). ^{13}C{^{1}H} NMR (151 MHz, $CDCl_3$) δ 213.3 (d, *J* = 25.3 Hz), 142.8, 136.5 (d, *J* = 34.8 Hz), 130.6, 128.7, 62.2, 21.8, 16.2 (d, *J* = 6.5 Hz). ^{31}P{^{1}H} NMR (243 MHz, $CDCl_3$) δ 108.8.

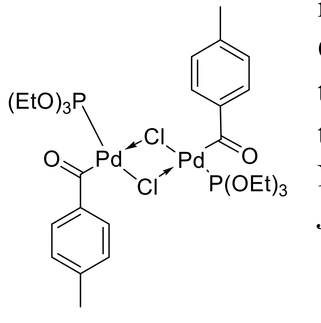

Single crystals suitable for X-ray diffraction analysis were grown by layering a solution of the compound in THF with *n*-hexane.

p*-Tolyl(trimethylsilyl)methanone tosylhydrazone (S3)**. ***Note: *This reaction was conducted in air*. MeOH (2 mL) was added at room temperature to a vigorously stirred suspension of toluene-*p*-sulfonyl hydrazide (3.725 g, 20 mmol) and *p*-tolyl(trimethylsilyl)methanone (3.847 g, 20 mmol). The suspension initially turned clear within minutes before it solidified in seconds while the yellow color faded away. ***Note***: *The reaction is exothermic*. Once the yellow color disappeared, the flask was immediately immersed in an ice bath. The resulting white solid material was ground into a powder, washed with cold MeOH (2 × 5 mL) and dried under vacuum to give the title compound as a white solid (5.214 g, 72%). ^{1}H NMR (400 MHz, $CDCl_3$) δ 7.78 (app d, *J* = 8.3 Hz, 2H), 7.67 (br s, 1H), 7.31 (app d, *J* = 8.1 Hz, 2H), 7.20 (app d, *J* = 7.7 Hz, 2H), 6.74 (app d, *J* = 8.1 Hz, 2H), 2.45 (s, 3H), 2.36 (s, 3H), 0.08 (s, 9H). ^{13}C{^{1}H} NMR (101 MHz, $CDCl_3$) δ 168.7, 144.0, 138.9, 135.8, 131.5, 130.4, 129.5, 128.1, 126.0, 21.8, 21.4, −2.2.

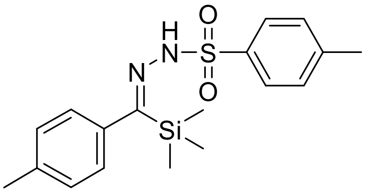

The spectroscopic data match the literature.[10,11]

***p*-Tolyl(trimethylsilyl)diazomethane (1).**[10] A three-necked flask equipped with a condenser was charged with *p*-tolyl(trimethylsilyl)methanone tosylhydrazone (5.000 g, 13.9 mmol) and THF (45 mL). The mixture was cooled to 0 °C before *n*BuLi (1.6 M, 9.1 mL, 14.6 mmol) was added dropwise. Once the addition was complete, the mixture was stirred at 0 °C for 1 h. The volatile materials were removed under vacuum. The residue was suspended in hexane (100 mL) and the resulting mixture was stirred at reflux temperature (80°C bath temperature) for 3 h. The reaction was quenched with pentane (technical grade, stored in air; ca. 16 ppm water (Karl Fischer titration)) at 0 °C. The red solution was filtered through a plug of Celite to remove inorganic salts, and the filtrate was evaporated on a rotary evaporator to afford the title compound as a red oil (2.711 g, 96%). ^{1}H NMR (400 MHz, $CDCl_3$) δ 7.14 – 7.08 (m, 2H), 6.92 (app d, *J* = 8.2 Hz, 2H), 2.31 (s, 3H), 0.34 (s, 9H). ^{1}H NMR (400 MHz, CD_2Cl_2) δ 7.14 – 7.09 (m, 2H), 6.91 (app d, *J* = 8.2 Hz, 2H), 2.29 (s, 3H), 0.33 (s, 9H). ^{1}H NMR (400 MHz, C_6D_6) δ 6.96 (s, 4H), 2.11 (s, 3H), 0.13 (s, 9H). ^{13}C{^{1}H} NMR (101 MHz, $CDCl_3$) δ 133.1, 130.1, 129.9, 123.1, 41.7 (brs, *C*N_2), 21.0,

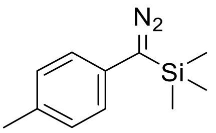

−0.8. IR (ATR, neat) 2958, 2922, 2032, 1509, 1283, 1252, 1174, 940, 835, 805, 753, 633, 612, 497 cm^{-1}. HRMS-EI (*m/z*): calcd. for $C_{11}H_{16}N_2Si^+$ [M^+]: 204.1077; found: 204.1076.

Ruthenium Carbene and Alkylidyne Complexes

{Ru[=C(*p*-tolyl)TMS]Cl(NCMe)$_2$}$_2$(*μ*-Cl)$_2$ (2). A three-necked flask equipped with a gas inlet, an oil bubbler and a stir bar was charged with [(*p*-cymene)$RuCl_2$]$_2$ (5.171 g, 8.4 mmol) and degassed MeCN (80 mL). The mixture was stirred until a homogeneous orange solution had formed. *p*-Tolyl(trimethylsilyl)-diazomethane (**1**) (3.630 g, 17.8 mmol) was then added dropwise to the solution. After stirring at room temperature for 30 min, a green crystalline solid started to precipitate from the mixture. After 5 h, the green solid material was collected by filtration, washed with MeCN (3 × 5 mL), toluene (10 mL) and pentane (3 × 10 mL), and dried under vacuum to give the title complex as a green crystalline solid (5.651 g, 78%).

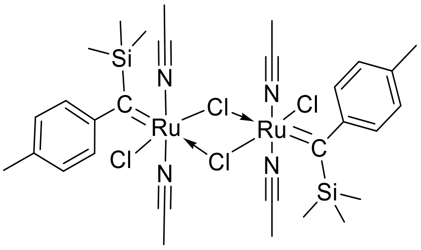

Since the compound is only sparingly soluble in acetonitrile, only the ^{1}H NMR spectrum could be recorded. In CD_3CN solution, the compound forms a pair of CD_3CN-ligated *cis-trans* isomers, which are tentatively assigned as {**Ru[=C(*p*-tolyl)TMS]Cl$_2$(NCMe)$_3$**} containing three (deuterated) acetonitrile ligands, see copies of spectra. ^{1}H NMR (400 MHz, CD_3CN) δ [7.25 (d, *J* = 7.8 Hz), 7.15 (d, *J* = 7.8 Hz), 2H], [6.79 (d, *J* = 8.1 Hz), 6.70 (d, *J* = 8.0 Hz), 2H], [2.36 (s), 2.31 (s), 3H], 1.96 (s, 6H, free C*H*$_3$CN), [0.20 (s), 0.16 (s), 9H]. IR (ATR, powder): 2968, 2917, 1496, 1415, 1245, 1021, 947, 909, 877, 834, 795, 763, 743, 685, 622, 512, 459 cm^{-1}. Anal. calcd. for $C_{30}H_{44}Cl_4N_4Ru_2Si_2$: C 41.86, H 5.15, N 6.51; found: C 41.72, H 5.11, N 6.48.

{Ru(=CH-*p*-tolyl)(^{iPr}PNP)Cl} (7). A Schlenk flask was charged with the Ru-P^N^P alkylidyne complex **4** (63 mg, 0.10 mmol)[12] and toluene (5 mL). HCl (0.5 M in Et_2O, 0.2 mL, 0.10 mmol) was added at room temperature to the mixture and stirring continued 10 min. The volatiles were evaporated under vacuum and the residue was washed with pentane (3 × 1 mL) and dried under vacuum to give the title complex as a greenish brown solid (52 mg, 78%).

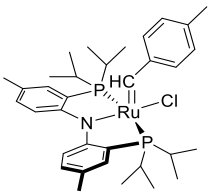

The combined pentane filtrates were stored at room temperature overnight to give single crystals suitable for X-ray diffraction analysis.

^{1}H NMR (600 MHz, C_6D_6) δ 17.98 (t, *J* = 8.8 Hz, 1H), 7.99 (br, 2H), 7.63 (dt, *J* = 8.5, 2.1 Hz, 2H), 6.92 – 6.88 (m, 2H), 6.85 (d, *J* = 8.0 Hz, 2H), 6.79 (dd, *J* = 8.5, 2.0 Hz, 2H), 2.51 – 2.43 (m, 2H), 2.17 (s, 6H), 2.10 – 2.03 (m, 2H), 1.70 (s, 3H), 1.68 (q, *J* = 7.6 Hz, 6H), 1.28 (q, *J* = 6.9 Hz, 6H), 0.86 – 0.77 (m, 12H). ^{13}C{^{1}H} NMR (151 MHz, C_6D_6) δ 299.4 (t, *J* = 10.9 Hz), 161.8 (t, *J* = 10.1 Hz), 150.7 (t, *J* = 3.0 Hz), 139.6, 132.3, 131.9, 130.2 (br), 129.8, 125.8, 121.4 (t, *J* = 16.8 ppm), 117.7 (br), 25.1 (t, *J* = 10.9 Hz), 22.0, 21.9 (t, *J* = 10.1 Hz), 20.6, 18.9, 17.64,

17.61 (t, J = 1.9 Hz), 16.9. $^{31}P\{^1H\}$ NMR (162 MHz, C_6D_6) δ 45.84. Anal. calcd. for $C_{34}H_{48}ClNP_2Ru$: C 61.02, H 7.23, N 2.09; found: C 60.09, H 7.16, N 2.02.

Complex 8. In Situ Method A: A Schlenk flask was charged with complex **2** (86 mg, 0.10 mmol), PCy_3 (123 mg, 0.44 mmol) and toluene (8 mL). The resulting green suspension was stirred at 50 °C for 30 min, leading to the formation of a clear dark turquoise solution. After cooling to room temperature, 2,6-di-*tert*-butylpyridium chloride (**18**) (55 mg, 0.24 mmol) was added, causing an immediate color change to purple. The mixture was filtered and the filtrate was concentrated to dryness under vacuum. The residue was washed with MeOH (3 × 2 mL) and dried under vacuum to give the title complex as a purple solid (142 mg, 85%). ^1H NMR (400 MHz, CD_2Cl_2) δ 19.81 (s, 1H), 8.33 (d, J = 8.0 Hz, 2H), 7.13 (d, J = 8.0 Hz, 2H), 2.68 – 2.54 (m, 6H), 2.09 (s, 3H), 1.86 – 1.59 (m, 30H), 1.50 – 1.35 (m, 12H), 1.31 – 1.12 (m, 18H). $^{13}C\{^1H\}$ NMR (151 MHz, CD_2Cl_2) δ 293.7 (t, J = 7.9 Hz), 151.4, 140.4, 131.5, 129.8, 32.4 (t, J = 9.1 Hz), 30.0, 28.2 (t, J = 5.2 Hz), 27.0, 22.4. $^{31}P\{^1H\}$ NMR (243 MHz, CD_2Cl_2) δ 35.7.
The spectroscopic data match the literature.[13]

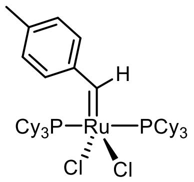

In Situ Method B: A Schlenk flask was charged with complex **2** (86 mg, 0.10 mmol), PCy_3 (123 mg, 0.44 mmol), 2,6-di-*tert*-butylpyridium chloride (**18**) (55 mg, 0.24 mmol) and toluene (8 mL). The resulting green suspension was stirred at 50 °C for 30 min, resulting in the formation of a brown solution. After cooling to room temperature, the mixture was filtered and the filtrate was concentrated to dryness under vacuum. The residue was washed with MeOH (3 × 2 mL) and dried under vacuum to give the title complex as a purple solid (117 mg, 70%).

[Ru(≡C-*p*-tolyl)Cl(PCy₃)₂]·toluene (9). A Schlenk flask was charged with {Ru[=C(*p*-tolyl)TMS]Cl(NCMe)₂}(*µ*-Cl)₂ (**2**) (172 mg, 0.20 mmol), PCy_3 (246 mg, 0.88 mmol) and toluene (15 mL). The resulting green suspension was stirred at 50 °C (bath temperature) for 30 min to form a clear dark turquoise solution. The solution was then stored in a freezer (–20 °C) for 2 d. The precipitated blue needle-shaped microcrystals were collected by filtration (*see below for the processing of the filtrate*), washed with cold toluene (2 × 3 mL) and dried under vacuum. The desired Ru carbyne complex was obtained as a pale blue microcrystalline solid, which turned out to contain solute toluene (306 mg, 85%). A toluene-free sample suitable for NMR analysis was obtained by dissolving the microcrystals in C_6D_6 and evaporating the volatiles under vacuum.

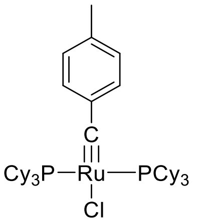

Single crystals suitable for X-ray diffraction analysis were obtained by layering a solution of the complex in toluene with MeCN.

^1H NMR (600 MHz, C_6D_6) δ 7.76 (d, J = 8.1 Hz, 2H), 6.63 (d, J = 7.9 Hz, 2H), 2.53 – 2.43 (m, 6H), 2.43 – 2.33 (m, 12H), 2.07 – 2.96 (m, 12H), 1.83 – 1.76 (m, 12H), 1.69 (s, 3H), 1.67 – 1.61 (m, 6H), 1.32 – 1.23 (m, 6H), 1.20 – 1.11 (m, 12H). $^{13}C\{^1H\}$ NMR (151 MHz, C_6D_6) δ 237.8 (t, J = 18.4 Hz), 140.4 (t, J = 3.4 Hz), 139.4, 129.1, 127.5, 36.1 (t, J = 8.8 Hz), 31.5, 28.2

(t, J = 5.1 Hz), 27.3, 21.9. $^{31}P\{^1H\}$ NMR (162 MHz, C_6D_6) δ 41.8. Anal. calcd. for $C_{44}H_{73}ClP_2Ru{\cdot}C_7H_8$: C 68.62, H 9.15; found: C 68.39, H 9.17.
The spectroscopic data match the literature.[13]

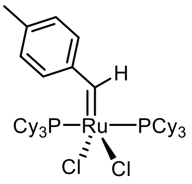

The filtrate was evaporated to dryness under vacuum to give a purple solid, which was washed with pentane (2 × 2 mL) and MeOH (2 × 1 mL) and dried under vacuum to give the carbene complex **8** as a purple solid (29 mg, 9%). For the spectral data, see above.

Complex 14a. A Schlenk flask was charged with {Ru[=C(*p*-tolyl)TMS]Cl(NCMe)$_2$}(μ-Cl)$_2$ (**2**) (86 mg, 0.10 mmol), PCy$_3$ (67 mg, 0.24 mmol), 1-isopropoxy-2-vinylbenzene (36 mg, 0.22 mmol) and toluene (5 mL). HCl (4 M in 1,4-dioxane, 50 μL, 0.20 mmol) was added at room temperature and the resulting mixture was stirred at 50 °C (bath temperature) for 1 h to form a homogeneous brown solution. The volatiles were evaporated under vacuum. The residue was purified by flash chromatography (SiO$_2$, hexane/CH$_2$Cl$_2$ = 5:1 to 1:1) to afford the title complex as dark red crystals (91 mg, 76%). ^1H NMR (400 MHz, CDCl$_3$) δ 17.42 (d, J = 4.4 Hz, 1H), 7.67 (dd, J = 7.6, 1.6 Hz, 1H), 7.61 (ddd, J = 8.8, 7.3, 1.6 Hz, 1H), 7.11 – 7.01 (m, 2H), 5.31 – 5.24 (m, 1H), 2.43 – 1.09 (m, 33H), 1.81 (d, J = 6.2 Hz, 6H). $^{13}C\{^1H\}$ NMR (101 MHz, CDCl$_3$) δ 280.1 (d, J = 13.8 Hz), 153.0, 144.1, 129.8, 123.0, 122.7, 113.5, 75.7, 35.8 (d, J = 24.6 Hz), 30.3 (d, J = 1.5 Hz), 27.9 (d, J = 10.6 Hz), 26.4, 22.3. $^{31}P\{^1H\}$ NMR (162 MHz, CDCl$_3$) δ 58.8.
The spectroscopic data match the literature.[14]

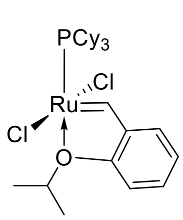

Complex 14b. A Schlenk flask was charged with {Ru[=C(*p*-tolyl)TMS]Cl(NCMe)$_2$}(μ-Cl)$_2$ (**2**) (86 mg, 0.10 mmol), PCy$_3$ (67 mg, 0.24 mmol), 1-isopropoxy-4-nitro-2-vinylbenzene (46 mg, 0.22 mmol) and toluene (5 mL). HCl (4 M in 1,4-dioxane, 55 μL, 0.22 mmol) was added at room temperature and the resulting mixture was stirred at 50 °C (bath temperature) for 1.5 h to form a brown solution. The volatiles were evaporated under vacuum and the residue was purified by flash chromatography (SiO$_2$; hexane/CH$_2$Cl$_2$ = 1:1 to CH$_2$Cl$_2$) to afford the title compound as a brown solid (92 mg, 71%). ^1H NMR (400 MHz, CDCl$_3$) δ 17.38 (d, J = 4.5 Hz, 1H), 8.60 (d, J = 2.7 Hz, 1H), 8.54 (dd, J = 9.1, 2.7 Hz, 1H), 7.18 (d, J = 9.2 Hz, 1H), 5.41 – 5.31 (m, 1H), 2.40 – 2.25 (m, 3H), 2.13 – 2.03 (m, 6H), 1.85 (d, J = 6.0 Hz, 6H), 1.923 – 1.68 (m, 15H), 1.39 – 1.18 (m, 9H). $^{13}C\{^1H\}$ NMR (101 MHz, CDCl$_3$) δ 273.5, 157.2, 143.4 (d, J = 16.6 Hz), 124.4, 117.8, 113.4, 78.4, 36.0 (d, J = 25.6 Hz), 30.3, 27.9 (d, J = 10.7 Hz), 26.4, 22.3. $^{31}P\{^1H\}$ NMR (162 MHz, CDCl$_3$) δ 60.1.
The spectroscopic data match the literature.[6]

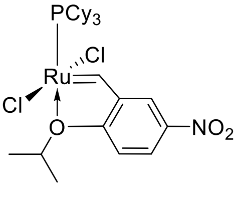

Ru(≡C-*p*-tolyl)Cl(IMes)$_2$ (25). A Schlenk flask was charged with {Ru[=C(*p*-tolyl)TMS]Cl(NCMe)$_2$}(μ-Cl)$_2$ (**2**) (172 mg, 0.20 mmol), IMes (268 mg, 0.88 mmol) and toluene (15 mL). The resulting suspension was stirred at 50 °C (bath temperature) for 2 h to form a clear dark turquoise solution. The solution was filtered to remove trace insoluble impurities and the filtrate was evaporated. The residue was washed with pentane (3 × 5 mL) and dried under vacuum. The title complex was obtained as a pale purple solid under natural light (246 mg). ***Note***: *Interestingly, the product appears gray when viewed under yellow laboratory lighting*. The pentane extracts were combined and stored in a freezer (–20 °C) for 2 d to give a second crop of product in form of brown crystals, which were collected by filtration and dried under vacuum (57 mg). Combined yield: 303 mg, 89%. ^{1}H NMR (600 MHz, C_6D_6) δ 6.88 (app d, J = 8.0 Hz, 2H), 6.86 (s, 8H), 6.52 (app d, J = 7.7 Hz, 2H), 6.48 (s, 4H), 2.38 (s, 12H), 2.09 (s, 24H), 1.85 (s, 3H). ^{13}C{^{1}H} NMR (151 MHz, C_6D_6) δ 229.3, 197.9, 141.6, 138.9, 136.6 (2 C overlapped), 135.9, 129.0, 127.7, 125.8, 121.9, 22.0, 21.4, 20.2. Anal. calcd. for $C_{50}H_{55}ClN_4Ru$: C 70.77, H 6.53, N 6.60; found: C 70.31, H 6.55, N 6.69.

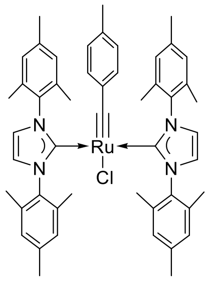

Ru(≡C-*p*-tolyl)Cl(IPr)$_2$ (26). A Schlenk flask was charged with {Ru[=C(*p*-tolyl)TMS]Cl(NCMe)$_2$}(μ-Cl)$_2$ (**2**) (172 mg, 0.20 mmol), IPr (342 mg, 0.88 mmol) and toluene (15 mL). The resulting green suspension was stirred at 50 °C (bath temperature) for 2 h to form a clear dark turquoise solution. The volatiles were evaporated under vacuum and the residue was extracted with pentane (3 × 5 mL). The combined pentane phases were stored in a freezer (–20 °C) for 3 d to give the title complex as dark green crystals, which were collected by filtration, washed with cold pentane and dried under vacuum (206 mg). The mother liquor was concentrated to about 3 mL and was stored in a freezer (–20 °C) for 3 d to give the second crop of product (58 mg). Combined yield: 264 mg, 65%.

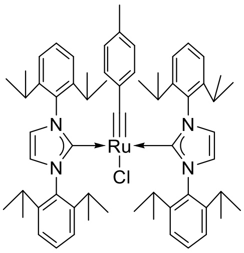

Single crystals suitable for X-ray diffraction analysis were obtained by storing a pentane solution at –20 °C.

^{1}H NMR (600 MHz, C_6D_6) δ 7.29 (t, J = 7.7 Hz, 4H), 7.18 – 7.16 (m, 2H), 7.10 (d, J = 7.7 Hz, 8H), 6.85 (s, 4H), 6.43 – 6.40 (m, 2H), 3.44 – 3.32 (m, 8H), 1.56 (s, 3H), 1.11 (d, J = 7.0 Hz, 24H), 0.79 (br s, 24H). ^{13}C{^{1}H} NMR (151 MHz, C_6D_6) δ 222.9, 197.4, 154.8, 146.7 (br), 139.3, 135.3, 129.8, 129.1, 124.8, 124.6, 123.9, 29.0, 26.4, 22.7 (br), 22.4. Anal. calcd. for $C_{62}H_{79}ClN_4Ru$: C 73.23, H 7.83, N 5.51; found: C 73.09, H 7.79, N 5.49.

Ru(≡C-*p*-tolyl)Cl(SIMes)$_2$ (27). A Schlenk flask was charged with {Ru[=C(p-tolyl)TMS]Cl(NCMe)2}(μ-Cl)2 (**2**) (172 mg, 0.20 mmol), SIMes (270 mg, 0.88 mmol) and toluene (10 mL). The resulting suspension was stirred at 50 °C (bath temperature) for 1 h to form a clear dark turquoise solution. The solution was filtered to remove trace insoluble impurities and the filtrate was evaporated. The residue was extracted with pentane (4 × 5 mL). The combined pentane extract was concentrated to a volume of ca. 10 mL and stored at room temperature over the weekend. The title complex precipitated as large green crystals whereas the byproduct, complex **35**, precipitated as tiny brown crystals. Large green crystals were carefully separated from the brown crystals by spatula, transferred into another Schlenk flask, washed with pentane (2 × 1 mL) and dried under vacuum (200 mg, 59%). ***Note**: About 25% of the Ru carbene complex **35** were formed as well.* ^{1}H NMR (400 MHz, C_6D_6) δ 6.92 (app d, *J* = 8.0 Hz, 2H), 6.86 (s, 8H), 6.57 (app d, *J* = 7.8 Hz, 2H), 3.38 (s, 8H), 2.40 (s, 12H), 2.18 (s, 24H), 1.89 (s, 3H). ^{13}C{^{1}H} NMR (101 MHz, C_6D_6) δ 229.3, 222.6, 141.6, 139.3, 137.2, 136.2, 135.6, 129.3, 127.7, 126.2, 51.3, 22.1, 21.4, 20.2. Anal. calcd. for $C_{50}H_{59}ClN_4Ru$: C 70.44, H 6.98, N 6.57; found: C 70.20, H 7.00, N 6.52.

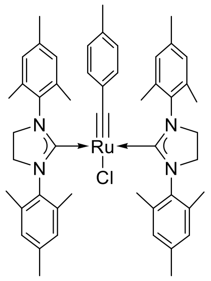

Complex 28. A Schlenk was charged with Ru(≡C-*p*-tolyl)Cl(IMes)$_2$ (**25**) (85 mg, 0.10 mmol) and 2,6-lutidinium hydrochloride (18 mg, 0.13 mmol) and toluene (5 mL). The mixture was stirred at room temperature for 30 min to form a brown solution, before it was filtered. The filtrate was concentrated to ca. 0.5 mL under vacuum and pentane (10 mL) was added. After stirring at room temperature for 30 min, a brown precipitate had formed, which was collected by filtration, washed with pentane (2 × 3 mL) and dried under vacuum. The desired product was obtained as a pale brown solid (55 mg, 62%). ^{1}H NMR (400 MHz, C_6D_6) δ 19.86 (s, 1H), 9.35 (dd, *J* = 8.1, 1.9 Hz, 1H), 6.73 – 6.63 (m, 1H), 6.65 (br, 8H), 6.61 – 6.52 (m, 1H), 6.30 (dd, *J* = 7.8, 2.0 Hz, 1H), 6.00 (s, 3H), 2.33 (s, 13H), 2.14 (br, 24H), 1.93 (s, 3H). ^{13}C{^{1}H} NMR (151 MHz, C_6D_6) δ 295.3, 192.2, 150.3, 137.6 (br), 137.3 (br), 137.2 (br), 135.6, 132.3, 130.0, 129.3 – 129.1 (m), 128.2 (overlapped with *C_6D_6*), 126.7, 124.5 (br), 22.3, 21.4 (br), 19.3 (br). HRMS (ESI$^+$) calcd. for $C_{50}H_{56}Cl_2N_4Ru^+$ $[M]^+$: 884.29200; found: 884.29249.

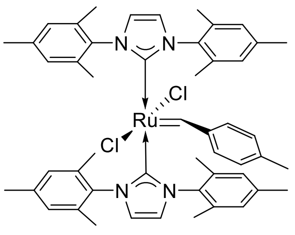

[Ru(=CH-*p*-tolyl)Cl(IPr)]$_2$(*μ*-Cl)$_2$ ([29]$_2$). A Schlenk tube was charged with Ru(≡C-*p*-tolyl)Cl(IPr)$_2$ (**26**) (81 mg, 0.080 mmol) and toluene (4 mL). HCl (4 M in 1,4-dioxane, 60 μL, 0.240 mmol) was added at room temperature, causing an immediate color change to orange. The color of the solution then gradually turned to green, while a white precipitate was formed over the course of 20 min. After stirring at room temperature for 40 min, the mixture was filtered and the green filtrate evaporated under vacuum to give a dark green residue, which was washed with pentane (3 × 2 mL) and dried under vacuum to give the title complex as a light green solid (48 mg, 90%).

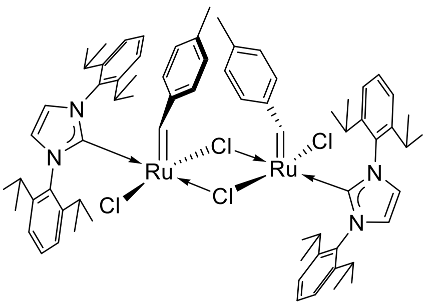

***Note**: The complex is rather unstable in solution even when kept cold. In the solid state, it could be stored at room temperature for a month without significant decomposition.*

Single crystals suitable for X-ray analysis were obtained by storing a saturated solution of the complex in CH_2Cl_2/*n*-hexane at –20 °C.

The DOSY spectra of this complex were compared with the DOSY spectra of the monomeric pyridine adduct **33a** generated in situ upon addition of excess pyridine; this comparison suggests that complex [**29**]$_2$ in solution is indeed a likely chloride-bridged dimer, see copies of spectra.

***Note**: The integrals of the NMR data refer to the monomeric subunit.* ^{1}H NMR (400 MHz, CD_2Cl_2, 298 K) δ 17.38 (s, 1H), 7.65 – 6.87 (br m, 8H), 6.82 (d, *J* = 7.8 Hz, 2H), 6.70 (d, *J* = 7.9 Hz, 2H), 2.76 (br s, 2H), 2.03 (s, 3H), 1.27 (d, *J* = 6.9 Hz, 6 H), 1.03 (br d, *J* = 6.0 Hz, 6H), 1.56 – 0.59 (br m, 14H). ^{1}H NMR (600 MHz, [D$_8$]-toluene, 298 K) 17.66 (s, 1H), 7.76 - 7.21 (br m, 5H), 6.79 (d, *J* = 8.1 Hz, 2H), 6.60 (br, 2H), 6.34 (br, 1H), 3.52 (br, 1H), 3.05 (sept, *J* = 6.6 Hz, 2H), 2.34 (br, 1H), 1.87 (s, 3H), 1.66 – 1.28 (br m, 9H), 1.14 – 0.90 (br m, 9H), 0.61 (br, 3H) 0.42 (br, 3H). The signals for the two *meta*-protons of the tolyl group are absent due to line broadening. ^{1}H NMR (600 MHz, [D$_8$]-toluene, 233 K) δ 17.70 (s, 1H), 7.61 (t, *J* = 7.8 Hz, 1H), 7.51 (dd, *J* = 7.8, 1.4 Hz, 1H), 7.38 (dd, *J* = 7.8, 1.4 Hz, 1H), 6.99 (t, *J* = 7.5 Hz, 1H), 6.96 (dd, *J* = 7.6, 1.6 Hz, 1H), 6.86 (d, *J* = 7.4 Hz, 2H), 6.56 (dd, *J* = 7.4, 1.6 Hz, 1H), 6.54 (d, *J* = 1.8 Hz, 1H), 6.21 (d, *J* = 1.8 Hz, 1H), 3.62 (sept, *J* = 6.7 Hz, 1H), 3.14 (sept, *J* = 6.7 Hz, 1H), 2.98 (sept, *J* = 6.7 Hz, 1H), 2.36 (sept, *J* = 6.8 Hz, 1H), 1.86 (s, 3H), 1.58 (d, *J* = 6.5 Hz, 3H), 1.54 (d, *J* = 6.6 Hz, 3H), 1.33 (d, *J* = 6.6 Hz, 3H), 1.19 (d, *J* = 6.7 Hz, 3H), 1.13 (d, *J* = 6.9 Hz, 3H), 1.02 (d, *J* = 6.8 Hz, 3H), 0.61 (d, *J* = 7.0 Hz, 3H), 0.47 (d, *J* = 6.5 Hz, 3H). The signals for the two *meta*-protons of the tolyl group are absent due to line broadening. ^{13}C{^{1}H} NMR (151 MHz, [D$_8$]-toluene, 233 K) δ 306.7, 184.4, 150.1, 148.5, 148.1, 146.4 (2C), 139.6, 136.4, 134.9, 131.2, 129.6, 128.3, 125.9, 125.5, 124.6 (2C), 124.5, 123.4, 29.4, 29.2, 28.6, 27.5, 27.3, 27.2, 25.9, 25.4, 23.8, 23.5, 23.1, 22.5, 21.9.

Complex 31. Method A: A Schlenk flask was charged with Ru(≡C-*p*-tolyl)Cl(IPr)$_2$ (**26**) (58 mg, 0.057 mmol), PCy_3 (19 mg, 0.068 mmol) and toluene (5 mL). HCl (0.5 M in Et_2O, 0.23 mL, 0.115 mmol) was added at room temperature, resulting in a brown solution. The mixture was stirred at room temperature for 1 h to form a reddish solution and a white precipitate. The mixture was filtered and the filtrate concentrated under vacuum. The residue was washed with MeOH (2 × 1 mL) and dried under vacuum to give the title complex as a pink solid (44 mg, 82%).

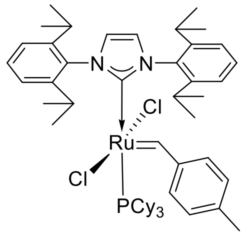

Method B: A Schlenk flask was charged with Ru carbene dimer **2** (90 mg, 0.10 mmol), IPr (180 mg, 0.46 mmol) and toluene (5 mL). The mixture was stirred at 50 °C (bath temperature) for 1 h to form a dark green solution. After cooling to room temperature, PCy_3 (146 mg, 0.52 mmol), and HCl (4 M in 1,4-dioxane, 130 µL, 0.52 mmol) were added to give a reddish-brown solution, from which a white solid started to precipitate. The resulting mixture was then stirred at 50 °C

(bath temperature) for 1 h, cooled to ambient temperature and filtered. The filtrate was concentrated under vacuum, and the residue was washed with MeOH (3 × 1 mL) and dried under vacuum to give the title complex as a pink solid (121 mg, 61%). ^{1}H NMR (400 MHz, CD_2Cl_2) δ 19.40 (s, 1H), 7.85 (br s, 2H), 7.49 (t, *J* = 7.7 Hz, 1H), 7.39 (d, *J* = 7.8 Hz, 2H), 7.09 (s, 1H), 7.04 (s, 1H), 6.87 – 6.82 (m, 3H), 6.81 – 6.75 (m, 2H), 3.55 (sept, *J* = 6.2 Hz, 2H), 3.13 – 3.04 (m, 2H), 2.18 – 2.04 (m, 3H), 2.02 (s, 3H), .51 – 1.22 (m, 27H), 1.10 – 0.89 (m, 27H). $^{13}C\{^{1}H\}$ NMR (101 MHz, CD_2Cl_2) δ 295.9 (d, *J* = 9.1 Hz), 190.8 (d, *J* = 84.1 Hz), 150.0, 148.7, 146.2, 138.7, 137.6, 136.1, 131.9, 131.4, 130.0, 128.6, 126.3, 126.1, 124.3, 123.8, 32.2 (d, *J* = 16.8 Hz), 29.6, 29.2, 28.2, 28.1 (d, *J* = 9.9 Hz), 27.0, 26.6, 26.4, 22.9, 22.8, 22.3. $^{31}P\{^{1}H\}$ NMR (162 MHz, CD_2Cl_2) δ 28.9. HRMS (ESI^{+}) calcd. for $C_{53}H_{77}Cl_2N_2PRu^{+}$ $[M]^{+}$: 944.42394; found: 944.42365.

Complex 32. A Schlenk flask was charged with [Ru(≡C-*p*-tolyl)Cl(IPr)$_2$] (**26**) (51 mg, 0.05 mmol), DMSO (5 mg, 0.06 mmol) and toluene (5 mL). HCl (0.5 M in Et_2O, 0.3 mL, 0.15 mmol) was added and the resulting mixture was stirred at room temperature for 1 h to afford a greenish brown solution and a white precipitate. The mixture was filtered and the filtrate was concentrated under vacuum. The residue was washed with pentane (3 × 1 mL) and dried under vacuum to give the title complex as a dusty lilac solid (30 mg, 77%).

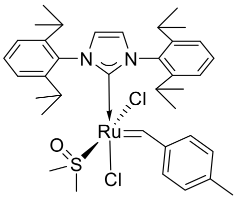

^{1}H NMR (400 MHz, CD_2Cl_2) δ 15.94 (s, 1H), 7.66 (br d, *J* = 7.8 Hz, 2H), 7.61 (t, *J* = 7.7 Hz, 1H), 7.53 (dd, *J* = 7.8, 1.6 Hz, 1H), 7.47 (dd, *J* = 7.7, 1.6 Hz, 1H), 7.30 (s, 1H), 7.29 (d, *J* = 2.7 Hz, 1H), 7.20 (d, *J* = 1.9 Hz, 1H), 7.01 (d, *J* = 8.0 Hz, 2H), 6.95 (d, *J* = 2.0 Hz, 1H), 6.79 (dd, *J* = 5.9, 3.4 Hz, 1H), 3.96 (sept, *J* = 6.7 Hz, 1H), 3.40 (sept, *J* = 6.7 Hz, 1H), 3.10 (sept, *J* = 6.7 Hz, 1H), 2.65 (sept, *J* = 6.7 Hz, 1H), 2.35 (s, 3H), 2.14 (s, 3H), 1.97 (s, 3H), 1.57 – 1.44 (m, 9H), 1.20 (d, *J* = 6.8 Hz, 3H), 1.08 (d, *J* = 6.8 Hz, 3H), 0.96 (d, *J* = 6.9 Hz, 3H), 0.91 (d, *J* = 6.8 Hz, 3H), 0.65 (d, *J* = 6.6 Hz, 3H). $^{13}C\{^{1}H\}$ NMR (101 MHz, CD_2Cl_2) δ 304.3, 179.4, 149.6, 149.0, 148.9, 148.1, 147.1, 144.1, 136.0 (2C), 131.7 (2C), 131.6, 130.8, 128.6 (2C), 126.4, 125.9, 125.8, 125.7, 125.4, 123.4, 48.3, 44.1, 29.4, 29.1, 28.6, 28.1, 28.0, 27.0, 26.5, 26.2, 23.6, 23.1, 23.0, 22.9, 22.4. HRMS (ESI^{+}) calcd. for $C_{37}H_{50}ClN_2ORuS^{+}$ $[M\text{-}Cl]^{+}$: 707.23703; found: 707.23647.

Complex 33a. A Schlenk flask was charged with {Ru[=C(*p*-tolyl)TMS]Cl(NCMe)$_2$}(μ-Cl)$_2$ (**2**) (23 mg, 0.027 mmol), IPr (46 mg, 0.118 mmol) and toluene (3 mL). The resulting green suspension was stirred at 50 °C (bath temperature) for 1 h to afford a clear dark turquoise solution. After cooling to room temperature, pyridinium hydrochloride (19 mg, 0.160 mmol) was added to the solution. The resulting mixture was stirred at 50 °C (bath temperature) for another 1 h to afford a light green solution and a white precipitate. After cooling to ambient temperature, the mixture was filtered and the filtrate was concentrated under vacuum. The residue was washed with pentane (3 × 3 mL) and dried under vacuum to give the title complex as a light green solid (39 mg, 88%).

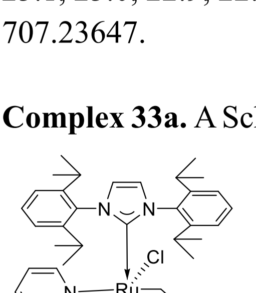

^{1}H NMR (600 MHz, C_6D_6) δ 19.55 (s, 1H), 8.59 (br s, 2H), 8.40 (app d, *J* = 5.0 Hz, 2H), 7.81 (app d, *J* = 8.3 Hz, 2H), 7.37 – 7.32 (m, 2H), 7.29 – 7.20 (m, 4H), 6.94 (br t, *J* = 7.6 Hz, 1H), 6.78 (br s, 2H), 6.71 (app d, *J* = 7.6 Hz, 2H), 6.63 (br s, 2H), 6.42 (t, *J* = 7.6 Hz, 1H), 6.15 – 6.08

(m, 2H), 3.63 (br s, 4H), 1.53 (s, 3H), 1.48 (br s, 12H), 1.12 (d, J = 6.9 Hz, 12H). $^{13}C\{^1H\}$ NMR (151 MHz, C_6D_6) δ 311.3, 188.3, 153.5, 151.0, 150.3 (br), 148.2 (br), 140.1, 137.2 (br), 135.8, 135.2, 130.4 (br), 130.3, 129.2, 125.9, 124.4 (br), 123.4 (br), 123.1, 29.0 (br), 26.7 (br), 23.3, 22.2. Anal. calcd. for $C_{45}H_{54}Cl_2N_4Ru$: C 65.68, H 6.61, N 6.81; found: C 64.82, H 6.73, N 6.55.

Complex 33b. A Schlenk flask was charged with {Ru[=C(p-tolyl)TMS]Cl(NCMe)$_2$}(μ-Cl)$_2$ (**2**) (86 mg, 0.10 mmol), IPr (171 mg, 0.44 mmol) and toluene (5 mL). The resulting green suspension was stirred at 50 ºC (bath temperature) for 1 h to afford a clear dark turquoise solution. After cooling to ambient temperature, HCl (4 M in 1,4-dioxane, 125 µL, 0.50 mmol) was added and the mixture stirred at room temperature for 5 min before 3-bromopyridine (79 mg, 0.50 mmol) was introduced. The resulting mixture was stirred at 50 ºC (bath temperature) for another 1 h to afford a green solution and a white precipitate. After cooling to ambient temperature, the mixture was filtered and the filtrate was concentrated under vacuum. The residue was washed with pentane (3 × 3 mL) and dried under vacuum to give the title complex as a yellow solid (162 mg, 83%). ^1H NMR (400 MHz, CD_2Cl_2) δ 18.98 (s, 1H), 8.70 (br s, 1H), 8.54 (br s, 1H), 8.16 (br s, 1H), 8.00 (br d, J = 5.5 Hz, 1H), 7.81 (br d, J = 8.1 Hz, 1H), 7.62 (br d, J = 8.2 Hz, 1H), 7.49 (t, J = 7.8 Hz, 2H), 7.29 (d, J = 3.6 Hz, 4H), 7.27 (d, J = 4.1 Hz, 2H), 7.17 (s, 2H), 7.20 – 7.14 (m, 1H), 6.86 (br t, J = 7.0 Hz, 1H), 6.81 (d, J = 8.0 Hz, 2H), 3.18 (br s, 4H), 1.92 (s, 3H), 1.18 (br s, 12H), 1.09 (d, J = 6.9 Hz, 12H). $^{13}C\{^1H\}$ NMR (101 MHz, CD_2Cl_2) δ 312.4, 185.2, 154.3 (br), 151.9 (br), 151.4 (br), 150.4, 148.5 (br), 148.0 (br), 141.1, 139.4 (br), 138.9 (br), 136.5, 130.5, 129.8, 129.1, 126.3, 125.1 (br), 124.7 (br), 124.3, 28.9, 26.6, 22.9, 22.4 (***Note****: the two Br-C signals of 3-bromopyridine ligands were not observed due to line broadening*). Anal. calcd. for $C_{45}H_{52}Br_2Cl_2N_4Ru$: C 55.11, H 5.34, N 5.71; found: C 55.06, H 5.32, N 5.67.

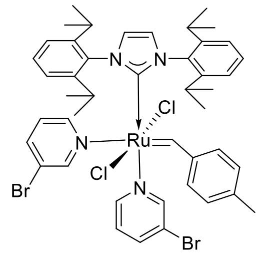

Complex 34a. Method A: A Schlenk flask was charged with Ru(≡C-p-tolyl)Cl(IPr)$_2$ (**26**) (51 mg, 0.05 mmol), 1-isopropoxy-2-vinylbenzene (10 mg, 0.06 mmol) and toluene (5 mL). HCl (0.5 M in Et_2O, 0.30 mL, 0.15 mmol) was added, resulting in the formation of a brown solution, which was stirred at 50 ºC (bath temperature) for 1 h during which a white precipitate was formed. After cooling to ambient temperature, the mixture was filtered and the filtrate concentrated under vacuum. The residue was purified by flash chromatography (SiO_2; hexane/Et_2O = 30:1, then CH_2Cl_2) to afford the title complex as a brown solid (30 mg, 85%).

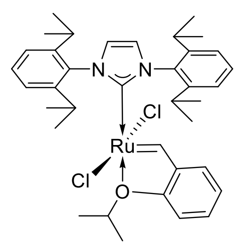

Method B (Gram-Scale Experiment): A Schlenk flask was charged with Ru carbene dimer (**2**) (861 mg, 1.0 mmol), IPr (1.710 g, 4.4 mmol) and toluene (40 mL). The mixture was stirred at 50 ºC (bath temperature) for 1 h to form a dark green solution. After reaching ambient temperature, 1-isopropoxy-2-vinylbenzene (357 mg, 2.2 mmol) and HCl (4 M in 1,4-dioxane, 1.25 mL, 5.0 mmol) were added, resulting in the formation of a brown solution, from which a gray solid started to precipitate. The resulting mixture was stirred at 50 ºC (bath temperature) for 1 h before it was cooled to room temperature and filtered. The brown filtrate was concentrated under vacuum to form a brown crystalline solid, which was washed with pentane

(4 × 5 mL) and dried under vacuum to give the title complex (1.043 g, 76%). ^{1}H NMR (400 MHz, CD_2Cl_2) δ 16.50 (s, 1H), 7.63 (t, *J* = 7.8 Hz, 2H), 7.56 – 7.49 (m, 1H), 7.43 (d, *J* = 7.8 Hz, 4H), 7.19 (s, 2H), 6.97 (dd, *J* = 7.5, 1.7 Hz, 1H), 6.93 – 6.83 (m, 2H), 4.92 (sept, *J* = 6.2 Hz, 1H), 3.08 (sept, *J* = 6.8 Hz, 4H), 1.37 (d, *J* = 6.2 Hz, 6H), 1.19 (d, *J* = 6.7 Hz, 12H), 1.14 (d, *J* = 6.9 Hz, 12H). ^{13}C{^{1}H} NMR (101 MHz, CD_2Cl_2) δ 286.9, 177.6, 152.7, 148.6, 145.0, 136.6, 130.8, 129.1, 126.6, 124.2, 122.8, 121.9, 113.4, 75.6, 29.2, 26.5, 22.8, 21.8.
The spectroscopic data match the literature.[15]

Recovery of the Imidazolium Salt 30: In air, the gray precipitate that has been filtered off was dissolved in water (100 mL). Traces of ruthenium black were removed by filtration. The aqueous phase was washed with *tert*-butyl methyl ether (3 x) and then extracted with CH_2Cl_2 (3 x). The combined CH_2Cl_2 extracts were dried over $MgSO_4$ and evaporated to give pure imidazolium chloride **30** as an off-white solid (990 mg, 53% (based on IPr)).

Complex 34b. A Schlenk flask was charged with {Ru[=C(*p*-tolyl)TMS]Cl(NCMe)$_2$}(μ-Cl)$_2$ (**2**) (86 mg, 0.10 mmol), IPr (171 mg, 0.44 mmol) and toluene (5 mL). The mixture was stirred at 50 °C (bath temperature) for 1 h to form a dark green solution. After reaching ambient temperature, 1-isopropoxy-4-nitro-2-vinylbenzene (46 mg, 0.22 mmol) and HCl (4 M in 1,4-dioxane, 125 µL, 0.5 mmol) were added, resulting in a brown solution from which a white solid started to precipitate. The mixture was stirred at 50 °C (bath temperature) for 1.5 h. After cooling to room temperature, the mixture was filtered and the filtrate was evaporated under vacuum. The residue was purified by flash chromatography (SiO_2; hexane/ CH_2Cl_2 = 2:1 to pure CH_2Cl_2) to afford the title complex as a brown solid (125 mg, 83%). ^{1}H NMR (400 MHz, CD_2Cl_2) δ 16.43 (s, 1H), 8.42 (dd, *J* = 9.1, 2.7 Hz, 1H), 7.79 (d, *J* = 2.7 Hz, 1H), 7.68 (t, *J* = 7.8 Hz, 2H), 7.46 (d, *J* = 7.8 Hz, 4H), 7.22 (s, 2H), 6.97 (d, *J* = 9.1 Hz, 1H), 5.03 (sept, *J* = 6.2 Hz, 1H), 3.04 (sept, *J* = 6.8 Hz, 4H), 1.41 (d, *J* = 6.2 Hz, 6H), 1.19 (d, *J* = 6.7 Hz, 12H), 1.14 (d, *J* = 6.9 Hz, 12H). ^{13}C{^{1}H} NMR (101 MHz, CD_2Cl_2) δ 280.6, 174.0, 157.1, 148.6, 144.6, 143.7, 136.2, 131.1, 126.9, 124.4, 123.9, 116.3, 113.3, 78.4, 29.2, 26.5, 22.8, 21.8.
The spectroscopic data match the literature.[15]

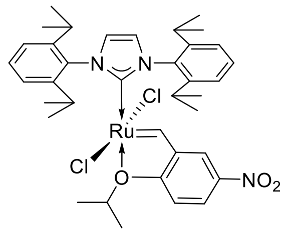

Complex 35. A Schlenk was charged with {Ru[=C(*p*-tolyl)TMS]Cl(NCMe)$_2$}(μ-Cl)$_2$ (**2**) (86 mg, 0.10 mmol), SIMes (135 mg, 0.44 mmol) and toluene (5 mL). The resulting green suspension was stirred at 50 °C (bath temperature) for 1 h to afford a clear dark turquoise solution. After cooling to room temperature, HCl (4 M in 1,4-dioxane, 75 µL, 0.30 mmol) was introduced and the resulting mixture stirred at 50 °C for 1 h to afford a brown solution, from which a white solid started to precipitate. After cooling to ambient temperature, the mixture was filtered and the filtrate was concentrated under vacuum. The residue was washed with pentane (3 × 3 mL) and dried under vacuum to give the title complex as a pale brown solid (163 mg, 92%). ^{1}H NMR (400 MHz, C_6D_6) δ 19.29 (s, 1H), 9.33 (dd, *J* = 8.3, 2.0 Hz, 1H), 6.82 (br s, 4H), 6.74 (dd, *J* = 8.1, 1.9 Hz, 1H), 6.56 – 6.48 (m, 2H), 6.08 (dd, *J* = 7.8, 2.0 Hz, 1H), 5.65 (s, 2H), 3.08 (s, 2H), 2.92 (s, 3H), 2.55 (s, 4H), 2.52 –

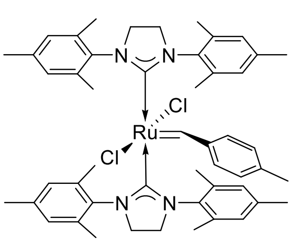

2.46 (m, 16H), 2.37 (s, 8H), 2.06 (s, 6H), 1.95 (s, 3H), 1.88 (s, 6H). $^{13}C\{^1H\}$ NMR (151 MHz, C_6D_6) δ 295.0, 222.2, 149.6, 138.4 (br), 138.2 (br), 137.5 – 137.0 (m), 136.4 (br), 135.5, 132.2, 130.6 – 130.2 (m), 130.0, 129.4 (br), 128.9 (br), 127.6, 126.5, 53.0 (br), 51.7 (br), 22.2, 21.5 (br), 21.1 (br), 20.0 (br), 19.8 (br), 19.3 (br), 19.1 (br). HRMS (ESI$^+$) calcd. for $C_{50}H_{60}Cl_2N_4Ru^+$ $[M]^+$: 888.32330; found: 888.32394.

Complex 15. A Schlenk flask was charged with Ru(≡C-*p*-tolyl)Cl(SIMes)$_2$ (**27**) (85 mg, 0.10 mmol), 1-isopropoxy-2-vinylbenzene (24 mg, 0.15 mmol) and toluene (5 mL). HCl (0.5 M in Et_2O, 0.60 mL, 0.30 mmol) was added, resulting in the formation of a brown solution. The resulting mixture was stirred at 100 °C (bath temperature) for 16 h. After reaching ambient temperature, the mixture was filtered and the filtrate was concentrated under vacuum. The residue was purified by flash chromatography (SiO_2; hexane/Et_2O = 30:1, then CH_2Cl_2) to afford the title complex as a pale green solid (23 mg, 37%). ^1H NMR (400 MHz, CD_2Cl_2) δ 16.51 (s, 1H), 7.55 (ddd, *J* = 8.8, 7.2, 1.8 Hz, 1H), 7.07 (s, 4H), 6.96 (dd, *J* = 7.5, 1.8 Hz, 1H), 6.90 (t, *J* = 7.3 Hz, 1H), 6.84 (d, *J* = 8.3 Hz, 1H), 4.88 (sept, *J* = 6.1 Hz, 1H), 4.16 (s, 4H), 2.44 (s, 12H), 2.41 (s, 6H), 1.23 (d, *J* = 6.0 Hz, 6H). $^{13}C\{^1H\}$ NMR (101 MHz, CD_2Cl_2) δ 296.0, 211.2, 152.4, 145.6, 139.3, 129.8, 129.7, 122.7, 122.6, 113.3, 75.5, 51.9, 21.3, 21.2, 19.6.

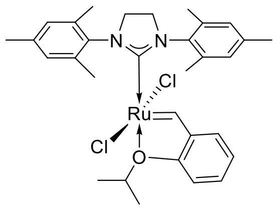

The spectroscopic data match the literature.[15]

Complex 36. A Schlenk flask was charged with {Ru[=C(*p*-tolyl)TMS]Cl(NCMe)$_2$}(μ-Cl)$_2$ (**2**) (86 mg, 0.10 mmol), SIDep (147 mg, 0.44 mmol) and toluene (5 mL). The resulting green suspension was stirred at 50 °C (bath temperature) for 1 h, resulting in the formation of a clear dark turquoise solution. After cooling to ambient temperature, HCl (4 M in 1,4-dioxane, 125 µL, 0.50 mmol) and 1-isopropoxy-2-vinylbenzene (36 mg, 0.22 mmol) were added, giving rise to a brown solution. This mixture was stirred at 80 °C (bath temperature) for 3 h. After reaching ambient temperature, the mixture was filtered and the filtrate was concentrated under vacuum. The residue was purified by flash chromatography (SiO_2; hexane/ CH_2Cl_2 = 2:1 to pure CH_2Cl_2) to afford the title complex as a dark green crystalline solid (89 mg, 68%). ^1H NMR (400 MHz, $CDCl_3$) δ 16.40 (s, 1H), 7.54 – 7.40 (m, 3H), 7.32 (d, *J* = 7.7 Hz, 4H), 6.89 (dd, *J* = 7.6, 1.7 Hz, 1H), 6.82 (td, *J* = 7.5, 0.8 Hz, 1H), 6.76 (d, *J* = 8.3 Hz, 1H), 4.86 (sept, *J* = 6.1 Hz, 1H), 4.21 (s, 4H), 3.03 (br, 4H), 2.92 – 2.78 (m, 4H), 1.27 (d, *J* = 7.4 Hz, 6H), 1.25 (d, *J* = 6.4 Hz, 12H). $^{13}C\{^1H\}$ NMR (151 MHz, CD_2Cl_2) δ 294.3, 212.1, 152.4, 145.2 (br), 145.1, 138.0 (br), 129.8, 129.7, 126.7, 122.6, 122.5, 113.3, 75.5, 53.4 (br), 25.2 (br), 21.4, 15.1 (br). HRMS (ESI$^+$) calcd. for $C_{33}H_{42}Cl_2N_2ORu^+$ $[M]^+$: 654.17121; found: 654.17129.

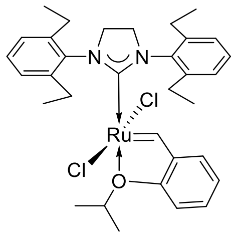

Ru[=C(*p*-tolyl)TMS]Cl$_2$(py)$_3$ (39). To a green suspension of the Ru carbene dimer **2** (2.15 g, 5.0 mmol) in MeCN (40 mL) was added pyridine (4.3 mL, 50.0 mmol). The mixture was stirred at room temperature for 1 h until a clear dark green solution had formed. All volatile materials were evaporated under vacuum. The residue was washed with pentane (2 × 20 mL) and dried under vacuum to give the title compound as a green solid (2.67 g, 91%). In solution, the product consists of a mixture of *trans* (major) and *cis* (minor) isomers in ca. 95:5 ratio (NMR).

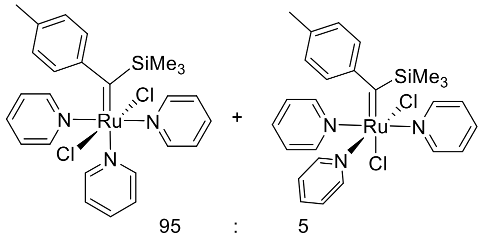

Reverse vapor diffusion of a solution of the *cis/trans* mixture in CH_2Cl_2 into *n*-hexane furnished single crystals suitable for X-ray diffraction analysis of the minor *cis*-isomer as well as the chloride-bridged dimer.

Characterization data of ***trans*-39**: ^{1}H NMR (600 MHz, C_6D_6) δ 9.04 (d, *J* = 5.1 Hz, 4H), 8.95 (dt, *J* = 4.8, 1.7 Hz, 2H), 7.19 (d, *J* = 7.8 Hz, 2H), 6.97 (dt, *J* = 7.8, 0.7 Hz, 2H), 6.88 (tt, *J* = 7.6, 1.7 Hz, 1H), 6.63 – 6.56 (m, 4H), 6.28 – 6.20 (m, 4H), 2.11 (s, 3H), 0.28 (s, 9H). ^{13}C{^{1}H} NMR (151 MHz, C_6D_6) δ 403.4, 173.9, 157.5, 151.5, 136.1, 135.6, 132.9, 127.1, 123.6, 122.7, 113.4, 21.2, −1.3. ^{29}Si{^{1}H} NMR (119 MHz, C_6D_6) δ 7.5. Anal. calcd. for $C_{26}H_{31}Cl_2N_3RuSi$: C 55.33, H 5.34, N, 7.18; found: C 53.26, H 5.31, N 7.16.

Ru[=C(H)Ph]Cl$_2$(py)$_3$ (41). A Schlenk tube was charged with complex **39** (234 mg, 0.4 mmol), styrene (167 mg, 1.6 mmol) and CH_2Cl_2 (10 mL). The mixture was stirred at 40 °C (bath temperature) for 3 h, resulting in the formation of a green suspension. After cooling to room temperature, pyridine (0.1 mL) was added and stirring continued at ambient temperature for 20 min until a clear green solution had formed. All volatile materials were then removed under vacuum and the green residue was washed with pentane (2 × 10 mL) and diethyl ether (3 × 3 mL) to give the title complex as a green solid (173 mg, 87%), which consists in solution of a mixture of two geometric isomers (dr ≈ 3:2, NMR).

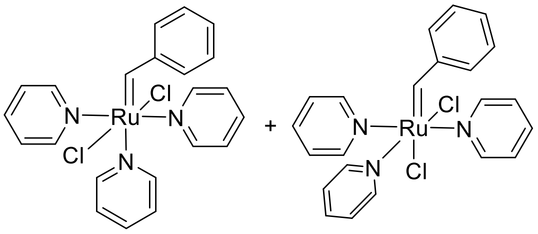

Single crystals suitable for X-ray diffraction analysis grown by the vapor diffusion technique (*n*-hexane) contained only the *trans*-isomer.

Characterization data of *trans*-**41:** ^{1}H NMR (600 MHz, CD_2Cl_2, 273 K) δ 21.43 (br s, 1H), 8.57 (br s, 2H), 8.28 (dt, *J* = 5.1, 1.6 Hz, 4H), 7.96 (dd, *J* = 8.3, 1.5 Hz, 2H), 7.86 (br s, 1H), 7.74 – 7.70 (m, 1H), 7.56 (tt, *J* = 7.6, 1.6 Hz, 2H), 7.40 (br s, 2H), 7.23 – 7.19 (m, 2H), 7.05 – 6.98 (m, 4H). ^{13}C{^{1}H} NMR (151 MHz, CD_2Cl_2, 273 K) δ 328.3, 157.4, 155.6, 151.5 (br), 137.1 (br), 136.1, 130.4, 130.3, 129.3, 124.4 (br), 123.7.

Characterization data of *cis*-**41**: ^{1}H NMR (600 MHz, CD_2Cl_2, 273 K) δ 20.48 (s, 1H), 8.35 – 8.32 (m, 2H), 8.31 (dt, *J* = 5.3, 1.5 Hz, 4H), 8.03 – 7.99 (m, 2H), 7.73 – 7.70 (m, 1H), 7.68 (tt,

J = 7.6, 1.5 Hz, 1H), 7.63 (tt, J = 7.6, 1.7 Hz, 2H), 7.24 – 7.18 (m, 2H), 7.18 – 7.15 (m, 2H), 7.11 – 7.07 (m, 4H). $^{13}C\{^1H\}$ NMR (151 MHz, CD_2Cl_2, 273 K) δ 325.0, 157.6, 154.7, 154.6, 137.2, 136.6, 131.5, 131.4, 129.2, 124.6, 124.1.

Anal. calcd. for $C_{22}H_{21}Cl_2N_3Ru$: C 52.91, H 4.24, N 8.41; found: C 52.87, H 4.26, N 8.37

"Instant Procedure" for Catalytic Olefin Metathesis

3-Cyclopentene-1,1-dicarboxylic acid, diethyl ester (20) A Schlenk flask was charged with diethyl 2,2-diallylmalonate (240 mg 1.0 mmol), complex **2** (4.3 mg, 0.005 mmol), PCy_3 (2.8 mg, 0.01 mmol), 2,6-di-*tert*-butylpyridium hydrochloride (**18**) (2.5 mg, 0.01 mmol) and toluene (10 mL). An oil bubbler was attached to the Schlenk flask and the mixture was stirred at 50 °C (bath temperature) for 1 h under a gentle flow of Ar. The volatiles were evaporated under vacuum and the residue was purified by flash chromatography (SiO_2, hexanes/EtOAc = 10:1) to give the title compound as a colorless oil (193 mg, 91%). ^1H NMR (400 MHz, $CDCl_3$) δ 5.60 (d, J = 0.8 Hz, 2H), 4.19 (q, J = 7.1 Hz, 4H), 3.00 (s, 4H), 1.24 (t, J = 7.1 Hz, 6H). $^{13}C\{^1H\}$ NMR (101 MHz, $CDCl_3$) δ 172.4, 127.9, 61.6, 59.0, 41.0, 14.2.

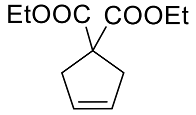

The spectroscopic data match the literature.[16]

N-Tosyl-2,5-dihydro-1*H*-pyrrole (21) A Schlenk tube was charged with N,N-bis(allyl)-toluene-*p*-sulfonamide (251 mg, 1.0 mmol), complex **2** (4.3 mg, 0.005 mmol), PCy_3 (2.8 mg, 0.01 mmol), 2,6-di-*tert*-butylpyridium hydrochloride (**18**) (2.5 mg, 0.01 mmol) and toluene (10 mL). An oil bubbler was attached to the Schlenk tube and the mixture was stirred at 50 °C (bath temperature) for 1 h under a gentle flow of Ar. The volatiles were evaporated under vacuum and the residue was purified flash chromatography (SiO_2, hexanes/EtOAc = 5:1) to give the title compound as a white crystalline material (205 mg, 92%). ^1H NMR (400 MHz, $CDCl_3$) δ 7.72 (d, J = 8.0 Hz, 2H), 7.31 (d, J = 7.9 Hz, 2H), 5.65 (s, 2H), 4.12 (s, 4H), 2.42 (s, 3H). $^{13}C\{^1H\}$ NMR (101 MHz, $CDCl_3$) δ 143.6, 134.4, 129.9, 127.6, 125.6, 55.0, 21.7.

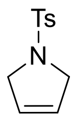

The spectroscopic data match the literature.[17]

2-Methyl-6-[(triethylsilyl)oxy]bicyclo[4.3.0]nona-2,9-diene (22) A Schlenk flask was charged with triethyl((5-(prop-1-yn-1-yl)nona-1,8-dien-5-yl)oxy)silane (146 mg, 0.5 mmol), complex **2** (10.8 mg, 0.0125 mmol), PCy_3 (7.0 mg, 0.025 mmol), 2,6-di-*tert*-butylpyridium hydrochloride (**18**) (5.7 mg, 0.025 mmol) and toluene (10 mL). An oil bubbler was attached to the Schlenk flask and the mixture was stirred at 50 °C (bath temperature) for 2 h under a gentle flow of Ar. The volatiles were evaporated under vacuum and the residue was purified by flash chromatography (SiO_2, pretreated with Et_3N; hexanes/Et_3N = 100:1) to give the title compound as a colorless oil (106 mg, 80%). ^1H NMR (400 MHz, C_6D_6) δ 5.49 (br d, J = 6.5 Hz, 1H), 5.43 (br s, 1H), 2.65 – 2.51 (m, 2H), 2.17 – 2.03 (m, 4H), 1.79 (q, J = 1.1 Hz, 3H), 1.77 – 1.68 (m, 1H), 1.48 – 1.40 (m, 1H), 1.02 (t, J = 7.9 Hz, 9H), 0.57 (q, J = 8.0 Hz, 6H). $^{13}C\{^1H\}$ NMR (101 MHz, C_6D_6) δ 145.7, 128.8, 126.7, 124.4, 83.4, 39.6, 37.6, 30.1, 24.2, 19.4, 7.6, 6.5.

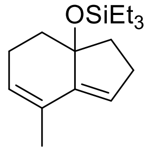

The spectroscopic data match the literature.[18]

Oxacyclohenicos-11-en-2-one (23) A three-neck flask equipped with a reflux condenser and

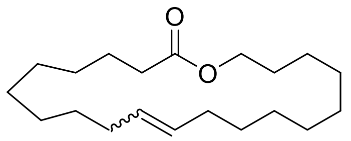

an oil bubbler was charged with undec-10-en-1-yl undec-10-enoate (133 mg, 0.4 mmol) and toluene (100 mL). Complex **2** (17.2 mg, 0.02 mmol), PCy_3 (11.2 mg, 0.04 mmol), and 2,6-di-*tert*-butylpyridium hydrochloride (**18**) (9.1 mg, 0.04 mmol) were added in 2 portions at the beginning and after 16 h, respectively. The mixture was stirred at 50 °C for 30 h. After cooling to room temperature, the brown solution was filtered through a plug of silica, which was carefully rinsed with toluene. The filtrate was evaporated under vacuum to afford a pale yellow oil which was purified by flash chromatography (silica, hexanes/EtOAc = 50 :1) to give the title compound as a colorless oil (96 mg, 78%, *E*/*Z* = 2:1). ^{1}H NMR (400 MHz, $CDCl_3$) δ 5.39 – 5.28 (m, 2H), 4.11 (td, *J* = 5.8, 3.4 Hz, 2H), 2.36 – 2.26 (m, 2H), 2.07 – 1.93 (m, 4H), 1.68 – 1.57 (m, 4H), 1.41 – 1.22 (m, 22H). ^{13}C{^{1}H} NMR (101 MHz, $CDCl_3$) δ 174.2, 174.1, 131.1, 130.8, 130.3, 130.2, 64.4, 64.1, 35.0, 34.7, 32.1, 31.9, 29.6, 29.6, 29.5, 29.4, 29.3, 29.2, 29.2, 29.2, 29.0, 29.0, 28.9, 28.9, 28.8, 28.7, 28.7, 28.6, 28.5, 28.1, 27.8, 26.8, 26.7, 26.3, 26.0, 25.4, 25.3.
The spectroscopic data match the literature.[19]

4-Phenylbut-2-en-1-yl acetate (24). A Schlenk flask was charged with *cis*-1,4-diacetoxy-2-

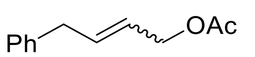

butene (344 mg, 2.0 mmol), allylbenzene (118 mg, 1.0 mmol), complex **2** (21.5 mg, 0.025 mmol), PCy_3 (14.0 mg, 0.05 mmol), 2,6-di-*tert*-butylpyridium hydrochloride (**18**) (11.4 mg, 0.05 mmol), and toluene (10 mL). An oil bubbler was attached to the Schlenk flask and the mixture was stirred at 50 °C (bath temperature) for 12 h under a gentle stream of Ar. The volatiles were evaporated under vacuum. The residue was purified by flash chromotography (silica, hexanes/EtOAc = 50 :1) to give the title compound as a colorless oil (136 mg, 71%, *E*:*Z* = 3.9:1). ^{1}H NMR (400 MHz, $CDCl_3$) δ 7.33 – 7.27 (m, 2H), 7.25 – 7.11 (m, 3H), [5.99 – 5.88 (m, 1H, *E*-isomer), 5.88 – 5.78 (m, 1H, *Z*-isomer)], 5.74 – 5.57 (m, 1H), [4.78 – 4.64 (m, 2H, *Z*-isomer), 4.55 (dq, *J* = 6.4, 1.1 Hz, 2H, *E*-isomer)], [3.48 (dd, *J* = 7.5, 1.5 Hz, 2H, *Z*-isomer), 3.41 (d, *J* = 6.7 Hz, 2H, *E*-isomer)], [2.08 (s, 3H, *Z*-isomer), 2.07 (s, 3H, *E*-isomer)]. ^{13}C{^{1}H} NMR (101 MHz, $CDCl_3$) δ 171.0, 139.7, 134.6, 133.6, 128.7, 128.7, 128.6, 128.6, 128.5, 126.4, 126.3, 125.4, 124.4, 65.0, 60.3, 38.8, 33.9, 21.1.
The spectroscopic data match the literature.[20]

3-Methyl-5-pentylfuran-2(5*H*)-one (38). A Schlenk flask was charged with complex **2** (8.6

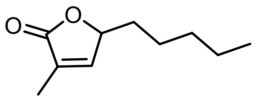

mg, 0.01 mmol), IPr (15.5 mg, 0.04 mmol) and toluene (10 mL). The resulting mixture was stirred at 50°C (bath temperature) for 30 min. 2-Methylacrylic acid 1-vinyl-hexylester (78 mg, 0.4 mmol) and [$HPCy_3$]Cl (6.3 mg, 0.02 mmol) were added and the resulting mixture was stirred at 80°C (bath temperature) for 2 h under a gentle stream of Ar. After reaching ambient temperature, all volatile materials were evaporated and the residue was purified by flash chromatography (silica, hexane/EtOAc = 30:1 to 10:1) to give the title compound as a pale brown oil (50 mg, 74%). The NMR spectrum of the crude product indicated ≈18% of remaining substrate. ^{1}H NMR (400 MHz, $CDCl_3$) δ 7.02 (app quint., *J* = 1.6 Hz, 1H), 5.08 – 4.70 (m, 1H), 1.91 (t, *J* = 1.8 Hz, 3H),

1.76 – 1.54 (m, 2H), 1.53 – 1.35 (m, 2H), 1.36 – 1.24 (m, 4H), 0.95 – 0.81 (m, 3H). $^{13}C\{^1H\}$ NMR (101 MHz, $CDCl_3$) δ 174.5, 148.9, 129.9, 81.3, 33.6, 31.6, 24.8, 22.6, 14.1, 10.8.
The spectroscopic data match the literature.[21]

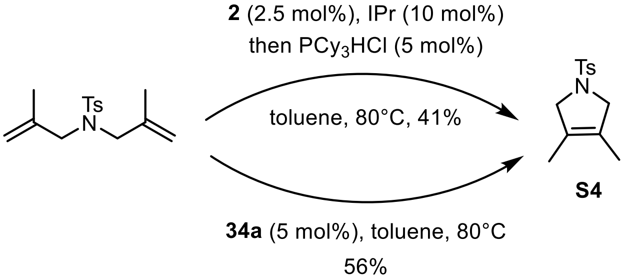

This challenging example was chosen to show that catalyst testing under "instant" metathesis conditions mirrors the results obtained with isolated complexes.

3,4-Dimethyl-1-tosyl-2,5-dihydro-1*H*-pyrrole (**S4**). **Method A**: A Schlenk flask was charged with 4-methyl-*N*,*N*-bis(2-methylallyl)benzenesulfonamide (140 mg, 0.500 mmol), Hoveyda-Grubbs catalyst **34a** (17.7 mg, 0.025 mmol) and toluene (5 mL). An oil bubbler was attached to the Schlenk flask and the mixture was stirred at 80 °C (bath temperature) for 20 h under a gentle flow of Ar. The volatiles were evaporated under vacuum and the residue was purified by flash chromatography (SiO_2; hexanes/EtOAc = 20:1 to 10:1) to give the title compound as a white solid (71 mg, 56%).

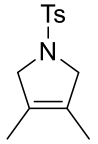

Method B: A Schlenk flask was charged with {Ru[=C(*p*-tolyl)TMS]Cl(NCMe)$_2$}(μ-Cl)$_2$ (**2**) (10.8 mg, 0.0125 mmol), IPr (19.4 mg, 0.050 mmol) and toluene (5 mL). The resulting mixture was stirred at 50 °C (bath temperature) for 30 min. 4-Methyl-*N*,*N*-bis(2-methylallyl)benzenesulfonamide (140 mg, 0.500 mmol) and [$HPCy_3$]Cl (8.0 mg, 0.025 mmol) were added to the solution. An oil bubbler was attached to the Schlenk flask and the mixture was stirred at 80 °C (bath temperature) for 20 h under a gentle flow of Ar. The volatiles were evaporated under vacuum and the residue was purified by flash chromatography (SiO_2; hexanes/EtOAc = 20:1 to 10:1) to give the title compound as a white solid (52 mg, 41%).
^1H NMR (400 MHz, $CDCl_3$) δ 7.72 (app d, J = 8.3 Hz, 2H), 7.32 (app d, J = 7.6 Hz, 2H), 3.97 (s, 4H), 2.42 (s, 3H), 1.54 (s, 6H). $^{13}C\{^1H\}$ NMR (101 MHz, $CDCl_3$) δ 143.4, 134.4, 129.8, 127.6, 126.4, 59.0, 21.7, 11.3.
The spectroscopic data match the literature.[22]

Supporting Crystallographic Information

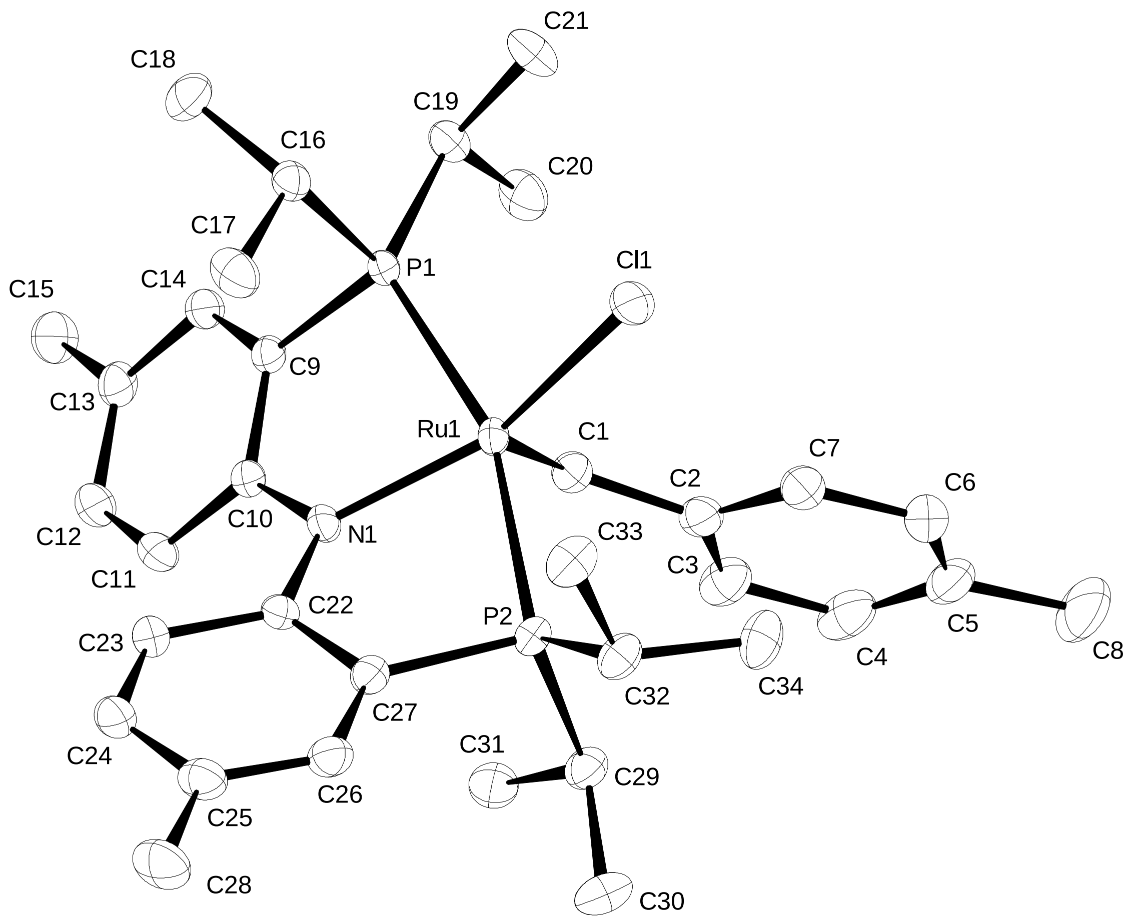

Figure S1. Crystallographic numbering scheme of the Ru carbene complex **7**. The thermal ellipsoid plot is shown at the 50% probability level, H-atoms omitted for clarity

X-ray Crystal Structure Analysis of Complex 7: $C_{34} H_{48} Cl N P_2 Ru$, M_r = 669.19 g · mol^{-1}, colorless plate, crystal size 0.186 x 0.116 x 0.06 mm^3, monoclinic, space group *C*2/c**,** [15], *a* = 37.5011(19) Å, *b* = 9.5596(4) Å, *c* = 18.6217(9) Å, β = 101.357(3)°, *V* = 6545.1(5) Å^3, *T* = 150(2) K, *Z* = 8, D_{calc} = 1.358 g · cm^3, λ = 0.71073 Å, $\mu(Mo\text{-}K_\alpha)$ = 0.682 mm^{-1}, analytical absorption correction ($T_{\min}$ = 0.93, $T_{\max}$ = 0.98), Bruker-AXS D8 VENTURE diffractometer with APEX-II detector and IµS micro focus X-ray source, 2.201 < θ < 31.513°, 505846 measured reflections, 10889 independent reflections, 8996 reflections with $I > 2\sigma(I)$, R_{int} = 0.1478, 367 parameters, *S* = 1.048, residual electron density +0.9 (0.63 Å from Ru1) / -0.9 (0.79 Å from Ru1) e · Å^{-3}. The structure was solved by *SHELXT* and refined by full-matrix least-squares (*SHELXL*) against F^2 to R_1 = 0.035 [$I > 2\sigma(I)$], wR_2 = 0.083. **CCDC-2431917**

Table S1. Crystallographic details for complex **7**

Identification code	15932	
Empirical formula	$C_{34} H_{48} Cl N P_2 Ru$	
Color	colourless	
Formula weight	669.19 g · mol^{-1}	
Temperature	100(2) K	
Wavelength	0.71073 Å	
Crystal system	MONOCLINIC	
Space group	**C2/c, (no. 15)**	
Unit cell dimensions	a = 37.5011(19) Å	α= 90°.
	b = 9.5596(4) Å	β= 101.357(3)°.
	c = 18.6217(9) Å	γ = 90°.
Volume	6545.1(5) Å^3	
Z	8	
Density (calculated)	1.358 Mg · m^{-3}	
Absorption coefficient	0.682 mm^{-1}	
F(000)	2800 e	
Crystal size	0.186 x 0.116 x 0.06 mm^3	
θ range for data collection	2.201 to 31.513°.	
Index ranges	-54 ≤ h ≤ 54, -14≤ k ≤ 14, -27 ≤ l ≤ 27	
Reflections collected	505846	
Independent reflections	10889 [R_{int} = 0.1478]	
Reflections with I>2σ(I)	8996	
Completeness to θ = 25.242°	100.0 %	
Absorption correction	Gaussian	
Max. and min. transmission	0.98 and 0.93	
Refinement method	Full-matrix least-squares on F^2	
Data / restraints / parameters	10889 / 0 / 367	
Goodness-of-fit on F^2	1.048	
Final R indices [I>2σ(I)]	R_1 = 0.0352	wR^2 = 0.0768
R indices (all data)	R_1 = 0.0490	wR^2 = 0.0834
Largest diff. peak and hole	0.9 and -0.9 e · Å^{-3}	

Table S2. Bond lengths [Å] and angles [°] of complex **7**

Ru(1)-P(1)	2.3167(5)	Ru(1)-Cl(1)	2.3878(5)
Ru(1)-P(2)	2.3706(5)	Ru(1)-N(1)	2.0522(15)
Ru(1)-C(1)	1.8449(19)	P(1)-C(16)	1.8477(19)
P(1)-C(9)	1.8142(18)	P(1)-C(19)	1.8403(19)
P(2)-C(32)	1.838(2)	P(2)-C(27)	1.809(2)
P(2)-C(29)	1.862(2)	N(1)-C(10)	1.401(2)
N(1)-C(22)	1.397(2)	C(10)-C(9)	1.410(3)
C(10)-C(11)	1.405(3)	C(14)-C(9)	1.393(2)
C(14)-C(13)	1.397(3)	C(16)-C(17)	1.529(3)
C(16)-C(18)	1.529(3)	C(19)-C(21)	1.533(3)
C(19)-C(20)	1.525(3)	C(22)-C(27)	1.416(3)
C(22)-C(23)	1.412(3)	C(13)-C(12)	1.391(3)
C(13)-C(15)	1.511(3)	C(32)-C(33)	1.527(3)
C(32)-C(34)	1.524(3)	C(26)-C(27)	1.397(3)
C(26)-C(25)	1.393(3)	C(2)-C(1)	1.466(3)
C(2)-C(3)	1.396(3)	C(2)-C(7)	1.386(3)
C(25)-C(24)	1.390(3)	C(25)-C(28)	1.508(3)
C(1)-H(1)	1.02(3)	C(29)-C(30)	1.530(3)
C(29)-C(31)	1.535(3)	C(12)-C(11)	1.385(3)
C(23)-C(24)	1.387(3)	C(3)-C(4)	1.377(3)
C(7)-C(6)	1.398(3)	C(4)-C(5)	1.389(4)
C(5)-C(6)	1.402(4)	C(5)-C(8)	1.506(3)
P(1)-Ru(1)-Cl(1)	93.396(17)	P(1)-Ru(1)-P(2)	159.848(18)
P(2)-Ru(1)-Cl(1)	101.478(17)	N(1)-Ru(1)-P(1)	80.37(4)
N(1)-Ru(1)-Cl(1)	153.83(5)	N(1)-Ru(1)-P(2)	80.31(4)
C(1)-Ru(1)-P(1)	92.36(6)	C(1)-Ru(1)-Cl(1)	107.55(6)
C(1)-Ru(1)-P(2)	95.98(6)	C(1)-Ru(1)-N(1)	98.14(8)
C(16)-P(1)-Ru(1)	112.20(6)	C(9)-P(1)-Ru(1)	99.34(6)
C(9)-P(1)-C(16)	105.69(8)	C(9)-P(1)-C(19)	107.83(9)
C(19)-P(1)-Ru(1)	126.57(7)	C(19)-P(1)-C(16)	103.55(9)
C(32)-P(2)-Ru(1)	123.39(7)	C(32)-P(2)-C(29)	105.49(9)
C(27)-P(2)-Ru(1)	96.92(6)	C(27)-P(2)-C(32)	105.98(9)
C(27)-P(2)-C(29)	103.92(9)	C(29)-P(2)-Ru(1)	118.21(7)
C(10)-N(1)-Ru(1)	119.01(12)	C(22)-N(1)-Ru(1)	117.78(12)
C(22)-N(1)-C(10)	122.46(15)	N(1)-C(10)-C(9)	118.65(16)
N(1)-C(10)-C(11)	123.88(17)	C(11)-C(10)-C(9)	117.34(17)
C(9)-C(14)-C(13)	121.31(18)	C(17)-C(16)-P(1)	110.52(14)
C(17)-C(16)-C(18)	111.24(18)	C(18)-C(16)-P(1)	112.75(14)
C(10)-C(9)-P(1)	112.89(13)	C(14)-C(9)-P(1)	126.14(14)
C(14)-C(9)-C(10)	120.97(17)	C(21)-C(19)-P(1)	110.20(13)
C(20)-C(19)-P(1)	111.59(14)	C(20)-C(19)-C(21)	110.87(17)

N(1)-C(22)-C(27)	118.45(16)	N(1)-C(22)-C(23)	124.22(17)
C(23)-C(22)-C(27)	117.15(17)	C(14)-C(13)-C(15)	121.12(18)
C(12)-C(13)-C(14)	117.32(17)	C(12)-C(13)-C(15)	121.51(18)
C(33)-C(32)-P(2)	109.76(14)	C(34)-C(32)-P(2)	109.69(14)
C(34)-C(32)-C(33)	111.22(18)	C(25)-C(26)-C(27)	122.05(19)
C(3)-C(2)-C(1)	119.3(2)	C(7)-C(2)-C(1)	122.47(19)
C(7)-C(2)-C(3)	118.0(2)	C(22)-C(27)-P(2)	114.45(14)
C(26)-C(27)-P(2)	125.00(15)	C(26)-C(27)-C(22)	120.53(18)
C(26)-C(25)-C(28)	121.2(2)	C(24)-C(25)-C(26)	117.03(18)
C(24)-C(25)-C(28)	121.8(2)	Ru(1)-C(1)-H(1)	115.8(14)
C(2)-C(1)-Ru(1)	130.27(15)	C(2)-C(1)-H(1)	113.9(14)
C(30)-C(29)-P(2)	113.56(14)	C(30)-C(29)-C(31)	109.92(18)
C(31)-C(29)-P(2)	110.11(14)	C(11)-C(12)-C(13)	122.31(18)
C(24)-C(23)-C(22)	120.68(19)	C(12)-C(11)-C(10)	120.70(18)
C(23)-C(24)-C(25)	122.55(19)	C(4)-C(3)-C(2)	121.5(2)
C(2)-C(7)-C(6)	120.6(2)	C(3)-C(4)-C(5)	121.2(2)
C(4)-C(5)-C(6)	117.6(2)	C(4)-C(5)-C(8)	120.8(3)
C(6)-C(5)-C(8)	121.6(3)	C(7)-C(6)-C(5)	121.0(2)

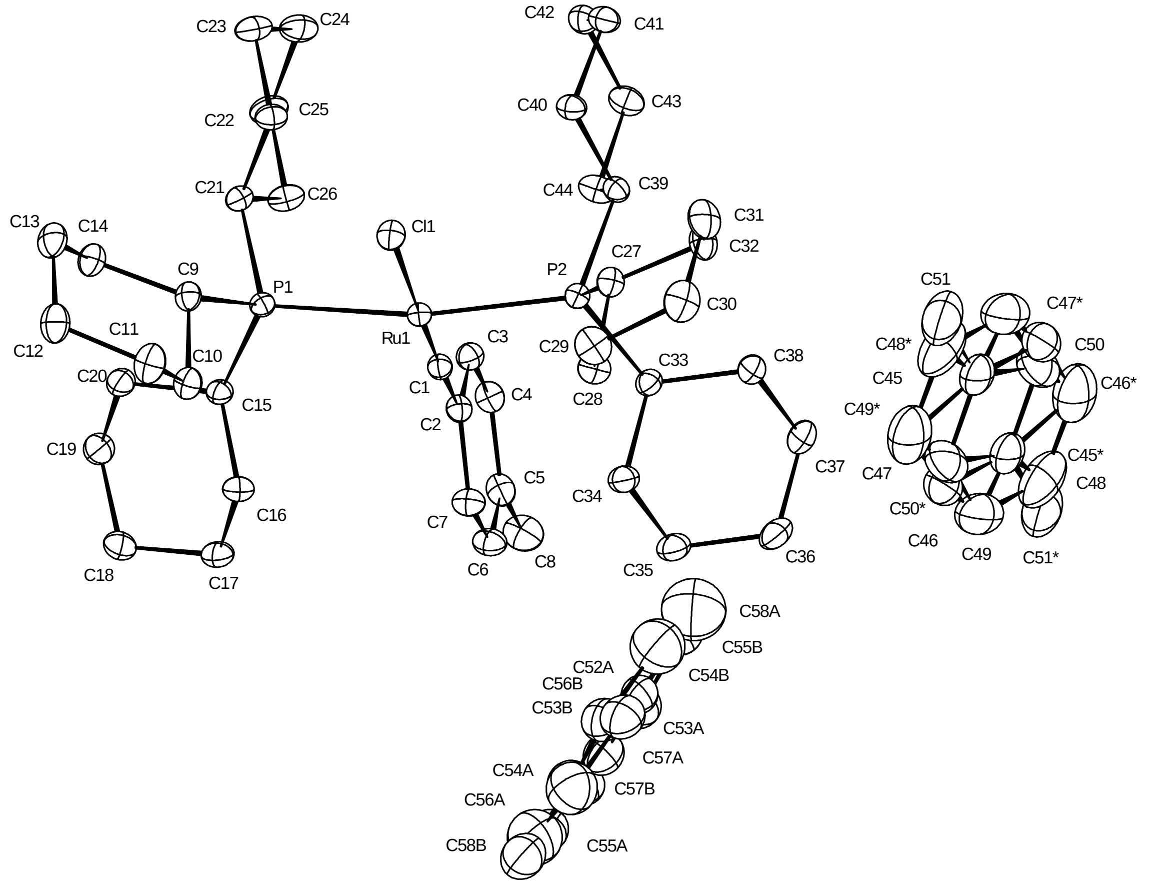

Figure S2. Crystallographic numbering scheme of the Ru alkylidyne complex **9**. The thermal ellipsoid plot is shown at the 50% probability level, H-atoms omitted for clarity

X-ray Crystal Structure Analysis of Complex 9: C_{109} H_{170} Cl_2 P_4 Ru_2, M_r = 1877.36 g · mol^{-1}, clear green prism, crystal size 0.205 x 0.121 x 0.07 mm^3, monoclinic, space group $P2_1/c$ [14], a = 9.4494(3) Å, b = 26.9549(10) Å, c = 20.0675(7) Å, β = 96.968(2)°, V = 5073.6(3) Å^3, T = 100(2) K, Z = 2, D_{calc} = 1.229 g · cm^3, λ = 0.71073 Å, $\mu(Mo\text{-}K_\alpha)$ = 0.459 mm^{-1}, analytical absorption correction ($T_{\min}$ = 0.90, $T_{\max}$ = 0.99), Bruker-AXS D8 VENTURE diffractometer with APEX-II detector and IµS micro focus X-ray source, 1.824 < θ < 28.571°, 399139 measured reflections, 12803 independent reflections, 10803 reflections with $I > 2\sigma(I)$, R_{int} = 0.1370, 627 parameters, S = 1.037, residual electron density +1.5 (0.57 Å from C56A) / -0.6 (0.42 Å from C58A) e · Å^{-3}. The structure was solved by *SHELXT* and refined by full-matrix least-squares (*SHELXL*) against F^2 to R_1 = 0.029 [$I > 2\sigma(I)$], wR_2 = 0.079. **CCDC-2431916**

Table S3. Crystallographic details for complex **9**

Identification code	15816	
Empirical formula	$C_{109} H_{170} Cl_2 P_4 Ru_2$	
Color	clear green	
Formula weight	1877.36 g·mol^{-1}	
Temperature	100(2) K	
Wavelength	0.71073 Å	
Crystal system	Monoclinic	
Space group	$P2_1/c$, (no. 14)	
Unit cell dimensions	a = 9.4494(3) Å	α= 90°.
	b = 26.9549(10) Å	β= 96.968(2)°.
	c = 20.0675(7) Å	γ = 90°.
Volume	5073.6(3) Å^3	
Z	2	
Density (calculated)	1.229 Mg·m^{-3}	
Absorption coefficient	0.459 mm^{-1}	
F(000)	2012 e	
Crystal size	0.205 x 0.121 x 0.07 mm^3	
θ range for data collection	1.824 to 28.571°.	
Index ranges	-12 ≤ h ≤ 12, -35≤ k ≤ 36, -26 ≤ l ≤ 26	
Reflections collected	399139	
Independent reflections	12803 [R_{int} = 0.1370]	
Reflections with I>2σ(I)	10803	
Completeness to θ = 25.242°	100.0 %	
Absorption correction	Numerical	
Max. and min. transmission	0.9875 and 0.9037	
Refinement method	Full-matrix least-squares on F^2	
Data / restraints / parameters	12803 / 492 / 627	
Goodness-of-fit on F^2	1.037	
Final R indices [I>2σ(I)]	R_1 = 0.0294	wR^2 = 0.0740
R indices (all data)	R_1 = 0.0415	wR^2 = 0.0789
Extinction coefficient	n/a	
Largest diff. peak and hole	1.452 and -0.623 e·Å^{-3}	

Table S4. Bond lengths [Å] and angles [°] of complex **9**

Ru(1)-Cl(1)	2.4357(4)	Ru(1)-P(1)	2.3845(4)
Ru(1)-P(2)	2.3878(4)	Ru(1)-C(1)	1.7103(16)
P(1)-C(9)	1.8489(16)	P(1)-C(15)	1.8518(16)
P(1)-C(21)	1.8538(16)	P(2)-C(27)	1.8457(16)
P(2)-C(33)	1.8514(17)	P(2)-C(39)	1.8530(16)
C(1)-C(2)	1.445(2)	C(2)-C(3)	1.402(2)
C(2)-C(7)	1.403(2)	C(3)-C(4)	1.384(2)
C(4)-C(5)	1.394(2)	C(5)-C(6)	1.397(2)
C(5)-C(8)	1.504(2)	C(6)-C(7)	1.384(2)
C(9)-C(10)	1.531(2)	C(9)-C(14)	1.534(2)
C(10)-C(11)	1.531(2)	C(11)-C(12)	1.525(3)
C(12)-C(13)	1.526(3)	C(13)-C(14)	1.532(2)
C(15)-C(16)	1.534(2)	C(15)-C(20)	1.536(2)
C(16)-C(17)	1.529(2)	C(17)-C(18)	1.526(3)
C(18)-C(19)	1.526(3)	C(19)-C(20)	1.534(2)
C(21)-C(22)	1.534(2)	C(21)-C(26)	1.532(2)
C(22)-C(23)	1.531(2)	C(23)-C(24)	1.524(3)
C(24)-C(25)	1.524(2)	C(25)-C(26)	1.527(2)
C(27)-C(28)	1.537(2)	C(27)-C(32)	1.537(2)
C(28)-C(29)	1.524(2)	C(29)-C(30)	1.528(3)
C(30)-C(31)	1.526(3)	C(31)-C(32)	1.534(2)
C(33)-C(34)	1.536(2)	C(33)-C(38)	1.538(2)
C(34)-C(35)	1.528(2)	C(35)-C(36)	1.526(3)
C(36)-C(37)	1.526(3)	C(37)-C(38)	1.529(3)
C(39)-C(40)	1.536(2)	C(39)-C(44)	1.534(2)
C(40)-C(41)	1.534(2)	C(41)-C(42)	1.523(2)
C(42)-C(43)	1.526(3)	C(43)-C(44)	1.531(2)
C(52B)-C(53B)	1.362(8)	C(52B)-C(57B)	1.465(7)
C(52B)-C(58B)	1.403(10)	53B)-C(54B)	1.452(9)
C(54B)-C(55B)	1.473(8)	C(55B)-C(56B)	1.379(7)
C(56B)-C(57B)	1.444(7)	C(52A)-C(53A)	1.339(6)
C(52A)-C(57A)	1.428(5)	C(52A)-C(58A)	1.502(7)
C(53A)-C(54A)	1.350(6)	C(54A)-C(55A)	1.296(7)
C(55A)-C(56A)	1.289(7)	C(56A)-C(57A)	1.457(5)
C(45)-C(46)	1.375(7)	C(45)-C(50)	1.386(7)
C(45)-C(51)	1.495(7)	C(46)-C(47)	1.388(8)
C(47)-C(48)	1.379(8)	C(48)-C(49)	1.373(8)
C(49)-C(50)	1.381(7)		
P(1)-Ru(1)-Cl(1)	89.312(14)	P(1)-Ru(1)-P(2)	169.282(15)
P(2)-Ru(1)-Cl(1)	88.908(14)	C(1)-Ru(1)-Cl(1)	178.12(5)
C(1)-Ru(1)-P(1)	90.79(5)	C(1)-Ru(1)-P(2)	90.64(5)

C(9)-P(1)-Ru(1)	114.19(5)	C(9)-P(1)-C(15)	110.40(7)
C(9)-P(1)-C(21)	103.43(7)	C(15)-P(1)-Ru(1)	115.17(5)
C(15)-P(1)-C(21)	102.66(7)	C(21)-P(1)-Ru(1)	109.69(5)
C(27)-P(2)-Ru(1)	114.94(5)	C(27)-P(2)-C(33)	110.15(8)
C(27)-P(2)-C(39)	103.05(7)	C(33)-P(2)-Ru(1)	114.49(5)
C(33)-P(2)-C(39)	103.13(7)	C(39)-P(2)-Ru(1)	109.79(5)
C(2)-C(1)-Ru(1)	178.20(13)	C(3)-C(2)-C(1)	120.91(15)
C(3)-C(2)-C(7)	118.52(15)	C(7)-C(2)-C(1)	120.56(15)
C(4)-C(3)-C(2)	120.47(15)	C(3)-C(4)-C(5)	121.04(16)
C(4)-C(5)-C(6)	118.53(15)	C(4)-C(5)-C(8)	120.62(16)
C(6)-C(5)-C(8)	120.84(16)	C(7)-C(6)-C(5)	120.93(16)
C(6)-C(7)-C(2)	120.50(15)	C(10)-C(9)-P(1)	112.49(11)
C(10)-C(9)-C(14)	110.04(14)	C(14)-C(9)-P(1)	119.94(12)
C(11)-C(10)-C(9)	109.65(14)	C(12)-C(11)-C(10)	111.19(15)
C(11)-C(12)-C(13)	111.76(15)	C(12)-C(13)-C(14)	111.97(15)
C(13)-C(14)-C(9)	109.52(14)	C(16)-C(15)-P(1)	114.12(11)
C(16)-C(15)-C(20)	110.19(13)	C(20)-C(15)-P(1)	116.23(11)
C(17)-C(16)-C(15)	109.74(14)	C(18)-C(17)-C(16)	111.34(14)
C(19)-C(18)-C(17)	110.81(14)	C(18)-C(19)-C(20)	111.23(15)
C(19)-C(20)-C(15)	110.74(14)	C(22)-C(21)-P(1)	110.32(11)
C(26)-C(21)-P(1)	111.34(11)	C(26)-C(21)-C(22)	109.47(13)
C(23)-C(22)-C(21)	111.03(14)	C(24)-C(23)-C(22)	111.46(15)
C(25)-C(24)-C(23)	110.92(15)	C(24)-C(25)-C(26)	111.11(14)
C(25)-C(26)-C(21)	111.72(14)	C(28)-C(27)-P(2)	112.07(11)
C(32)-C(27)-P(2)	120.67(12)	C(32)-C(27)-C(28)	109.37(14)
C(29)-C(28)-C(27)	109.66(14)	C(28)-C(29)-C(30)	110.89(16)
C(31)-C(30)-C(29)	111.81(15)	C(30)-C(31)-C(32)	111.87(16)
C(31)-C(32)-C(27)	109.18(14)	C(34)-C(33)-P(2)	113.30(11)
C(34)-C(33)-C(38)	109.84(14)	C(38)-C(33)-P(2)	116.58(11)
C(35)-C(34)-C(33)	110.45(14)	C(36)-C(35)-C(34)	111.36(15)
C(37)-C(36)-C(35)	111.05(15)	C(36)-C(37)-C(38)	110.93(16)
C(37)-C(38)-C(33)	110.78(15)	C(40)-C(39)-P(2)	111.18(11)
C(44)-C(39)-P(2)	111.01(11)	C(44)-C(39)-C(40)	109.73(14)
C(41)-C(40)-C(39)	110.55(14)	C(42)-C(41)-C(40)	110.56(14)
C(41)-C(42)-C(43)	110.76(14)	C(42)-C(43)-C(44)	111.08(14)
C(43)-C(44)-C(39)	112.17(14)	C(53B)-C(52B)-C(57B)	125.9(6)
C(53B)-C(52B)-C(58B)	117.4(7)	C(58B)-C(52B)-C(57B)	116.5(6)
C(52B)-C(53B)-C(54B)	119.3(7)	C(53B)-C(54B)-C(55B)	124.9(7)
C(56B)-C(55B)-C(54B)	102.6(6)	C(55B)-C(56B)-C(57B)	144.3(6)
C(56B)-C(57B)-C(52B)	103.1(5)	C(53A)-C(52A)-C(57A)	115.5(4)
C(53A)-C(52A)-C(58A)	114.8(5)	C(57A)-C(52A)-C(58A)	129.7(5)
C(52A)-C(53A)-C(54A)	123.8(5)	C(55A)-C(54A)-C(53A)	119.0(6)
C(56A)-C(55A)-C(54A)	125.9(6)	C(55A)-C(56A)-C(57A)	116.5(5)
C(46)-C(45)-C(50)	117.6(5)	C(46)-C(45)-C(51)	121.4(6)

C(50)-C(45)-C(51)	121.0(6)	C(45)-C(46)-C(47)	122.4(6)
C(48)-C(47)-C(46)	118.6(6)	C(49)-C(48)-C(47)	120.2(7)
C(48)-C(49)-C(50)	120.2(6)	C(49)-C(50)-C(45)	121.0(7)

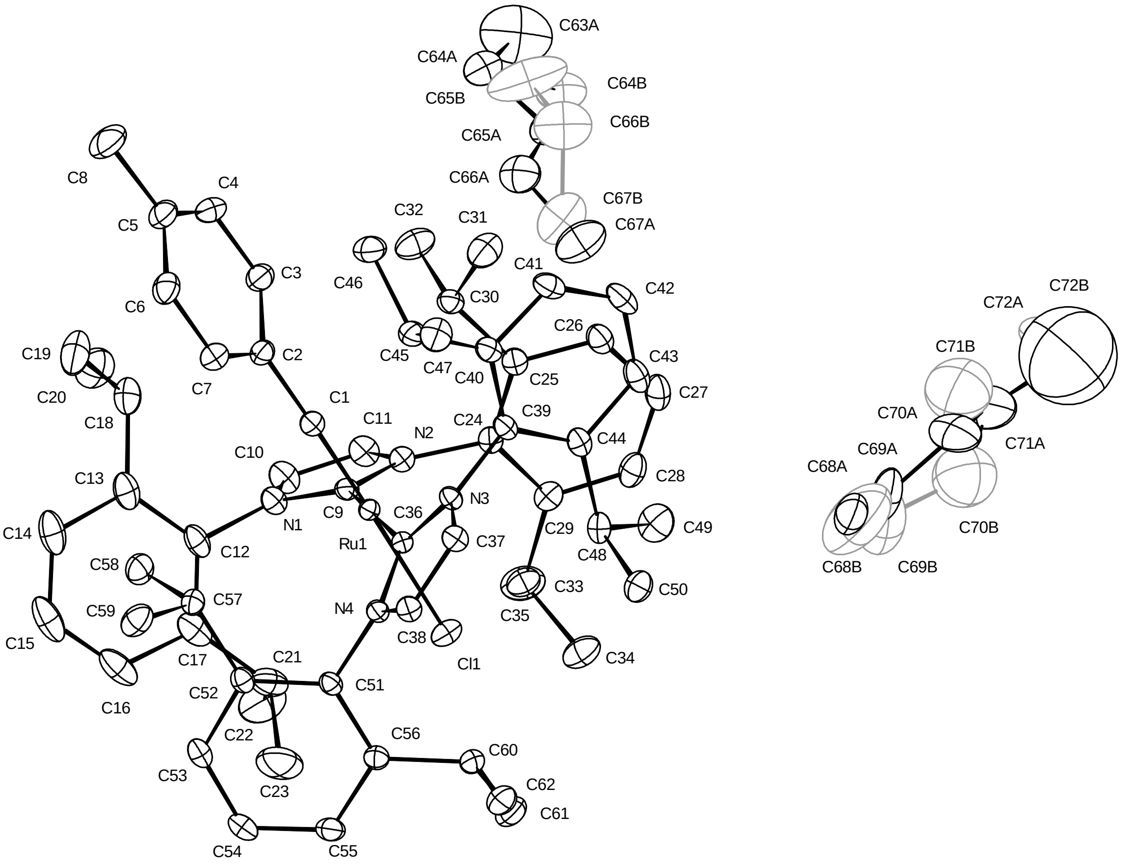

Figure S3. Crystallographic numbering scheme of the Ru alkylidyne complex **26**. The thermal ellipsoid plot is shown at the 50% probability level, H-atoms omitted for clarity

X-ray Crystal Structure Analysis of Complex 26: C_{72} H_{103} Cl N_4 Ru, M_r = 1161.10 g·mol^{-1}, green prism, crystal size 0.254 x 0.221 x 0.200 mm^3, monoclinic, space group *C*2/*c* [15], *a* = 29.1245(8) Å, *b* = 12.5484(4) Å, *c* = 37.3598(10) Å, β = 102.7620(10)°, *V* = 13316.4(7) Å^3, *T* = 100(2) K, *Z* = 8, D_{calc} = 1.158 g·cm^3, λ = 0.71073 Å, $\mu(Mo\text{-}K_\alpha)$ = 0.318 mm^{-1}, Gaussian absorption correction ($T_{\min}$ = 0.95, $T_{\max}$ = 0.96), Bruker AXS D8-Venture diffractometer with IµS Diamond Mo-anode X-ray source and PHOTON III detector, 2.003 < θ < 33.142°, 653774 measured reflections, 25410 independent reflections, 20025 reflections with $I > 2\sigma(I)$, R_{int} = 0.0985. The structure was solved by *SHELXT* and refined by full-matrix least-squares (*SHELXL*) against F^2 to R_1 = 0.0400 [$I > 2\sigma(I)$], wR_2 = 0.0865, 818 parameters, 60 restraints. **CCDC-2515554**

Table S5. Crystallographic details for complex **26**

Identification code	16524	
Empirical formula	C_{72} H_{103} Cl N_4 Ru	
Color	green	
Formula weight	1161.10 g·mol^{-1}	
Temperature	100(2) K	
Wavelength	0.71073 Å	
Crystal system	Monoclinic	
Space group	*C*2/*c*, (no. 15)	
Unit cell dimensions	a = 29.1245(8) Å	α= 90°.
	b = 12.5484(4) Å	β= 102.7620(10)°.
	c = 37.3598(10) Å	γ = 90°.
Volume	13316.4(7) Å^3	
Z	8	
Density (calculated)	1.158 Mg·m^{-3}	
Absorption coefficient	0.318 mm^{-1}	
F(000)	4992 e	
Crystal size	0.254 x 0.221 x 0.200 mm^3	
θ range for data collection	2.003 to 33.142°.	
Index ranges	-44 ≤ h ≤ 44, -19≤ k ≤ 19, -57 ≤ l ≤ 57	
Reflections collected	653774	
Independent reflections	25410 [R_{int} = 0.1379]	
Reflections with I>2σ(I)	20025	
Completeness to θ = 25.242°	100.0 %	
Absorption correction	Gaussian	
Max. and min. transmission	0.95671 and 0.94741	
Refinement method	Full-matrix least-squares on F^2	
Data / restraints / parameters	25410 / 60 / 818	
Goodness-of-fit on F^2	1.037	
Final R indices [I>2σ(I)]	R_1 = 0.0400	wR^2 = 0.0798
R indices (all data)	R_1 = 0.0613	wR^2 = 0.0865
Extinction coefficient	n/a	
Largest diff. peak and hole	0.634 and -0.666 e·Å^{-3}	

Table S6. Bond lengths [Å] and angles [°] of complex **26**

Ru(1)-Cl(1)	2.4199(3)	Ru(1)-C(1)	1.7178(13)
Ru(1)-C(9)	2.1076(13)	Ru(1)-C(36)	2.1106(13)
N(1)-C(9)	1.3725(17)	N(1)-C(10)	1.3912(18)
N(1)-C(12)	1.4470(19)	N(2)-C(9)	1.3694(17)
N(2)-C(11)	1.3911(17)	N(2)-C(24)	1.4487(18)
N(3)-C(36)	1.3732(16)	N(3)-C(37)	1.3923(16)
N(3)-C(39)	1.4490(17)	N(4)-C(36)	1.3704(16)
N(4)-C(38)	1.3885(17)	N(4)-C(51)	1.4444(17)
C(1)-C(2)	1.4456(19)	C(2)-C(3)	1.405(2)
C(2)-C(7)	1.402(2)	C(3)-C(4)	1.384(2)
C(4)-C(5)	1.393(2)	C(5)-C(6)	1.392(2)
C(5)-C(8)	1.507(2)	C(6)-C(7)	1.387(2)
C(10)-C(11)	1.338(2)	C(12)-C(13)	1.402(2)
C(12)-C(17)	1.405(2)	C(13)-C(14)	1.396(2)
C(13)-C(18)	1.514(2)	C(14)-C(15)	1.374(3)
C(15)-C(16)	1.381(3)	C(16)-C(17)	1.402(2)
C(17)-C(21)	1.512(3)	C(18)-C(19)	1.532(2)
C(18)-C(20)	1.542(2)	C(21)-C(22)	1.531(3)
C(21)-C(23)	1.526(2)	C(24)-C(25)	1.400(2)
C(24)-C(29)	1.4064(19)	C(25)-C(26)	1.396(2)
C(25)-C(30)	1.518(2)	C(26)-C(27)	1.387(2)
C(27)-C(28)	1.380(2)	C(28)-C(29)	1.395(2)
C(29)-C(33)	1.518(2)	C(30)-C(31)	1.522(2)
C(30)-C(32)	1.530(2)	C(33)-C(34)	1.533(2)
C(33)-C(35)	1.536(2)	C(37)-C(38)	1.3398(19)
C(39)-C(40)	1.4029(19)	C(39)-C(44)	1.4097(19)
C(40)-C(41)	1.399(2)	C(40)-C(45)	1.518(2)
C(41)-C(42)	1.379(2)	C(42)-C(43)	1.384(2)
C(43)-C(44)	1.394(2)	C(44)-C(48)	1.516(2)
C(45)-C(46)	1.536(2)	C(45)-C(47)	1.538(2)
C(48)-C(49)	1.525(2)	C(48)-C(50)	1.534(2)
C(51)-C(52)	1.3998(18)	C(51)-C(56)	1.4082(18)
C(52)-C(53)	1.3959(19)	C(52)-C(57)	1.5186(19)
C(53)-C(54)	1.386(2)	C(54)-C(55)	1.387(2)
C(55)-C(56)	1.395(2)	C(56)-C(60)	1.5205(19)
C(57)-C(58)	1.5278(19)	C(57)-C(59)	1.534(2)
C(60)-C(61)	1.533(2)	C(60)-C(62)	1.534(2)
C(63A)-C(64A)	1.442(14)	C(64A)-C(65A)	1.505(5)
C(65A)-C(66A)	1.503(5)	C(66A)-C(67A)	1.503(6)
C(63B)-C(64B)	1.629(12)	C(64B)-C(65B)	1.485(7)
C(65B)-C(66B)	1.464(7)	C(66B)-C(67B)	1.477(10)
C(68A)-C(69A)	1.439(11)	C(69A)-C(70A)	1.465(11)

C(70A)-C(71A)	1.607(8)	C(71A)-C(72A)	1.58(2)
C(68B)-C(69B)	1.63(2)	C(69B)-C(70B)	1.530(16)
C(70B)-C(71B)	1.386(12)	C(71B)-C(72B)	1.788(14)

C(1)-Ru(1)-Cl(1)	178.67(5)	C(1)-Ru(1)-C(9)	94.49(6)
C(1)-Ru(1)-C(36)	92.69(5)	C(9)-Ru(1)-Cl(1)	84.98(4)
C(9)-Ru(1)-C(36)	172.76(5)	C(36)-Ru(1)-Cl(1)	87.86(4)
C(9)-N(1)-C(10)	111.68(12)	C(9)-N(1)-C(12)	126.85(11)
C(10)-N(1)-C(12)	121.06(12)	C(9)-N(2)-C(11)	111.95(12)
C(9)-N(2)-C(24)	129.54(11)	C(11)-N(2)-C(24)	118.42(11)
C(36)-N(3)-C(37)	111.77(11)	C(36)-N(3)-C(39)	127.65(11)
C(37)-N(3)-C(39)	119.72(11)	C(36)-N(4)-C(38)	112.09(11)
C(36)-N(4)-C(51)	127.46(11)	C(38)-N(4)-C(51)	120.26(11)
C(2)-C(1)-Ru(1)	178.32(11)	C(3)-C(2)-C(1)	120.99(13)
C(7)-C(2)-C(1)	120.81(12)	C(7)-C(2)-C(3)	118.20(13)
C(4)-C(3)-C(2)	120.63(14)	C(3)-C(4)-C(5)	121.20(14)
C(4)-C(5)-C(8)	120.79(15)	C(6)-C(5)-C(4)	118.17(14)
C(6)-C(5)-C(8)	121.03(16)	C(7)-C(6)-C(5)	121.40(14)
C(6)-C(7)-C(2)	120.40(14)	N(1)-C(9)-Ru(1)	129.36(10)
N(2)-C(9)-Ru(1)	127.85(9)	N(2)-C(9)-N(1)	102.77(11)
C(11)-C(10)-N(1)	106.93(12)	C(10)-C(11)-N(2)	106.67(12)
C(13)-C(12)-N(1)	119.21(14)	C(13)-C(12)-C(17)	122.72(14)
C(17)-C(12)-N(1)	117.82(14)	C(12)-C(13)-C(18)	122.55(14)
C(14)-C(13)-C(12)	117.45(16)	C(14)-C(13)-C(18)	119.97(15)
C(15)-C(14)-C(13)	121.19(17)	C(14)-C(15)-C(16)	120.47(16)
C(15)-C(16)-C(17)	121.32(17)	C(12)-C(17)-C(21)	121.99(14)
C(16)-C(17)-C(12)	116.80(16)	C(16)-C(17)-C(21)	121.12(16)
C(13)-C(18)-C(19)	112.65(15)	C(13)-C(18)-C(20)	110.68(15)
C(19)-C(18)-C(20)	109.11(15)	C(17)-C(21)-C(22)	110.39(14)
C(17)-C(21)-C(23)	113.16(16)	C(23)-C(21)-C(22)	110.48(16)
C(25)-C(24)-N(2)	119.04(12)	C(25)-C(24)-C(29)	122.50(13)
C(29)-C(24)-N(2)	117.82(12)	C(24)-C(25)-C(30)	121.01(12)
C(26)-C(25)-C(24)	117.75(13)	C(26)-C(25)-C(30)	121.18(13)
C(27)-C(26)-C(25)	120.82(15)	C(28)-C(27)-C(26)	120.23(14)
C(27)-C(28)-C(29)	121.45(14)	C(24)-C(29)-C(33)	122.93(13)
C(28)-C(29)-C(24)	117.19(14)	C(28)-C(29)-C(33)	119.87(13)
C(25)-C(30)-C(31)	113.09(12)	C(25)-C(30)-C(32)	110.24(12)
C(31)-C(30)-C(32)	110.94(13)	C(29)-C(33)-C(34)	111.77(14)
C(29)-C(33)-C(35)	111.14(13)	C(34)-C(33)-C(35)	109.71(13)
N(3)-C(36)-Ru(1)	128.72(9)	N(4)-C(36)-Ru(1)	128.61(9)
N(4)-C(36)-N(3)	102.62(11)	C(38)-C(37)-N(3)	106.79(11)
C(37)-C(38)-N(4)	106.72(11)	C(40)-C(39)-N(3)	119.12(12)
C(40)-C(39)-C(44)	122.73(13)	C(44)-C(39)-N(3)	117.60(12)
C(39)-C(40)-C(45)	123.49(12)	C(41)-C(40)-C(39)	117.29(14)

C(41)-C(40)-C(45)	119.15(13)	C(42)-C(41)-C(40)	121.29(14)
C(41)-C(42)-C(43)	120.14(14)	C(42)-C(43)-C(44)	121.61(15)
C(39)-C(44)-C(48)	122.05(12)	C(43)-C(44)-C(39)	116.94(14)
C(43)-C(44)-C(48)	120.93(13)	C(40)-C(45)-C(46)	111.72(13)
C(40)-C(45)-C(47)	110.65(12)	C(46)-C(45)-C(47)	109.38(13)
C(44)-C(48)-C(49)	113.10(13)	C(44)-C(48)-C(50)	110.97(12)
C(49)-C(48)-C(50)	109.99(12)	C(52)-C(51)-N(4)	119.45(12)
C(52)-C(51)-C(56)	122.69(12)	C(56)-C(51)-N(4)	117.64(11)
C(51)-C(52)-C(57)	122.79(12)	C(53)-C(52)-C(51)	117.38(13)
C(53)-C(52)-C(57)	119.79(12)	C(54)-C(53)-C(52)	121.33(13)
C(53)-C(54)-C(55)	120.03(13)	C(54)-C(55)-C(56)	121.18(13)
C(51)-C(56)-C(60)	122.42(12)	C(55)-C(56)-C(51)	117.35(12)
C(55)-C(56)-C(60)	120.23(12)	C(52)-C(57)-C(58)	111.33(12)
C(52)-C(57)-C(59)	111.04(12)	C(58)-C(57)-C(59)	110.36(12)
C(56)-C(60)-C(61)	112.45(13)	C(56)-C(60)-C(62)	111.50(12)
C(61)-C(60)-C(62)	108.90(12)	C(63A)-C(64A)-C(65A)	107.4(7)
C(66A)-C(65A)-C(64A)	114.1(3)	C(67A)-C(66A)-C(65A)	113.7(5)
C(65B)-C(64B)-C(63B)	109.0(6)	C(66B)-C(65B)-C(64B)	120.2(5)
C(65B)-C(66B)-C(67B)	117.5(6)	C(68A)-C(69A)-C(70A)	112.1(7)
C(69A)-C(70A)-C(71A)	110.3(5)	C(72A)-C(71A)-C(70A)	97.1(9)
C(70B)-C(69B)-C(68B)	113.4(11)	C(71B)-C(70B)-C(69B)	110.9(10)
C(70B)-C(71B)-C(72B)	111.7(8)		

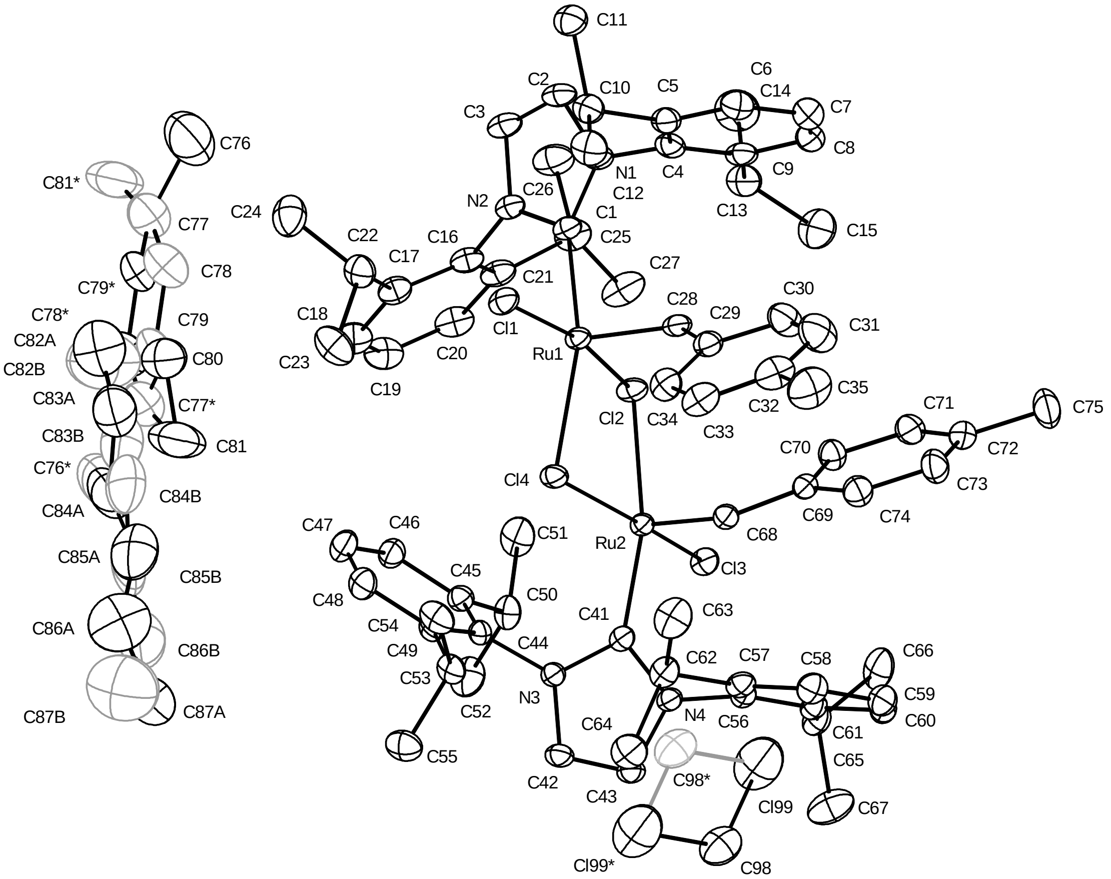

Figure S4. Crystallographic numbering scheme of the dinuclear Ru carbene complex **[29]₂**. The thermal ellipsoid plot is shown at the 50% probability level, H-atoms omitted for clarity

X-ray Crystal Structure Analysis of Complex [29]₂: C_{159} H_{216} Cl_{10} N_8 Ru_4, M_r = 2998.17 g·mol^{-1}, green plate, crystal size 0.212 x 0.161 x 0.021 mm^3, monoclinic, space group $P2_1/c$ [14], a = 10.4626(5) Å, b = 25.3289(14) Å, c = 28.7414(15) Å, β = 93.106(3)°, V = 7605.5(7) Å^3, T = 100(2) K, Z = 2, D_{calc} = 1.309 g·cm^3, λ = 0.71073 Å, $\mu(Mo\text{-}K_\alpha)$ = 0.617 mm^{-1}, Gaussian absorption correction ($T_{\min}$ = 0.92, $T_{\max}$ = 0.99), Bruker AXS D8-Venture diffractometer with IµS Diamond Mo-anode X-ray source and PHOTON III detector, 2.145 < θ < 30.999°, 455641 measured reflections, 24221 independent reflections, 18545 reflections with $I > 2\sigma(I)$, R_{int} = 0.1438. The structure was solved by *SHELXT* and refined by full-matrix least-squares (*SHELXL*) against F^2 to R_1 = 0.0593 [$I > 2\sigma(I)$], wR_2 = 0.1415, 934 parameters, 255 restraints. **CCDC-2515555**

Table S7. Crystallographic details for complex **[29]**$_2$

Identification code	16633	
Empirical formula	C_{159} H_{216} Cl_{10} N_8 Ru_4	
Color	green	
Formula weight	2998.17 g·mol^{-1}	
Temperature	100(2) K	
Wavelength	0.71073 Å	
Crystal system	Monoclinic	
Space group	$P2_1/c$, (no. 14)	
Unit cell dimensions	a = 10.4626(5) Å	α= 90°.
	b = 25.3289(14) Å	β= 93.106(3)°.
	c = 28.7414(15) Å	γ = 90°.
Volume	7605.5(7) Å^3	
Z	2	
Density (calculated)	1.309 Mg·m^{-3}	
Absorption coefficient	0.617 mm^{-1}	
F(000)	3144 e	
Crystal size	0.212 x 0.161 x 0.021 mm^3	
θ range for data collection	2.145 to 30.999°.	
Index ranges	-15 ≤ h ≤ 15, -36≤ k ≤ 36, -41 ≤ l ≤ 41	
Reflections collected	455641	
Independent reflections	24221 [R_{int} = 0.1438]	
Reflections with I>2σ(I)	18545	
Completeness to θ = 25.242°	99.9 %	
Absorption correction	Gaussian	
Max. and min. transmission	0.98879 and 0.92101	
Refinement method	Full-matrix least-squares on F^2	
Data / restraints / parameters	24221 / 255 / 934	
Goodness-of-fit on F^2	1.105	
Final R indices [I>2σ(I)]	R_1 = 0.0593	wR^2 = 0.1311
R indices (all data)	R_1 = 0.0841	wR^2 = 0.1415
Extinction coefficient	n/a	
Largest diff. peak and hole	1.351 and -1.205 e·Å^{-3}	

Table S8. Bond lengths [Å] and angles [°] of complex **[29]₂**

Ru(1)-Cl(1)	2.3436(9)	Ru(1)-Cl(2)	2.3992(8)
Ru(1)-Cl(4)	2.4357(8)	Ru(1)-C(1)	2.007(3)
Ru(1)-C(28)	1.828(4)	Ru(2)-Cl(2)	2.4430(8)
Ru(2)-Cl(3)	2.3485(8)	Ru(2)-Cl(4)	2.4016(8)
Ru(2)-C(41)	2.003(3)	Ru(2)-C(68)	1.833(3)
N(1)-C(1)	1.367(4)	N(1)-C(2)	1.389(4)
N(1)-C(4)	1.444(5)	N(2)-C(1)	1.367(4)
N(2)-C(3)	1.394(4)	N(2)-C(16)	1.457(5)
N(3)-C(41)	1.374(4)	N(3)-C(42)	1.384(4)
N(3)-C(44)	1.445(4)	N(4)-C(41)	1.366(4)
N(4)-C(43)	1.399(4)	N(4)-C(56)	1.446(4)
C(2)-H(2)	0.9500	C(2)-C(3)	1.334(5)
C(3)-H(3)	0.9500	C(4)-C(5)	1.392(5)
C(4)-C(9)	1.406(5)	C(5)-C(6)	1.392(6)
C(5)-C(10)	1.523(5)	C(6)-H(6)	0.9500
C(6)-C(7)	1.379(6)	C(7)-H(7)	0.9500
C(7)-C(8)	1.387(6)	C(8)-H(8)	0.9500
C(8)-C(9)	1.384(6)	C(9)-C(13)	1.521(5)
C(10)-H(10)	1.0000	C(10)-C(11)	1.536(5)
C(10)-C(12)	1.524(6)	C(11)-H(11A)	0.9800
C(11)-H(11B)	0.9800	C(11)-H(11C)	0.9800
C(12)-H(12A)	0.9800	C(12)-H(12B)	0.9800
C(12)-H(12C)	0.9800	C(13)-H(13)	1.0000
C(13)-C(14)	1.528(6)	C(13)-C(15)	1.529(6)
C(14)-H(14A)	0.9800	C(14)-H(14B)	0.9800
C(14)-H(14C)	0.9800	C(15)-H(15A)	0.9800
C(15)-H(15B)	0.9800	C(15)-H(15C)	0.9800
C(16)-C(17)	1.393(5)	C(16)-C(21)	1.412(5)
C(17)-C(18)	1.397(6)	C(17)-C(22)	1.512(5)
C(18)-C(19)	1.389(6)	C(19)-H(19)	0.9500
C(19)-C(20)	1.374(6)	C(20)-H(20)	0.9500
C(20)-C(21)	1.393(6)	C(21)-C(25)	1.517(6)
C(22)-C(23)	1.534(6)	C(22)-C(24)	1.533(6)
C(23)-H(23A)	0.9800	C(23)-H(23B)	0.9800
C(23)-H(23C)	0.9800	C(24)-H(24A)	0.9800
C(24)-H(24B)	0.9800	C(24)-H(24C)	0.9800
C(25)-H(25)	1.0000	C(25)-C(26)	1.542(5)
C(25)-C(27)	1.534(5)	C(26)-H(26A)	0.9800
C(26)-H(26B)	0.9800	C(26)-H(26C)	0.9800
C(27)-H(27A)	0.9800	C(27)-H(27B)	0.9800
C(27)-H(27C)	0.9800	C(28)-C(29)	1.455(5)
C(28)-H(28)	0.97(4)	C(29)-C(30)	1.402(5)

C(29)-C(34)	1.408(5)	C(30)-H(30)	0.9500
C(30)-C(31)	1.392(6)	C(31)-H(31)	0.9500
C(31)-C(32)	1.398(7)	C(32)-C(33)	1.375(6)
C(32)-C(35)	1.508(6)	C(33)-H(33)	0.9500
C(33)-C(34)	1.386(5)	C(34)-H(34)	0.9500
C(35)-H(35A)	0.9800	C(35)-H(35B)	0.9800
C(35)-H(35C)	0.9800	C(42)-H(42)	0.9500
C(42)-C(43)	1.334(5)	C(43)-H(43)	0.9500
C(44)-C(45)	1.400(5)	C(44)-C(49)	1.407(4)
C(45)-C(46)	1.400(5)	C(45)-C(50)	1.508(5)
C(46)-H(46)	0.9500	C(46)-C(47)	1.383(5)
C(47)-H(47)	0.9500	C(47)-C(48)	1.381(5)
C(48)-H(48)	0.9500	C(48)-C(49)	1.394(5)
C(49)-C(53)	1.517(5)	C(50)-H(50)	1.0000
C(50)-C(51)	1.538(5)	C(50)-C(52)	1.524(6)
C(51)-H(51A)	0.9800	C(51)-H(51B)	0.9800
C(51)-H(51C)	0.9800	C(52)-H(52A)	0.9800
C(52)-H(52B)	0.9800	C(52)-H(52C)	0.9800
C(53)-H(53)	1.0000	C(53)-C(54)	1.534(5)
C(53)-C(55)	1.535(5)	C(54)-H(54A)	0.9800
C(54)-H(54B)	0.9800	C(54)-H(54C)	0.9800
C(55)-H(55A)	0.9800	C(55)-H(55B)	0.9800
C(55)-H(55C)	0.9800	C(56)-C(57)	1.406(5)
C(56)-C(61)	1.400(5)	C(57)-C(58)	1.397(5)
C(57)-C(62)	1.515(5)	C(58)-H(58)	0.9500
C(58)-C(59)	1.383(5)	C(59)-H(59)	0.9500
C(59)-C(60)	1.381(6)	C(60)-H(60)	0.9500
C(60)-C(61)	1.395(5)	C(61)-C(65)	1.511(5)
C(62)-H(62)	1.0000	C(62)-C(63)	1.528(6)
C(62)-C(64)	1.540(5)	C(63)-H(63A)	0.9800
C(63)-H(63B)	0.9800	C(63)-H(63C)	0.9800
C(64)-H(64A)	0.9800	C(64)-H(64B)	0.9800
C(64)-H(64C)	0.9800	C(65)-H(65)	1.0000
C(65)-C(66)	1.526(5)	C(65)-C(67)	1.523(6)
C(66)-H(66A)	0.9800	C(66)-H(66B)	0.9800
C(66)-H(66C)	0.9800	C(67)-H(67A)	0.9800
C(67)-H(67B)	0.9800	C(67)-H(67C)	0.9800
C(68)-C(69)	1.458(5)	C(68)-H(68)	0.97(4)
C(69)-C(70)	1.397(5)	C(69)-C(74)	1.395(5)
C(70)-H(70)	0.9500	C(70)-C(71)	1.398(5)
C(71)-H(71)	0.9500	C(71)-C(72)	1.383(6)
C(72)-C(73)	1.402(6)	C(72)-C(75)	1.507(5)
C(73)-H(73)	0.9500	C(73)-C(74)	1.383(5)
C(74)-H(74)	0.9500	C(75)-H(75A)	0.9800
C(75)-H(75B)	0.9800	C(75)-H(75C)	0.9800

Cl(99)-C(98)#1	1.750(10)	Cl(99)-C(98)	1.684(11)
C(98)-H(98A)	0.9900	C(98)-H(98B)	0.9900
C(82A)-H(82A)	0.9800	C(82A)-H(82B)	0.9800
C(82A)-H(82C)	0.9800	C(82A)-C(83A)	1.433(12)
C(83A)-H(83A)	0.9900	C(83A)-H(83B)	0.9900
C(83A)-C(84A)	1.482(11)	C(84A)-H(84A)	0.9900
C(84A)-H(84B)	0.9900	C(84A)-C(85A)	1.424(11)
C(85A)-H(85A)	0.9900	C(85A)-H(85B)	0.9900
C(85A)-C(86A)	1.353(11)	C(86A)-H(86A)	0.9900
C(86A)-H(86B)	0.9900	C(86A)-C(87A)	1.44(2)
C(87A)-H(87A)	0.9800	C(87A)-H(87B)	0.9800
C(87A)-H(87C)	0.9800	C(82B)-H(82D)	0.9800
C(82B)-H(82E)	0.9800	C(82B)-H(82F)	0.9800
C(82B)-C(83B)	1.434(12)	C(83B)-H(83C)	0.9900
C(83B)-H(83D)	0.9900	C(83B)-C(84B)	1.452(12)
C(84B)-H(84C)	0.9900	C(84B)-H(84D)	0.9900
C(84B)-C(85B)	1.391(11)	C(85B)-H(85C)	0.9900
C(85B)-H(85D)	0.9900	C(85B)-C(86B)	1.320(10)
C(86B)-H(86C)	0.9900	C(86B)-H(86D)	0.9900
C(86B)-C(87B)	1.420(12)	C(87B)-H(87D)	0.9800
C(87B)-H(87E)	0.9800	C(87B)-H(87F)	0.9800
C(76)-H(76A)	0.9800	C(76)-H(76B)	0.9800
C(76)-H(76C)	0.9800	C(76)-C(77)	1.480(10)
C(77)-H(77A)	0.9900	C(77)-H(77B)	0.9900
C(77)-C(78)	1.448(10)	C(78)-H(78A)	0.9900
C(78)-H(78B)	0.9900	C(78)-C(79)	1.445(9)
C(79)-H(79A)	0.9900	C(79)-H(79B)	0.9900
C(79)-C(80)	1.487(11)	C(80)-H(80A)	0.9900
C(80)-H(80B)	0.9900	C(80)-C(81)	1.479(10)
C(81)-H(81A)	0.9800	C(81)-H(81B)	0.9800
C(81)-H(81C)	0.9800		

Cl(1)-Ru(1)-Cl(2)	168.89(3)	Cl(1)-Ru(1)-Cl(4)	90.66(3)
Cl(2)-Ru(1)-Cl(4)	83.05(3)	C(1)-Ru(1)-Cl(1)	88.50(9)
C(1)-Ru(1)-Cl(2)	94.78(9)	C(1)-Ru(1)-Cl(4)	162.71(11)
C(28)-Ru(1)-Cl(1)	99.81(11)	C(28)-Ru(1)-Cl(2)	90.43(11)
C(28)-Ru(1)-Cl(4)	101.26(10)	C(28)-Ru(1)-C(1)	95.90(14)
Cl(3)-Ru(2)-Cl(2)	89.74(3)	Cl(3)-Ru(2)-Cl(4)	166.44(3)
Cl(4)-Ru(2)-Cl(2)	82.84(3)	C(41)-Ru(2)-Cl(2)	163.49(10)
C(41)-Ru(2)-Cl(3)	88.57(9)	C(41)-Ru(2)-Cl(4)	95.31(9)
C(68)-Ru(2)-Cl(2)	101.45(10)	C(68)-Ru(2)-Cl(3)	101.53(11)
C(68)-Ru(2)-Cl(4)	91.10(11)	C(68)-Ru(2)-C(41)	94.98(14)
Ru(1)-Cl(2)-Ru(2)	94.74(3)	Ru(2)-Cl(4)-Ru(1)	94.87(3)
C(1)-N(1)-C(2)	111.0(3)	C(1)-N(1)-C(4)	126.9(3)
C(2)-N(1)-C(4)	121.3(3)	C(1)-N(2)-C(3)	111.3(3)

C(1)-N(2)-C(16)	126.1(3)	C(3)-N(2)-C(16)	122.0(3)
C(41)-N(3)-C(42)	111.0(3)	C(41)-N(3)-C(44)	123.0(3)
C(42)-N(3)-C(44)	124.3(3)	C(41)-N(4)-C(43)	110.3(3)
C(41)-N(4)-C(56)	127.6(3)	C(43)-N(4)-C(56)	121.3(3)
N(1)-C(1)-Ru(1)	133.0(3)	N(2)-C(1)-Ru(1)	122.3(2)
N(2)-C(1)-N(1)	103.6(3)	N(1)-C(2)-H(2)	126.3
C(3)-C(2)-N(1)	107.4(3)	C(3)-C(2)-H(2)	126.3
N(2)-C(3)-H(3)	126.7	C(2)-C(3)-N(2)	106.6(3)
C(2)-C(3)-H(3)	126.7	C(5)-C(4)-N(1)	119.4(3)
C(5)-C(4)-C(9)	123.0(4)	C(9)-C(4)-N(1)	117.4(3)
C(4)-C(5)-C(6)	117.0(3)	C(4)-C(5)-C(10)	122.8(3)
C(6)-C(5)-C(10)	120.2(3)	C(5)-C(6)-H(6)	119.2
C(7)-C(6)-C(5)	121.5(4)	C(7)-C(6)-H(6)	119.2
C(6)-C(7)-H(7)	120.0	C(6)-C(7)-C(8)	120.0(4)
C(8)-C(7)-H(7)	120.0	C(7)-C(8)-H(8)	119.5
C(9)-C(8)-C(7)	121.0(4)	C(9)-C(8)-H(8)	119.5
C(4)-C(9)-C(13)	121.8(4)	C(8)-C(9)-C(4)	117.4(4)
C(8)-C(9)-C(13)	120.9(3)	C(5)-C(10)-H(10)	108.1
C(5)-C(10)-C(11)	109.6(3)	C(5)-C(10)-C(12)	111.8(3)
C(11)-C(10)-H(10)	108.1	C(12)-C(10)-H(10)	108.1
C(12)-C(10)-C(11)	111.0(3)	C(10)-C(11)-H(11A)	109.5
C(10)-C(11)-H(11B)	109.5	C(10)-C(11)-H(11C)	109.5
H(11A)-C(11)-H(11B)	109.5	H(11A)-C(11)-H(11C)	109.5
H(11B)-C(11)-H(11C)	109.5	C(10)-C(12)-H(12A)	109.5
C(10)-C(12)-H(12B)	109.5	C(10)-C(12)-H(12C)	109.5
H(12A)-C(12)-H(12B)	109.5	H(12A)-C(12)-H(12C)	109.5
H(12B)-C(12)-H(12C)	109.5	C(9)-C(13)-H(13)	107.4
C(9)-C(13)-C(14)	111.3(4)	C(9)-C(13)-C(15)	113.3(4)
C(14)-C(13)-H(13)	107.4	C(14)-C(13)-C(15)	109.8(4)
C(15)-C(13)-H(13)	107.4	C(13)-C(14)-H(14A)	109.5
C(13)-C(14)-H(14B)	109.5	C(13)-C(14)-H(14C)	109.5
H(14A)-C(14)-H(14B)	109.5	H(14A)-C(14)-H(14C)	109.5
H(14B)-C(14)-H(14C)	109.5	C(13)-C(15)-H(15A)	109.5
C(13)-C(15)-H(15B)	109.5	C(13)-C(15)-H(15C)	109.5
H(15A)-C(15)-H(15B)	109.5	H(15A)-C(15)-H(15C)	109.5
H(15B)-C(15)-H(15C)	109.5	C(17)-C(16)-N(2)	119.5(3)
C(17)-C(16)-C(21)	122.9(4)	C(21)-C(16)-N(2)	117.4(3)
C(16)-C(17)-C(18)	117.7(4)	C(16)-C(17)-C(22)	122.3(3)
C(18)-C(17)-C(22)	120.0(4)	C(19)-C(18)-C(17)	120.6(4)
C(18)-C(19)-H(19)	119.8	C(20)-C(19)-C(18)	120.4(4)
C(20)-C(19)-H(19)	119.8	C(19)-C(20)-H(20)	119.1
C(19)-C(20)-C(21)	121.8(4)	C(21)-C(20)-H(20)	119.1
C(16)-C(21)-C(25)	123.1(3)	C(20)-C(21)-C(16)	116.6(4)
C(20)-C(21)-C(25)	120.3(3)	C(17)-C(22)-C(23)	111.9(3)
C(17)-C(22)-C(24)	109.0(3)	C(24)-C(22)-C(23)	110.3(4)

C(22)-C(23)-H(23A)	109.5	C(22)-C(23)-H(23B)	109.5
C(22)-C(23)-H(23C)	109.5	H(23A)-C(23)-H(23B)	109.5
H(23A)-C(23)-H(23C)	109.5	H(23B)-C(23)-H(23C)	109.5
C(22)-C(24)-H(24A)	109.5	C(22)-C(24)-H(24B)	109.5
C(22)-C(24)-H(24C)	109.5	H(24A)-C(24)-H(24B)	109.5
H(24A)-C(24)-H(24C)	109.5	H(24B)-C(24)-H(24C)	109.5
C(21)-C(25)-H(25)	108.1	C(21)-C(25)-C(26)	110.1(3)
C(21)-C(25)-C(27)	113.8(3)	C(26)-C(25)-H(25)	108.1
C(27)-C(25)-H(25)	108.1	C(27)-C(25)-C(26)	108.6(3)
C(25)-C(26)-H(26A)	109.5	C(25)-C(26)-H(26B)	109.5
C(25)-C(26)-H(26C)	109.5	H(26A)-C(26)-H(26B)	109.5
H(26A)-C(26)-H(26C)	109.5	H(26B)-C(26)-H(26C)	109.5
C(25)-C(27)-H(27A)	109.5	C(25)-C(27)-H(27B)	109.5
C(25)-C(27)-H(27C)	109.5	H(27A)-C(27)-H(27B)	109.5
H(27A)-C(27)-H(27C)	109.5	H(27B)-C(27)-H(27C)	109.5
Ru(1)-C(28)-H(28)	117(2)	C(29)-C(28)-Ru(1)	130.2(3)
C(29)-C(28)-H(28)	113(2)	C(30)-C(29)-C(28)	118.0(3)
C(30)-C(29)-C(34)	117.8(4)	C(34)-C(29)-C(28)	124.2(3)
C(29)-C(30)-H(30)	119.4	C(31)-C(30)-C(29)	121.2(4)
C(31)-C(30)-H(30)	119.4	C(30)-C(31)-H(31)	119.8
C(30)-C(31)-C(32)	120.4(4)	C(32)-C(31)-H(31)	119.8
C(31)-C(32)-C(35)	121.1(4)	C(33)-C(32)-C(31)	118.2(4)
C(33)-C(32)-C(35)	120.7(4)	C(32)-C(33)-H(33)	118.8
C(32)-C(33)-C(34)	122.4(4)	C(34)-C(33)-H(33)	118.8
C(29)-C(34)-H(34)	120.1	C(33)-C(34)-C(29)	119.9(4)
C(33)-C(34)-H(34)	120.1	C(32)-C(35)-H(35A)	109.5
C(32)-C(35)-H(35B)	109.5	C(32)-C(35)-H(35C)	109.5
H(35A)-C(35)-H(35B)	109.5	H(35A)-C(35)-H(35C)	109.5
H(35B)-C(35)-H(35C)	109.5	N(3)-C(41)-Ru(2)	121.6(2)
N(4)-C(41)-Ru(2)	133.1(2)	N(4)-C(41)-N(3)	104.1(3)
N(3)-C(42)-H(42)	126.5	C(43)-C(42)-N(3)	107.0(3)
C(43)-C(42)-H(42)	126.5	N(4)-C(43)-H(43)	126.2
C(42)-C(43)-N(4)	107.5(3)	C(42)-C(43)-H(43)	126.2
C(45)-C(44)-N(3)	119.5(3)	C(45)-C(44)-C(49)	123.2(3)
C(49)-C(44)-N(3)	117.2(3)	C(44)-C(45)-C(46)	116.6(3)
C(44)-C(45)-C(50)	122.9(3)	C(46)-C(45)-C(50)	120.4(3)
C(45)-C(46)-H(46)	119.2	C(47)-C(46)-C(45)	121.5(3)
C(47)-C(46)-H(46)	119.2	C(46)-C(47)-H(47)	119.9
C(48)-C(47)-C(46)	120.2(3)	C(48)-C(47)-H(47)	119.9
C(47)-C(48)-H(48)	119.4	C(47)-C(48)-C(49)	121.3(3)
C(49)-C(48)-H(48)	119.4	C(44)-C(49)-C(53)	122.4(3)
C(48)-C(49)-C(44)	117.1(3)	C(48)-C(49)-C(53)	120.5(3)
C(45)-C(50)-H(50)	108.2	C(45)-C(50)-C(51)	111.9(3)
C(45)-C(50)-C(52)	109.0(3)	C(51)-C(50)-H(50)	108.2
C(52)-C(50)-H(50)	108.2	C(52)-C(50)-C(51)	111.3(3)

C(50)-C(51)-H(51A)	109.5	C(50)-C(51)-H(51B)	109.5
C(50)-C(51)-H(51C)	109.5	H(51A)-C(51)-H(51B)	109.5
H(51A)-C(51)-H(51C)	109.5	H(51B)-C(51)-H(51C)	109.5
C(50)-C(52)-H(52A)	109.5	C(50)-C(52)-H(52B)	109.5
C(50)-C(52)-H(52C)	109.5	H(52A)-C(52)-H(52B)	109.5
H(52A)-C(52)-H(52C)	109.5	H(52B)-C(52)-H(52C)	109.5
C(49)-C(53)-H(53)	107.9	C(49)-C(53)-C(54)	113.0(3)
C(49)-C(53)-C(55)	110.5(3)	C(54)-C(53)-H(53)	107.9
C(54)-C(53)-C(55)	109.6(3)	C(55)-C(53)-H(53)	107.9
C(53)-C(54)-H(54A)	109.5	C(53)-C(54)-H(54B)	109.5
C(53)-C(54)-H(54C)	109.5	H(54A)-C(54)-H(54B)	109.5
H(54A)-C(54)-H(54C)	109.5	H(54B)-C(54)-H(54C)	109.5
C(53)-C(55)-H(55A)	109.5	C(53)-C(55)-H(55B)	109.5
C(53)-C(55)-H(55C)	109.5	H(55A)-C(55)-H(55B)	109.5
H(55A)-C(55)-H(55C)	109.5	H(55B)-C(55)-H(55C)	109.5
C(57)-C(56)-N(4)	118.0(3)	C(61)-C(56)-N(4)	119.2(3)
C(61)-C(56)-C(57)	122.6(3)	C(56)-C(57)-C(62)	122.4(3)
C(58)-C(57)-C(56)	117.1(3)	C(58)-C(57)-C(62)	120.5(3)
C(57)-C(58)-H(58)	119.4	C(59)-C(58)-C(57)	121.3(3)
C(59)-C(58)-H(58)	119.4	C(58)-C(59)-H(59)	119.8
C(60)-C(59)-C(58)	120.3(4)	C(60)-C(59)-H(59)	119.8
C(59)-C(60)-H(60)	119.5	C(59)-C(60)-C(61)	121.0(3)
C(61)-C(60)-H(60)	119.5	C(56)-C(61)-C(65)	122.8(3)
C(60)-C(61)-C(56)	117.6(3)	C(60)-C(61)-C(65)	119.6(3)
C(57)-C(62)-H(62)	107.7	C(57)-C(62)-C(63)	113.3(3)
C(57)-C(62)-C(64)	110.6(3)	C(63)-C(62)-H(62)	107.7
C(63)-C(62)-C(64)	109.6(3)	C(64)-C(62)-H(62)	107.7
C(62)-C(63)-H(63A)	109.5	C(62)-C(63)-H(63B)	109.5
C(62)-C(63)-H(63C)	109.5	H(63A)-C(63)-H(63B)	109.5
H(63A)-C(63)-H(63C)	109.5	H(63B)-C(63)-H(63C)	109.5
C(62)-C(64)-H(64A)	109.5	C(62)-C(64)-H(64B)	109.5
C(62)-C(64)-H(64C)	109.5	H(64A)-C(64)-H(64B)	109.5
H(64A)-C(64)-H(64C)	109.5	H(64B)-C(64)-H(64C)	109.5
C(61)-C(65)-H(65)	107.9	C(61)-C(65)-C(66)	112.0(3)
C(61)-C(65)-C(67)	109.8(3)	C(66)-C(65)-H(65)	107.9
C(67)-C(65)-H(65)	107.9	C(67)-C(65)-C(66)	111.0(3)
C(65)-C(66)-H(66A)	109.5	C(65)-C(66)-H(66B)	109.5
C(65)-C(66)-H(66C)	109.5	H(66A)-C(66)-H(66B)	109.5
H(66A)-C(66)-H(66C)	109.5	H(66B)-C(66)-H(66C)	109.5
C(65)-C(67)-H(67A)	109.5	C(65)-C(67)-H(67B)	109.5
C(65)-C(67)-H(67C)	109.5	H(67A)-C(67)-H(67B)	109.5
H(67A)-C(67)-H(67C)	109.5	H(67B)-C(67)-H(67C)	109.5
Ru(2)-C(68)-H(68)	112(2)	C(69)-C(68)-Ru(2)	130.8(3)
C(69)-C(68)-H(68)	117(2)	C(70)-C(69)-C(68)	124.4(3)
C(74)-C(69)-C(68)	117.6(3)	C(74)-C(69)-C(70)	118.0(3)

C(69)-C(70)-H(70)	120.0	C(69)-C(70)-C(71)	120.1(3)
C(71)-C(70)-H(70)	120.0	C(70)-C(71)-H(71)	119.1
C(72)-C(71)-C(70)	121.9(4)	C(72)-C(71)-H(71)	119.1
C(71)-C(72)-C(73)	117.8(3)	C(71)-C(72)-C(75)	121.7(4)
C(73)-C(72)-C(75)	120.5(4)	C(72)-C(73)-H(73)	119.7
C(74)-C(73)-C(72)	120.7(4)	C(74)-C(73)-H(73)	119.7
C(69)-C(74)-H(74)	119.2	C(73)-C(74)-C(69)	121.6(4)
C(73)-C(74)-H(74)	119.2	C(72)-C(75)-H(75A)	109.5
C(72)-C(75)-H(75B)	109.5	C(72)-C(75)-H(75C)	109.5
H(75A)-C(75)-H(75B)	109.5	H(75A)-C(75)-H(75C)	109.5
H(75B)-C(75)-H(75C)	109.5	C(98)-Cl(99)-C(98)#1	65.4(5)
Cl(99)-C(98)-Cl(99)#1	114.6(5)	Cl(99)#1-C(98)-H(98A)	108.6
Cl(99)-C(98)-H(98A)	108.6	Cl(99)#1-C(98)-H(98B)	108.6
Cl(99)-C(98)-H(98B)	108.6	H(98A)-C(98)-H(98B)	107.6
H(82A)-C(82A)-H(82B)	109.5	H(82A)-C(82A)-H(82C)	109.5
H(82B)-C(82A)-H(82C)	109.5	C(83A)-C(82A)-H(82A)	109.5
C(83A)-C(82A)-H(82B)	109.5	C(83A)-C(82A)-H(82C)	109.5
C(82A)-C(83A)-H(83A)	107.5	C(82A)-C(83A)-H(83B)	107.5
C(82A)-C(83A)-C(84A)	119.4(14)	H(83A)-C(83A)-H(83B)	107.0
C(84A)-C(83A)-H(83A)	107.5	C(84A)-C(83A)-H(83B)	107.5
C(83A)-C(84A)-H(84A)	108.3	C(83A)-C(84A)-H(84B)	108.3
H(84A)-C(84A)-H(84B)	107.4	C(85A)-C(84A)-C(83A)	116.1(14)
C(85A)-C(84A)-H(84A)	108.3	C(85A)-C(84A)-H(84B)	108.3
C(84A)-C(85A)-H(85A)	100.2	C(84A)-C(85A)-H(85B)	100.2
H(85A)-C(85A)-H(85B)	104.2	C(86A)-C(85A)-C(84A)	146.4(16)
C(86A)-C(85A)-H(85A)	100.2	C(86A)-C(85A)-H(85B)	100.2
C(85A)-C(86A)-H(86A)	103.3	C(85A)-C(86A)-H(86B)	103.3
C(85A)-C(86A)-C(87A)	135.4(16)	H(86A)-C(86A)-H(86B)	105.2
C(87A)-C(86A)-H(86A)	103.3	C(87A)-C(86A)-H(86B)	103.3
C(86A)-C(87A)-H(87A)	109.5	C(86A)-C(87A)-H(87B)	109.5
C(86A)-C(87A)-H(87C)	109.5	H(87A)-C(87A)-H(87B)	109.5
H(87A)-C(87A)-H(87C)	109.5	H(87B)-C(87A)-H(87C)	109.5
H(82D)-C(82B)-H(82E)	109.5	H(82D)-C(82B)-H(82F)	109.5
H(82E)-C(82B)-H(82F)	109.5	C(83B)-C(82B)-H(82D)	109.5
C(83B)-C(82B)-H(82E)	109.5	C(83B)-C(82B)-H(82F)	109.5
C(82B)-C(83B)-H(83C)	106.7	C(82B)-C(83B)-H(83D)	106.7
C(82B)-C(83B)-C(84B)	122.6(17)	H(83C)-C(83B)-H(83D)	106.6
C(84B)-C(83B)-H(83C)	106.7	C(84B)-C(83B)-H(83D)	106.7
C(83B)-C(84B)-H(84C)	108.0	C(83B)-C(84B)-H(84D)	108.0
H(84C)-C(84B)-H(84D)	107.2	C(85B)-C(84B)-C(83B)	117.3(17)
C(85B)-C(84B)-H(84C)	108.0	C(85B)-C(84B)-H(84D)	108.0
C(84B)-C(85B)-H(85C)	95.7	C(84B)-C(85B)-H(85D)	95.7
H(85C)-C(85B)-H(85D)	103.3	C(86B)-C(85B)-C(84B)	161.6(18)
C(86B)-C(85B)-H(85C)	95.7	C(86B)-C(85B)-H(85D)	95.7
C(85B)-C(86B)-H(86C)	103.7	C(85B)-C(86B)-H(86D)	103.7

C(85B)-C(86B)-C(87B)	134.0(17)	H(86C)-C(86B)-H(86D)	105.3
C(87B)-C(86B)-H(86C)	103.7	C(87B)-C(86B)-H(86D)	103.7
C(86B)-C(87B)-H(87D)	109.5	C(86B)-C(87B)-H(87E)	109.5
C(86B)-C(87B)-H(87F)	109.5	H(87D)-C(87B)-H(87E)	109.5
H(87D)-C(87B)-H(87F)	109.5	H(87E)-C(87B)-H(87F)	109.5
H(76A)-C(76)-H(76B)	109.5	H(76A)-C(76)-H(76C)	109.5
H(76B)-C(76)-H(76C)	109.5	C(77)-C(76)-H(76A)	109.5
C(77)-C(76)-H(76B)	109.5	C(77)-C(76)-H(76C)	109.5
C(76)-C(77)-H(77A)	108.7	C(76)-C(77)-H(77B)	108.7
H(77A)-C(77)-H(77B)	107.6	C(78)-C(77)-C(76)	114.1(11)
C(78)-C(77)-H(77A)	108.7	C(78)-C(77)-H(77B)	108.7
C(77)-C(78)-H(78A)	104.0	C(77)-C(78)-H(78B)	104.0
H(78A)-C(78)-H(78B)	105.4	C(79)-C(78)-C(77)	133.1(13)
C(79)-C(78)-H(78A)	104.0	C(79)-C(78)-H(78B)	104.0
C(78)-C(79)-H(79A)	110.5	C(78)-C(79)-H(79B)	110.5
C(78)-C(79)-C(80)	106.1(10)	H(79A)-C(79)-H(79B)	108.7
C(80)-C(79)-H(79A)	110.5	C(80)-C(79)-H(79B)	110.5
C(79)-C(80)-H(80A)	108.2	C(79)-C(80)-H(80B)	108.2
H(80A)-C(80)-H(80B)	107.4	C(81)-C(80)-C(79)	116.2(11)
C(81)-C(80)-H(80A)	108.2	C(81)-C(80)-H(80B)	108.2
C(80)-C(81)-H(81A)	109.5	C(80)-C(81)-H(81B)	109.5
C(80)-C(81)-H(81C)	109.5	H(81A)-C(81)-H(81B)	109.5
H(81A)-C(81)-H(81C)	109.5	H(81B)-C(81)-H(81C)	109.5

Symmetry transformations used to generate equivalent atoms:
#1 -x,-y+1,-z+1

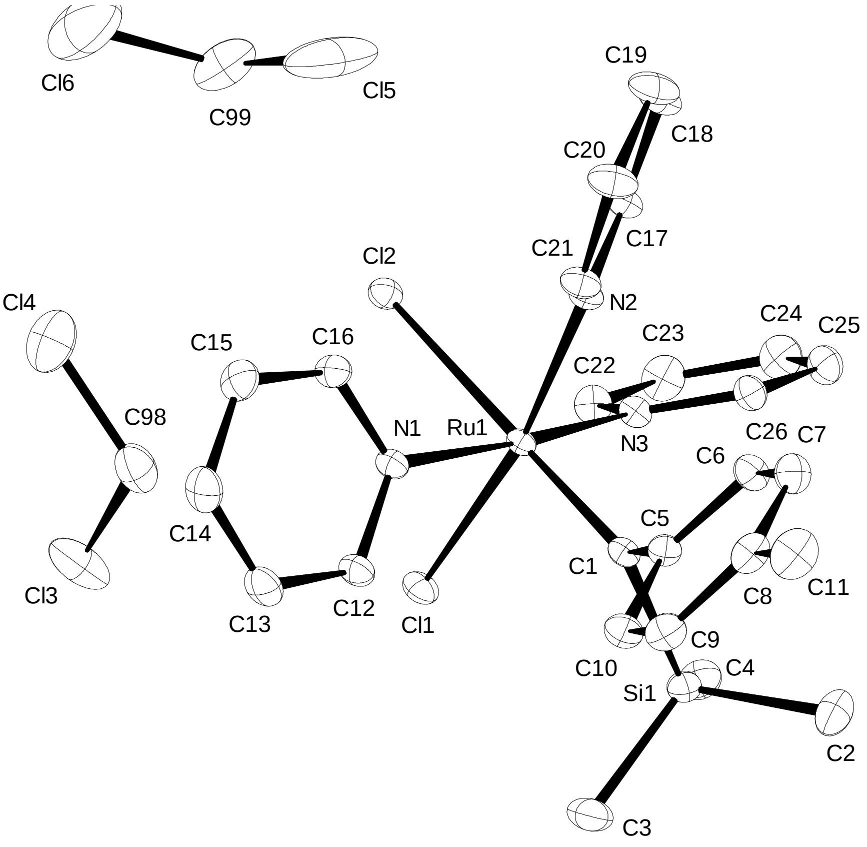

Figure S5. Crystallographic numbering scheme of Ru pyridine carbene complex *cis*-**39** (according to NMR, the complex having the two chloride atoms *cis* to each other is the minor isomer in solution). The thermal ellipsoid plot is shown at the 50% probability level, H-atoms omitted for clarity.

X-ray Crystal Structure Analysis of Complex *cis*-39: C_{28} H_{35} Cl_6 N_3 Ru Si, M_r = 755.45 g·mol^{-1}, green plate, crystal size 0.273 x 0.268 x 0.060 mm^3, triclinic, space group *P*-1 [2], a = 10.1000(2) Å, b = 13.5049(3) Å, c = 14.2875(3) Å, α = 112.7120(10)°, β = 92.2820(10)°, γ = 104.1340(10)°, V = 1723.57(6) Å^3, T = 100(2) K, Z = 2, D_{calc} = 1.456 g·cm^3, λ = 0.71073 Å, $\mu(Mo\text{-}K_\alpha)$ = 0.977 mm^{-1}, Gaussian absorption correction ($T_{\min}$ = 0.81, $T_{\max}$ = 0.94), Bruker AXS D8-Venture diffractometer with IμS Diamond Mo-anode X-ray source and PHOTON III detector, 2.103 < θ < 36.376°, 438396 measured reflections, 16739 independent reflections, 15470 reflections with $I > 2\sigma(I)$, R_{int} = 0.0691. The structure was solved by *SHELXT* and refined by full-matrix least-squares (*SHELXL*) against F^2 to R_1 = 0.0249 [$I > 2\sigma(I)$], wR_2 = 0.0646, 356 parameters. **CCDC-2515553**

Table S9. Crystallographic details for complex **39**

Identification code	15094	
Empirical formula	$C_{28} H_{35} Cl_6 N_3 Ru Si$	
Color	green	
Formula weight	755.45 g · mol^{-1}	
Temperature	100(2) K	
Wavelength	0.71073 Å	
Crystal system	TRICLINIC	
Space group	**P$\bar{1}$, (no. 2)**	
Unit cell dimensions	a = 10.1000(2) Å	α= 112.7120(10)°.
	b = 13.5049(3) Å	β= 92.2820(10)°.
	c = 14.2875(3) Å	γ = 104.1340(10)°.
Volume	1723.57(6) Å^3	
Z	2	
Density (calculated)	1.456 Mg · m^{-3}	
Absorption coefficient	0.977 mm^{-1}	
F(000)	768 e	
Crystal size	0.273 x 0.268 x 0.060 mm^3	
θ range for data collection	2.103 to 36.376°.	
Index ranges	$-16 \le h \le 16$, $-22 \le k \le 22$, $-23 \le l \le 23$	
Reflections collected	438396	
Independent reflections	16739 [R_{int} = 0.0691]	
Reflections with I>2σ(I)	15470	
Completeness to θ = 25.242°	100.0 %	
Absorption correction	Semi-empirical from equivalents	
Max. and min. transmission	0.94 and 0.81	
Refinement method	Full-matrix least-squares on F^2	
Data / restraints / parameters	16739 / 0 / 356	
Goodness-of-fit on F^2	1.027	
Final R indices [I>2σ(I)]	R_1 = 0.0249	wR^2 = 0.0625
R indices (all data)	R_1 = 0.0279	wR^2 = 0.0646
Largest diff. peak and hole	1.9 and -2.2 e · Å^{-3}	

Table S10. Bond lengths [Å] and angles [°] of complex **39**

Ru(1)-Cl(1)	2.4028(2)	Ru(1)-Cl(2)	2.5607(2)
Ru(1)-N(3)	2.1024(8)	Ru(1)-N(1)	2.1053(8)
Ru(1)-N(2)	2.1079(8)	Ru(1)-C(1)	1.8856(9)
Cl(3)-C(98)	1.7681(14)	Cl(4)-C(98)	1.7636(15)
Cl(5)-C(99)	1.756(2)	Cl(6)-C(99)	1.7531(19)
Si(1)-C(1)	1.9232(9)	Si(1)-C(4)	1.8702(11)
Si(1)-C(3)	1.8764(12)	Si(1)-C(2)	1.8806(12)
N(3)-C(22)	1.3478(12)	N(3)-C(26)	1.3505(12)
N(1)-C(16)	1.3472(12)	N(1)-C(12)	1.3514(12)
N(2)-C(21)	1.3519(12)	N(2)-C(17)	1.3521(12)
C(22)-C(23)	1.3904(15)	C(23)-C(24)	1.3840(17)
C(24)-C(25)	1.3899(16)	C(25)-C(26)	1.3824(13)
C(16)-C(15)	1.3867(14)	C(15)-C(14)	1.3861(16)
C(14)-C(13)	1.3881(17)	C(13)-C(12)	1.3882(14)
C(1)-C(5)	1.4876(13)	C(5)-C(10)	1.4034(13)
C(5)-C(6)	1.4033(13)	C(10)-C(9)	1.3920(14)
C(9)-C(8)	1.3946(16)	C(8)-C(7)	1.3956(15)
C(8)-C(11)	1.5065(15)	C(7)-C(6)	1.3920(14)
C(21)-C(20)	1.3838(14)	C(20)-C(19)	1.3860(16)
C(19)-C(18)	1.3874(16)	C(18)-C(17)	1.3859(14)
Cl(1)-Ru(1)-Cl(2)	86.842(8)	N(3)-Ru(1)-Cl(1)	90.69(2)
N(3)-Ru(1)-Cl(2)	86.84(2)	N(3)-Ru(1)-N(1)	173.45(3)
N(3)-Ru(1)-N(2)	90.68(3)	N(1)-Ru(1)-Cl(1)	87.01(2)
N(1)-Ru(1)-Cl(2)	86.90(2)	N(1)-Ru(1)-N(2)	90.54(3)
N(2)-Ru(1)-Cl(1)	170.03(2)	N(2)-Ru(1)-Cl(2)	83.37(2)
C(1)-Ru(1)-Cl(1)	92.66(3)	C(1)-Ru(1)-Cl(2)	178.12(3)
C(1)-Ru(1)-N(3)	91.35(3)	C(1)-Ru(1)-N(1)	94.88(3)
C(1)-Ru(1)-N(2)	97.18(3)	C(4)-Si(1)-C(1)	118.29(5)
C(4)-Si(1)-C(3)	108.90(6)	C(4)-Si(1)-C(2)	106.25(6)
C(3)-Si(1)-C(1)	108.00(5)	C(3)-Si(1)-C(2)	108.56(6)
C(2)-Si(1)-C(1)	106.49(5)	C(22)-N(3)-Ru(1)	119.80(6)
C(22)-N(3)-C(26)	117.72(8)	C(26)-N(3)-Ru(1)	122.39(6)
C(16)-N(1)-Ru(1)	119.96(6)	C(16)-N(1)-C(12)	117.48(8)
C(12)-N(1)-Ru(1)	122.20(6)	C(21)-N(2)-Ru(1)	123.14(6)
C(21)-N(2)-C(17)	117.08(8)	C(17)-N(2)-Ru(1)	119.42(6)
N(3)-C(22)-C(23)	122.42(10)	C(24)-C(23)-C(22)	119.29(10)
C(23)-C(24)-C(25)	118.66(9)	C(26)-C(25)-C(24)	118.88(10)
N(3)-C(26)-C(25)	123.02(9)	N(1)-C(16)-C(15)	122.97(9)
C(14)-C(15)-C(16)	119.06(10)	C(15)-C(14)-C(13)	118.67(9)
C(14)-C(13)-C(12)	118.98(10)	N(1)-C(12)-C(13)	122.82(9)
Ru(1)-C(1)-Si(1)	125.06(5)	C(5)-C(1)-Ru(1)	124.29(6)

C(5)-C(1)-Si(1)	110.57(6)	C(10)-C(5)-C(1)	121.46(8)
C(6)-C(5)-C(1)	121.30(8)	C(6)-C(5)-C(10)	117.11(9)
C(9)-C(10)-C(5)	121.18(9)	C(10)-C(9)-C(8)	121.50(9)
C(9)-C(8)-C(7)	117.53(9)	C(9)-C(8)-C(11)	121.08(10)
C(7)-C(8)-C(11)	121.40(10)	C(6)-C(7)-C(8)	121.33(10)
C(7)-C(6)-C(5)	121.32(9)	N(2)-C(21)-C(20)	122.91(9)
C(21)-C(20)-C(19)	119.41(10)	C(20)-C(19)-C(18)	118.40(9)
C(17)-C(18)-C(19)	119.05(9)	N(2)-C(17)-C(18)	123.12(9)
Cl(4)-C(98)-Cl(3)	111.40(7)	Cl(6)-C(99)-Cl(5)	113.06(11)

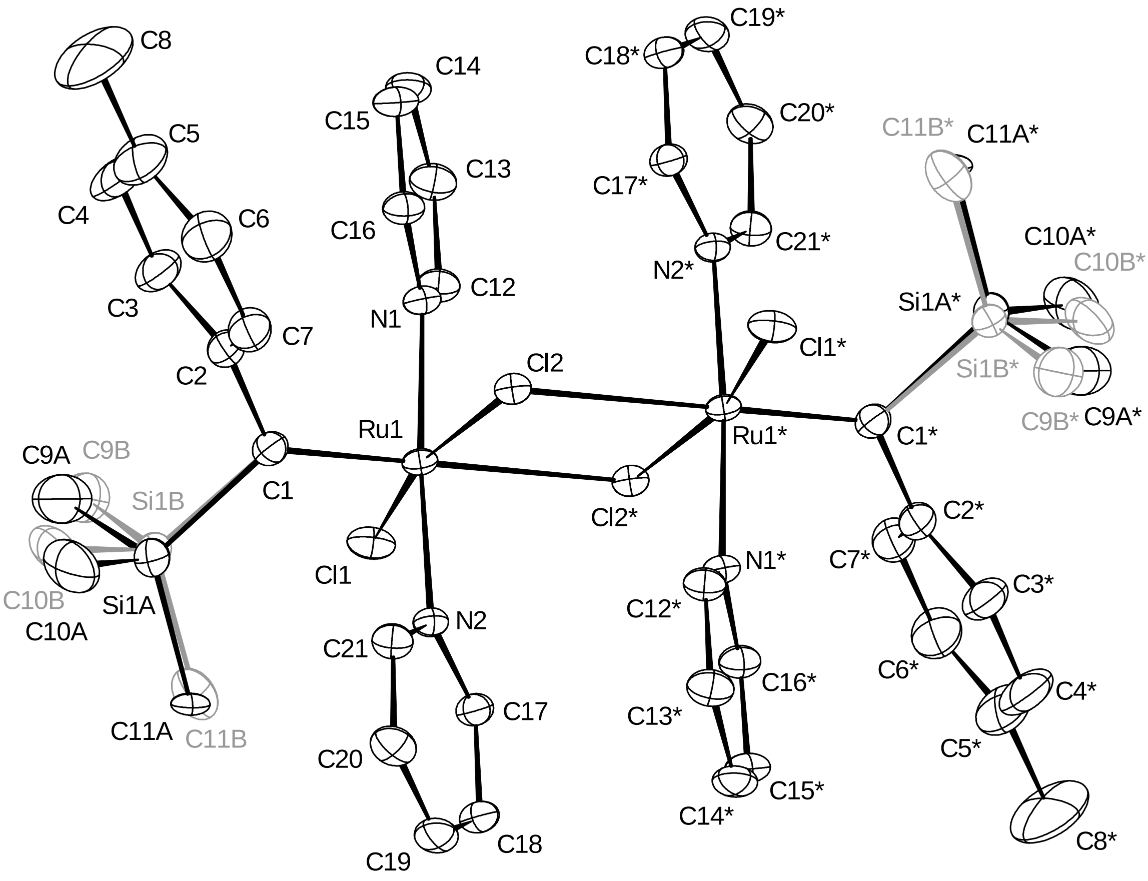

Figure S6. Crystallographic numbering scheme of Ru pyridine carbene complex **40**. The thermal ellipsoid plot is shown at the 50% probability level, H-atoms omitted for clarity.

X-ray Crystal Structure Analysis of Complex 40: C_{45} H_{57} Cl_4 N_4 Ru_2 Si_2, M_r = 1054.06 g·mol^{-1}, green plate, crystal size 0.22 x 0.081 x 0.041 mm^3, monoclinic, space group *C*2/*c* [15], a = 31.2427(13) Å, b = 9.0754(3) Å, c = 18.1996(7) Å, β = 102.918(2)°, V = 5029.7(3) Å^3, T = 100(2) K, Z = 4, D_{calc} = 1.392 g·cm^3, λ = 0.71073 Å, $\mu(Mo\text{-}K_\alpha)$ = 0.894 mm^{-1}, Gaussian absorption correction ($T_{\min}$ = 0.86, $T_{\max}$ = 0.98), Bruker AXS D8-Venture diffractometer with IµS Diamond Mo-anode X-ray source and PHOTON III detector, 2.342 < θ < 31.080°, 207189 measured reflections, 8052 independent reflections, 7056 reflections with $I > 2\sigma(I)$, R_{int} = 0.0985. The structure was solved by *SHELXT* and refined by full-matrix least-squares (*SHELXL*) against F^2 to R_1 = 0.0276 [$I > 2\sigma(I)$], wR_2 = 0.0745, 287 parameters, 48 restraints. **CCDC-2515552**

Table S11. Crystallographic details for complex **40**

Identification code	15707	
Empirical formula	$C_{45} H_{57} Cl_4 N_4 Ru_2 Si_2$	
Color	green	
Formula weight	1054.06 g·mol^{-1}	
Temperature	100(2) K	
Wavelength	0.71073 Å	
Crystal system	Monoclinic	
Space group	*C*2/*c*, (no. 15)	
Unit cell dimensions	a = 31.2427(13) Å	α= 90°.
	b = 9.0754(3) Å	β= 102.918(2)°.
	c = 18.1996(7) Å	γ = 90°.
Volume	5029.7(3) Å^3	
Z	4	
Density (calculated)	1.392 Mg·m^{-3}	
Absorption coefficient	0.894 mm^{-1}	
F(000)	2156 e	
Crystal size	0.22 x 0.081 x 0.041 mm^3	
θ range for data collection	2.342 to 31.080°.	
Index ranges	-45 ≤ h ≤ 45, -13≤ k ≤ 13, -25 ≤ l ≤ 26	
Reflections collected	207189	
Independent reflections	8052 [R_{int} = 0.0985]	
Reflections with I>2σ(I)	7056	
Completeness to θ = 25.242°	99.8 %	
Absorption correction	Numerical	
Max. and min. transmission	0.9757 and 0.8627	
Refinement method	Full-matrix least-squares on F^2	
Data / restraints / parameters	8052 / 48 / 287	
Goodness-of-fit on F^2	1.043	
Final R indices [I>2σ(I)]	R_1 = 0.0276	wR^2 = 0.0709
R indices (all data)	R_1 = 0.0335	wR^2 = 0.0745
Extinction coefficient	n/a	
Largest diff. peak and hole	0.603 and -0.483 e·Å^{-3}	

Table S12. Bond lengths [Å] and angles [°] of complex **40**

Ru(1)-Cl(1)	2.3953(4)	Ru(1)-Cl(2)	2.4105(4)
Ru(1)-Cl(2)#1	2.6530(4)	Ru(1)-N(1)	2.1159(14)
Ru(1)-N(2)	2.1011(13)	Ru(1)-C(1)	1.8678(17)
N(1)-C(12)	1.349(2)	N(1)-C(16)	1.349(2)
N(2)-C(17)	1.347(2)	N(2)-C(21)	1.349(2)
C(1)-C(2)	1.484(2)	C(1)-Si(1A)	1.940(10)
C(1)-Si(1B)	1.895(14)	C(2)-C(3)	1.396(3)
C(2)-C(7)	1.395(3)	C(3)-C(4)	1.384(3)
C(4)-C(5)	1.392(4)	C(5)-C(6)	1.376(4)
C(5)-C(8)	1.528(3)	C(6)-C(7)	1.395(3)
C(12)-C(13)	1.380(2)	C(13)-C(14)	1.387(3)
C(14)-C(15)	1.377(3)	C(15)-C(16)	1.386(2)
C(17)-C(18)	1.394(2)	C(18)-C(19)	1.383(3)
C(19)-C(20)	1.380(3)	C(20)-C(21)	1.385(2)
Si(1A)-C(9A)	1.856(12)	Si(1A)-C(10A)	1.918(11)
Si(1A)-C(11A)	1.868(14)	Si(1B)-C(9B)	1.913(17)
Si(1B)-C(10B)	1.813(16)	Si(1B)-C(11B)	1.82(2)
Cl(1)-Ru(1)-Cl(2)	166.630(16)	Cl(1)-Ru(1)-Cl(2)#1	85.768(14)
Cl(2)-Ru(1)-Cl(2)#1	80.909(13)	N(1)-Ru(1)-Cl(1)	90.99(4)
N(1)-Ru(1)-Cl(2)#1	86.76(4)	N(1)-Ru(1)-Cl(2)	89.37(4)
N(2)-Ru(1)-Cl(1)	90.26(4)	N(2)-Ru(1)-Cl(2)#1	89.31(4)
N(2)-Ru(1)-Cl(2)	88.49(4)	N(2)-Ru(1)-N(1)	175.78(5)
C(1)-Ru(1)-Cl(1)	92.92(6)	C(1)-Ru(1)-Cl(2)	100.41(5)
C(1)-Ru(1)-Cl(2)#1	178.55(6)	C(1)-Ru(1)-N(1)	92.65(7)
C(1)-Ru(1)-N(2)	91.30(7)	Ru(1)-Cl(2)-Ru(1)#1	99.093(13)
C(12)-N(1)-Ru(1)	121.14(11)	C(16)-N(1)-Ru(1)	121.46(11)
C(16)-N(1)-C(12)	117.37(14)	C(17)-N(2)-Ru(1)	121.95(11)
C(17)-N(2)-C(21)	117.42(14)	C(21)-N(2)-Ru(1)	120.63(11)
Ru(1)-C(1)-Si(1A)	126.9(3)	Ru(1)-C(1)-Si(1B)	125.0(3)
C(2)-C(1)-Ru(1)	122.86(13)	C(2)-C(1)-Si(1A)	110.2(3)
C(2)-C(1)-Si(1B)	111.7(4)	C(3)-C(2)-C(1)	121.54(18)
C(7)-C(2)-C(1)	120.82(18)	C(7)-C(2)-C(3)	117.64(18)
C(4)-C(3)-C(2)	120.9(2)	C(3)-C(4)-C(5)	121.1(2)
C(4)-C(5)-C(8)	120.9(3)	C(6)-C(5)-C(4)	118.4(2)
C(6)-C(5)-C(8)	120.7(3)	C(5)-C(6)-C(7)	120.9(2)
C(6)-C(7)-C(2)	121.0(2)	N(1)-C(12)-C(13)	122.74(16)
C(12)-C(13)-C(14)	119.32(17)	C(15)-C(14)-C(13)	118.42(16)
C(14)-C(15)-C(16)	119.29(16)	N(1)-C(16)-C(15)	122.73(16)
N(2)-C(17)-C(18)	122.47(16)	C(19)-C(18)-C(17)	119.16(17)
C(20)-C(19)-C(18)	118.78(16)	C(19)-C(20)-C(21)	118.96(17)
N(2)-C(21)-C(20)	123.16(16)	C(9A)-Si(1A)-C(1)	105.2(5)

C(9A)-Si(1A)-C(10A)	110.8(6)	C(9A)-Si(1A)-C(11A)	108.5(7)
C(10A)-Si(1A)-C(1)	106.5(5)	C(11A)-Si(1A)-C(1)	119.0(7)
C(11A)-Si(1A)-C(10A)	106.7(7)	C(1)-Si(1B)-C(9B)	109.1(7)
C(10B)-Si(1B)-C(1)	107.1(7)	C(10B)-Si(1B)-C(9B)	110.3(8)
C(10B)-Si(1B)-C(11B)	106.5(11)	C(11B)-Si(1B)-C(1)	116.4(11)
C(11B)-Si(1B)-C(9B)	107.4(11)		

Symmetry transformations used to generate equivalent atoms:
#1 -x+3/2,-y+3/2,-z+1

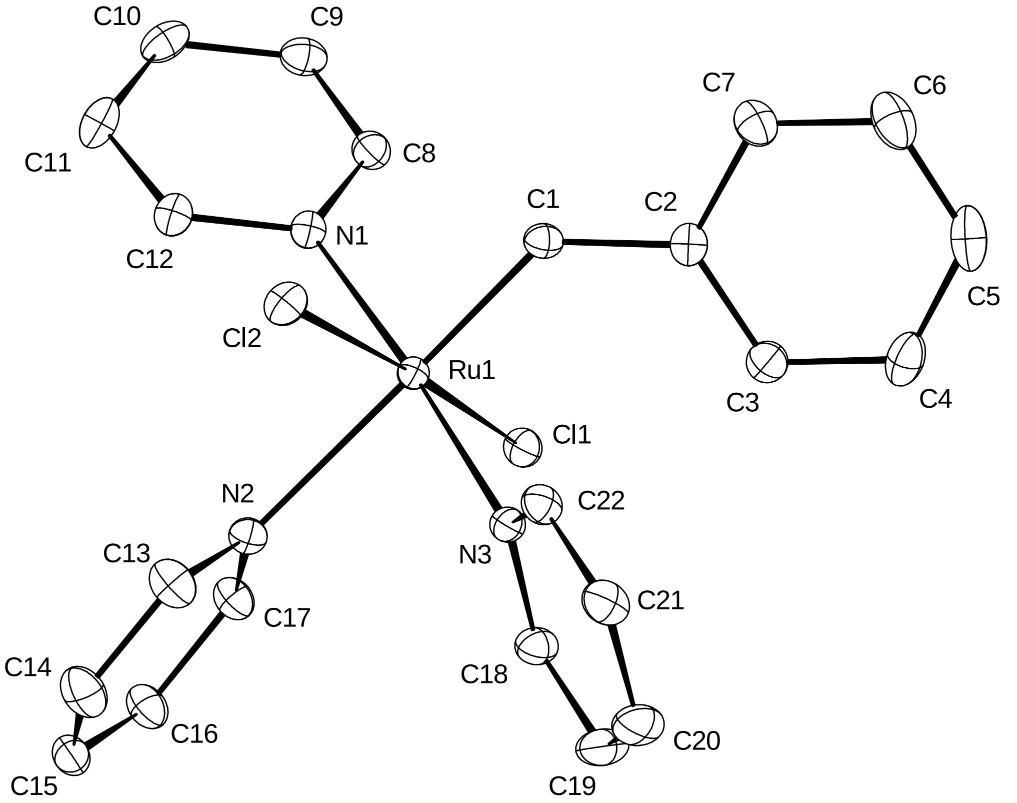

Figure S7. Crystallographic numbering scheme of Ru pyridine carbene complex **41**. The thermal ellipsoid plot is shown at the 50% probability level, H-atoms omitted for clarity.

X-ray Crystal Structure Analysis of Complex 41: C_{22} H_{20} Cl_2 N_3 Ru, M_r = 498.38 g·mol^{-1}, green plate, crystal size 0.442 x 0.263 x 0.02 mm^3, orthorhombic, space group $Pna2_1$ [33], a = 17.1766(9) Å, b = 9.0919(5) Å, c = 13.4778(7) Å, V = 2104.80(19) Å^3, T = 100(2) K, Z = 4, D_{calc} = 1.573 g·cm^3, λ = 0.71073 Å, $\mu(Mo\text{-}K_\alpha)$ = 1.011 mm^{-1}, Gaussian absorption correction ($T_{\min}$ = 0.82, $T_{\max}$ = 0.98), Bruker-AXS Kappa Mach3 with APEX-II detector and IµS microfocus source, 2.371 < θ < 31.534°, 58632 measured reflections, 6967 independent reflections, 6591 reflections with $I > 2\sigma(I)$, R_{int} = 0.0275. The structure was solved by *SHELXT* and refined by full-matrix least-squares (*SHELXL*) against F^2 to R_1 = 0.0196 [$I > 2\sigma(I)$], wR_2 = 0.0437, 253 parameters, 1 restraint, absolute structure parameter = -0.012(8). **CCDC-2515551**

Table S13. Crystallographic details for complex **41**

Identification code	15524	
Empirical formula	$C_{22} H_{20} Cl_2 N_3 Ru$	
Color	green	
Formula weight	498.38 g·mol^{-1}	
Temperature	100(2) K	
Wavelength	0.71073 Å	
Crystal system	Orthorhombic	
Space group	*Pna*2_1, (no. 33)	
Unit cell dimensions	a = 17.1766(9) Å	α= 90°.
	b = 9.0919(5) Å	β= 90°.
	c = 13.4778(7) Å	γ = 90°.
Volume	2104.80(19) Å^3	
Z	4	
Density (calculated)	1.573 Mg·m^{-3}	
Absorption coefficient	1.011 mm^{-1}	
F(000)	1004 e	
Crystal size	0.442 x 0.263 x 0.02 mm^3	
θ range for data collection	2.371 to 31.534°.	
Index ranges	-25 ≤ h ≤ 25, -13≤ k ≤ 13, -19 ≤ l ≤ 19	
Reflections collected	58632	
Independent reflections	6967 [R_{int} = 0.0275]	
Reflections with I>2σ(I)	6591	
Completeness to θ = 25.242°	100.0 %	
Absorption correction	Gaussian	
Max. and min. transmission	0.98011 and 0.82030	
Refinement method	Full-matrix least-squares on F^2	
Data / restraints / parameters	6967 / 1 / 253	
Goodness-of-fit on F^2	1.058	
Final R indices [I>2σ(I)]	R_1 = 0.0196	wR^2 = 0.0427
R indices (all data)	R_1 = 0.0224	wR^2 = 0.0437
Absolute structure parameter	-0.012(8)	
Extinction coefficient	n/a	
Largest diff. peak and hole	0.549 and -0.389 e·Å^{-3}	

Table S14. Bond lengths [Å] and angles [°] of complex **41**

Ru(1)-Cl(1)	2.3878(5)	Ru(1)-Cl(2)	2.3992(6)
Ru(1)-N(1)	2.0891(19)	Ru(1)-N(2)	2.3268(19)
Ru(1)-N(3)	2.0931(19)	Ru(1)-C(1)	1.868(2)
N(1)-C(8)	1.352(3)	N(1)-C(12)	1.350(3)
N(2)-C(13)	1.343(3)	N(2)-C(17)	1.347(3)
N(3)-C(18)	1.351(3)	N(3)-C(22)	1.350(3)
C(1)-C(2)	1.455(3)	C(2)-C(3)	1.402(3)
C(2)-C(7)	1.413(3)	C(3)-C(4)	1.392(3)
C(4)-C(5)	1.400(4)	C(5)-C(6)	1.385(4)
C(6)-C(7)	1.385(3)	C(8)-C(9)	1.381(3)
C(9)-C(10)	1.389(4)	C(10)-C(11)	1.384(4)
C(11)-C(12)	1.383(3)	C(13)-C(14)	1.391(3)
C(14)-C(15)	1.384(4)	C(15)-C(16)	1.384(3)
C(16)-C(17)	1.384(3)	C(18)-C(19)	1.382(3)
C(19)-C(20)	1.389(4)	C(20)-C(21)	1.377(4)
C(21)-C(22)	1.383(3)		
Cl(1)-Ru(1)-Cl(2)	175.292(19)	N(1)-Ru(1)-Cl(1)	89.11(5)
N(1)-Ru(1)-Cl(2)	88.72(5)	N(1)-Ru(1)-N(2)	92.02(7)
N(1)-Ru(1)-N(3)	176.42(7)	N(2)-Ru(1)-Cl(1)	88.34(5)
N(2)-Ru(1)-Cl(2)	87.56(5)	N(3)-Ru(1)-Cl(1)	91.25(6)
N(3)-Ru(1)-Cl(2)	90.65(6)	N(3)-Ru(1)-N(2)	84.43(7)
C(1)-Ru(1)-Cl(1)	92.95(7)	C(1)-Ru(1)-Cl(2)	91.15(7)
C(1)-Ru(1)-N(1)	87.99(8)	C(1)-Ru(1)-N(2)	178.71(9)
C(1)-Ru(1)-N(3)	95.55(9)	C(8)-N(1)-Ru(1)	121.95(15)
C(12)-N(1)-Ru(1)	120.64(15)	C(12)-N(1)-C(8)	117.4(2)
C(13)-N(2)-Ru(1)	120.74(16)	C(13)-N(2)-C(17)	116.6(2)
C(17)-N(2)-Ru(1)	122.19(15)	C(18)-N(3)-Ru(1)	121.03(15)
C(22)-N(3)-Ru(1)	121.28(16)	C(22)-N(3)-C(18)	117.7(2)
C(2)-C(1)-Ru(1)	133.89(17)	C(3)-C(2)-C(1)	123.4(2)
C(3)-C(2)-C(7)	117.9(2)	C(7)-C(2)-C(1)	118.6(2)
C(4)-C(3)-C(2)	120.5(2)	C(3)-C(4)-C(5)	120.5(2)
C(6)-C(5)-C(4)	119.6(2)	C(5)-C(6)-C(7)	120.1(2)
C(6)-C(7)-C(2)	121.3(2)	N(1)-C(8)-C(9)	123.0(2)
C(8)-C(9)-C(10)	118.9(2)	C(11)-C(10)-C(9)	118.6(2)
C(12)-C(11)-C(10)	119.3(2)	N(1)-C(12)-C(11)	122.7(2)
N(2)-C(13)-C(14)	123.3(2)	C(15)-C(14)-C(13)	119.2(2)
C(16)-C(15)-C(14)	118.0(2)	C(15)-C(16)-C(17)	119.3(2)
N(2)-C(17)-C(16)	123.5(2)	N(3)-C(18)-C(19)	122.6(2)
C(18)-C(19)-C(20)	119.1(2)	C(21)-C(20)-C(19)	118.5(2)
C(20)-C(21)-C(22)	119.6(2)	N(3)-C(22)-C(21)	122.4(2)

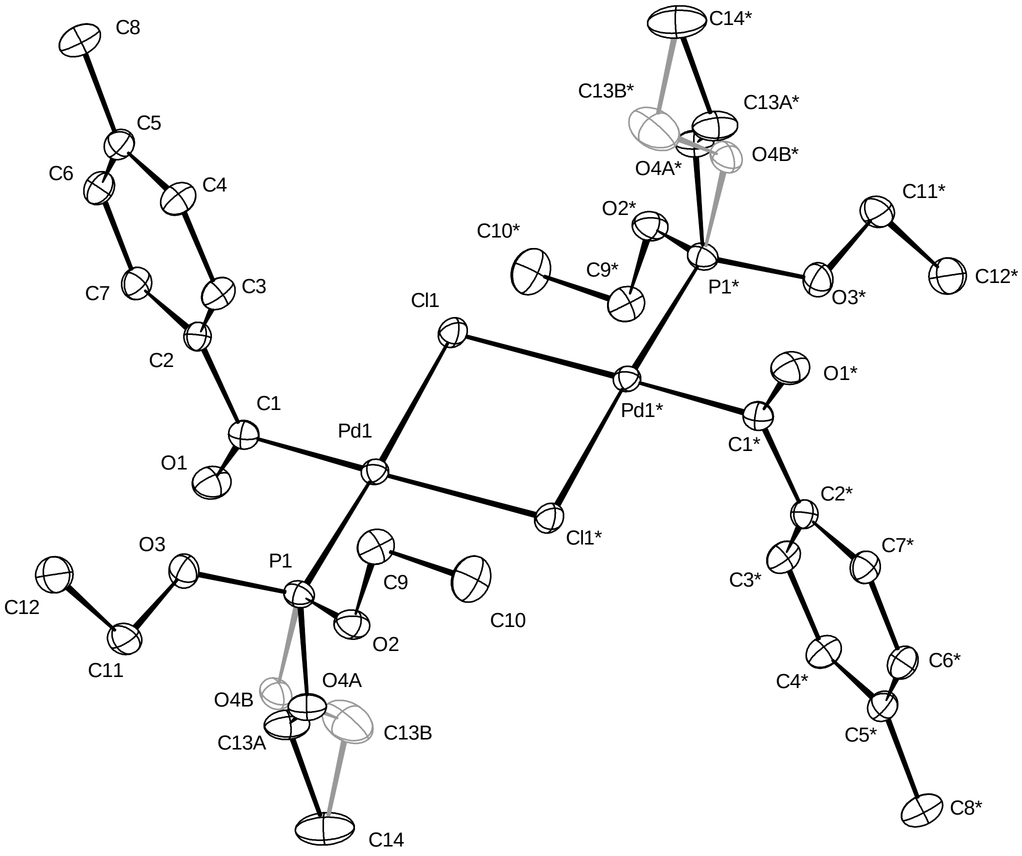

Figure S8. Crystallographic numbering scheme of the acyl palladium complex **S2**. The thermal ellipsoid plot is shown at the 50% probability level, H-atoms omitted for clarity.

X-ray Crystal Structure Analysis of Complex S2: $C_{28}H_{44}Cl_2O_8P_2Pd_2$, M_r = 854.27 g · mol^{-1}, yellow prism, crystal size 0.203 x 0.14 x 0.091 mm^3, monoclinic, space group $P2_1/c$ [14], a = 11.5236(8) Å, b = 15.7248(11) Å, c = 9.3527(6) Å, β = 91.143(2)°, V = 1694.4(2) Å^3, T = 100(2) K, Z = 2, D_{calc} = 1.674 g · cm^3, λ = 0.71073 Å, $\mu(Mo\text{-}K_\alpha)$ = 1.358 mm^{-1}, analytical absorption correction (T_{min} = 0.81, T_{max} = 0.90), Bruker-AXS Kappa Mach3 diffractometer with APEX-II detector and IμS micro focus X-ray source, 1.768 < θ < 33.692°, 112779 measured reflections, 6734 independent reflections, 6412 reflections with $I > 2\sigma(I)$, R_{int} = 0.0172, 212 parameters, S = 1.085, residual electron density +0.5 (0.70 Å from C7) / -0.5 (0.64 Å from Pd1) e · Å^{-3}. The structure was solved by *SHELXT* and refined by full-matrix least-squares (*SHELXL*) against F^2 to R_1 = 0.014 [$I > 2\sigma(I)$], wR_2 = 0.038. **CCDC-2431911**

Table S15. Crystallographic details for complex **S2**

Identification code	15072sadabs	
Empirical formula	$C_{28} H_{44} Cl_2 O_8 P_2 Pd_2$	
Color	yellow	
Formula weight	854.27 g·mol^{-1}	
Temperature	100(2) K	
Wavelength	0.71073 Å	
Crystal system	Monoclinic	
Space group	$P2_1$/c, (no. 14)	
Unit cell dimensions	a = 11.5236(8) Å	α= 90°.
	b = 15.7248(11) Å	β= 91.143(2)°.
	c = 9.3527(6) Å	γ = 90°.
Volume	1694.4(2) Å^3	
Z	2	
Density (calculated)	1.674 Mg·m^{-3}	
Absorption coefficient	1.358 mm^{-1}	
F(000)	864 e	
Crystal size	0.203 x 0.14 x 0.091 mm^3	
θ range for data collection	1.768 to 33.692°.	
Index ranges	-17 ≤ h ≤ 17, -24≤ k ≤ 24, -14 ≤ l ≤ 14	
Reflections collected	112779	
Independent reflections	6734 [R_{int} = 0.0172]	
Reflections with I>2σ(I)	6412	
Completeness to θ = 25.242°	100.0 %	
Absorption correction	Gaussian	
Max. and min. transmission	0.90463 and 0.80688	
Refinement method	Full-matrix least-squares on F^2	
Data / restraints / parameters	6734 / 0 / 212	
Goodness-of-fit on F^2	1.085	
Final R indices [I>2σ(I)]	R_1 = 0.0142	wR^2 = 0.0367
R indices (all data)	R_1 = 0.0155	wR^2 = 0.0379
Extinction coefficient	n/a	
Largest diff. peak and hole	0.530 and -0.475 e·Å^{-3}	

Table S16. Bond lengths [Å] and angles [°] of complex **S2**

Pd(1)-Cl(1)#1	2.4801(2)	Pd(1)-Cl(1)	2.4050(2)
Pd(1)-P(1)	2.2093(2)	Pd(1)-C(1)	1.9763(8)
P(1)-O(2)	1.5819(7)	P(1)-O(3)	1.5774(7)
P(1)-O(4A)	1.5952(13)	P(1)-O(4B)	1.569(5)
O(1)-C(1)	1.2089(10)	O(2)-C(9)	1.4550(11)
O(3)-C(11)	1.4638(11)	O(4A)-C(13A)	1.4449(16)
O(4B)-C(13B)	1.456(8)	C(1)-C(2)	1.4905(11)
C(2)-C(3)	1.3896(11)	C(2)-C(7)	1.3960(11)
C(3)-C(4)	1.3893(12)	C(4)-C(5)	1.3933(12)
C(5)-C(6)	1.3948(12)	C(5)-C(8)	1.5011(13)
C(6)-C(7)	1.3829(12)	C(9)-C(10)	1.4991(14)
C(11)-C(12)	1.4940(13)	C(13A)-C(14)	1.4887(16)
C(13B)-C(14)	1.503(6)		
Cl(1)-Pd(1)-Cl(1)#1	86.573(8)	P(1)-Pd(1)-Cl(1)#1	94.780(9)
P(1)-Pd(1)-Cl(1)	177.060(8)	C(1)-Pd(1)-Cl(1)	88.43(2)
C(1)-Pd(1)-Cl(1)#1	172.01(2)	C(1)-Pd(1)-P(1)	90.50(2)
Pd(1)-Cl(1)-Pd(1)#1	93.427(8)	O(2)-P(1)-Pd(1)	115.72(3)
O(2)-P(1)-O(4A)	93.07(5)	O(3)-P(1)-Pd(1)	113.66(3)
O(3)-P(1)-O(2)	106.40(4)	O(3)-P(1)-O(4A)	108.75(5)
O(4A)-P(1)-Pd(1)	117.08(5)	O(4B)-P(1)-Pd(1)	115.07(19)
O(4B)-P(1)-O(2)	108.96(19)	O(4B)-P(1)-O(3)	94.84(17)
C(9)-O(2)-P(1)	120.29(6)	C(11)-O(3)-P(1)	121.13(6)
C(13A)-O(4A)-P(1)	119.47(9)	C(13B)-O(4B)-P(1)	117.2(4)
O(1)-C(1)-Pd(1)	120.37(6)	O(1)-C(1)-C(2)	122.41(8)
C(2)-C(1)-Pd(1)	116.42(6)	C(3)-C(2)-C(1)	122.01(7)
C(3)-C(2)-C(7)	119.40(7)	C(7)-C(2)-C(1)	118.46(7)
C(4)-C(3)-C(2)	120.05(8)	C(3)-C(4)-C(5)	120.83(8)
C(4)-C(5)-C(6)	118.67(8)	C(4)-C(5)-C(8)	120.57(8)
C(6)-C(5)-C(8)	120.74(8)	C(7)-C(6)-C(5)	120.76(8)
C(6)-C(7)-C(2)	120.24(8)	O(2)-C(9)-C(10)	107.68(8)
O(3)-C(11)-C(12)	107.94(7)	O(4A)-C(13A)-C(14)	107.20(10)
O(4B)-C(13B)-C(14)	108.8(5)		

Symmetry transformations used to generate equivalent atoms:
#1 -x+1,-y+1,-z+1

Computational Study

All electronic structure calculations were performed using the ORCA 6.0 program package.[23] Geometry optimizations were conducted at the B3LYP-D4/def2-tzvp CPCM(toluene) level of theory[24] using tight convergence and large grid criteria. Minima were confirmed via normal mode analysis. Free energy corrections at 298.15 K have been computed using the standard harmonic oscillator approximation as implemented in the thermochemistry module of ORCA.[25]

Sample input parameters for geometry optimization and frequency calculation:

```
!B3LYP D4 def2-tzvp CPCM(toluene) TightOpt TightSCF DefGrid3 Freq
```

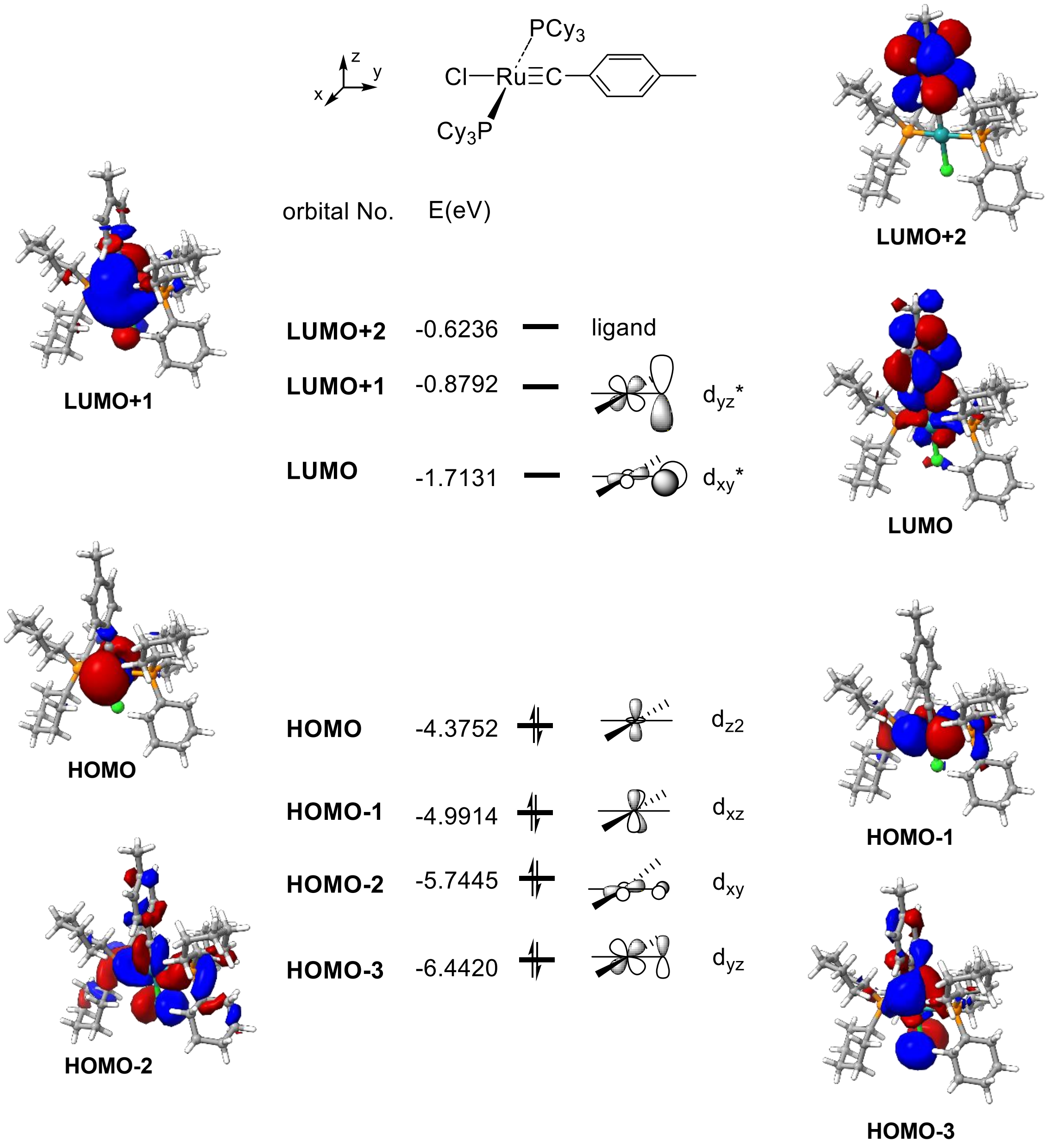

Figure S9. Frontier MO diagram of the Ru alkylidyne complex **9**

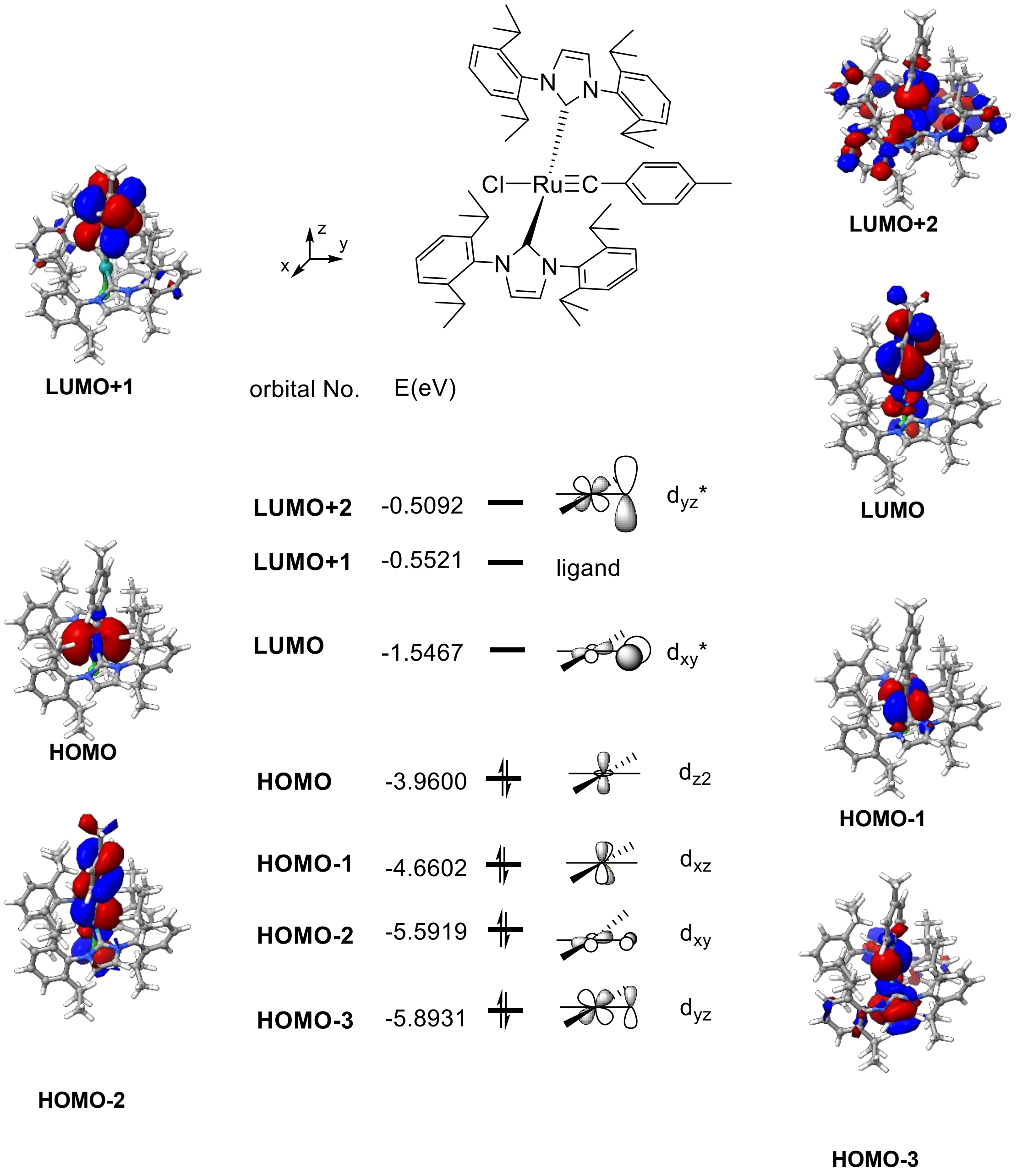

Figure S10. Frontier MO diagram of the Ru alkylidyne complex **26**

xyz Coordinates of Computed Structures

Alkylidyne 9

E -2958.18066947 Eh G = -2957.16457836 Eh

```
121
  Ru     4.04152700822292    5.02054049154079    12.54333941854921
  Cl     1.99312499437407    4.35480705319567    11.32417706485742
  P      4.13200363528077    2.84150979968990    13.53567899075629
  P      4.10771381630471    7.02751127718155    11.23368615596805
  C      5.44731024137850    5.47866029934921    13.37113689254496
  C      6.63608097948606    5.86522331411674    14.06167620585261
  C      7.89885446165674    5.58585141032273    13.51965948113783
  H      7.96286861529264    5.07241421700321    12.56991808303803
  C      9.04744783828852    5.96676096903550    14.19206337626240
  H     10.01712235129706    5.74794272627462    13.76048474615149
  C      8.97818836710722    6.63201258898787    15.41928148133369
  C      7.71673996613464    6.91233712837984    15.95241373080925
  H      7.64323476359997    7.43479296762832    16.89905770164195
  C      6.56219047078590    6.53716935116444    15.28987595180224
  H      5.59098282754246    6.76199861602342    15.70927746645657
  C     10.22664793096882    7.01590600202468    16.16159766874545
  H     11.08315393235156    7.08536081927816    15.49045594167903
  H     10.46497729318669    6.26916069557409    16.92521531579398
  H     10.10587493736712    7.97279922193218    16.67195634510693
  C      2.49206660841464    1.99266817588565    13.70242319615040
  H      2.11370527552706    2.09600910276335    12.68056793407819
  C      1.50845859053762    2.76760658778561    14.59084215760656
  H      1.80436343112965    2.67681586001449    15.63958018183775
  H      1.53407665393859    3.82855979668204    14.33770536482818
  C      0.09078938275302    2.21523032403045    14.42512685795567
  H     -0.59910185764091    2.75708349784854    15.07837227407384
  H     -0.23984755980956    2.39573193396013    13.39663774413258
  C      0.03599608319522    0.71589067459472    14.72606405869980
  H     -0.97196127009116    0.32969524093453    14.55034166863337
  H      0.25264428451515    0.55562222462416    15.78923399616542
  C      1.05285054541253   -0.06097426251094    13.88717328293315
  H      0.76978090500036   -0.00266998625106    12.83001856920634
  H      1.03863966692981   -1.12032652260730    14.15960817799129
  C      2.46958706271067    0.50021512615288    14.05422693411038
  H      3.16273697209145   -0.06365833699464    13.42750130888423
  H      2.78769940505783    0.35361646715608    15.08921799050162
  C      5.06513972887119    2.78720551668530    15.14529837124294
  H      6.01587468417731    3.24753190658470    14.85533658044162
  C      4.46241435496224    3.69159830241869    16.22889776261655
  H      4.22316327168399    4.66934286377457    15.80788476520241
  H      3.52121709756631    3.26456000165510    16.58373234327671
  C      5.42050851518577    3.82742443797593    17.41503738417609
  H      6.33181832288852    4.33650469980105    17.08453418234498
  H      4.97009582348920    4.45841092132871    18.18671870273768
  C      5.79023345990623    2.46101916130769    17.99517205930949
  H      6.51181743172098    2.57640850806981    18.80890181037119
  H      4.89601092735702    2.00085170103925    18.43221741337225
  C      6.35283058755279    1.53537443604700    16.91534851503881
  H      6.56018663981192    0.54612612357404    17.33318325130962
  H      7.31022616701734    1.93392305290089    16.55958350526110
  C      5.39245466983846    1.40670223117626    15.72821758606467
  H      4.47382761350571    0.92427858216327    16.06923415492772
  H      5.82918867235476    0.75447699605645    14.97031746653152
```

C	5.10826430774157	1.68273917239524	12.45497908687187
H	5.11575562839143	0.70204220770096	12.94104160657806
C	4.45048127462880	1.52918661738133	11.07520889486044
H	3.43083832134140	1.15364331289707	11.17470249879720
H	4.36949518255804	2.51620361182263	10.61158815984122
C	5.25612418113566	0.59896819658058	10.16616354905142
H	5.24890473183426	-0.41488822710572	10.58405954513534
H	4.77457085901884	0.53761663535584	9.18627746505826
C	6.70214744977793	1.07165401180775	10.01924969978632
H	7.27055953688928	0.37530385667732	9.39639110493663
H	6.71358277512665	2.03869276561314	9.50337224313260
C	7.36611064157297	1.22710564491868	11.38709995320155
H	8.38327757595772	1.61401621224259	11.27533657743488
H	7.45595924806393	0.24159168922634	11.85934751820408
C	6.56148449365913	2.15481935973412	12.29995719243104
H	6.55019776501677	3.16519141292025	11.88171406439161
H	7.05263803424566	2.22245183333255	13.27255903896121
C	2.44721384052458	7.67849447088535	10.72913408550347
H	2.04860822762921	6.80897413882095	10.19739887097070
C	1.49831596154576	7.90597329141784	11.91513458480786
H	1.54248905303191	7.05497529585188	12.59591681810201
H	1.80823440817992	8.78837383826164	12.48120739280951
C	0.06682891064288	8.11196527295270	11.41451903942915
H	-0.60200453629376	8.29275287507546	12.26081005239195
H	-0.27422535511816	7.18857271900045	10.93389089682395
C	-0.01809978643170	9.26903385764275	10.41665465262361
H	-1.03686327115803	9.36369961947408	10.03033627603993
H	0.20814595537220	10.20818018292953	10.93621299044177
C	0.96891299421359	9.08767823960203	9.26130086655504
H	0.93699573481870	9.95525900555149	8.59571164597169
H	0.67074583994399	8.21958590420981	8.66234145117874
C	2.39904195136822	8.87261972185809	9.76889201745636
H	2.73065650717461	9.77767951411173	10.28463988063381
H	3.07293808959646	8.72546296216033	8.92245906875979
C	5.13626689036554	8.38289087735638	11.99079067367205
H	6.04993272161996	7.83921434751850	12.25274230348242
C	4.54586672869296	8.90514958883477	13.30737459415288
H	3.64300996163910	9.48466118757411	13.09810902520867
H	4.24294722174548	8.06830361116030	13.93933476254036
C	5.54495646370393	9.80537020434484	14.03773639710824
H	5.09611264502521	10.18486299170891	14.96025056552671
H	6.41659247924830	9.21254494864141	14.33315182544949
C	5.99809474566104	10.96579719722401	13.15080459790129
H	5.14214220834817	11.62106777753765	12.95026263752020
H	6.74420135652685	11.57378012144424	13.67011313642785
C	6.55989056581675	10.45723790423152	11.82271995917881
H	7.47902121494200	9.89050328676306	12.01264783996191
H	6.83608100841392	11.29755933046420	11.17952113177758
C	5.55987270180714	9.55334073786150	11.09292699455555
H	6.00981858149115	9.19120519769972	10.16757005442101
H	4.68666547187687	10.14413034351989	10.80865240606648
C	4.96903587750135	6.72413767486528	9.61142160828220
H	5.01947964724059	7.68112835772478	9.08217044090009
C	4.20178207666797	5.73432577703998	8.72338587280796
H	4.07437676322488	4.79328491894582	9.26392383998891
H	3.19709645005754	6.10509616168488	8.51469867812673
C	4.93661904480787	5.47877563635047	7.40563627205487
H	4.37912492404098	4.74962193040952	6.81113771065255
H	4.96527906045467	6.40557680081017	6.82000711040222
C	6.36577316037670	4.99072502795865	7.64114482841612

H	6.88331965349387	4.85120169557881	6.68790691380624
H	6.33474645501019	4.01003606811395	8.12980132936247
C	7.13729308178333	5.96770206622876	8.52816991352193
H	7.26879545353174	6.91568395562023	7.99292203849144
H	8.14051224384491	5.58357286520784	8.73499381577729
C	6.40390267282548	6.22605570842419	9.84543532353958
H	6.96900620501990	6.94148060049301	10.44644385720781
H	6.35352515300514	5.30154658202142	10.42787618203533

Alkylidyne 26

E -3183.88281394 Eh G = -3182.70870655 Eh

147

Ru	7.24092379237467	3.68231938265828	13.94251912965474
Cl	9.55513514273996	3.37541198875333	13.16680202045816
N	6.07118751996738	3.13906619519740	11.06562263289150
N	7.01673963548970	5.06046558707006	11.13348795406914
N	8.18929547950354	4.26163278100038	16.89097474358118
N	8.43315249496122	2.18434576622118	16.42644366214283
C	5.63769870673179	3.89101544700459	14.48136256843580
C	4.29096397347806	4.06081014260700	14.93556274000643
C	3.40491179644279	4.92070358921479	14.26912341784348
H	3.74142834915830	5.45291942668120	13.39258182399054
C	2.10619467196755	5.08421535806785	14.71589998494120
H	1.44278948277121	5.75647353865255	14.18364515120574
C	1.63197475084760	4.39715419979452	15.83493317715988
C	2.51402930240326	3.54354680130343	16.49884696030207
H	2.17518280417600	3.00786939222371	17.37826672103557
C	3.81748440761835	3.37672845886928	16.06476642717208
H	4.48396740025160	2.72464462002253	16.60840696644768
C	0.20922540436941	4.55441558845815	16.29237199830400
H	-0.15297752875738	5.56902381091263	16.11855213660704
H	0.10556257762289	4.32775707569971	17.35428148928399
H	-0.45228145685008	3.87489269881951	15.74583504575325
C	6.75936948198796	3.96499942400229	11.90615666354050
C	5.89158623102601	3.71747293016235	9.81792777658828
H	5.38514196738627	3.20846472384486	9.01989731578586
C	6.47878521314874	4.92567149223594	9.86373141775533
H	6.56640416085603	5.69659869842483	9.12178380491411
C	5.73016261747785	1.76031819070162	11.29510321086755
C	4.39359457291546	1.41252428251116	11.52392405166834
C	4.08338069262930	0.05579085191446	11.60946074409562
H	3.06005037063612	-0.24687827756348	11.78325993852461
C	5.06670543833504	-0.91153002428833	11.49447652453478
H	4.80680225455186	-1.95962450917650	11.57962896517376
C	6.38333406902621	-0.54051484487905	11.27231637715773
H	7.14249488529017	-1.30444524787145	11.18033109123492
C	6.74207894129339	0.79895943532571	11.14436376006378
C	3.29058799642292	2.44780081784010	11.63780798042684
H	3.75514591295775	3.40262893190957	11.88565535548145
C	2.28676890052208	2.11667610404197	12.74695821160909
H	1.58039603008982	2.93869136501920	12.86358320972956
H	2.78304355845908	1.96270479610435	13.70346498463143
H	1.71034899543527	1.22002246782013	12.51090560116278
C	2.54865000671073	2.61240954605525	10.30180638264763
H	2.05675238900602	1.67809224015333	10.02010047184962
H	3.22069080293472	2.89304066899765	9.49139065423946
H	1.78143348468737	3.38578583473498	10.38677745800242

C	8.15932243763595	1.17981856511005	10.76101614627095
H	8.35261140136645	2.18205947786357	11.13417590978051
C	8.31236546798604	1.19905124183603	9.23151639980271
H	8.15995197775069	0.20021790851882	8.81371616426610
H	9.31778197777539	1.53039272540802	8.96084801941397
H	7.59874925293566	1.87451127504282	8.75885455485211
C	9.21918941049126	0.27805992211354	11.39229455215460
H	9.10338369075483	0.23084603307948	12.47233034321205
H	10.21127828416523	0.68134393885357	11.18197857011354
H	9.18577849013405	-0.73940921905955	10.99490145071331
C	7.81802087153797	6.20357737908058	11.48299533451670
C	7.24967109493346	7.22025978745643	12.25947302945133
C	8.02301091753430	8.34711947626913	12.52326445328474
H	7.61858747174558	9.14217082650441	13.13371462776017
C	9.30169534126840	8.47038308639358	12.00289822074669
H	9.88905920971593	9.35549910586815	12.21459059267950
C	9.83222501069462	7.46107776510078	11.21732131499154
H	10.83242923446659	7.56881056877687	10.81985317007075
C	9.10835736426372	6.30151882049758	10.94396208447514
C	5.81176453621132	7.13449686047525	12.72429108370538
H	5.58445684043681	6.07719182651536	12.83092522026388
C	5.57840153695220	7.78019785606013	14.08751293451077
H	5.72349943368028	8.86240227201087	14.06112576082464
H	6.24926693315779	7.36355937630526	14.83527409996526
H	4.55330992635597	7.59859041478894	14.41470254565141
C	4.87083668125055	7.72706208149713	11.66467601937057
H	3.82868003056085	7.61825113467941	11.97655384805190
H	4.98724168448388	7.23074193665897	10.70010924944769
H	5.07014477157035	8.79231384498135	11.52228197278047
C	9.71380516063076	5.23111841770387	10.05290672175523
H	9.13439222932831	4.31811210279803	10.18248622588304
C	11.16470007314486	4.90184570359701	10.42278567686736
H	11.83402848200456	5.73867782959438	10.21162364732097
H	11.50847777549451	4.05104431343768	9.82992370581437
H	11.24819537433534	4.63798273882770	11.47478881109474
C	9.63803597707310	5.64561971393723	8.57435426123758
H	8.61585973231054	5.84699070386396	8.25381481245294
H	10.04055879487731	4.85321146364262	7.93892598283203
H	10.22671175008408	6.54950301039826	8.39920050775046
C	8.02388308070193	3.36479902169277	15.87629250870135
C	8.66458722260132	3.64821571579173	18.04045487741357
H	8.86874570069934	4.19855739392775	18.93928637899869
C	8.81004306757383	2.34377521964119	17.75023352750413
H	9.14360014472825	1.51924346828794	18.35121249572232
C	8.11105560731563	5.69534362616858	16.80085973453559
C	7.02397420267282	6.36091189491916	17.37937652841440
C	7.05107929555302	7.75479994017581	17.39207471075299
H	6.22824374119271	8.29994414665467	17.83334042752388
C	8.10682176662690	8.45501146031136	16.83415039508702
H	8.10216897954972	9.53817353066956	16.84326787167553
C	9.16860970692606	7.77196289340450	16.26299063289197
H	9.98979793483230	8.32923618836725	15.83455969467932
C	9.20629884380584	6.37989684859212	16.25134739225475
C	5.86143098595099	5.62069909736221	18.01292368958416
H	5.85395739194226	4.60788875878234	17.60910947871095
C	4.51040809555178	6.26462865379775	17.68557239043238
H	4.41531176122596	7.25004906116998	18.14548649164482
H	3.70077449845723	5.64456319372614	18.07051981910088
H	4.36999297017561	6.37321294564846	16.61168039448156
C	6.03412837237087	5.52286917064461	19.53681129858390

H	6.96574401657660	5.02964966972120	19.81316128506059
H	5.20943036948171	4.95640796065901	19.97604614880196
H	6.03654301533909	6.51883853013487	19.98690737483021
C	10.43541743052245	5.65193574325495	15.74249901137544
H	10.13178612142288	4.66073694724646	15.41643834955377
C	11.07165792720090	6.32254394614858	14.52562553211016
H	11.51801991549932	7.28893214567183	14.77281137214728
H	10.34250371051460	6.47176354723214	13.73294429178078
H	11.86468348934513	5.68400933318738	14.13255049679173
C	11.46716840444120	5.49013876063437	16.87045114965760
H	12.33008821358993	4.92561659448873	16.50904038157554
H	11.05311040933960	4.95739491803692	17.72718762932654
H	11.82045896756769	6.46524855677595	17.21652424066926
C	8.58465028993996	0.91785351073742	15.75986753750438
C	7.45493754584330	0.11833697499874	15.55368367267794
C	7.64056935612392	-1.13551664305482	14.97775020088491
H	6.78464693512621	-1.76974331419146	14.79472368379715
C	8.90882661974103	-1.58739633124501	14.64926019793993
H	9.03621055576630	-2.56721066440060	14.20547323888565
C	10.01327399317620	-0.78539774728109	14.88269528241661
H	10.99783373168875	-1.14822147845891	14.62025463315191
C	9.87896027214963	0.48721573851492	15.43563212649802
C	6.08596160992792	0.56472492837504	16.01934709036845
H	6.08923717683766	1.65109420639981	15.99561367847765
C	4.95959819473304	0.10536468164870	15.09778898440114
H	5.14504154947327	0.42186817401062	14.07400139392060
H	4.01260380443011	0.54123263707402	15.42030578604454
H	4.83649063804258	-0.97978361922256	15.10382844423401
C	5.83981343786234	0.11267862877111	17.46626927048903
H	4.86920225361439	0.47156248117869	17.81918349700919
H	6.60619959533646	0.49486106534228	18.14252647837733
H	5.84053686234847	-0.97808483307429	17.53716119977433
C	11.11934810917578	1.32127203279329	15.70400861100634
H	10.80421439536999	2.35022173643560	15.87154263704456
C	12.09131705235702	1.33475203497378	14.51853783702898
H	12.54412938417191	0.35414003496006	14.35679839878821
H	12.90201777172389	2.03951713791828	14.71797627262835
H	11.58750551395029	1.64674125141688	13.60614249959103
C	11.84594219918059	0.82683243103506	16.96535148336552
H	11.20057031826346	0.83901923923274	17.84366971070067
H	12.71249079456454	1.45848087841612	17.17465788782229
H	12.20101036562821	-0.19730808997501	16.82644353368037

NMR and IR Spectra

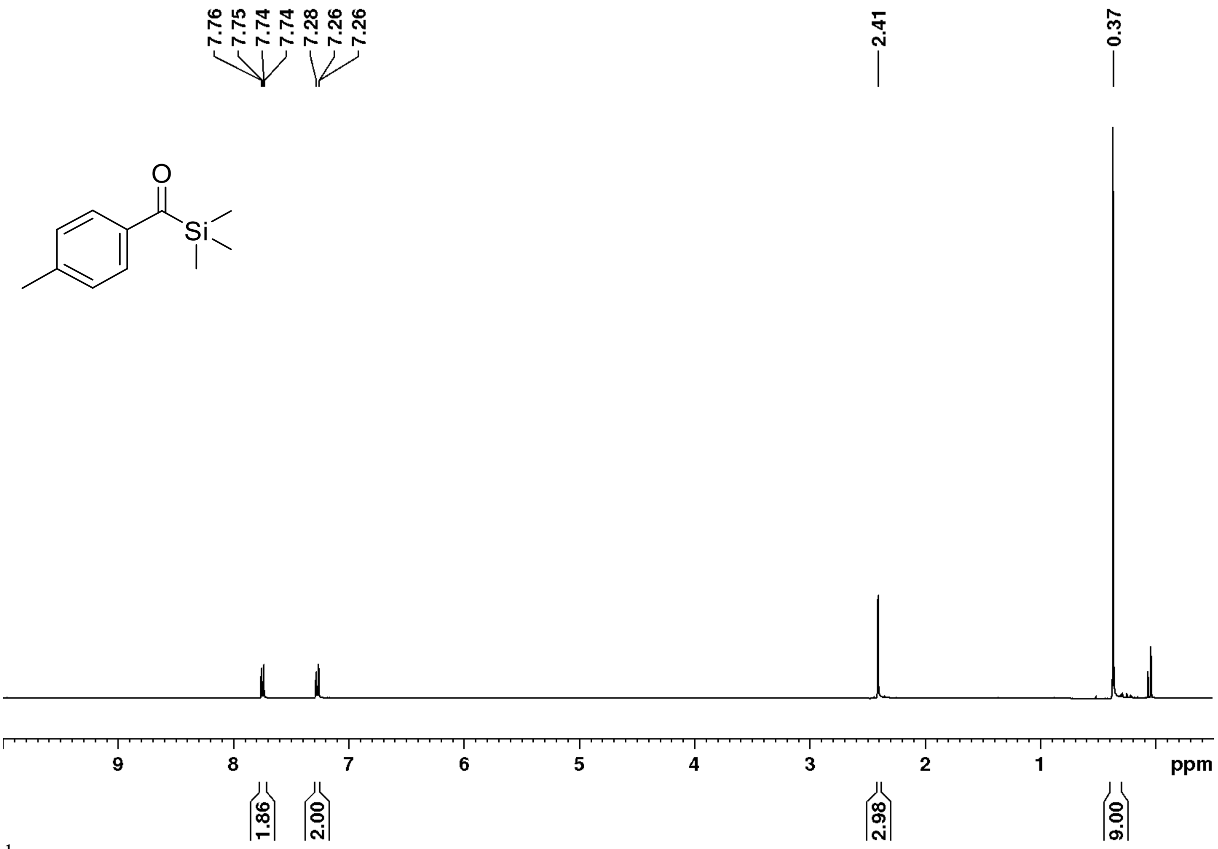

^{1}H NMR spectrum of *p*-tolyl(trimethylsilyl)methanone (**S1**) in $CDCl_3$ at 400 MHz

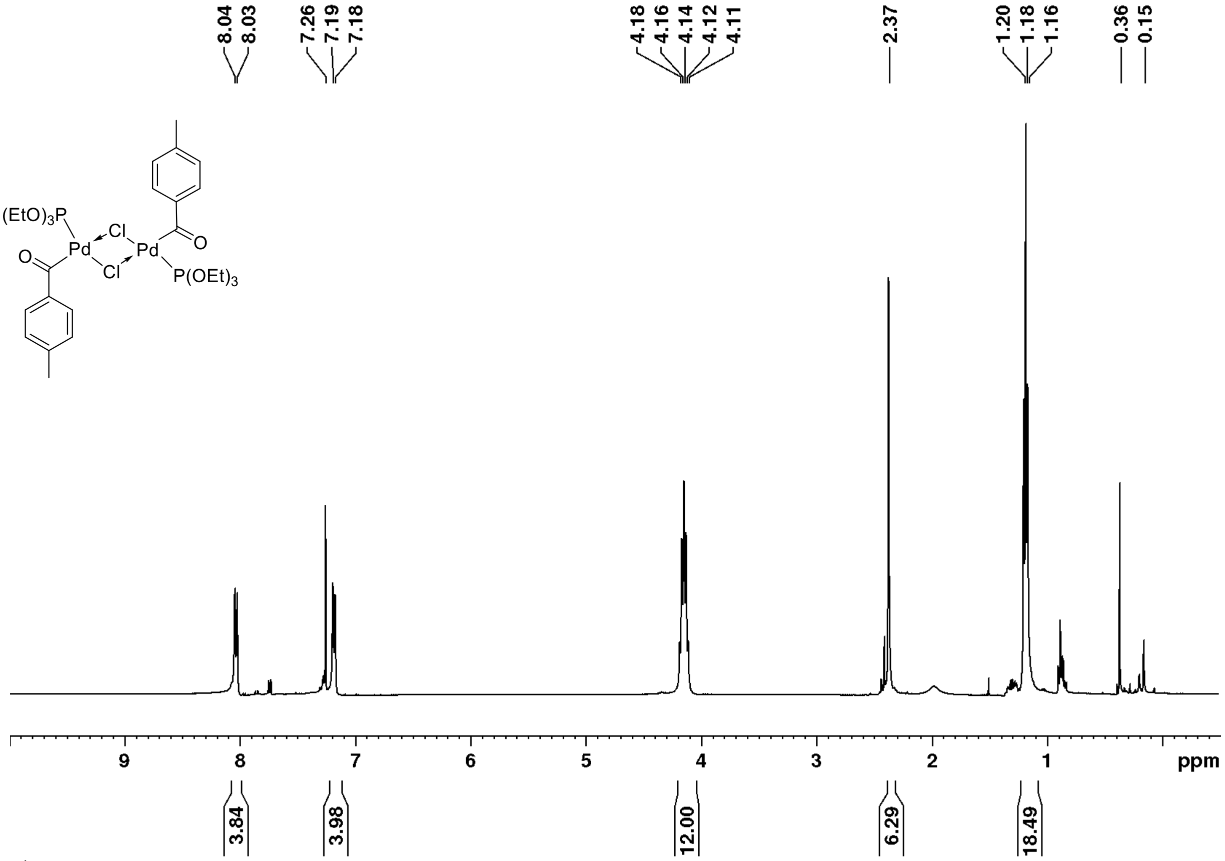

^{1}H NMR spectrum of {Pd[C(O)-*p*-tolyl]P(OEt)$_3$}$_2$(μ-Cl)$_2$ (**S2**) in CDCl$_3$ at 400 MHz

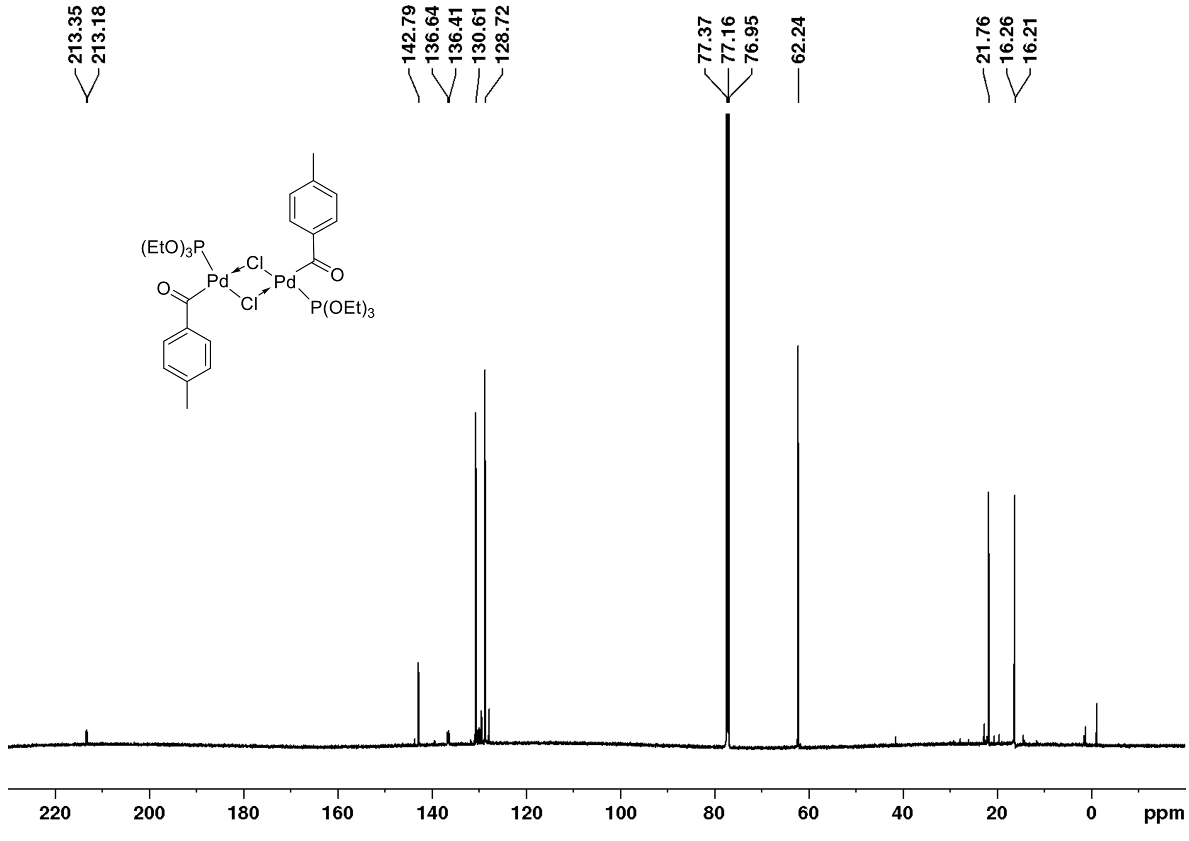

^{13}C{^{1}H} NMR spectrum of {Pd[C(O)-*p*-tolyl]P(OEt)$_3$}$_2$(μ-Cl)$_2$ (**S2**) in CDCl$_3$ at 151 MHz

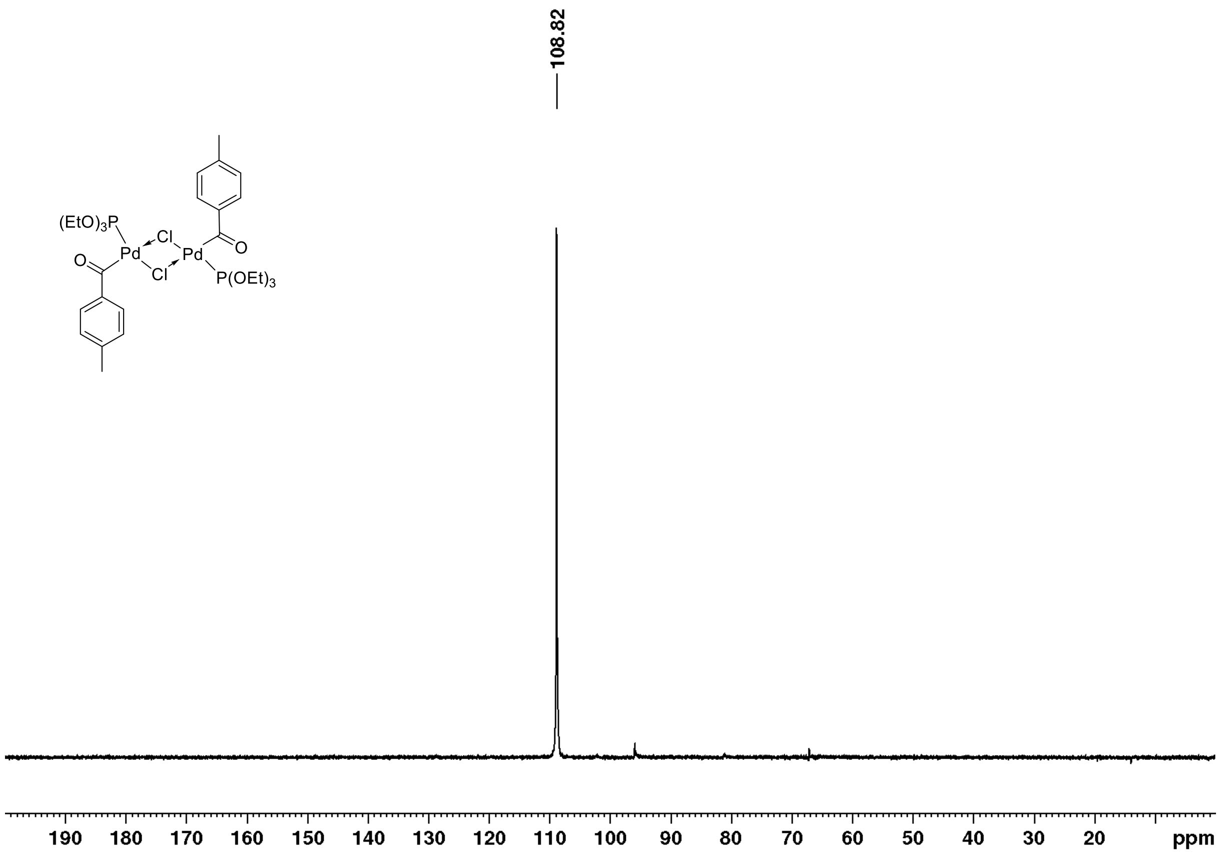

$^{31}P\{^1H\}$ NMR spectrum of $\{Pd[C(O)$-*p*-tolyl$]P(OEt)_3\}_2(\mu$-$Cl)_2$ (**S2**) in $CDCl_3$ at 243 MHz

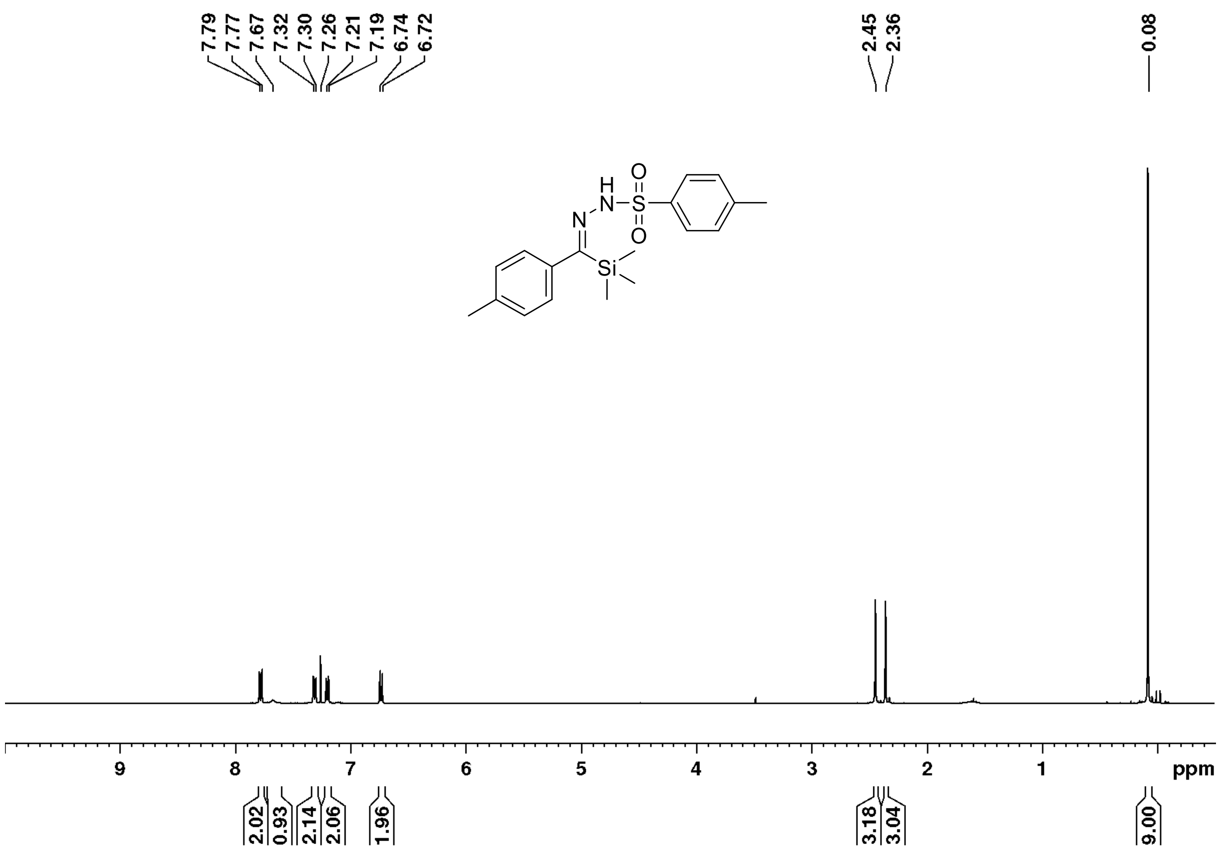

^{1}H NMR spectrum of **S3** in $CDCl_3$ at 400 MHz

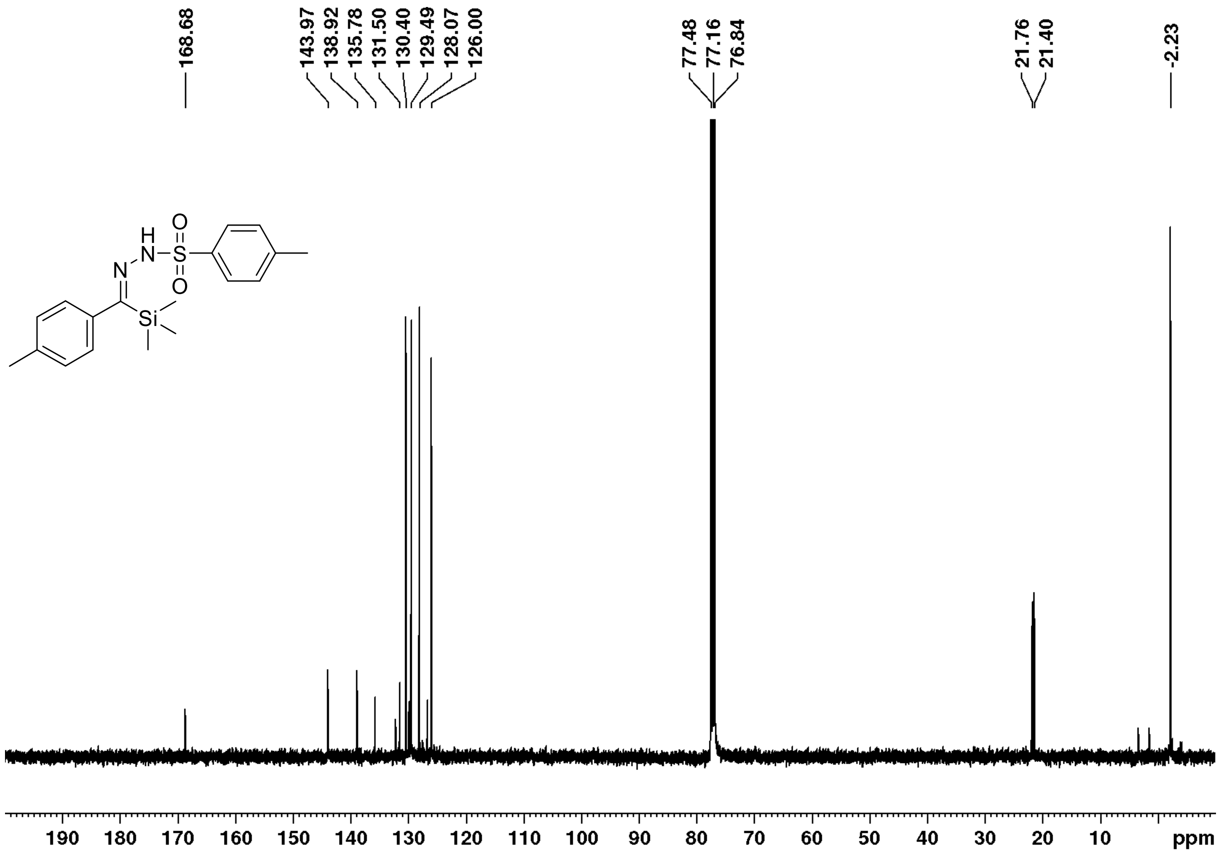

^{13}C{^{1}H} NMR spectrum of **S3** in $CDCl_3$ at 101 MHz

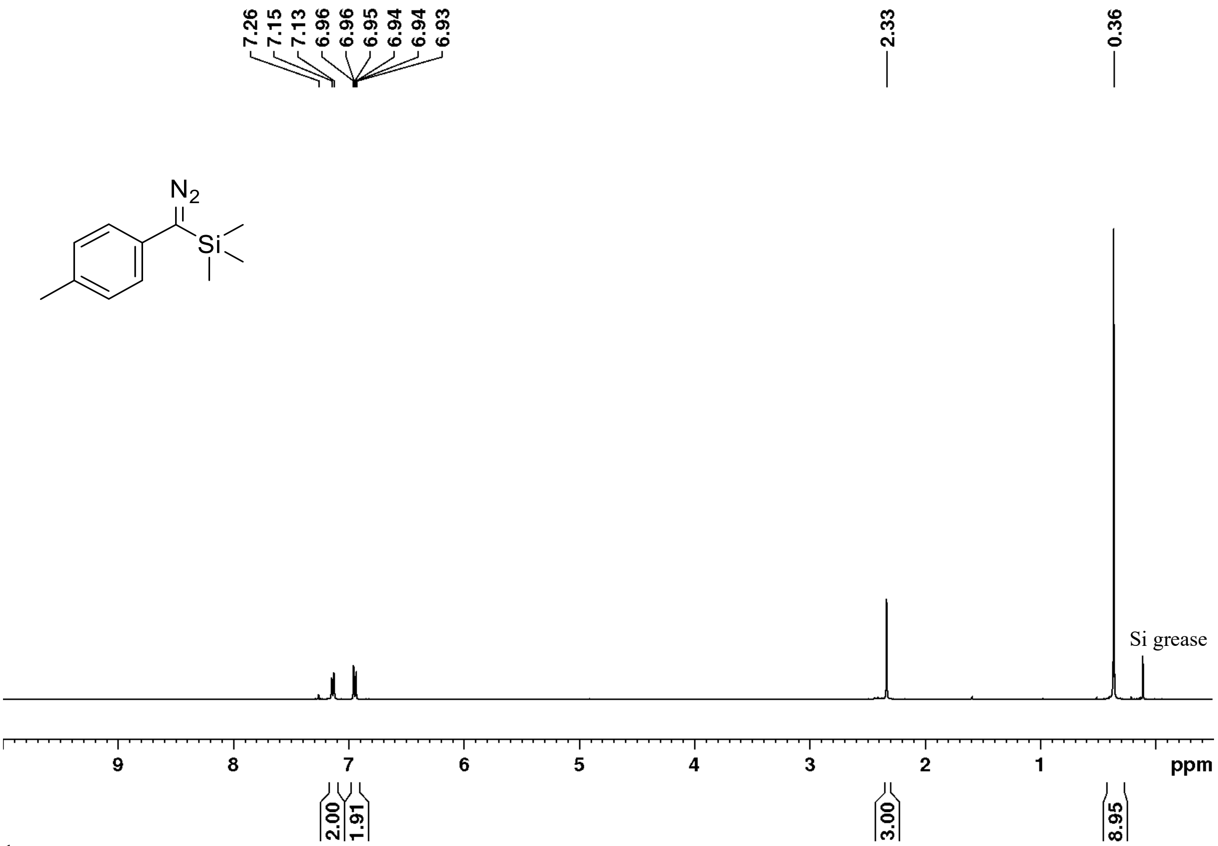

^{1}H NMR spectrum of **1** in $CDCl_3$ at 400 MHz

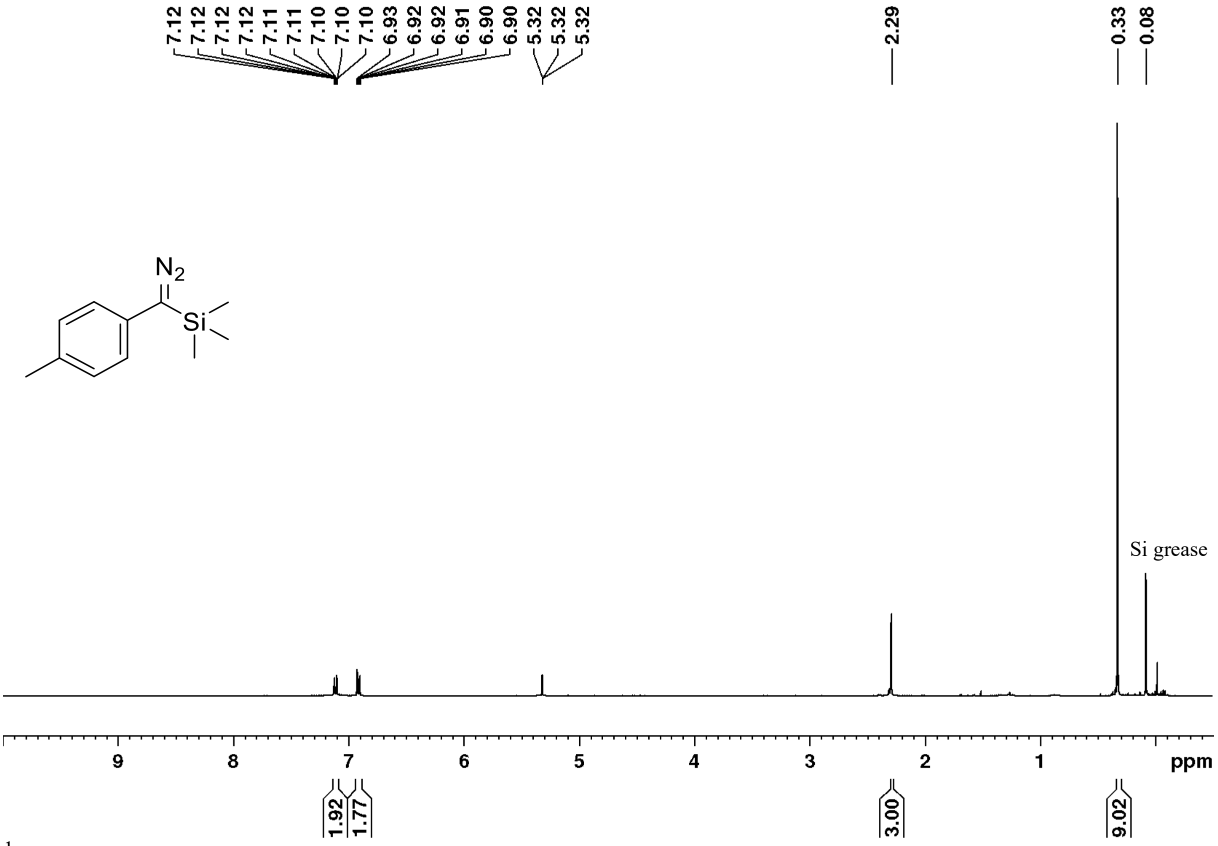

^{1}H NMR spectrum of **1** in CD_2Cl_2 at 400 MHz

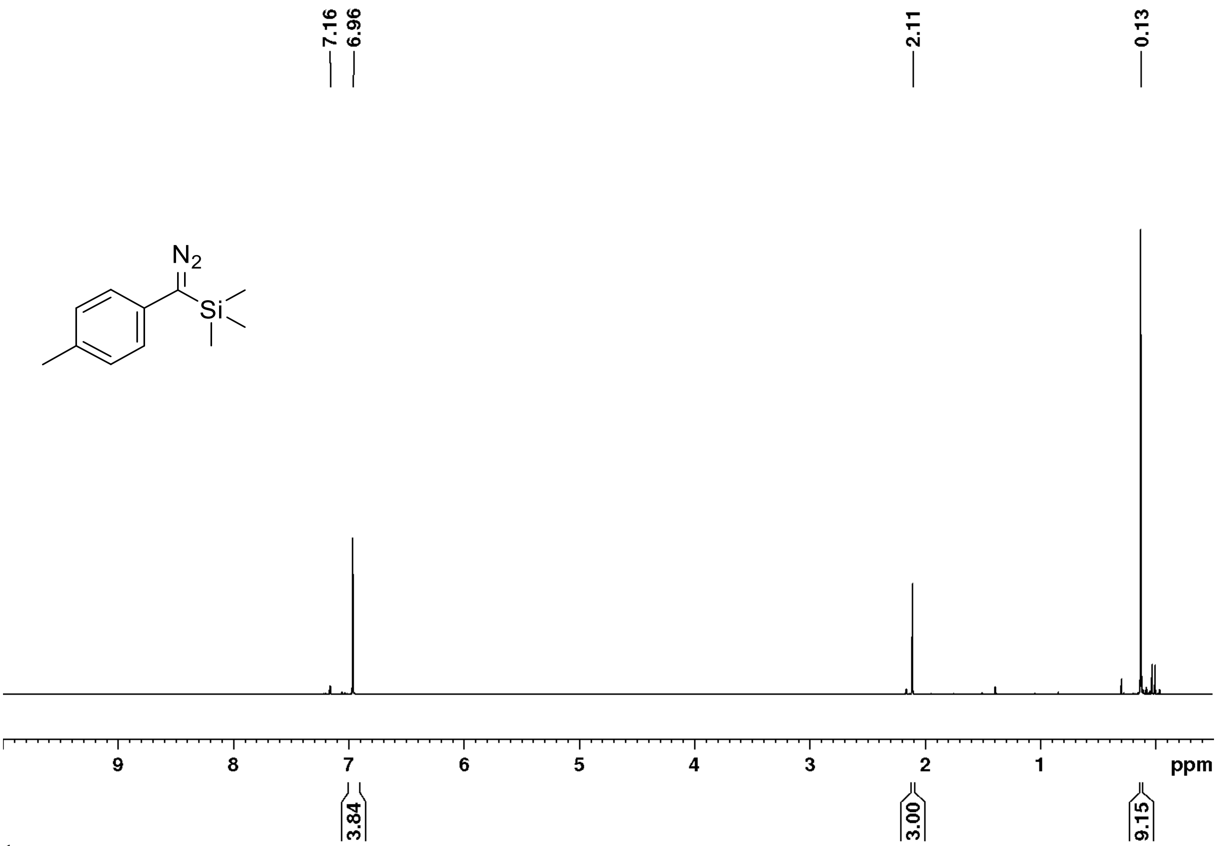

^{1}H NMR spectrum of **1** in C_6D_6 at 400 MHz

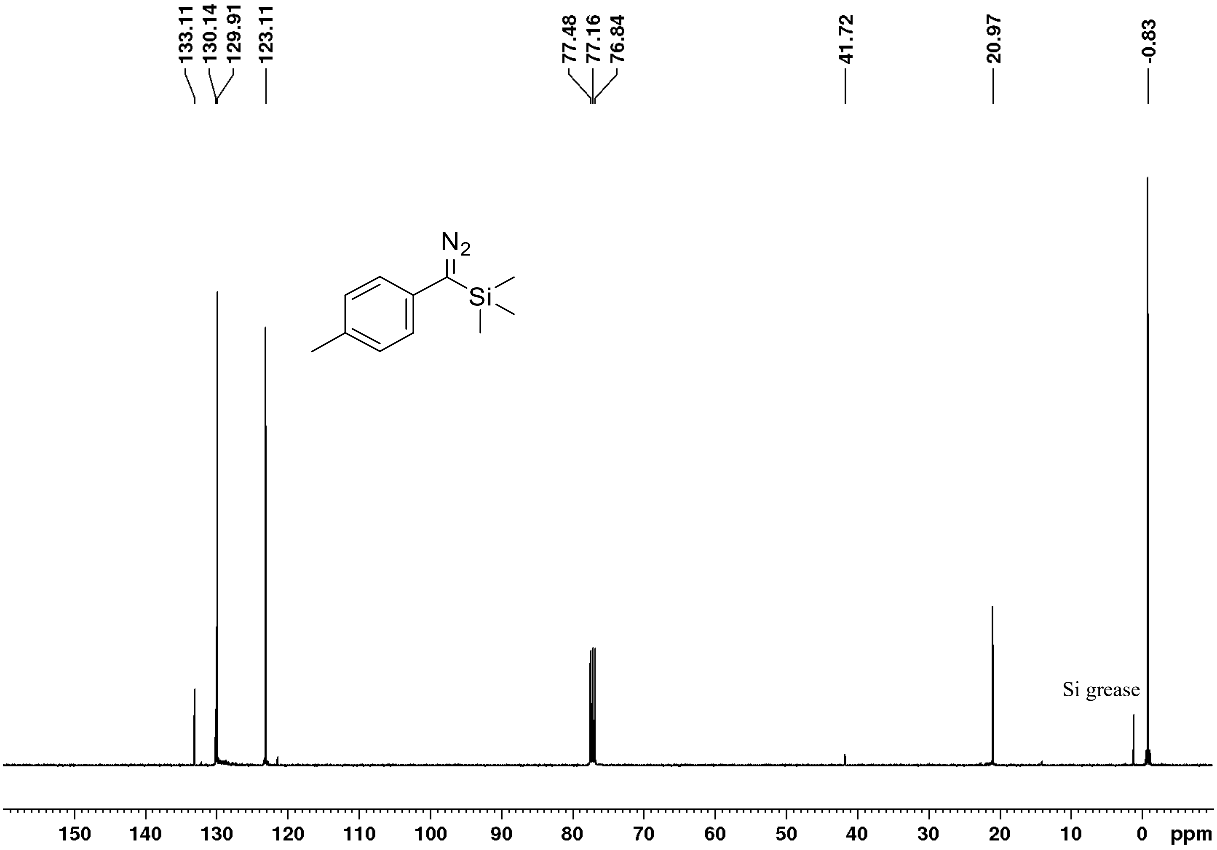

$^{13}C\{^{1}H\}$ NMR spectrum of **1** in $CDCl_3$ at 101 MHz

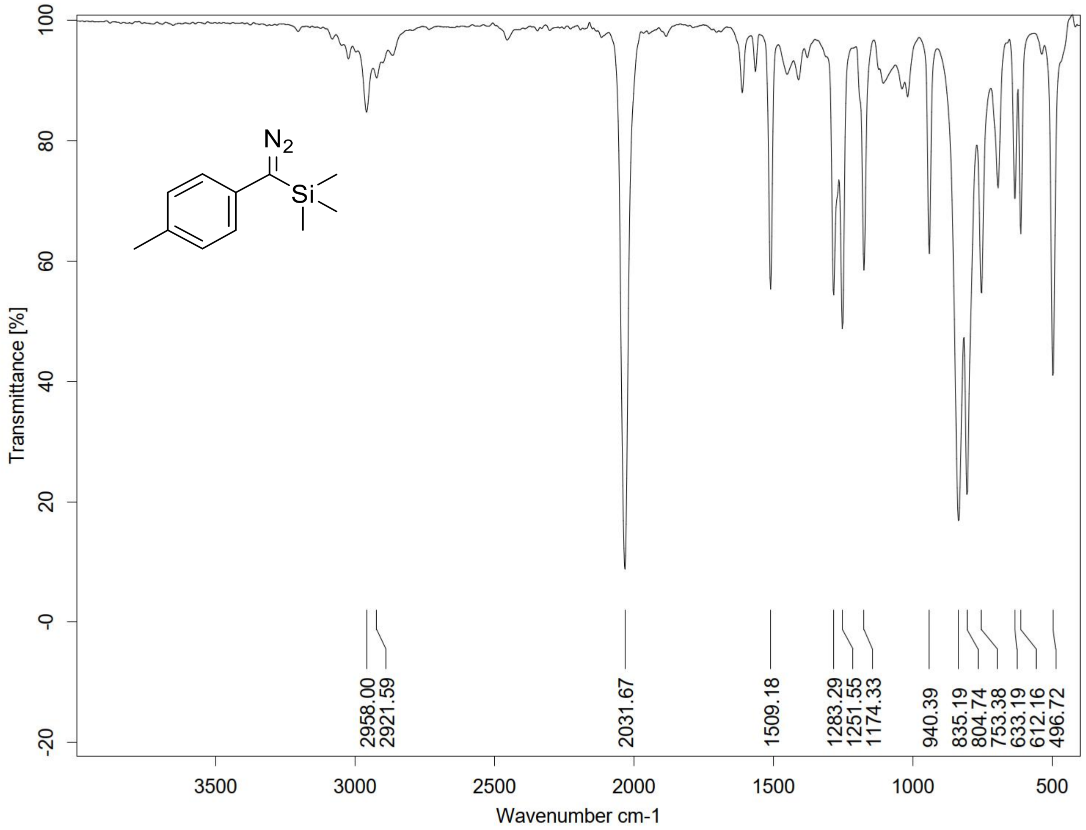

FT-IR spectrum of **1**

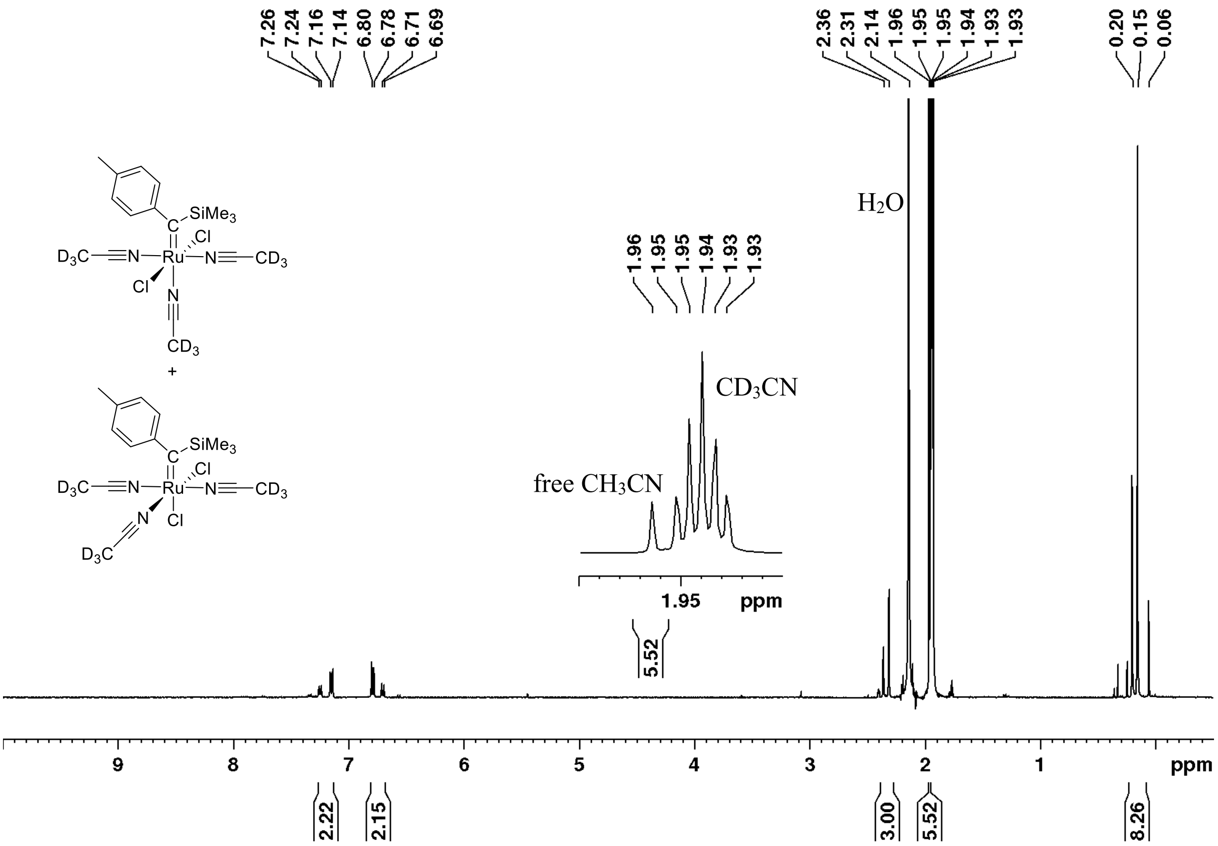

^{1}H NMR spectrum of **2** dissolved in CD_3CN at 400 MHz, showing a mixture of two isomeric complexes ligated to three CD_3CN ligands

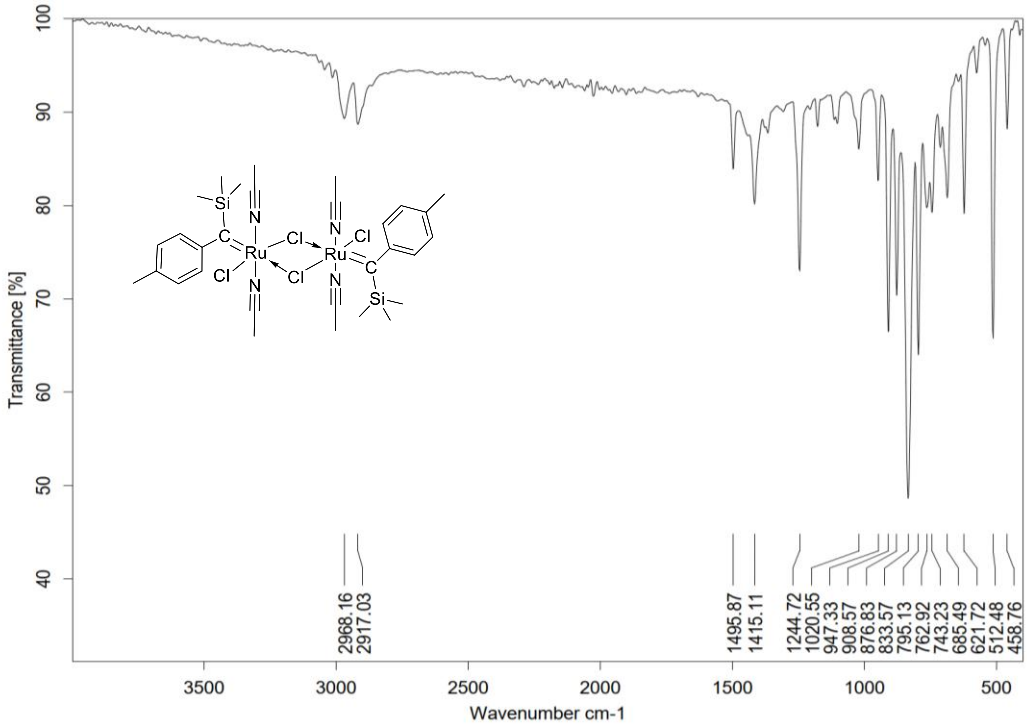

FT-IR spectrum of **2**

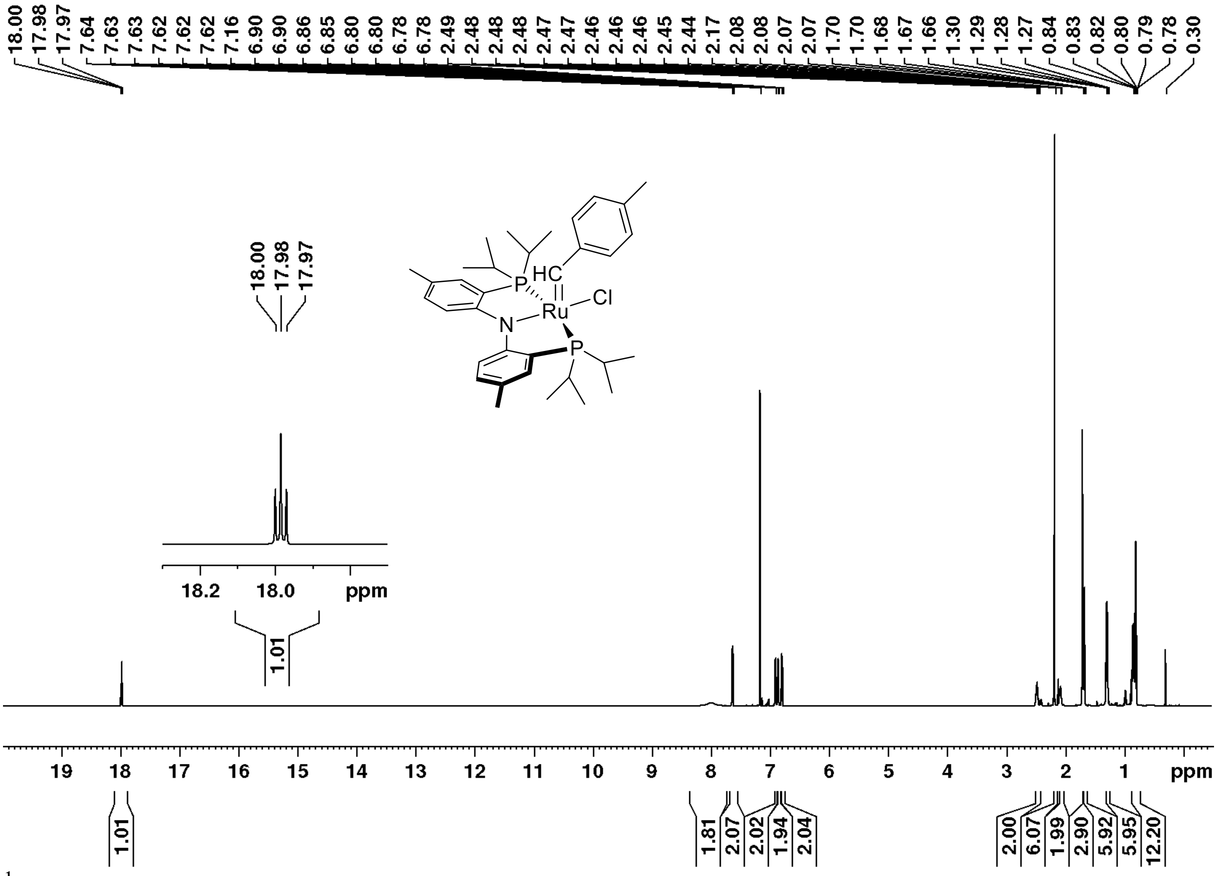

^{1}H NMR spectrum of ruthenium carbene **7** in C_6D_6 at 600 MHz

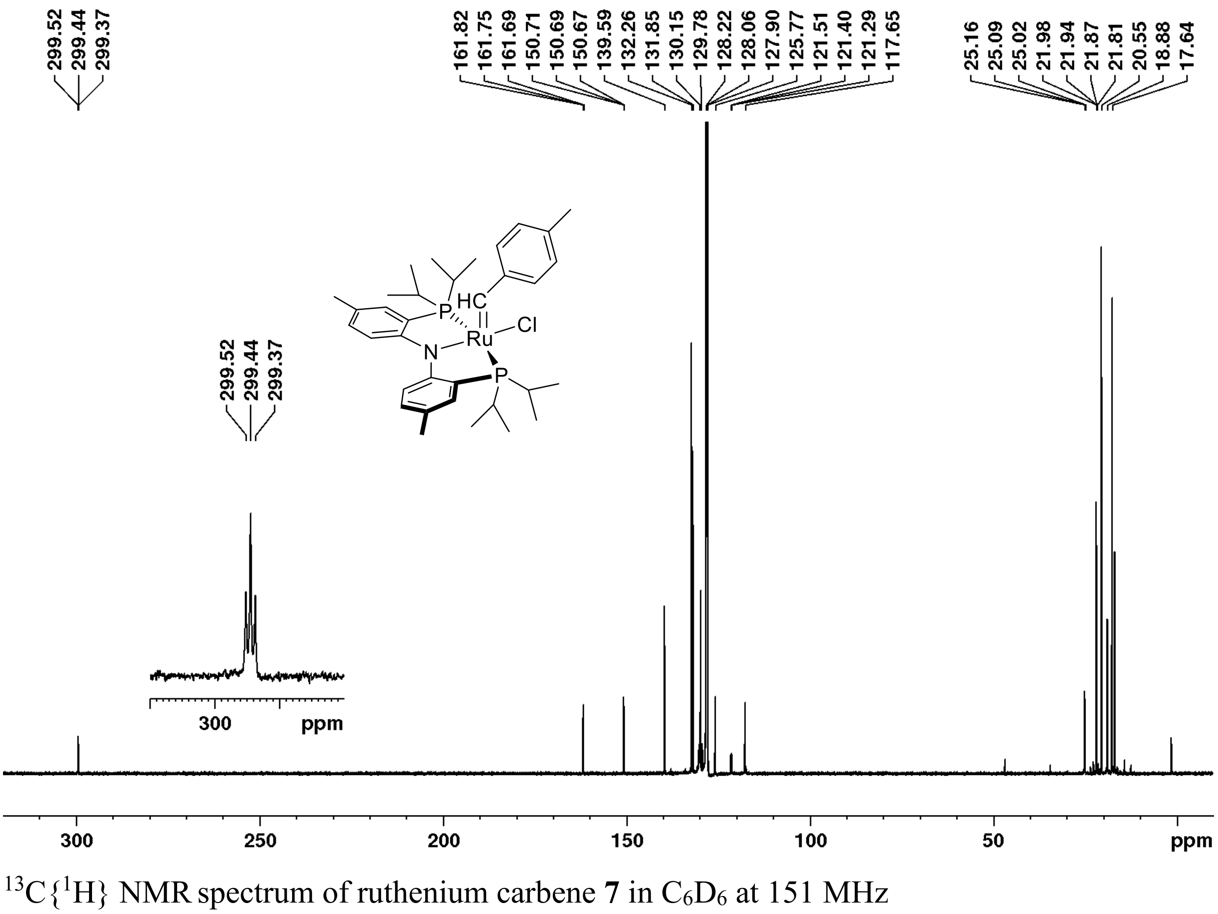

$^{13}C\{^{1}H\}$ NMR spectrum of ruthenium carbene **7** in C_6D_6 at 151 MHz

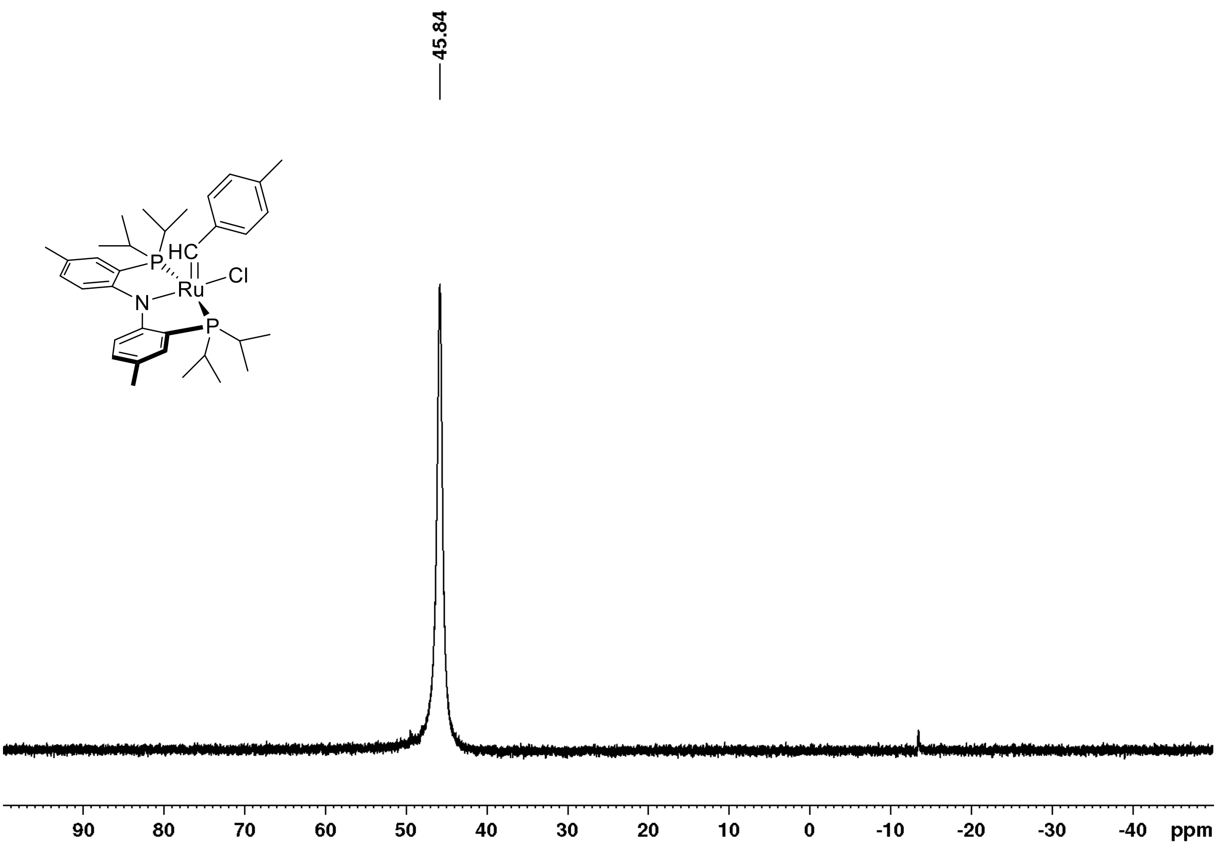

$^{31}P\{^{1}H\}$ NMR spectrum of ruthenium carbene **7** in C_6D_6 at 162 MHz

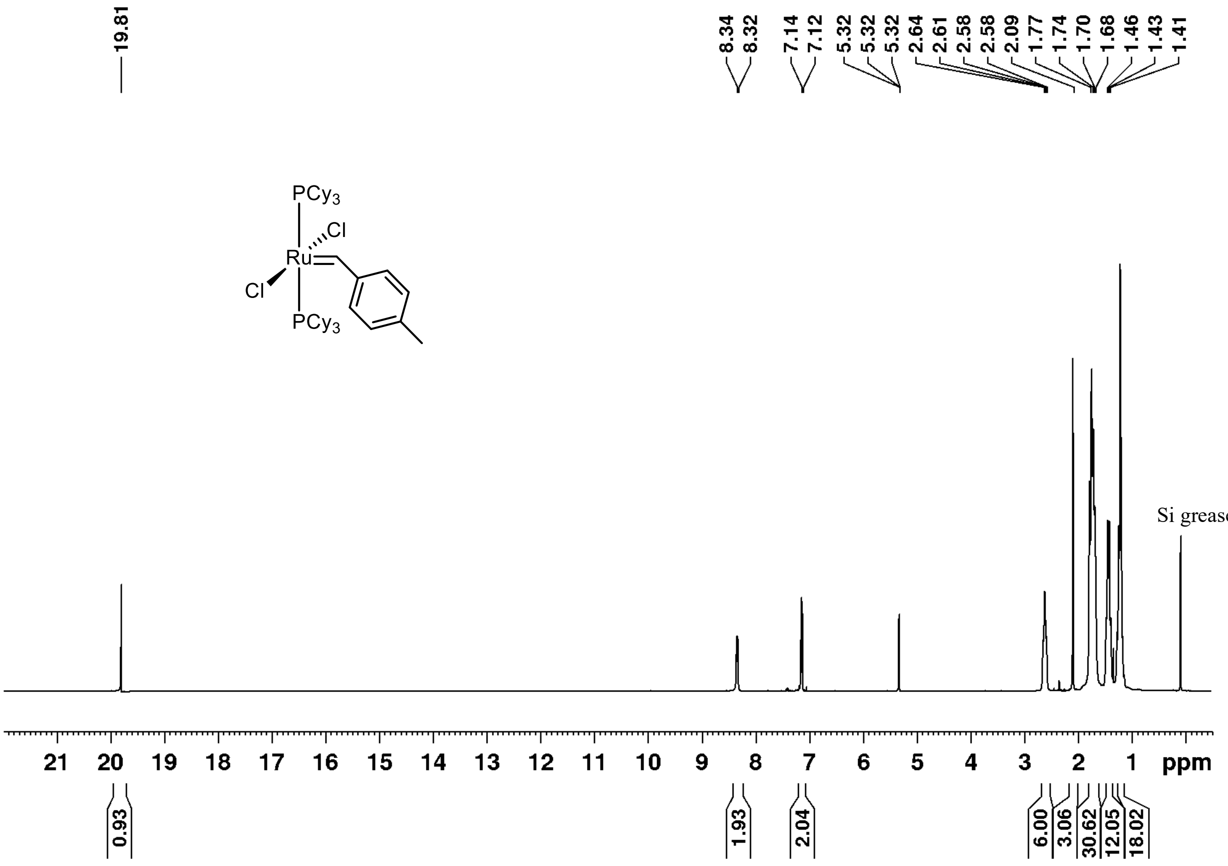

^{1}H NMR spectrum of complex **8** in CD_2Cl_2 at 400 MHz

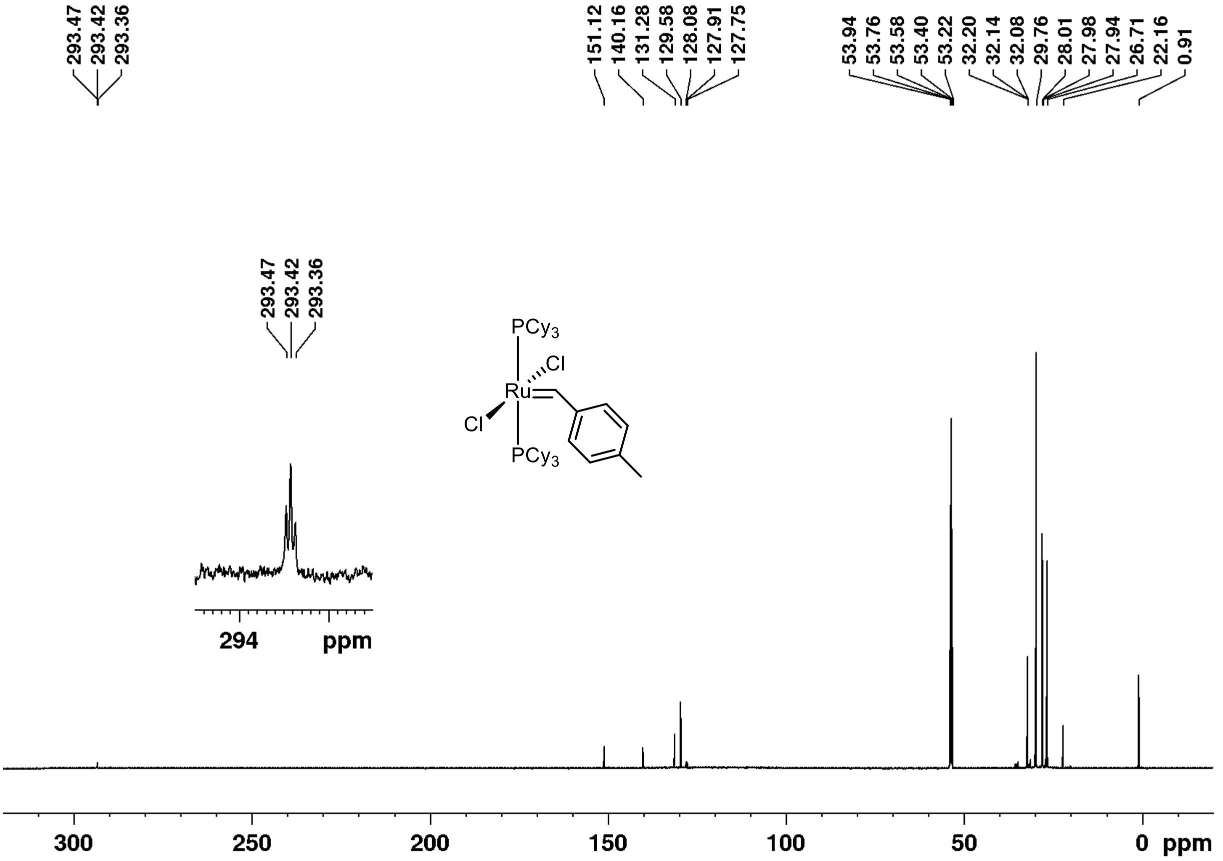

^{13}C{^{1}H} NMR spectrum of **8** in CD_2Cl_2 at 151 MHz

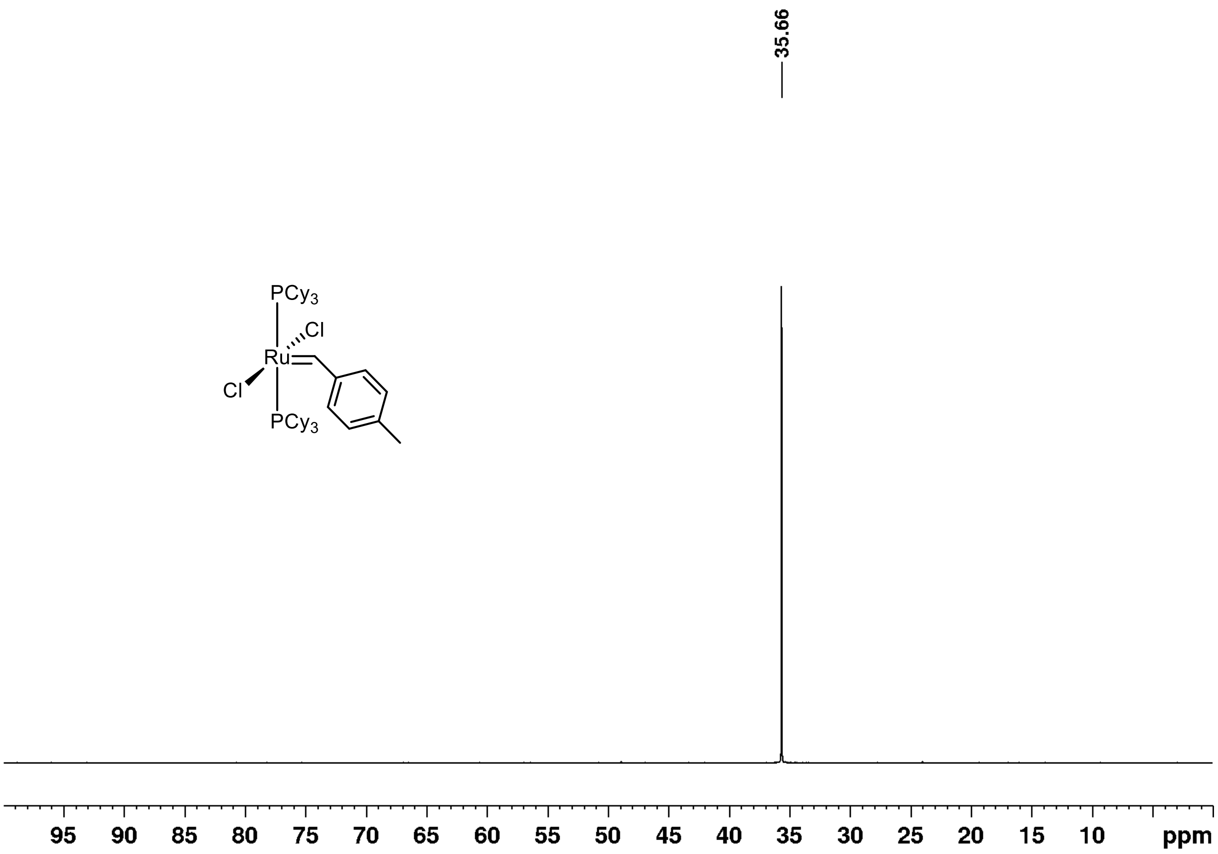

$^{31}P\{^{1}H\}$ NMR spectrum of **8** in CD_2Cl_2 at 162 MHz

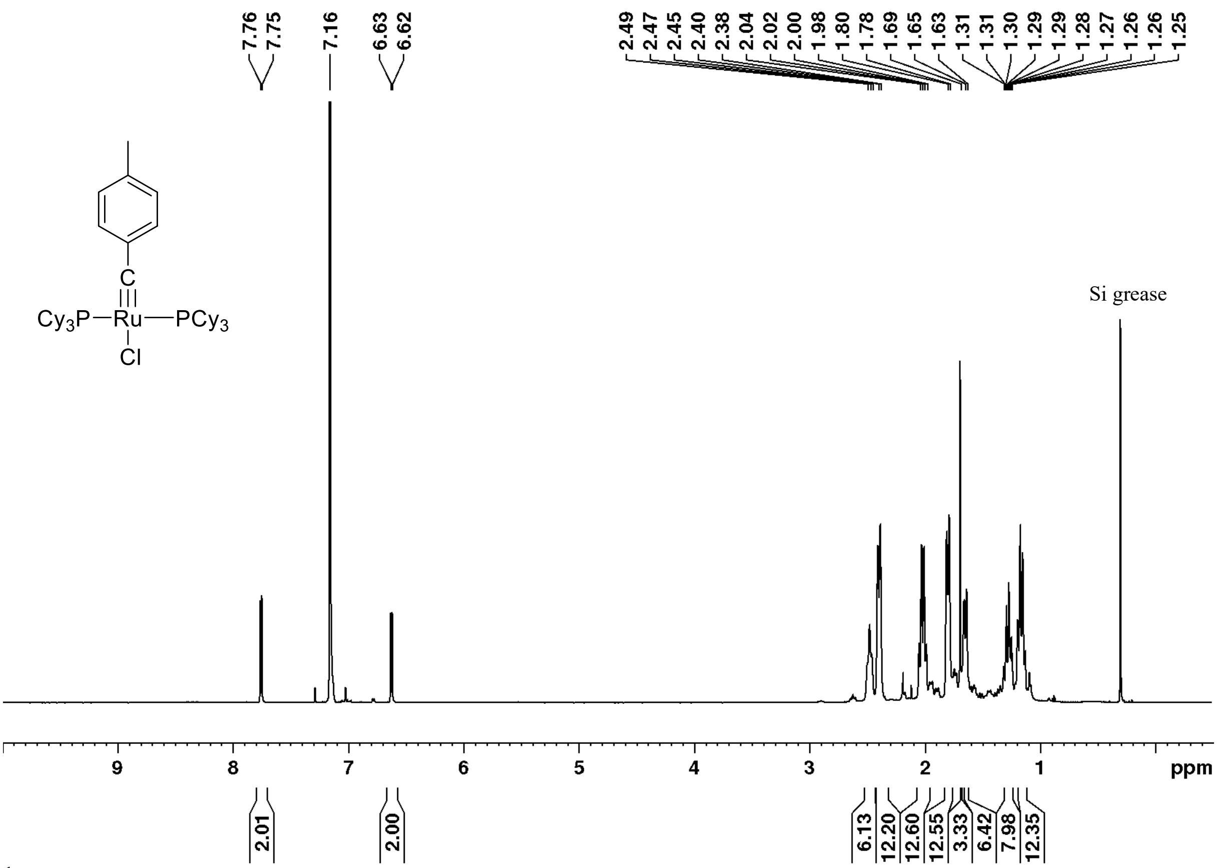

^1H NMR spectrum of ruthenium carbyne **9** in C_6D_6 at 600 MHz

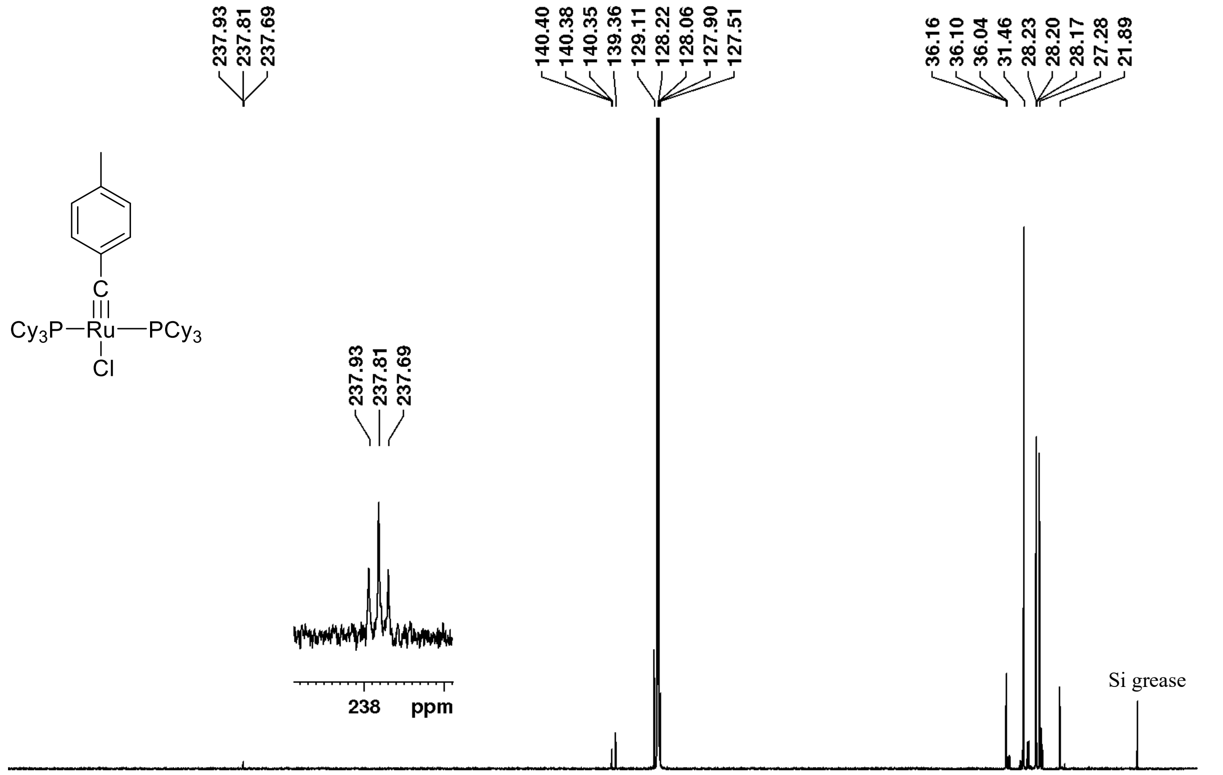

$^{13}C\{^1H\}$ NMR spectrum of ruthenium carbyne **9** in C_6D_6 at 151 MHz

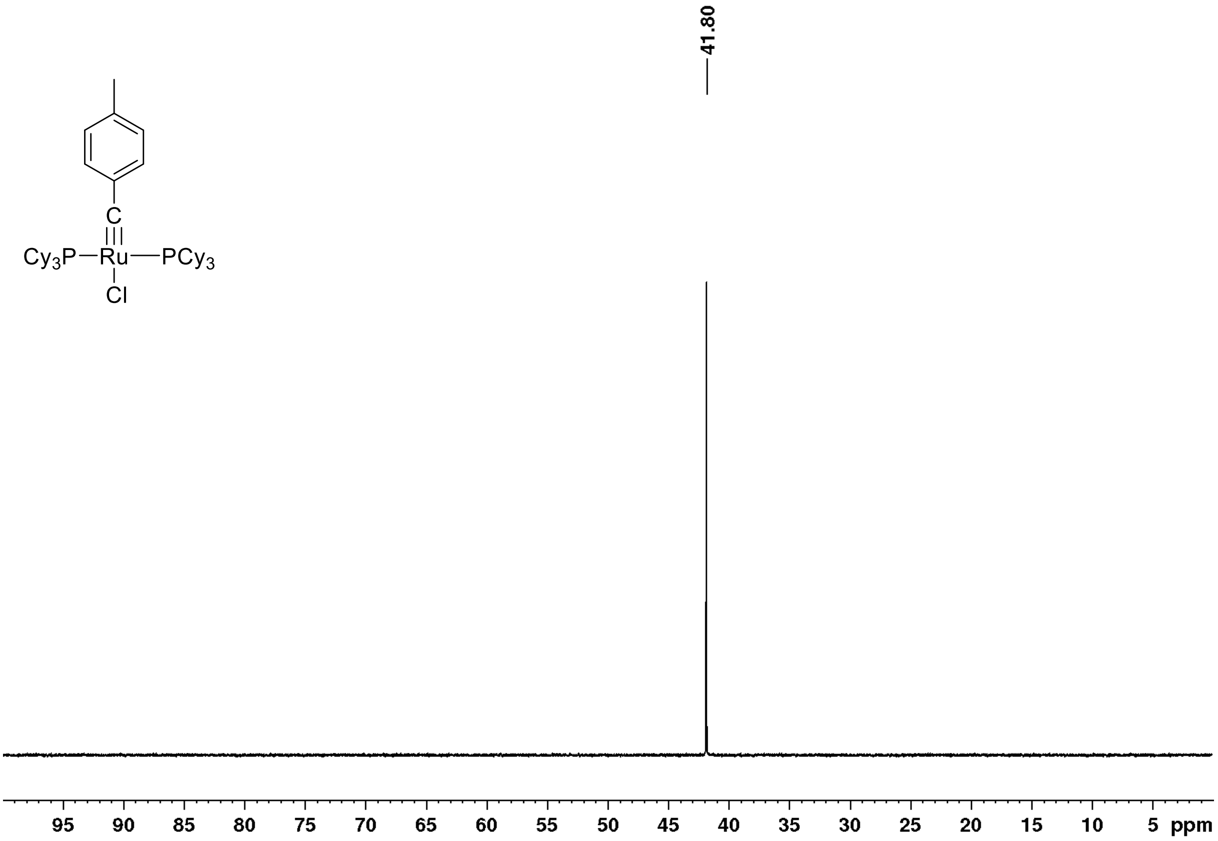

$^{31}P\{^{1}H\}$ NMR spectrum of ruthenium carbyne **9** in C_6D_6 at 162 MHz

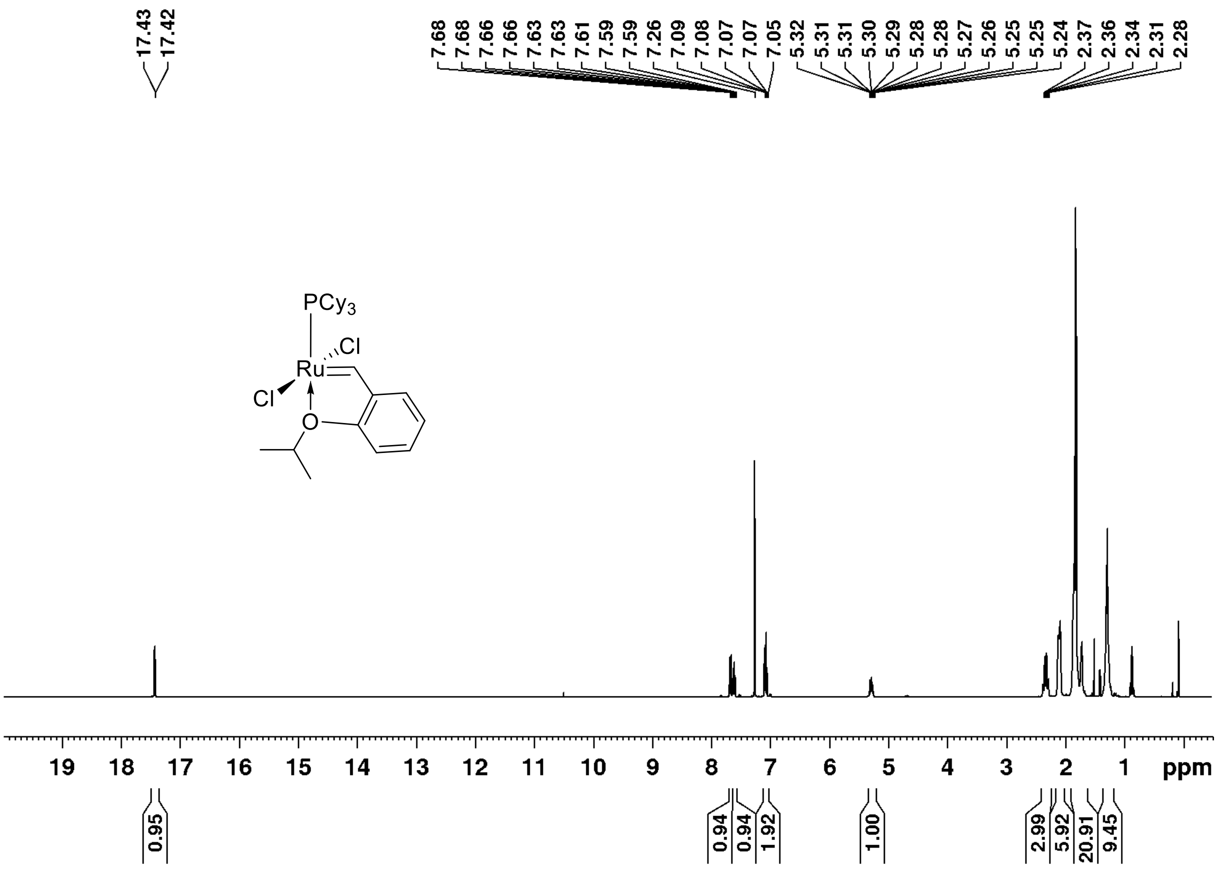

^{1}H NMR spectrum of complex **14a** in $CDCl_3$ at 400 MHz

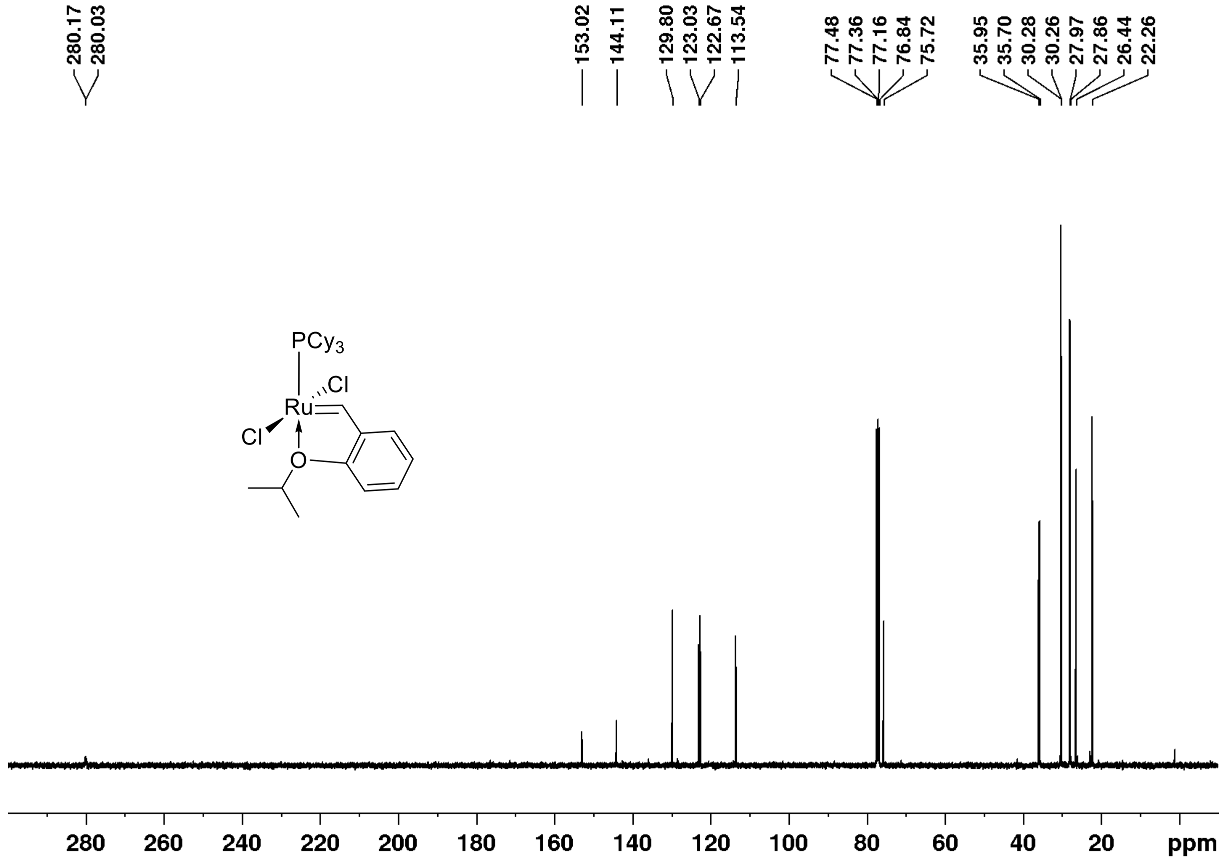

$^{13}C\{^1H\}$ NMR spectrum of complex **14a** in $CDCl_3$ at 101 MHz

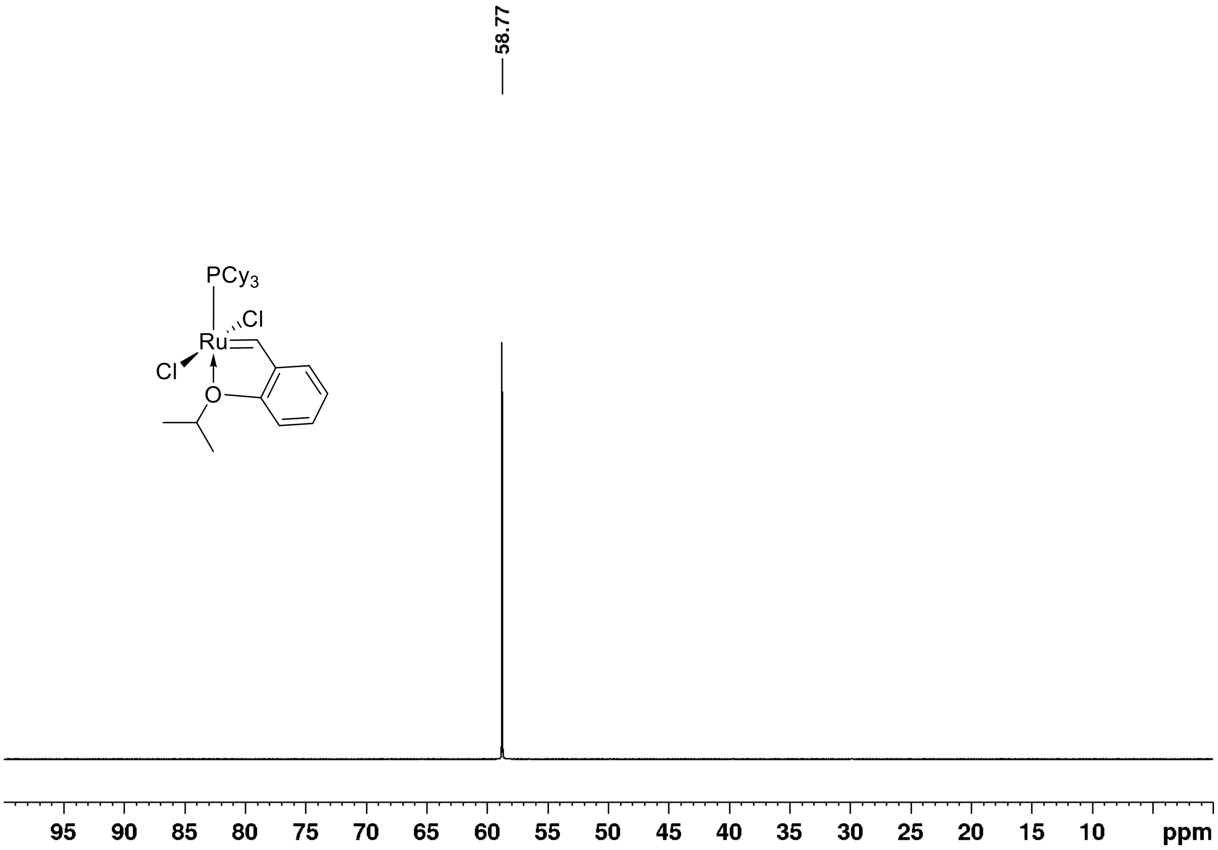

$^{31}P\{^1H\}$ NMR spectrum of complex **14a** in $CDCl_3$ at 162 MHz

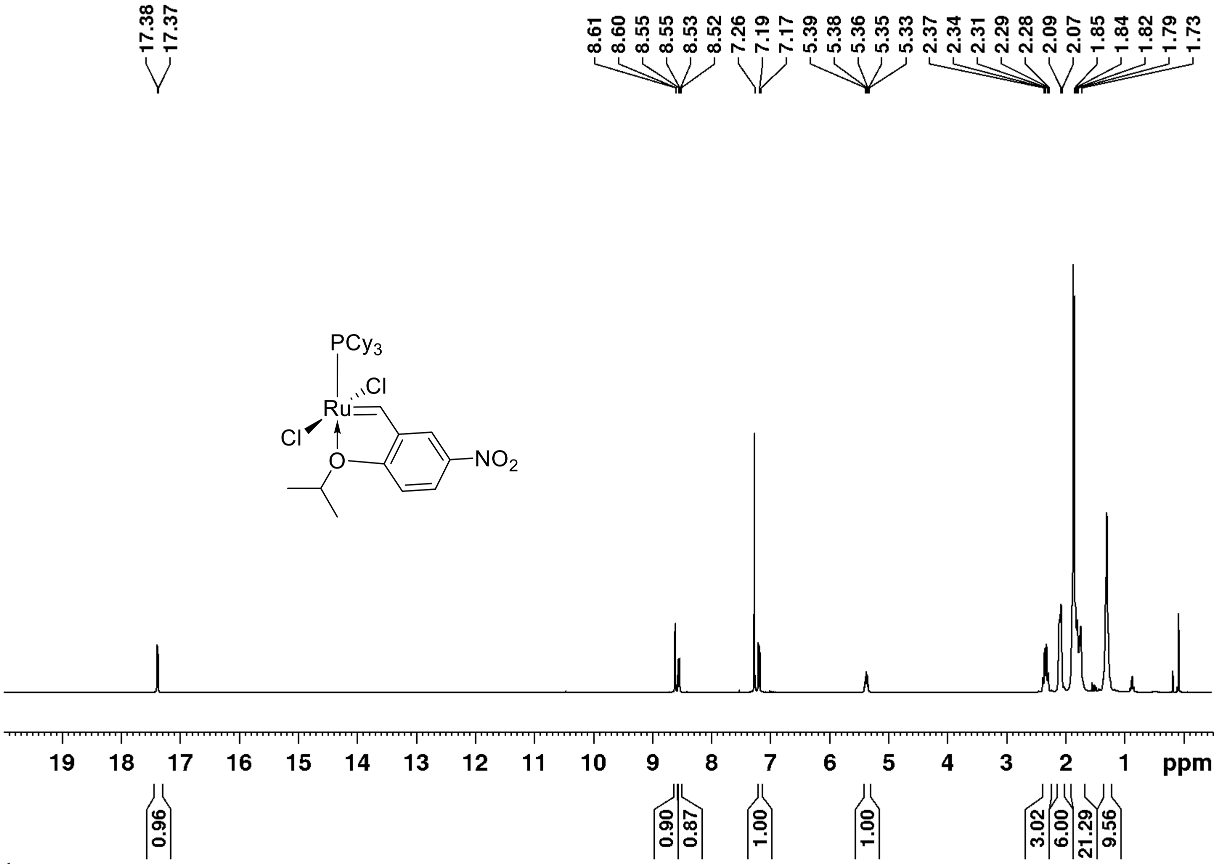

^{1}H NMR spectrum of complex **14b** in $CDCl_3$ at 400 MHz

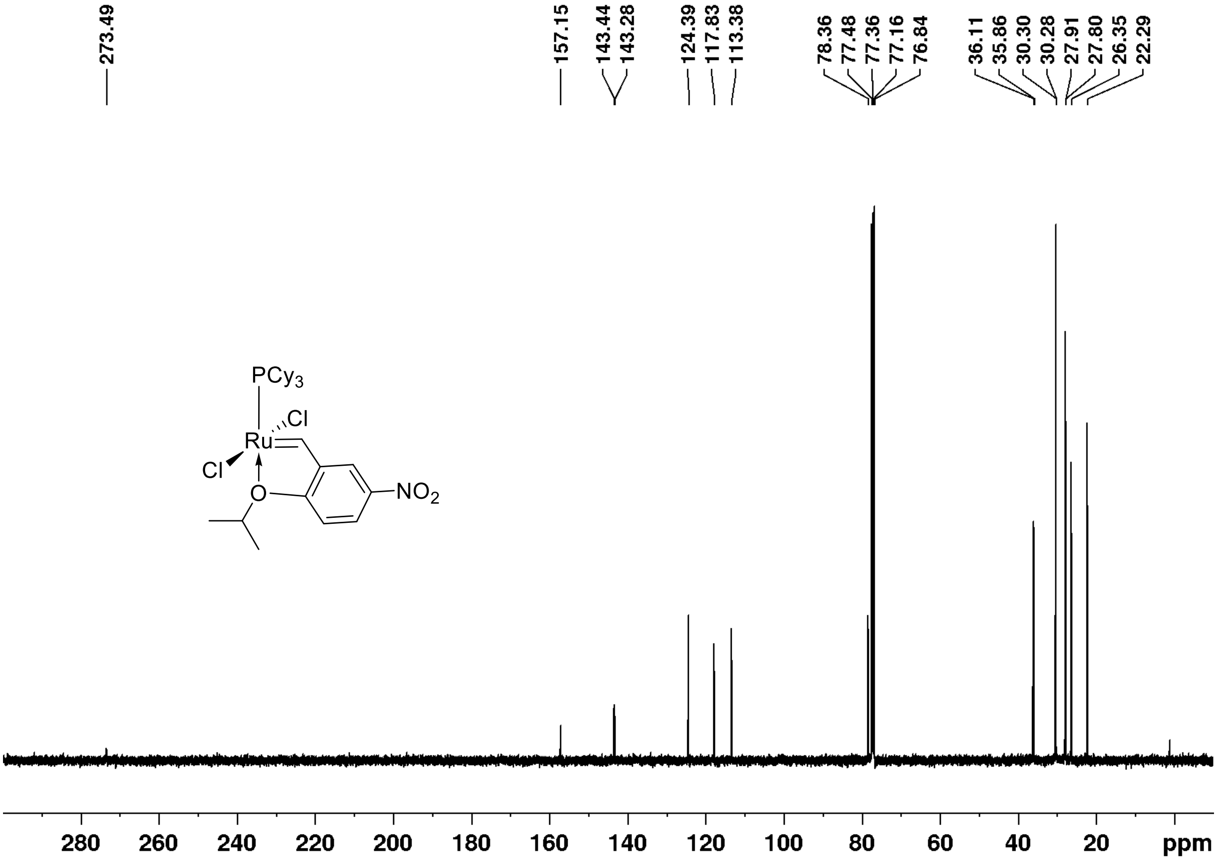

^{13}C{^{1}H} NMR spectrum of complex **14b** in $CDCl_3$ at 101 MHz

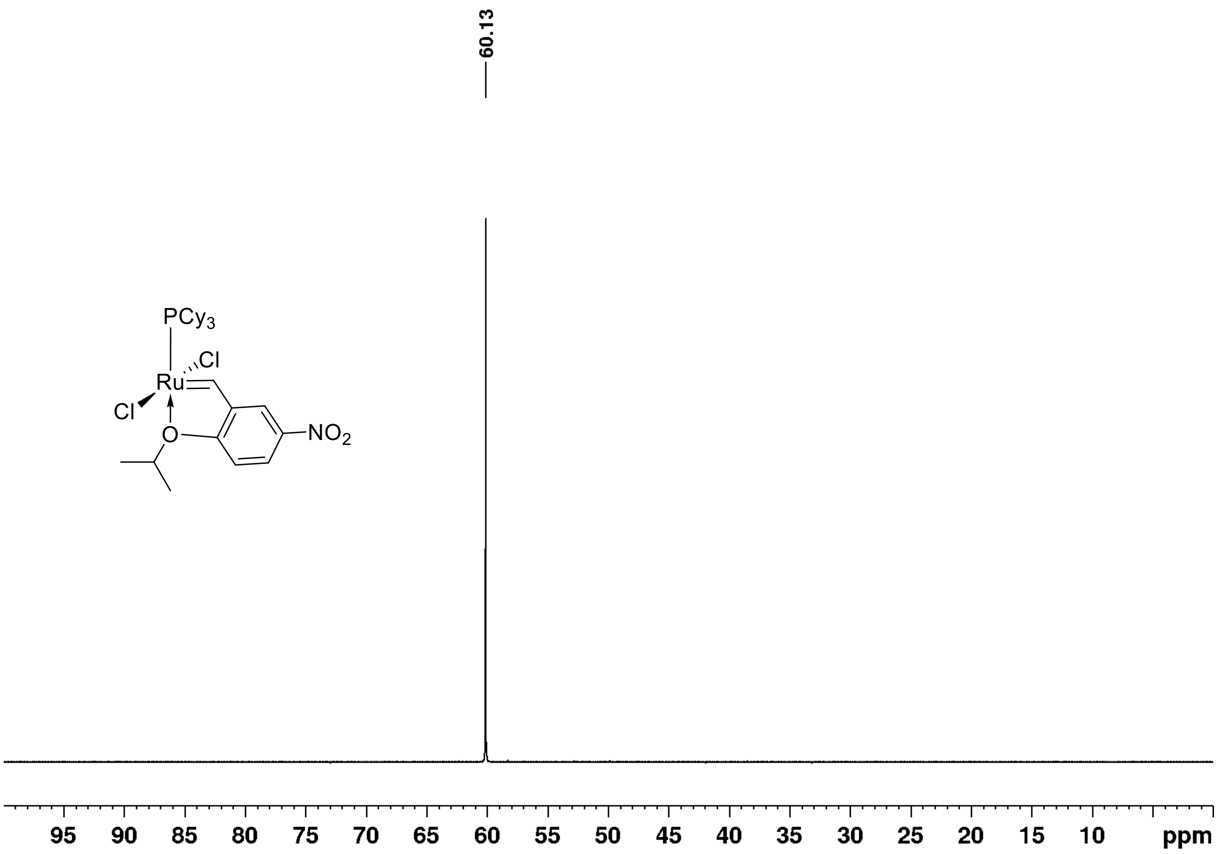

$^{31}P\{^{1}H\}$ NMR spectrum of complex **14b** in $CDCl_3$ at 162 MHz

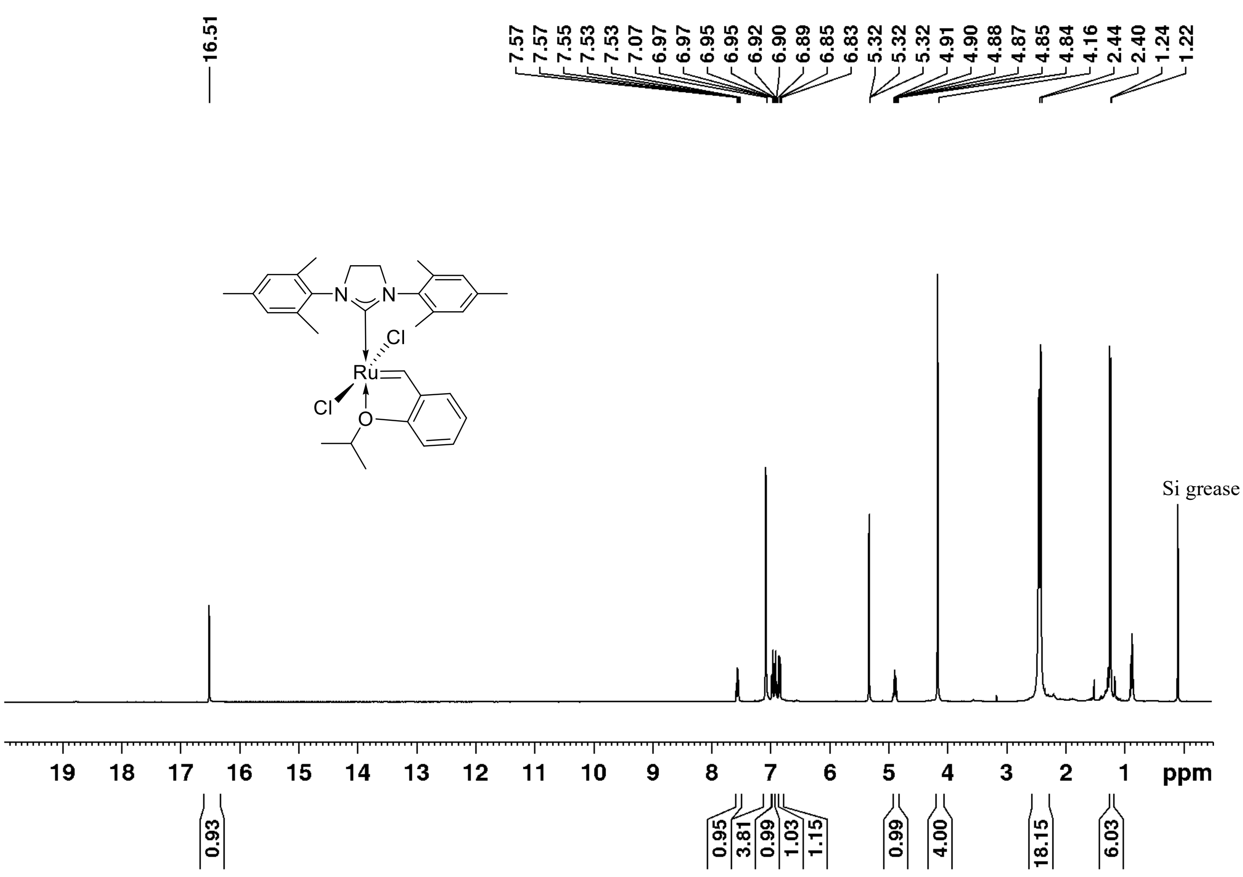

^{1}H NMR spectrum of complex **15** in CD_2Cl_2 at 400 MHz

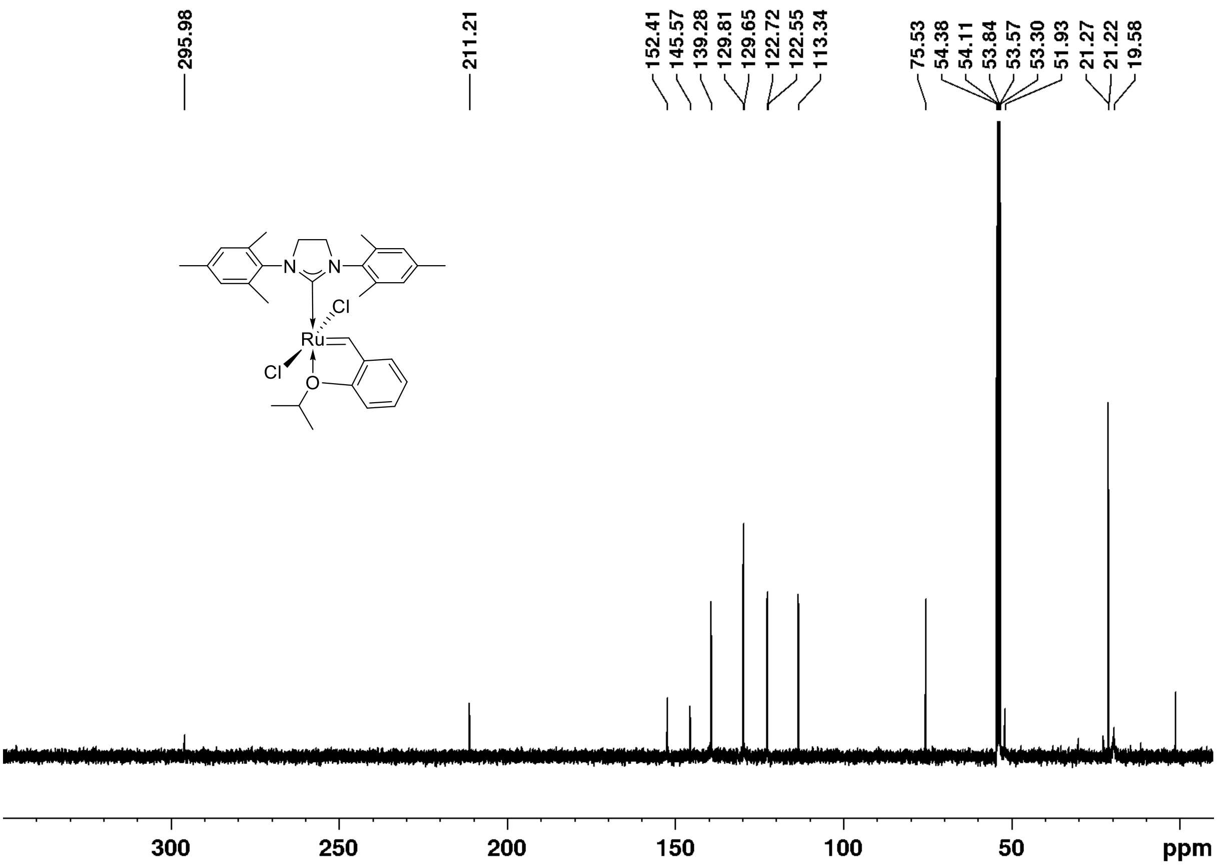

$^{13}C\{^{1}H\}$ NMR spectrum of complex **15** in CD_2Cl_2 at 101 MHz

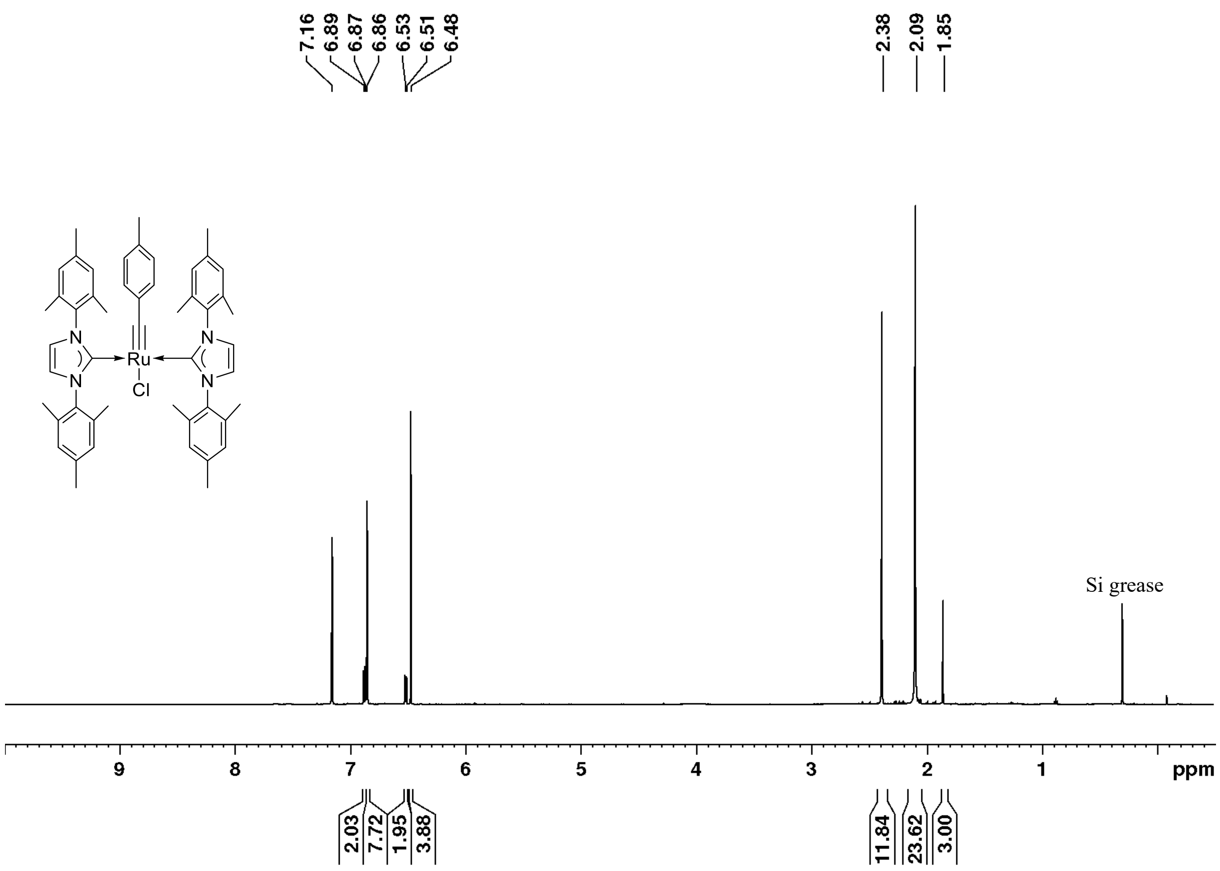

^{1}H NMR spectrum of complex **25** in C_6D_6 at 600 MHz

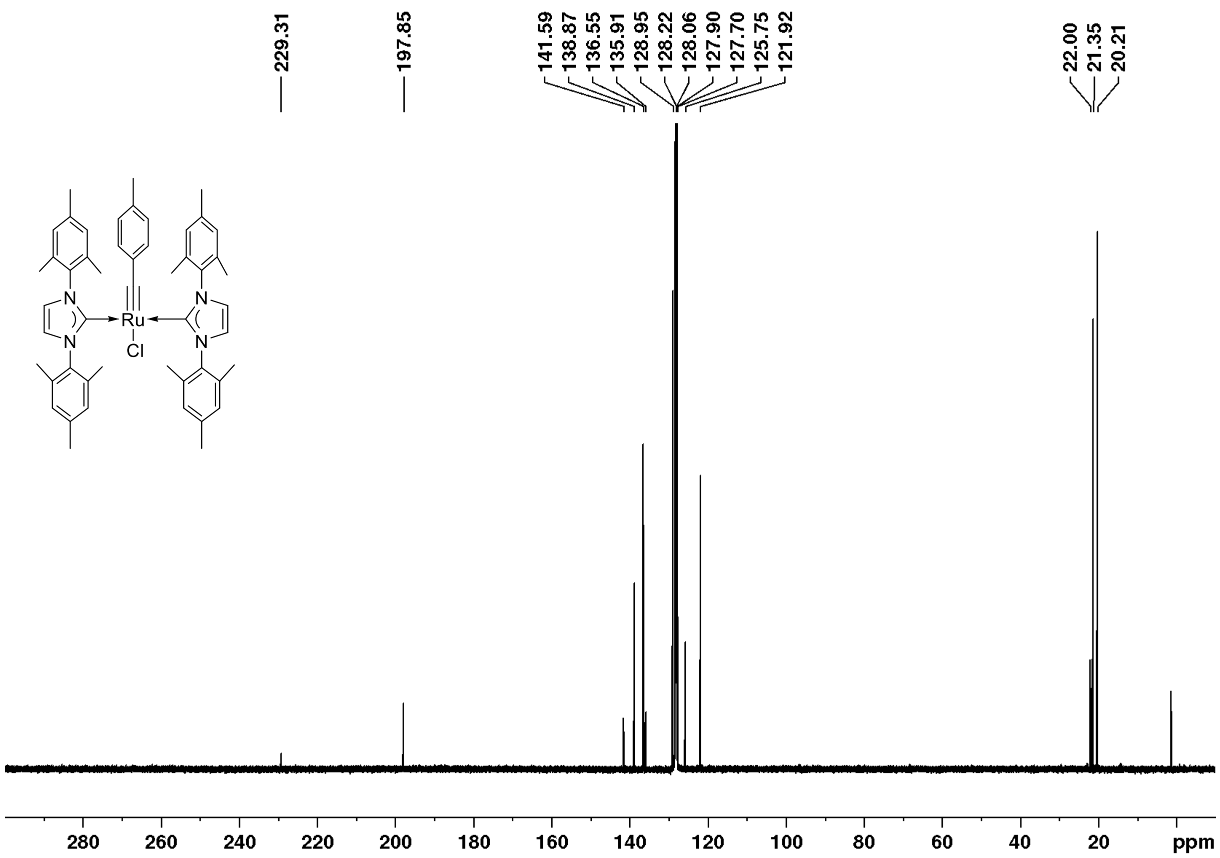

^{13}C{^{1}H} NMR spectrum of complex **25** in C_6D_6 at 151 MHz

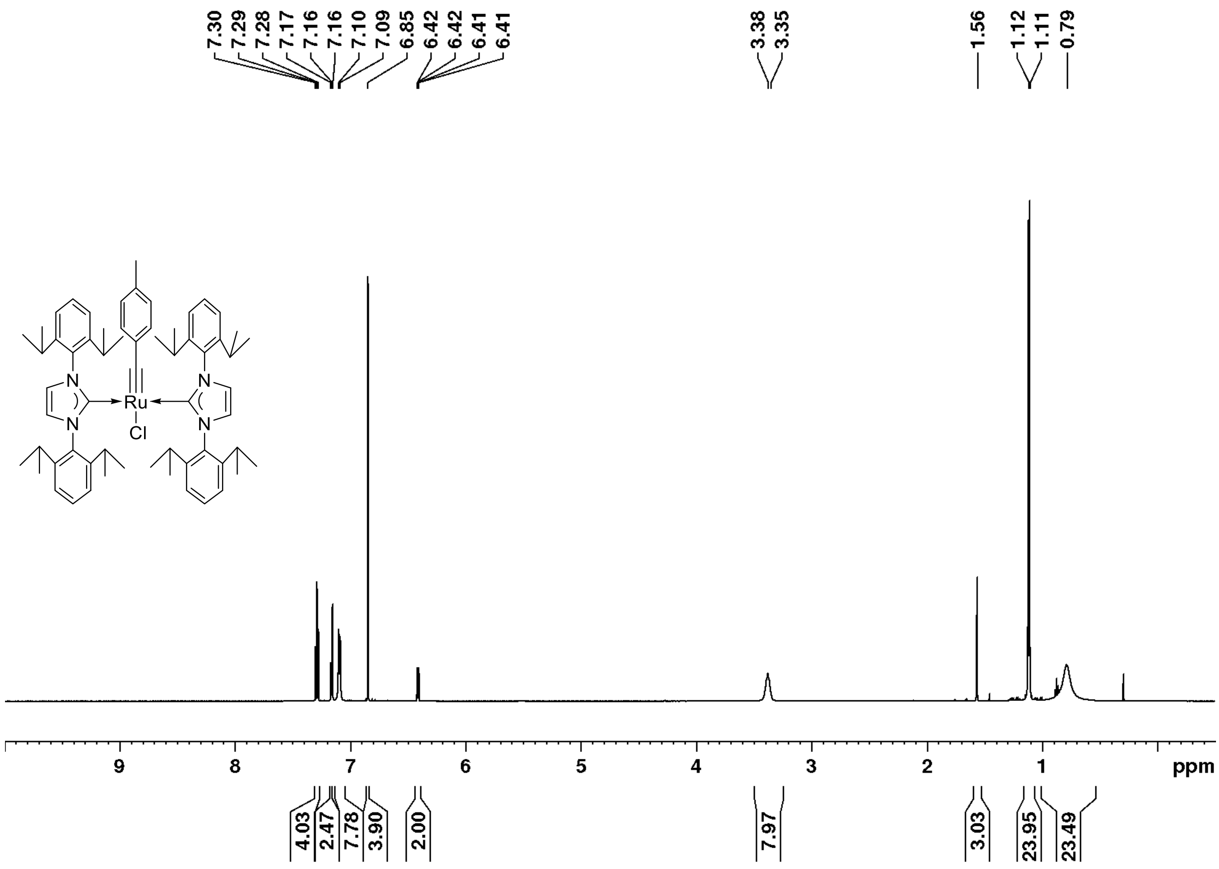

^{1}H NMR spectrum of complex **26** in C_6D_6 at 600 MHz

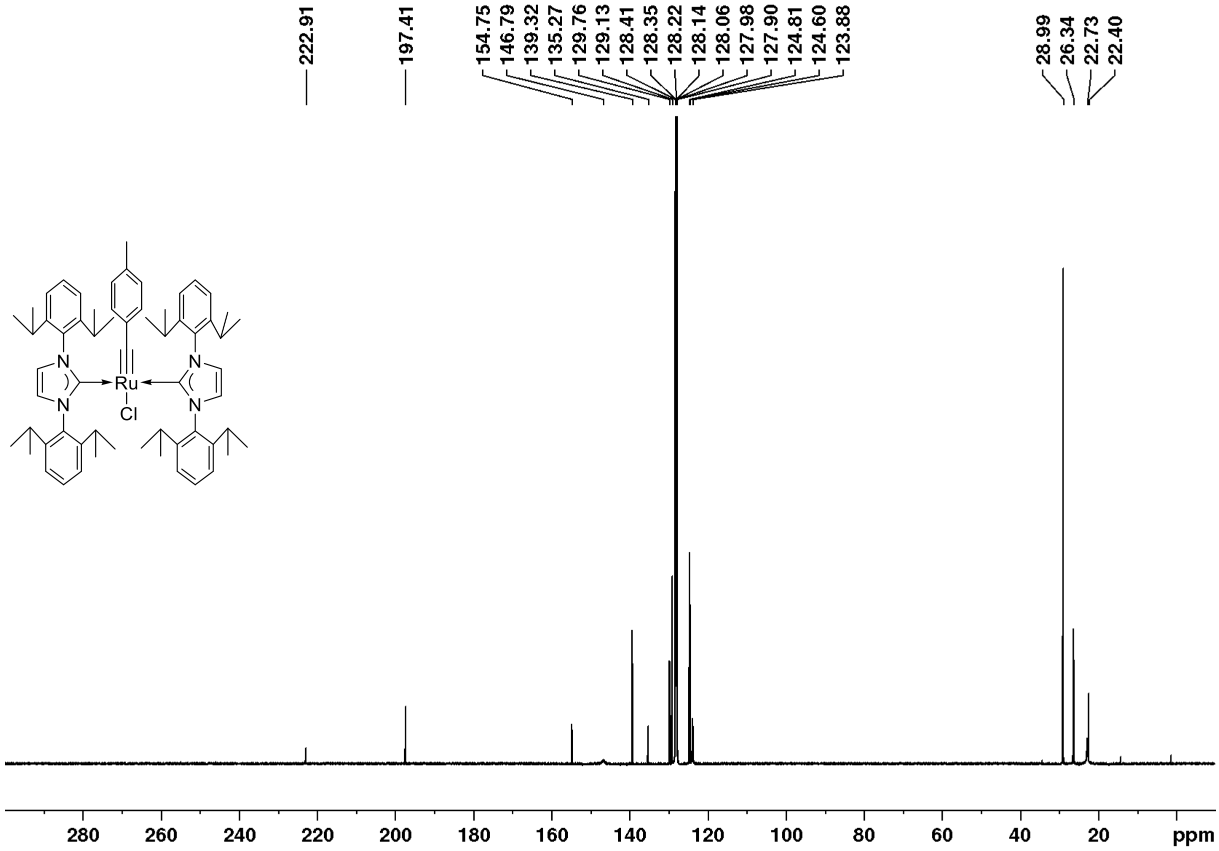

^{13}C{^{1}H} NMR spectrum of complex **26** in C_6D_6 at 151 MHz

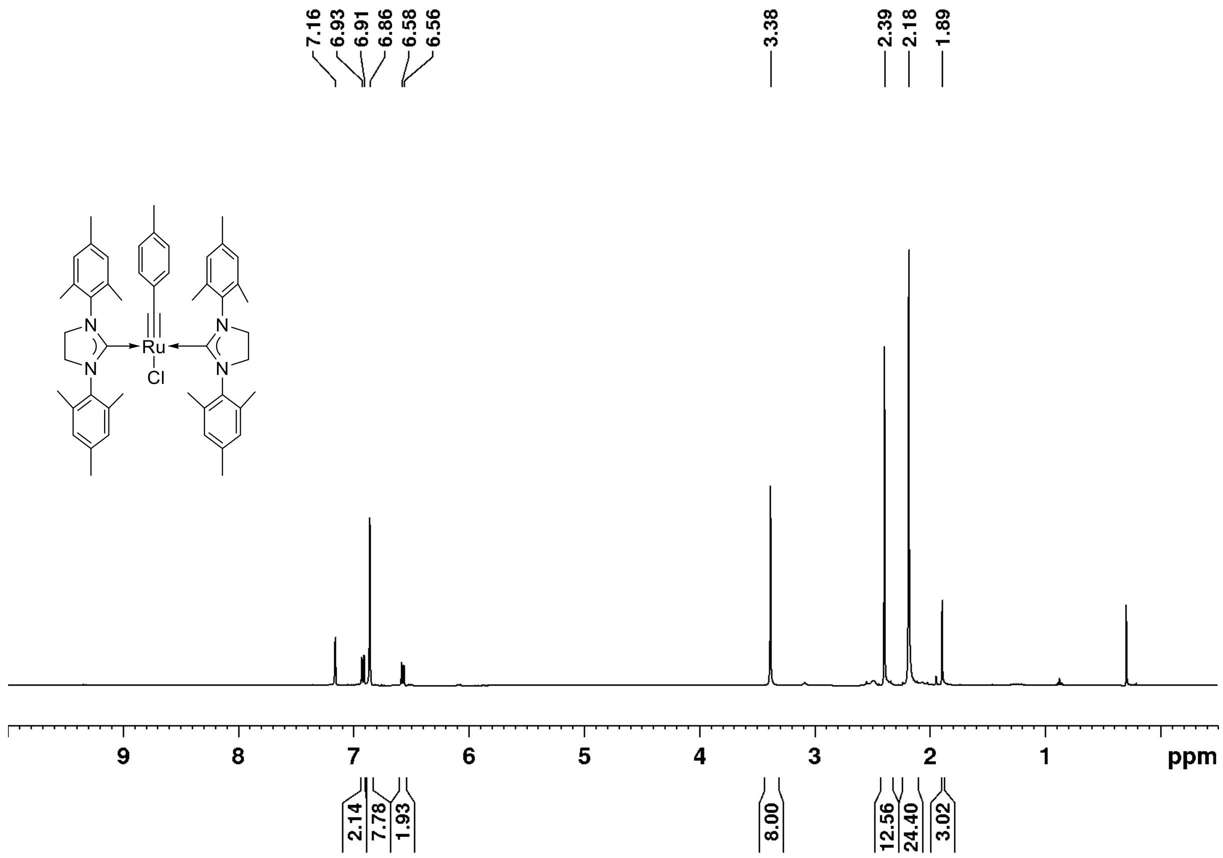

^{1}H NMR spectrum of complex **27** in C_6D_6 at 400 MHz

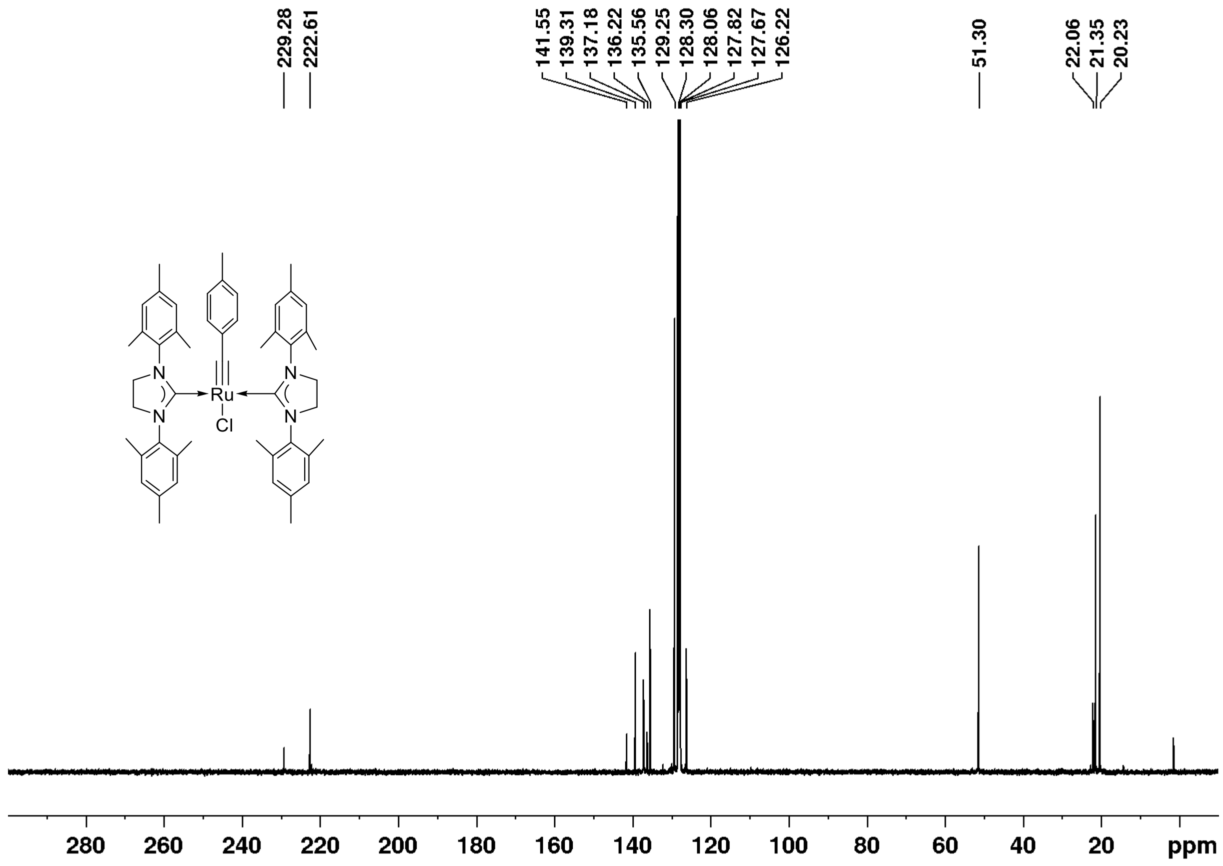

$^{13}C\{^{1}H\}$ NMR spectrum of complex **27** in C_6D_6 at 101 MHz

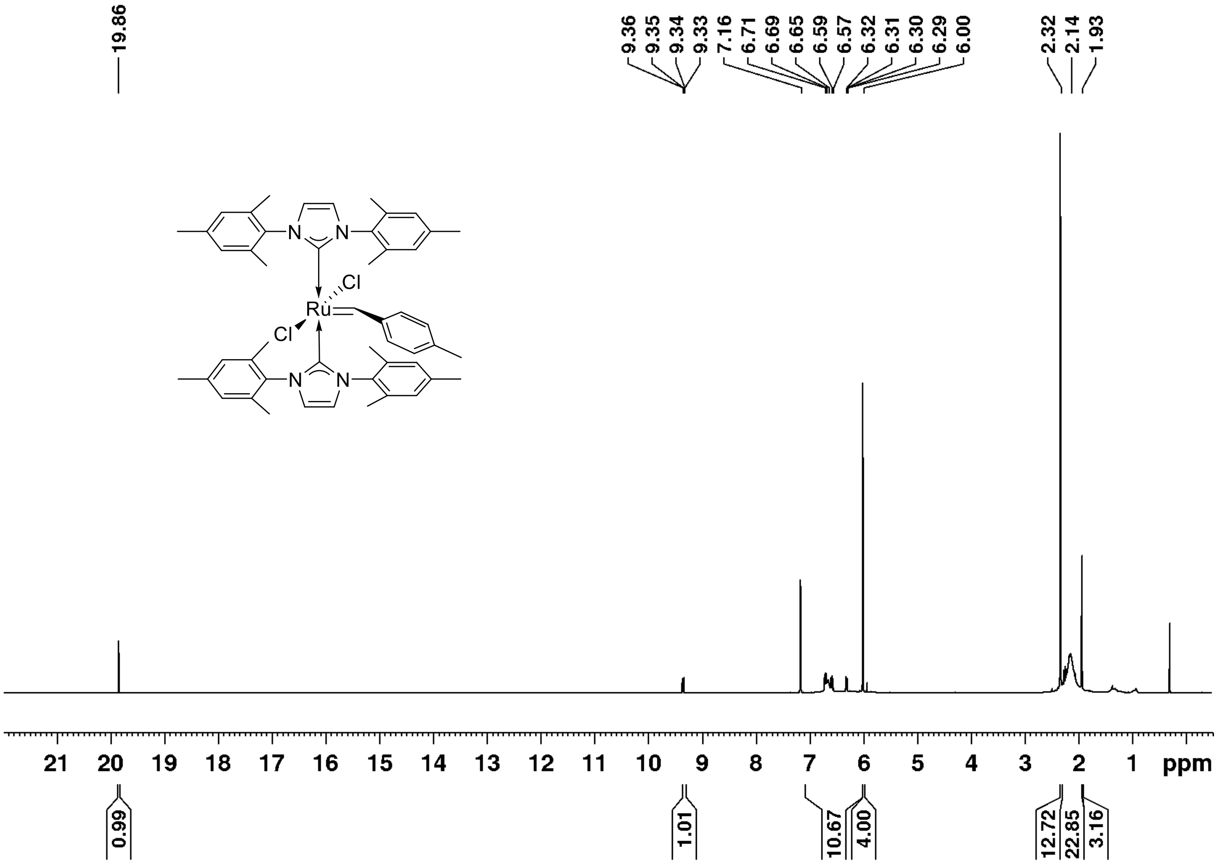

^{1}H NMR spectrum of complex **28** in C_6D_6 at 400 MHz

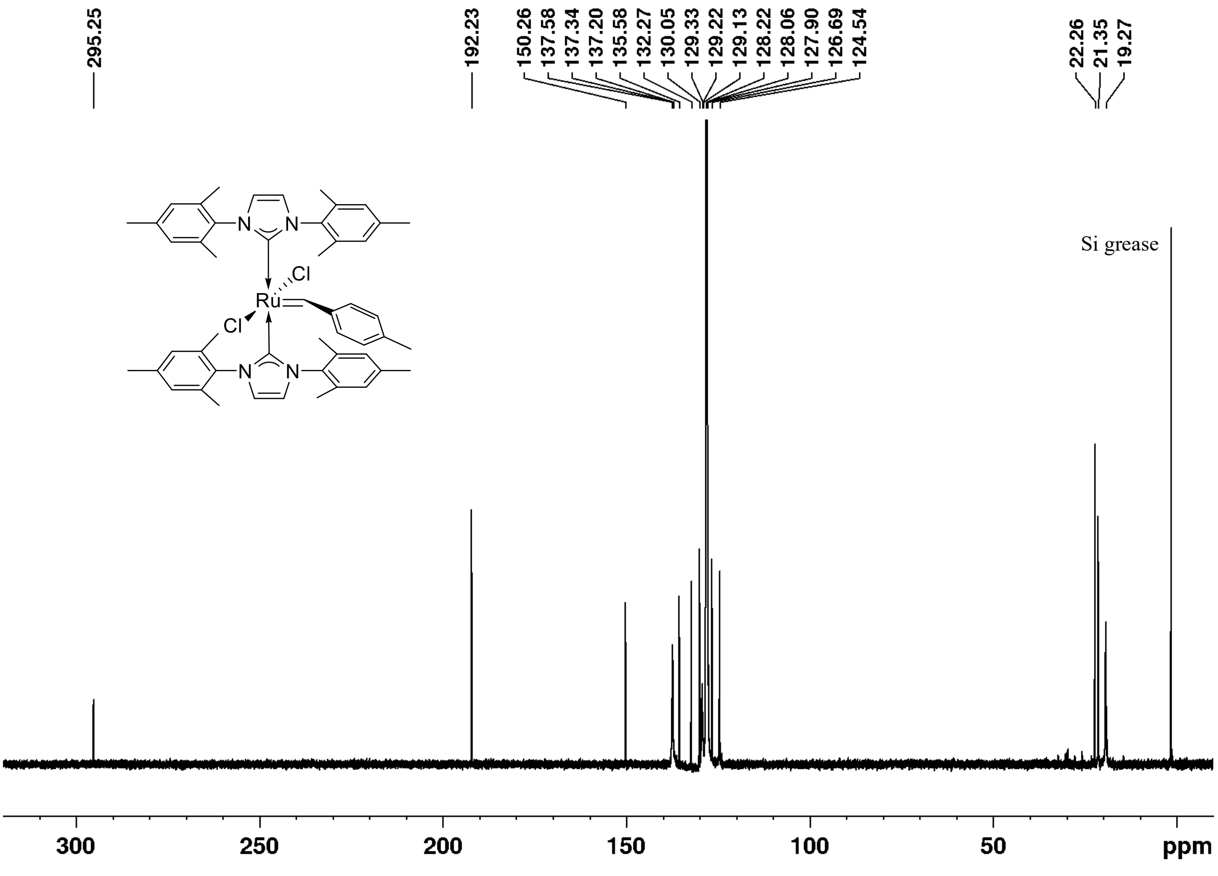

^{13}C{^{1}H} NMR spectrum of complex **28** in C_6D_6 at 151 MHz

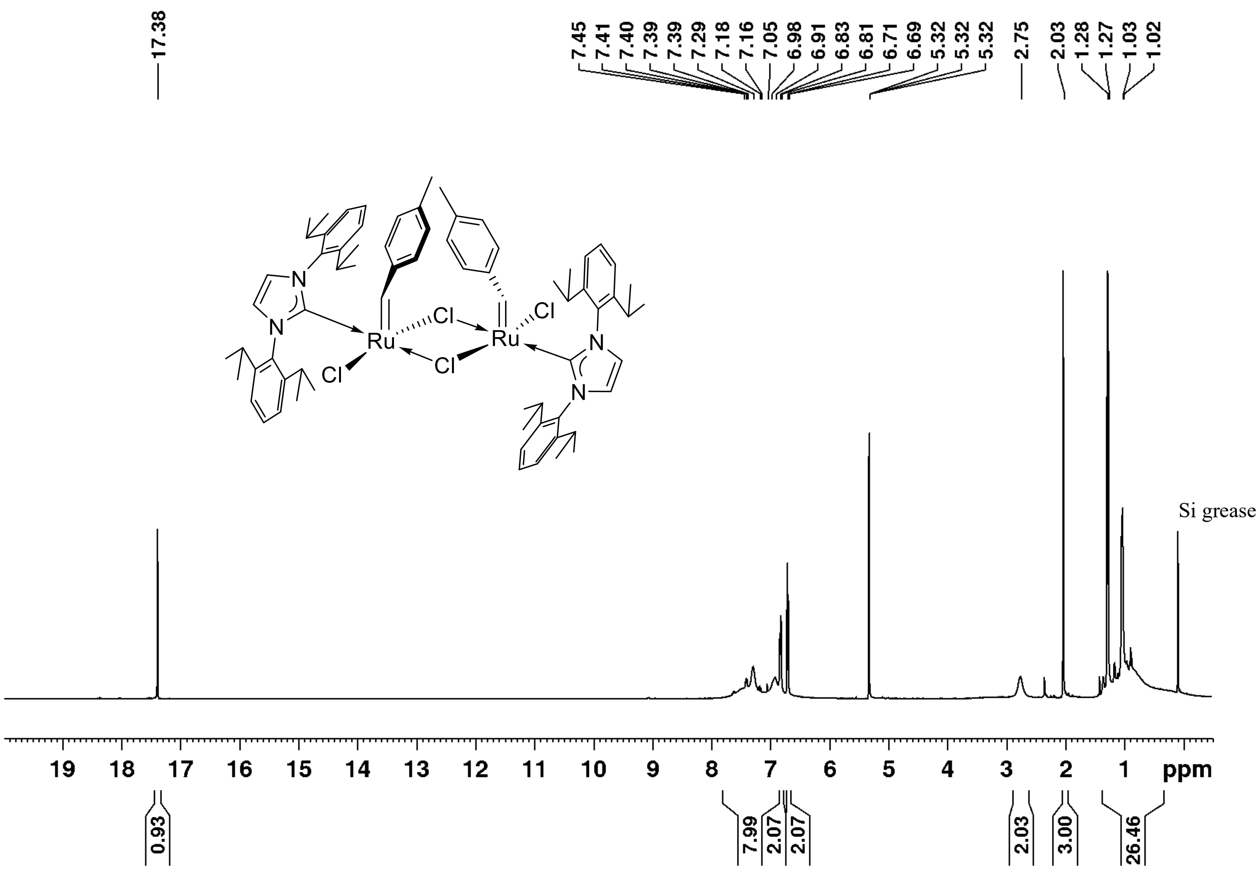

^{1}H NMR spectrum of complex **[29]2** in CD_2Cl_2 at 400 MHz at 298 K.

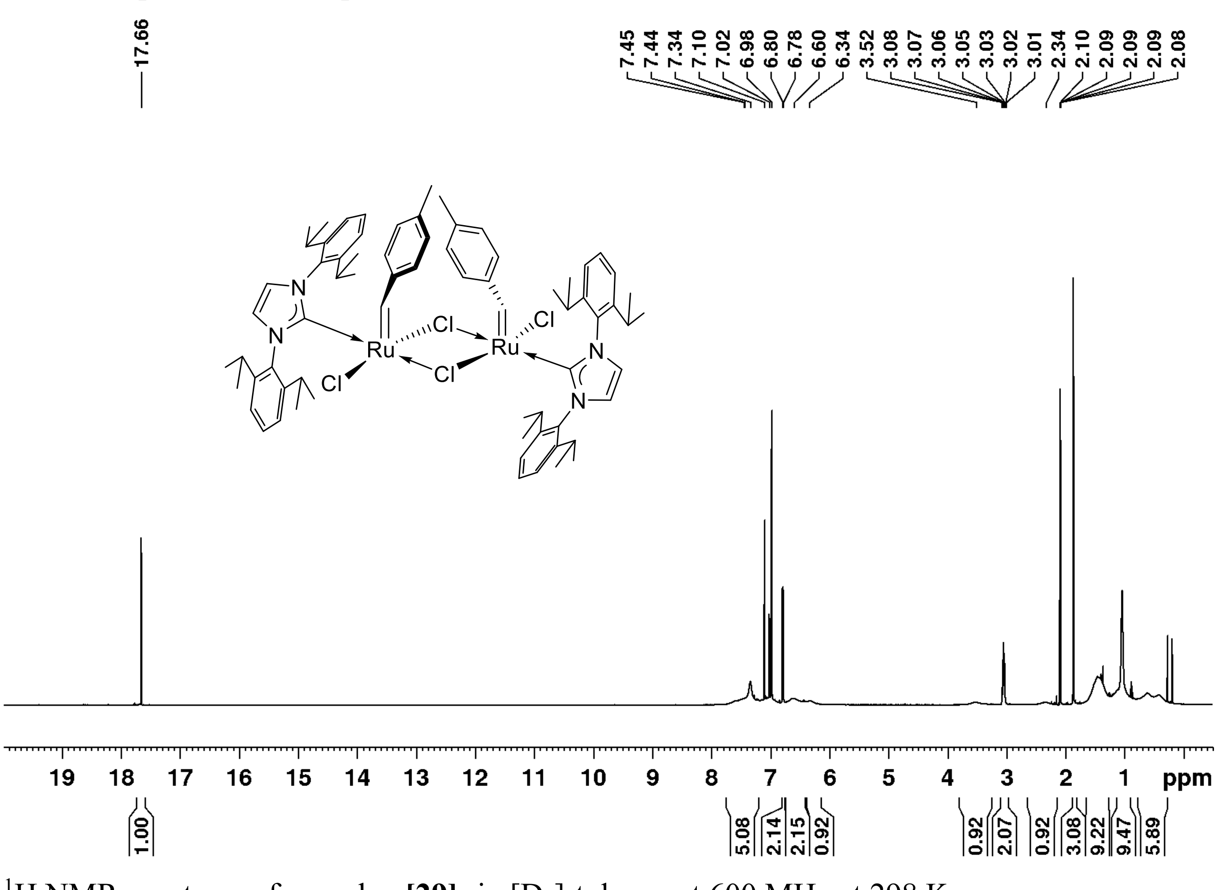

^{1}H NMR spectrum of complex **[29]2** in [D_8]-toluene at 600 MHz at 298 K.

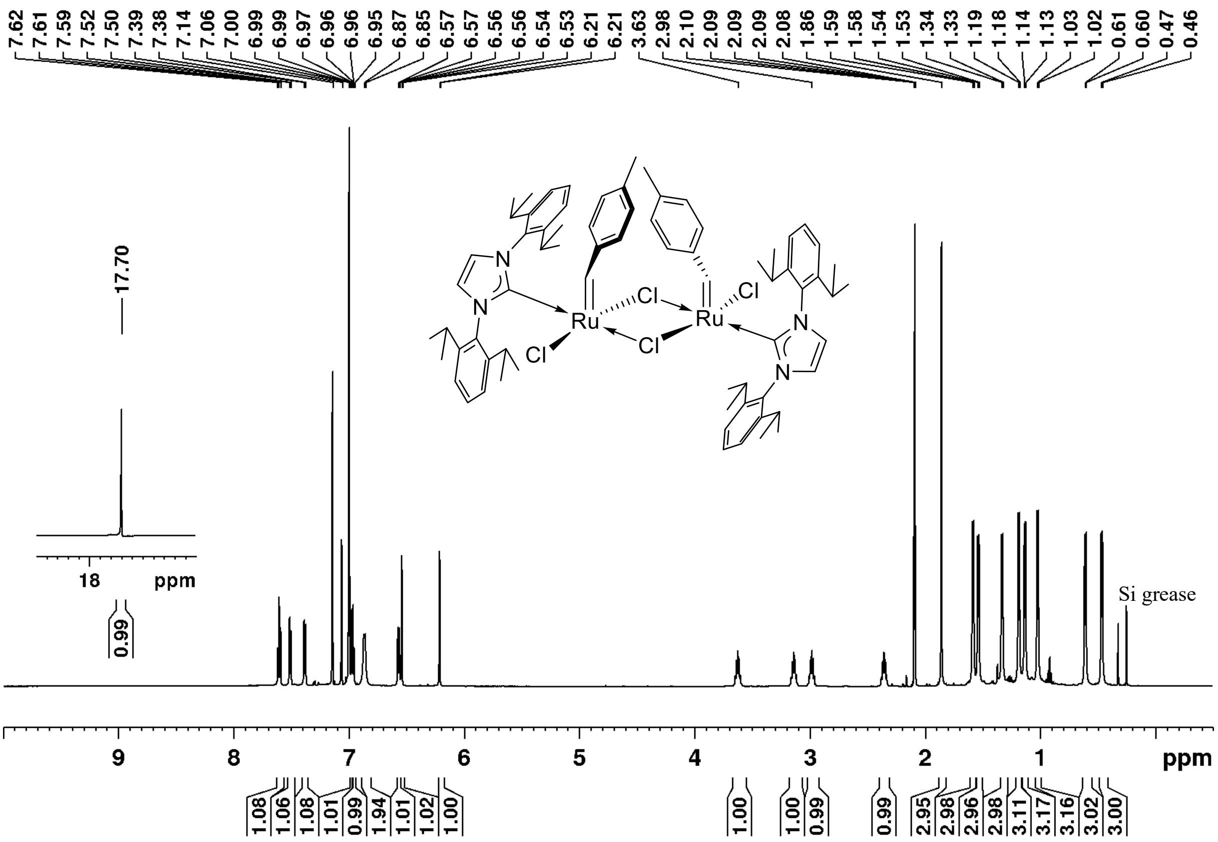

^{1}H NMR spectrum of complex **[29]₂** in [D₈]-toluene at 600 MHz at 233 K.

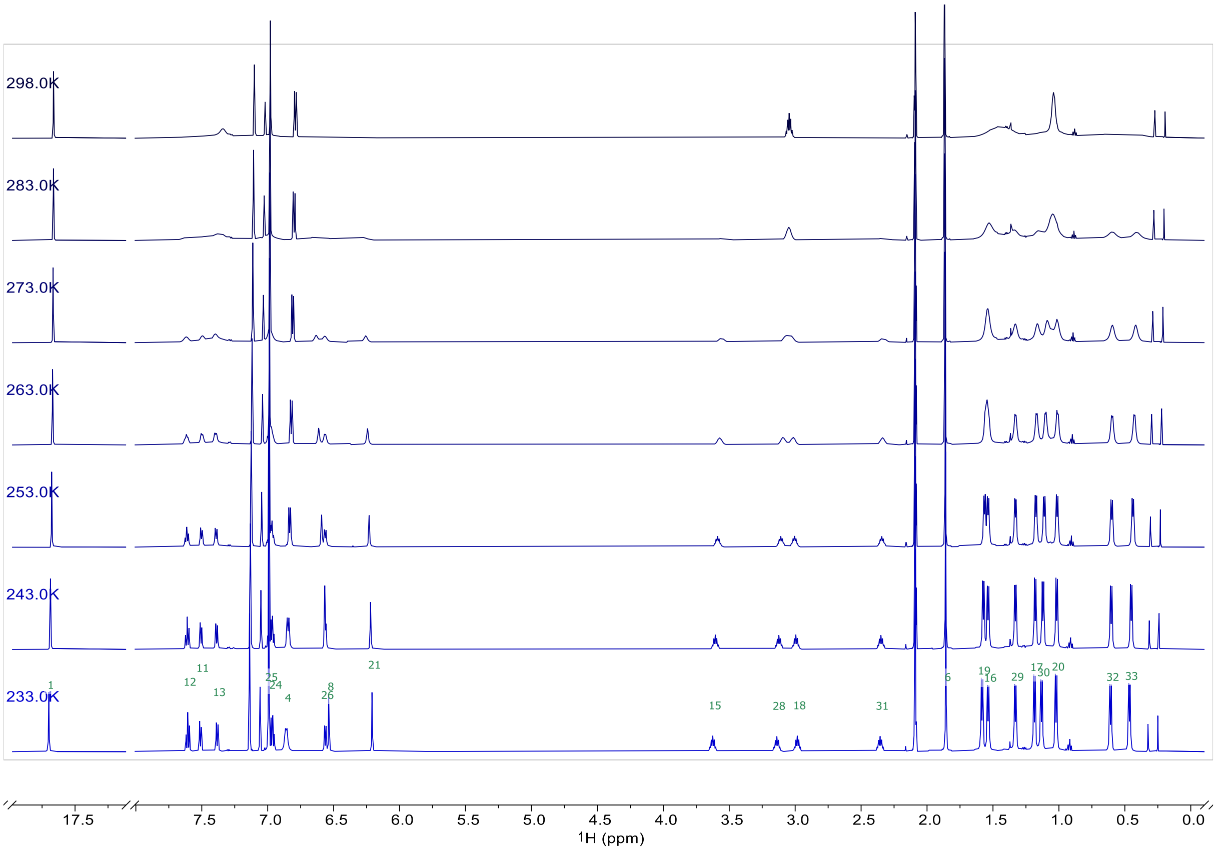

Variable Temperature ^{1}H NMR spectra of complex **[29]₂** in [D₈]-toluene at 600 MHz

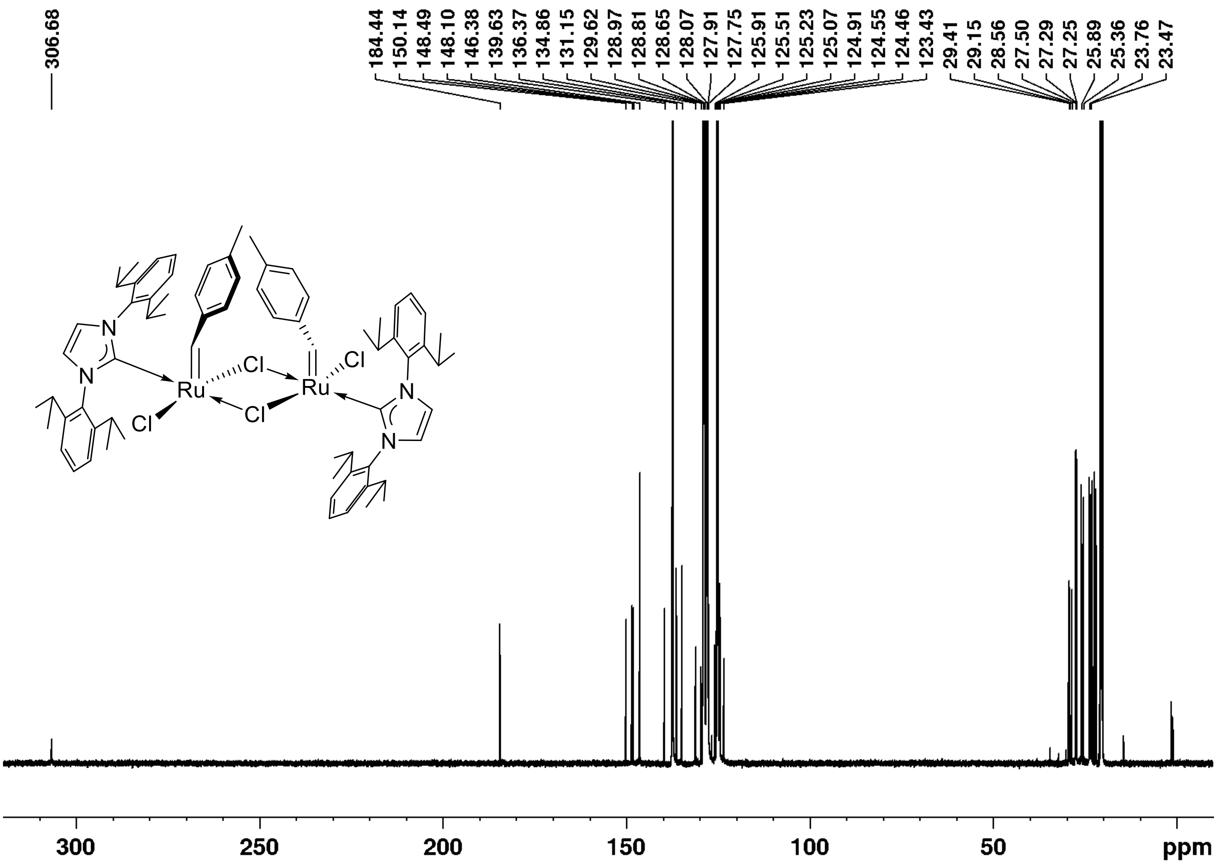

$^{13}C\{^{1}H\}$ NMR spectrum of complex **[29]$_2$** in [D$_8$]-toluene at 151 MHz at 233 K.

DOSY experiment. A DOSY experiment was carried out with a solution of complex **[29]$_2$** in [D$_8$]-toluene. After the measurement, excess pyridine was added to the NMR tube to generate the pyridine adduct **33a** *in situ*. For comparison, a DOSY experiment of this pyridine adduct was also carried out.

After addition of pyridine to the sample, the signals shifted significantly and the self-diffusion coefficient D became significantly faster (D = 6.57 × 10 -10 m²/s vs 7.34 × 10 -10 m²/s), which suggests that the starting complex is a dimeric entity.

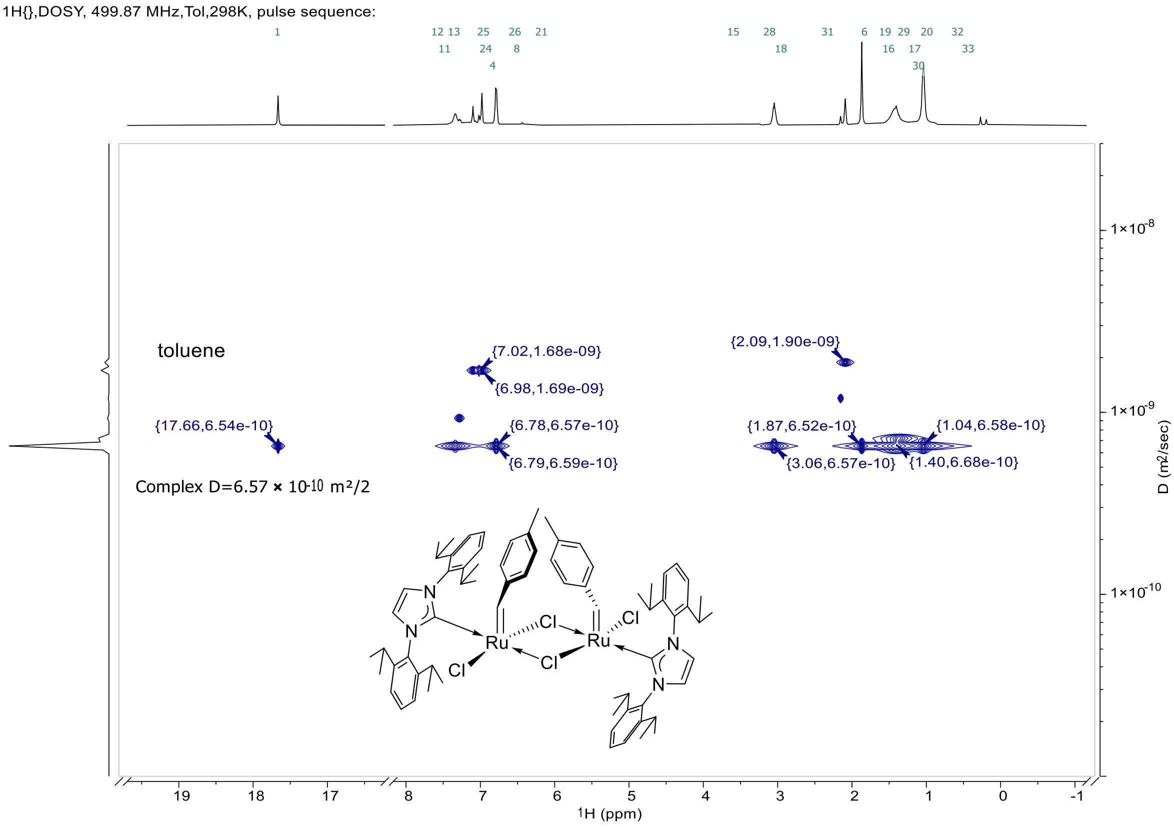

^{1}H DOSY NMR spectrum of dimeric Ru carbene complex **[29]$_2$** in [D$_8$]-toluene at 500 MHz at 298 K.

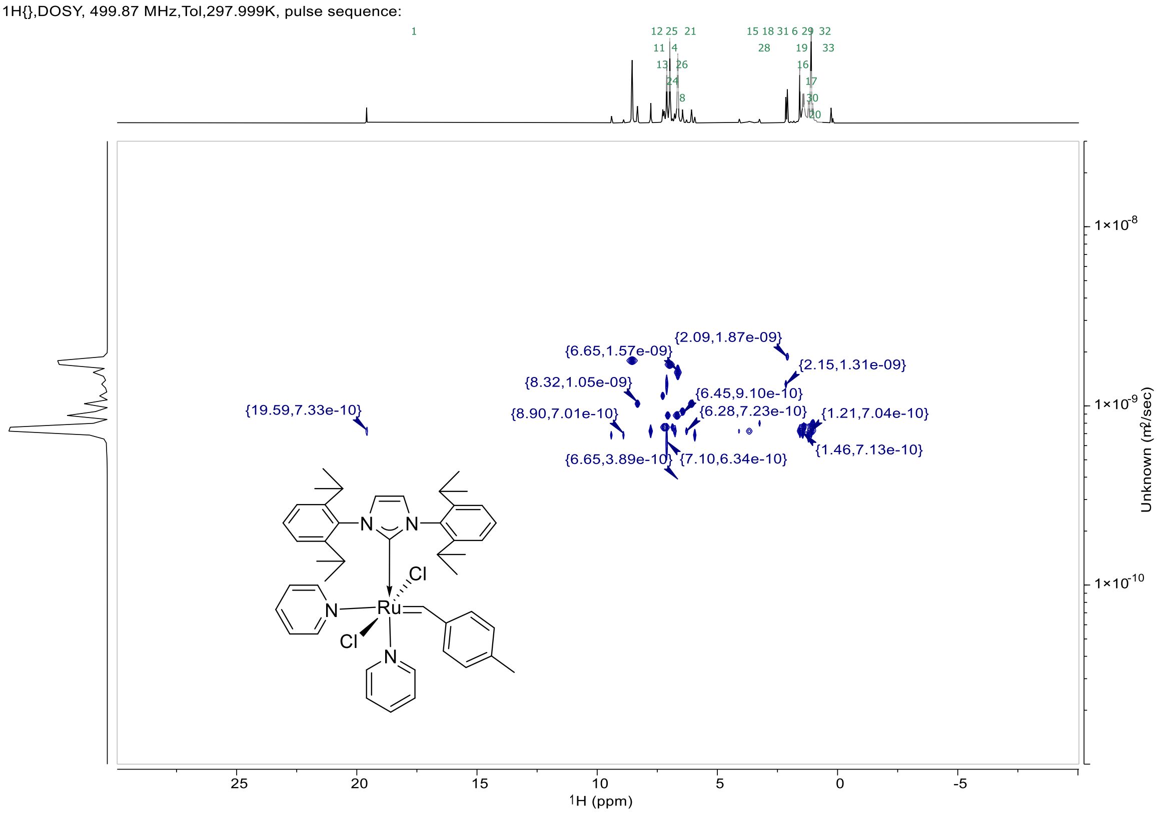

^{1}H DOSY NMR spectrum of Ru carbene-pyridine complex **33a** generated in situ from complex **[29]$_2$** and excess pyridine in [D$_8$]-toluene at 500 MHz at 298 K.

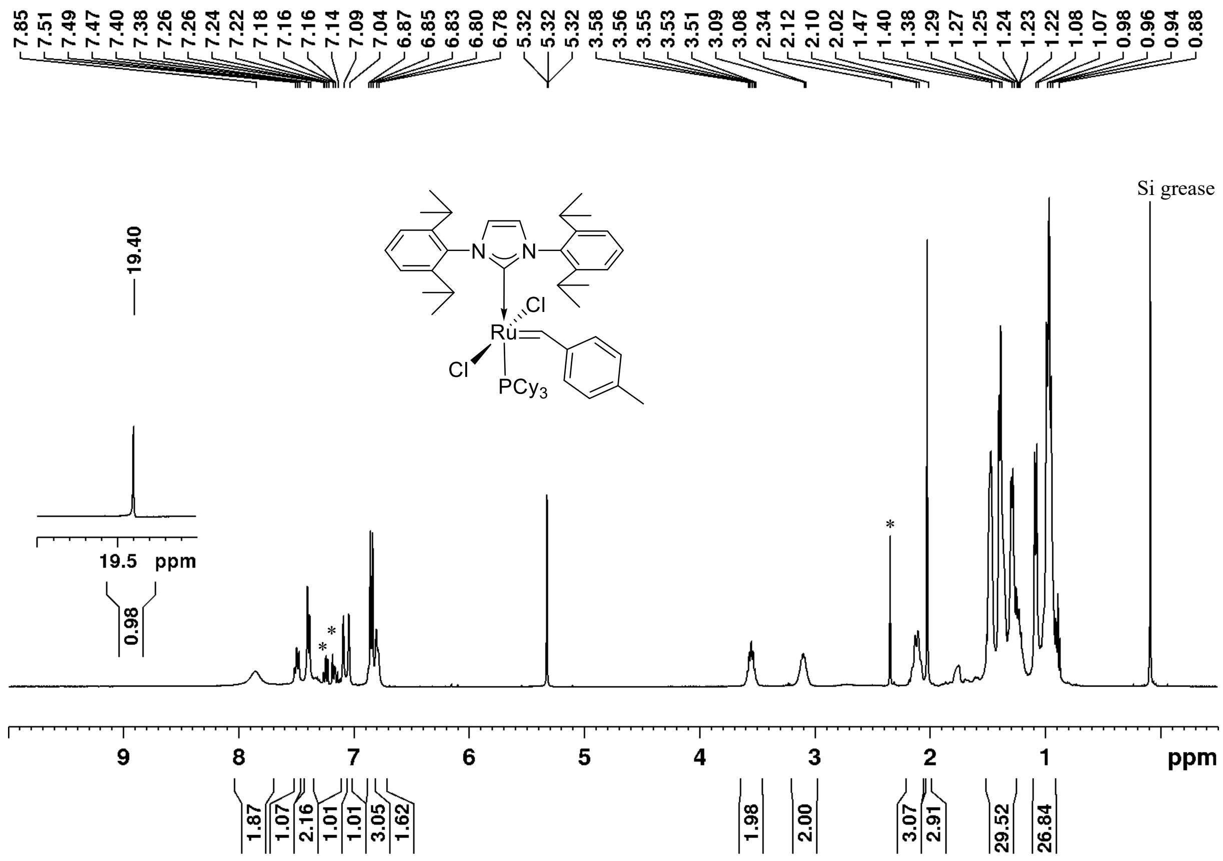

^{1}H NMR spectrum of complex **31** in CD_2Cl_2 at 400 MHz. * toluene

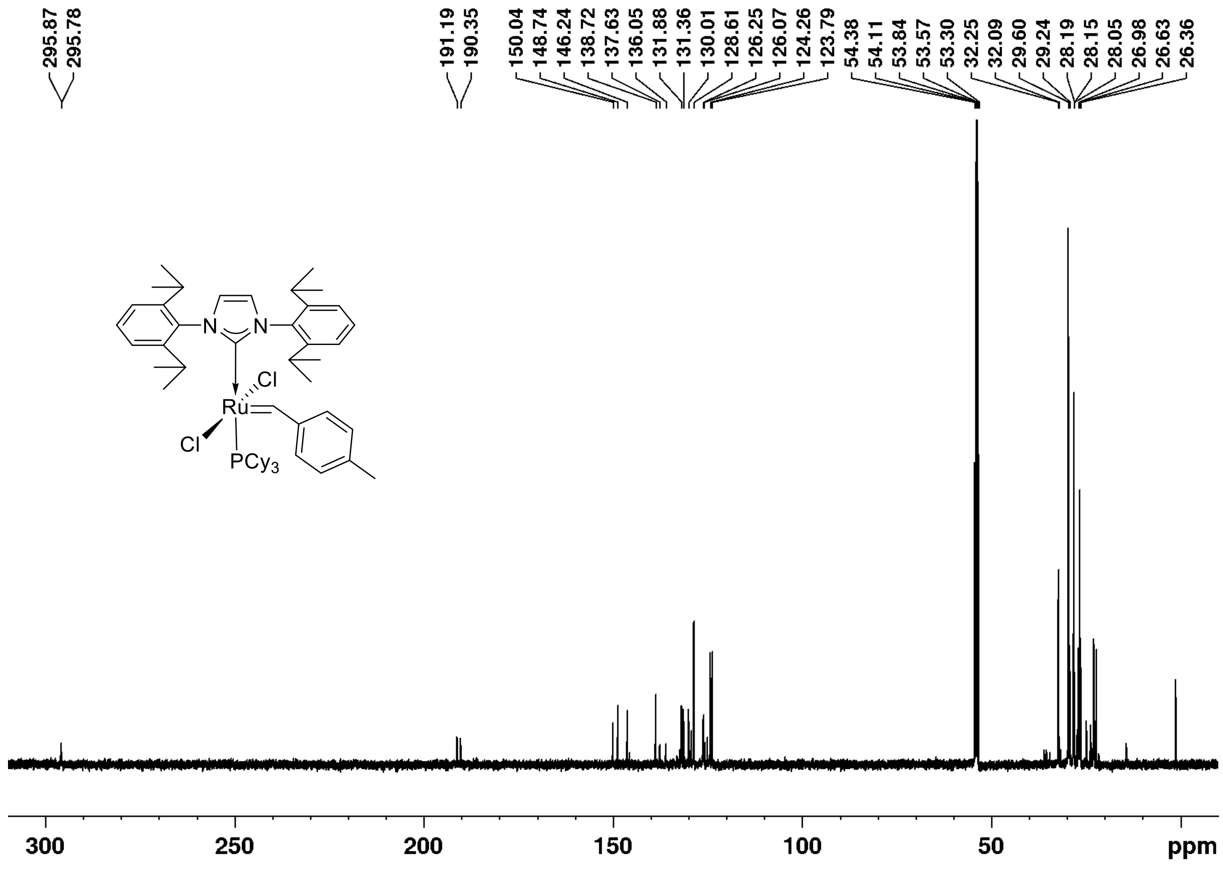

^{13}C{^{1}H} NMR spectrum of complex **31** in CD_2Cl_2 at 101 MHz

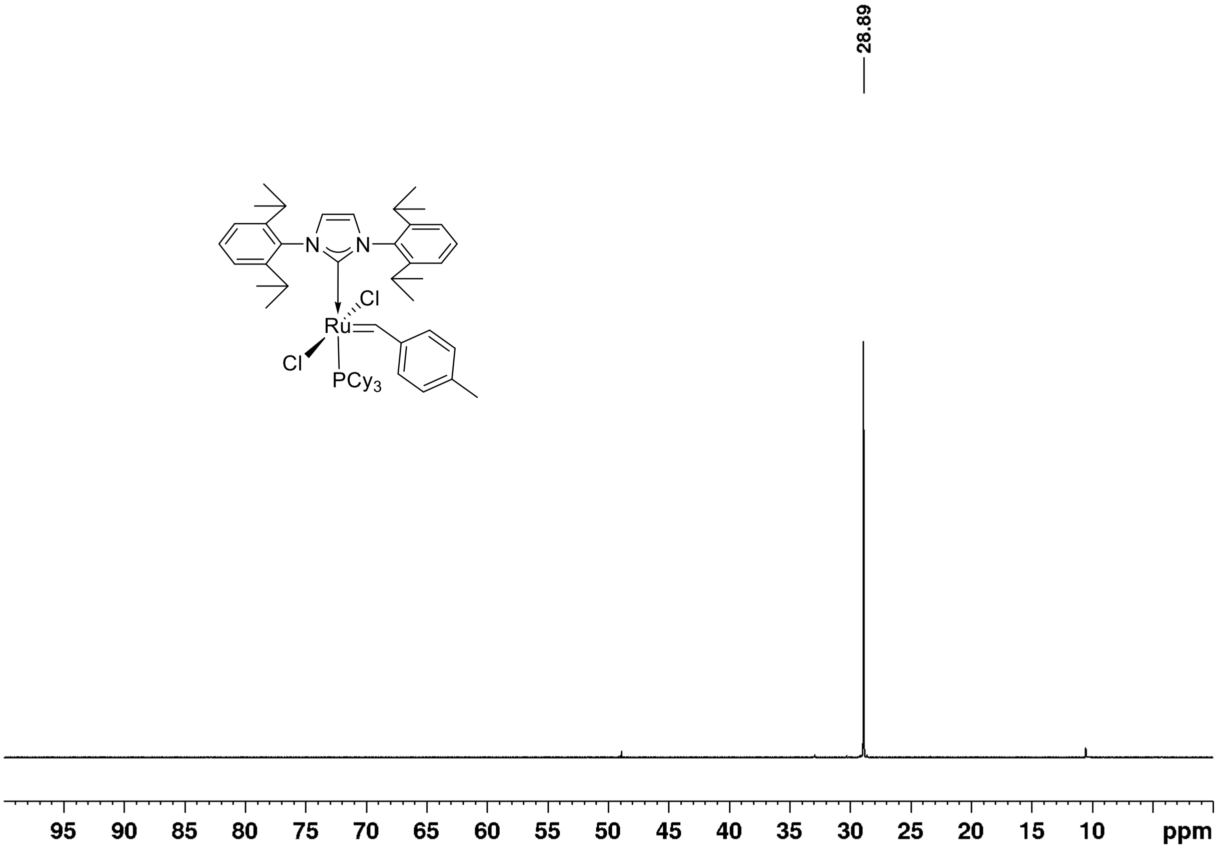

$^{31}P\{^{1}H\}$ NMR spectrum of complex **31** in CD_2Cl_2 at 162 MHz

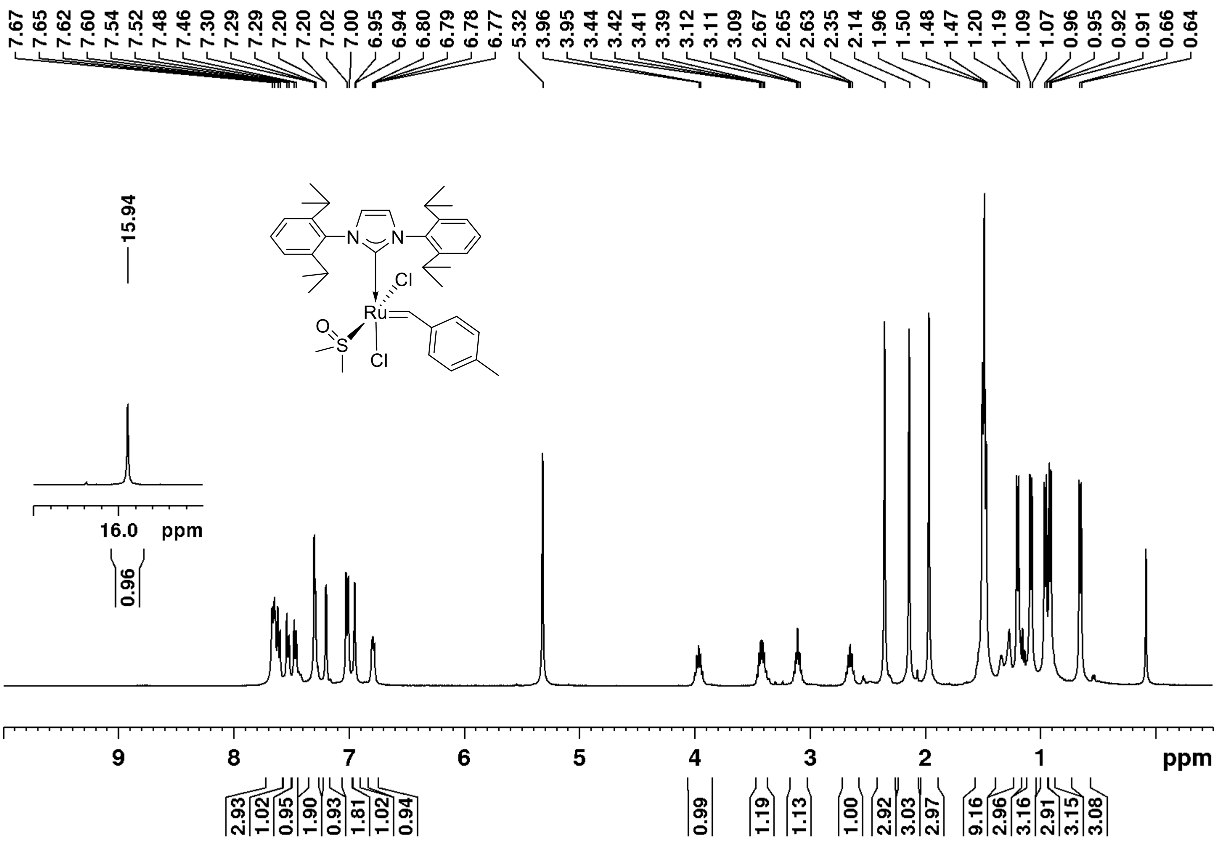

^{1}H NMR spectrum of complex **32** in CD_2Cl_2 at 400 MHz

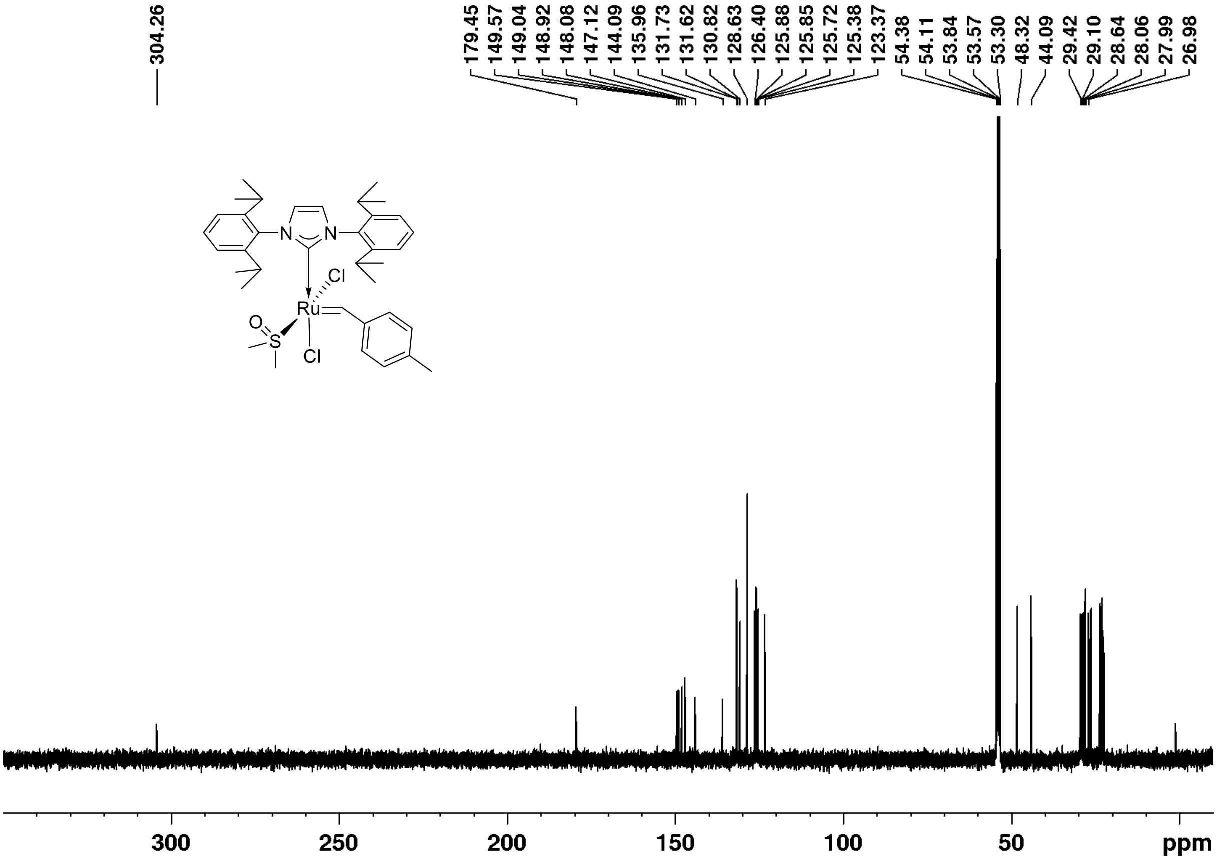

$^{13}C\{^1H\}$ NMR spectrum of complex **32** in CD_2Cl_2 at 101 MHz

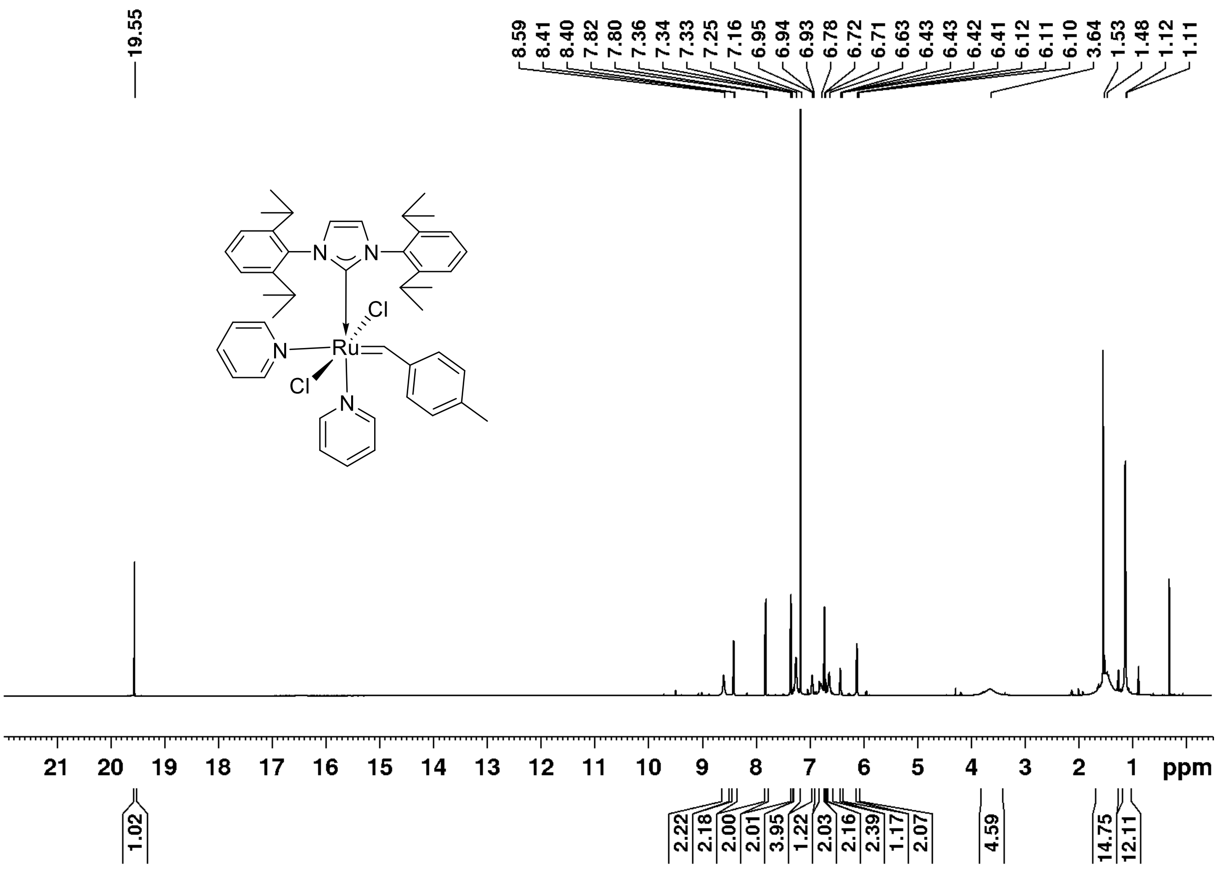

^{1}H NMR spectrum of complex **33a** in C_6D_6 at 600 MHz

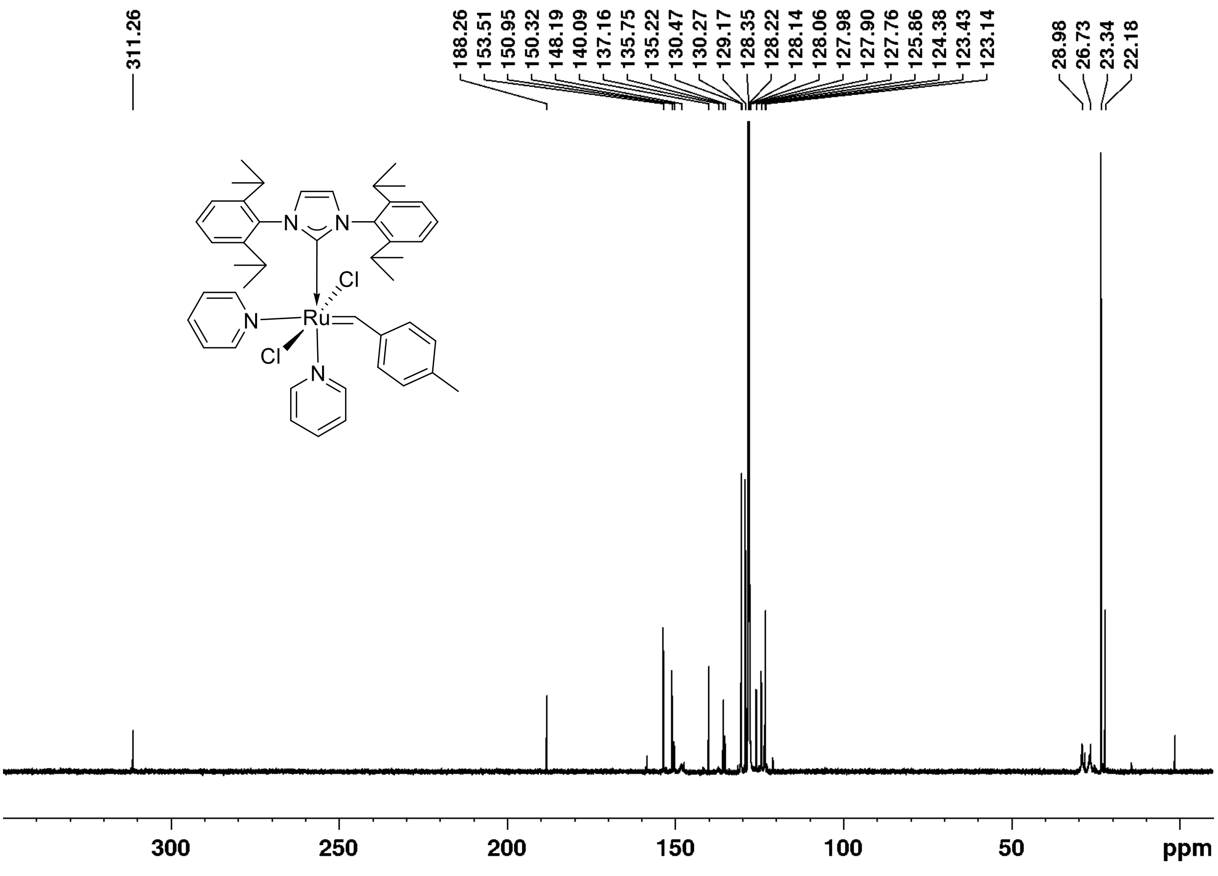

^{13}C{^{1}H} NMR spectrum of complex **33a** in C_6D_6 at 151 MHz

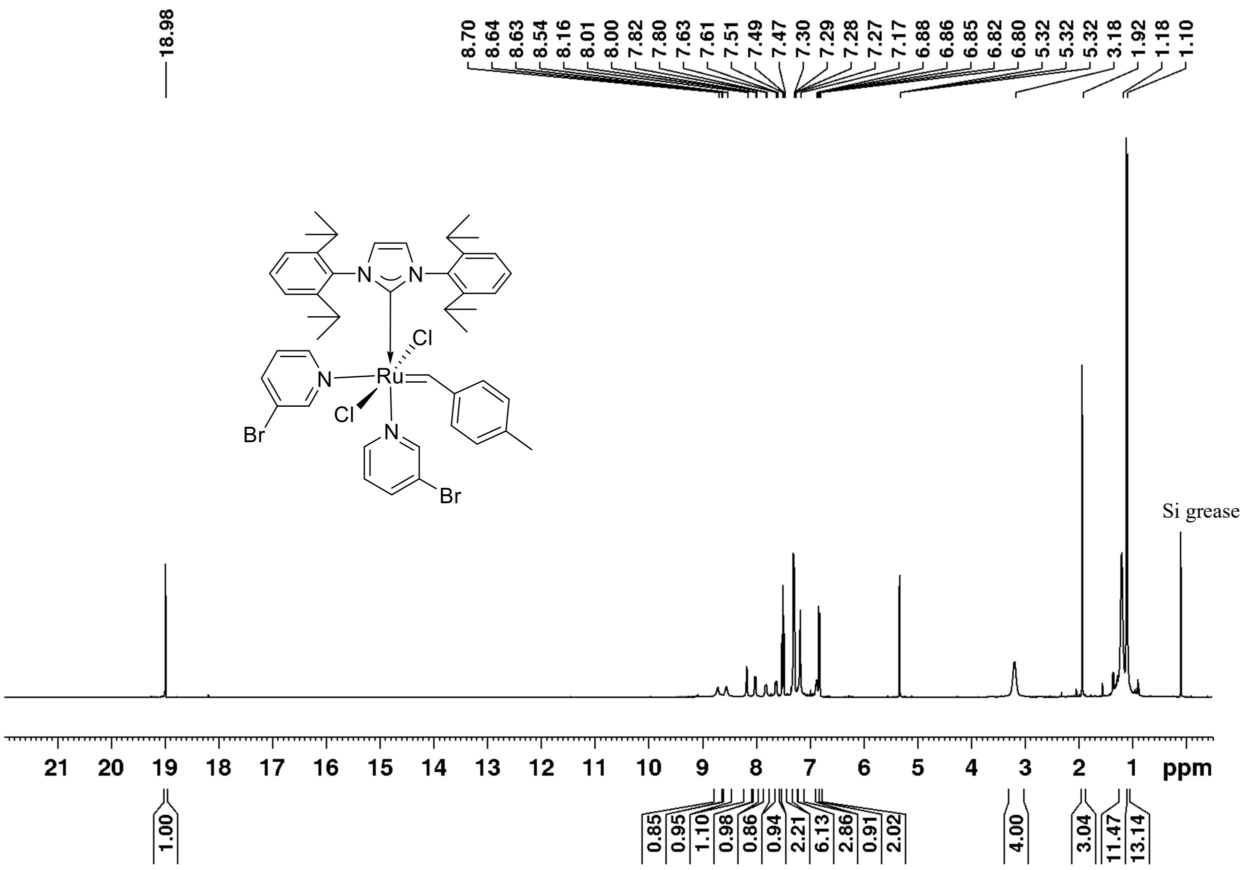

^1H NMR spectrum of complex **33b** in CD_2Cl_2 at 400 MHz

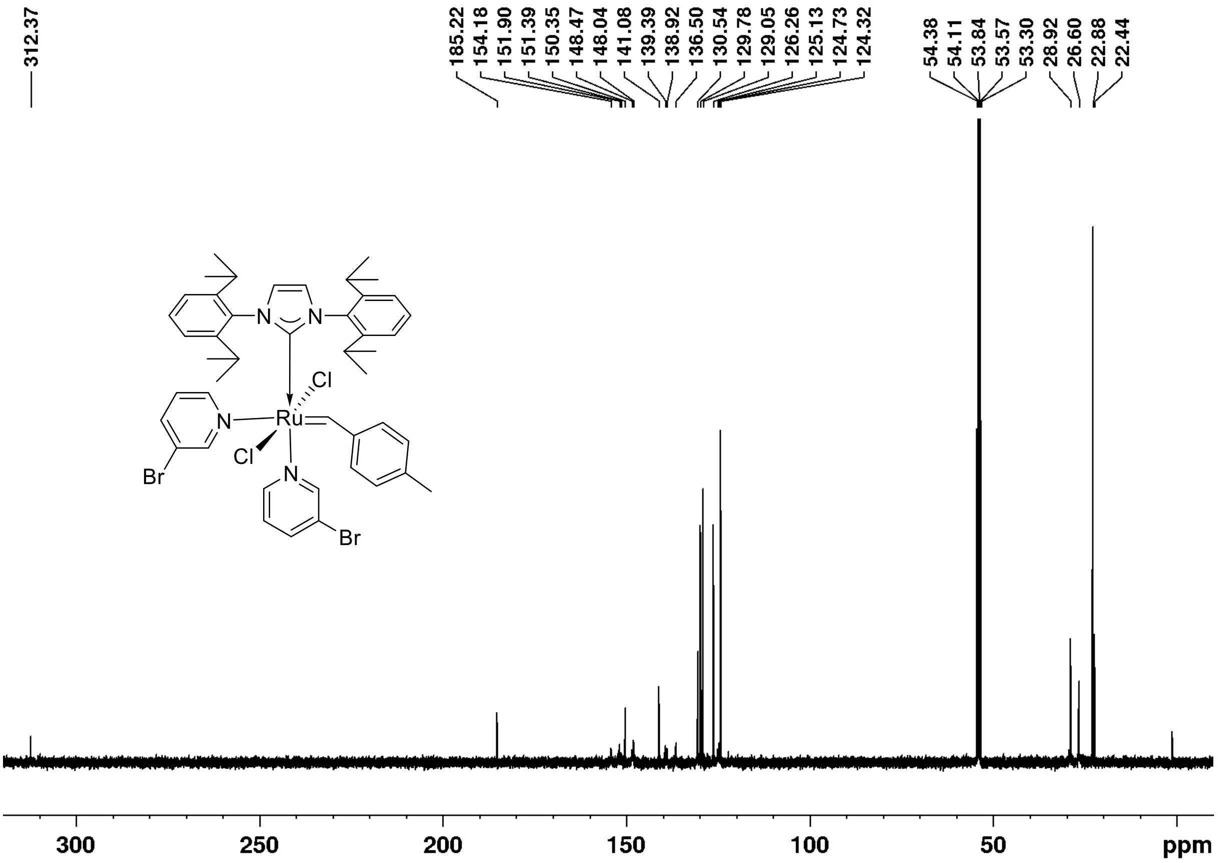

$^{13}C\{^1H\}$ NMR spectrum of complex **33b** in CD_2Cl_2 at 101 MHz

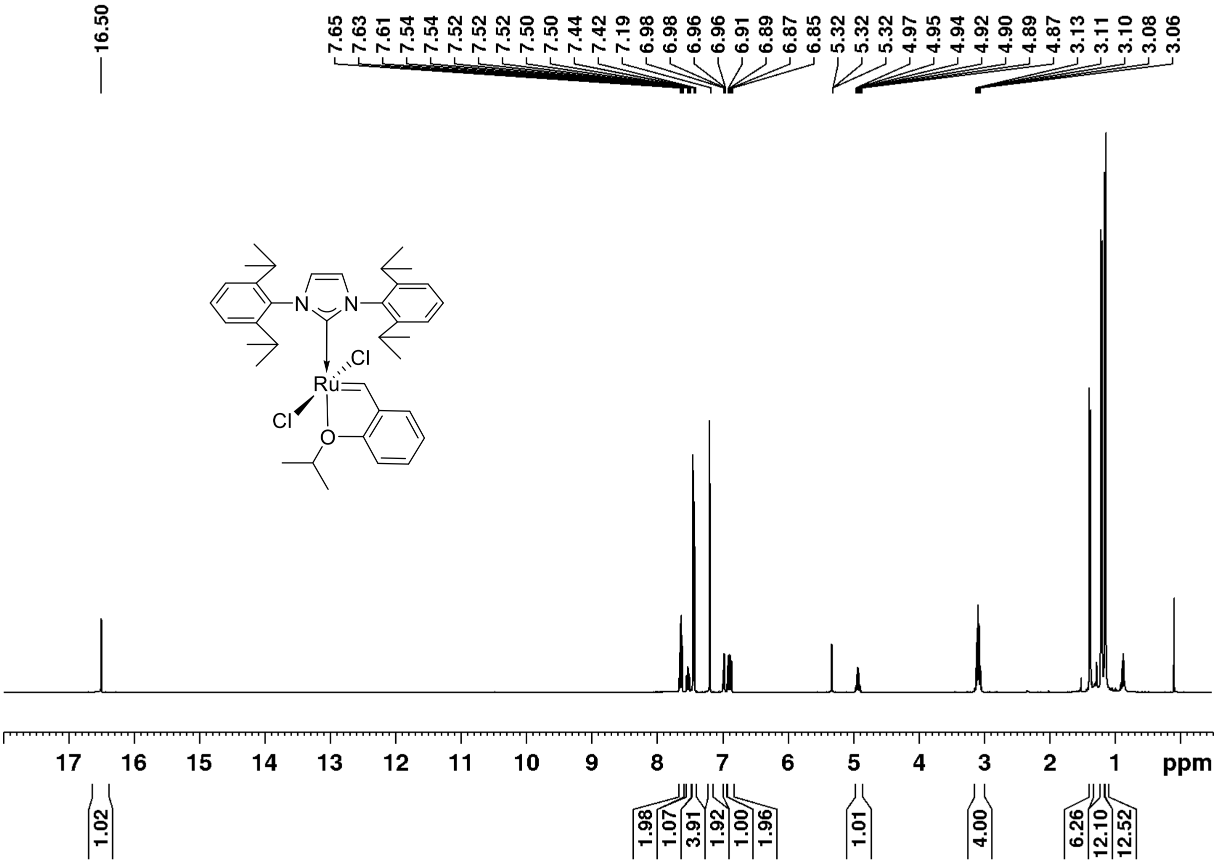

^{1}H NMR spectrum of complex **34a** in CD_2Cl_2 at 400 MHz

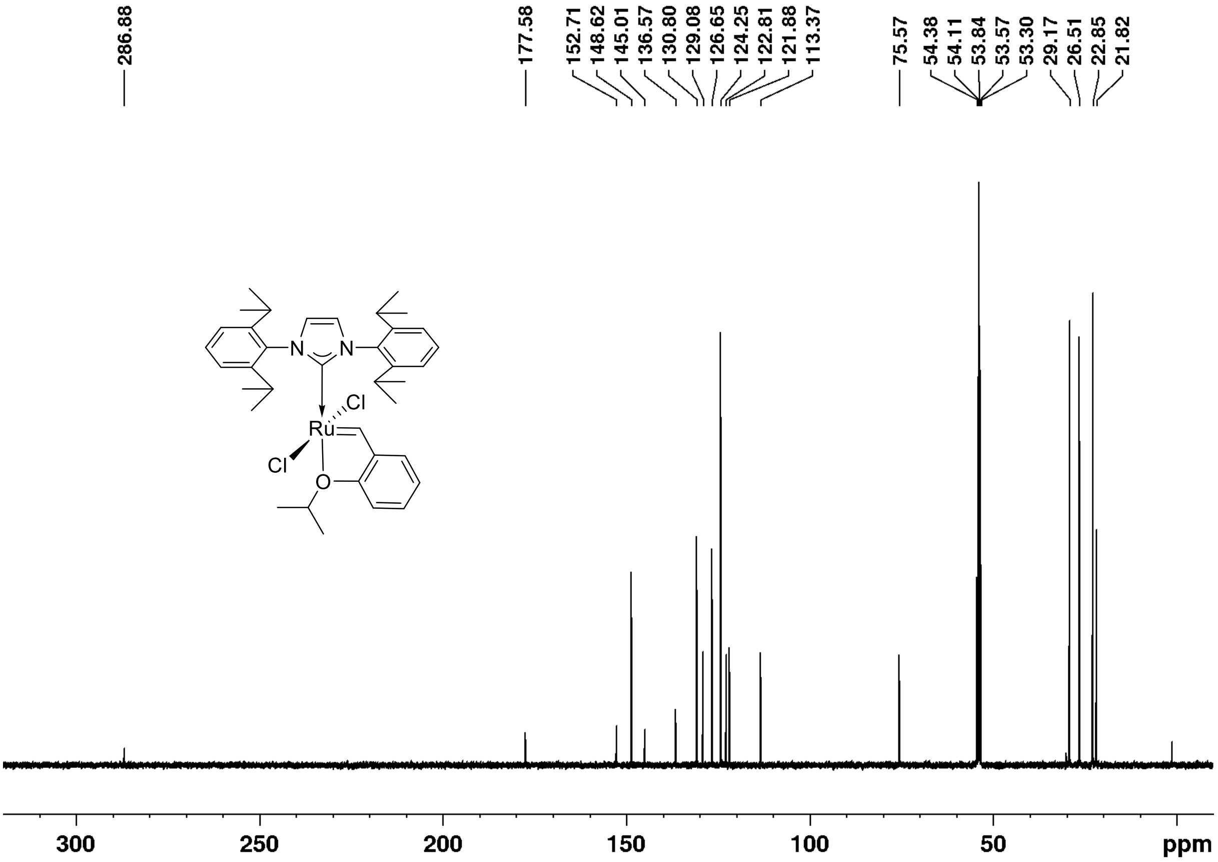

$^{13}C\{^{1}H\}$ NMR spectrum of complex **34a** in CD_2Cl_2 at 101 MHz

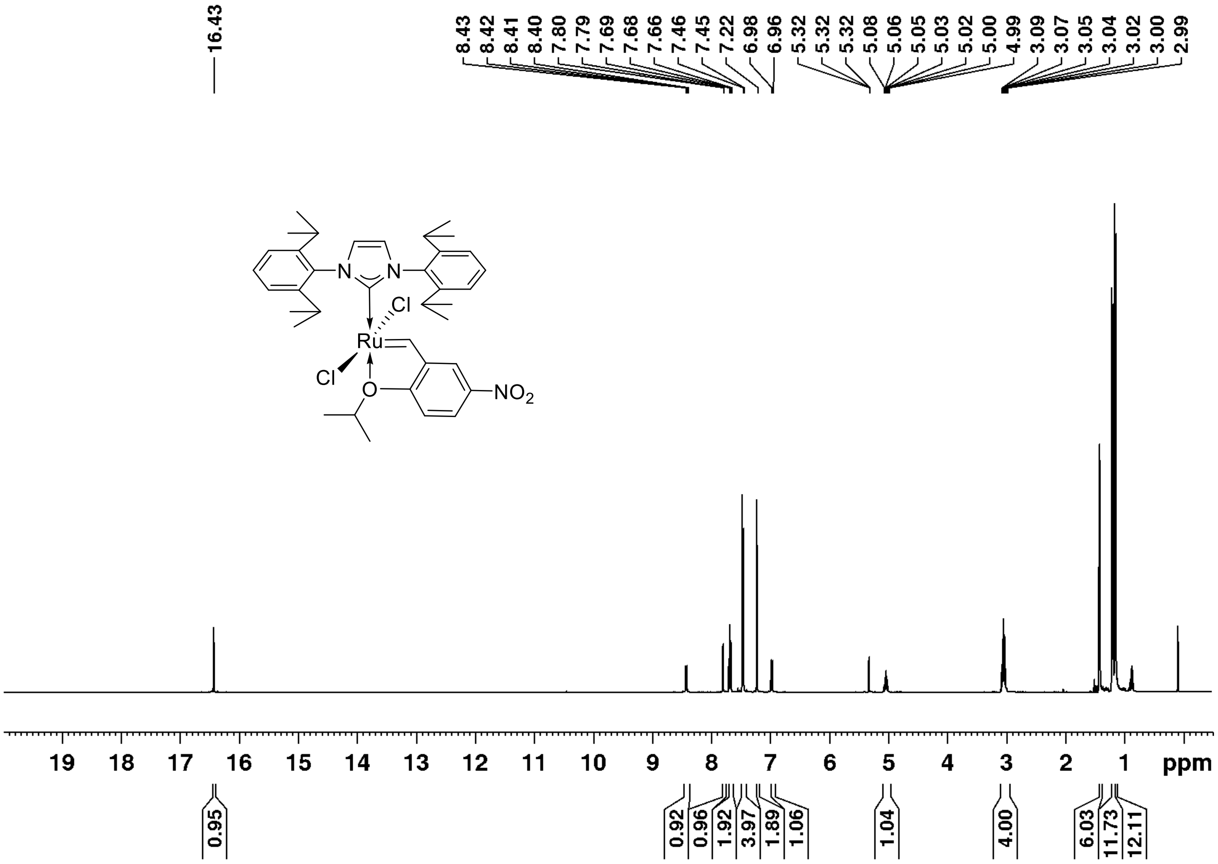

^{1}H NMR spectrum of complex **34b** in CD_2Cl_2 at 400 MHz

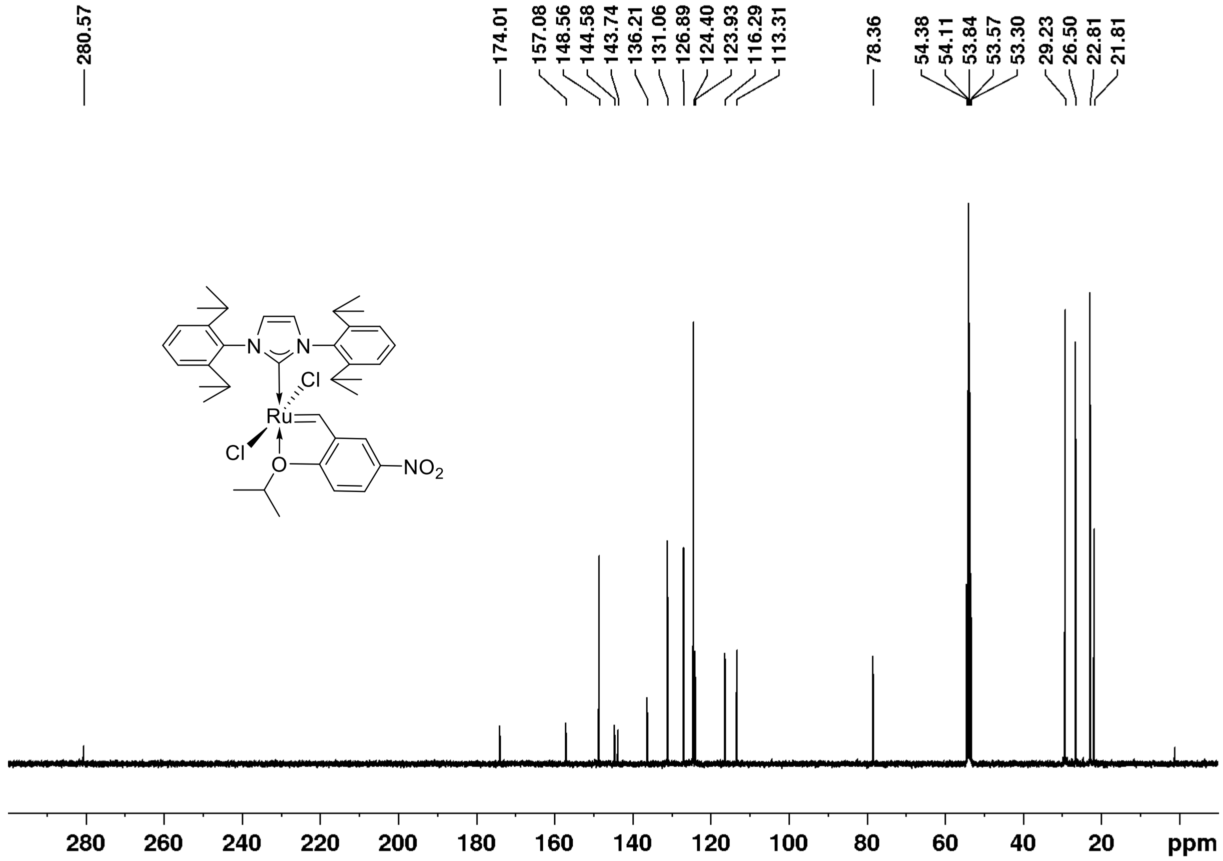

^{13}C{^{1}H} NMR spectrum of complex **34b** in CD_2Cl_2 at 101 MHz

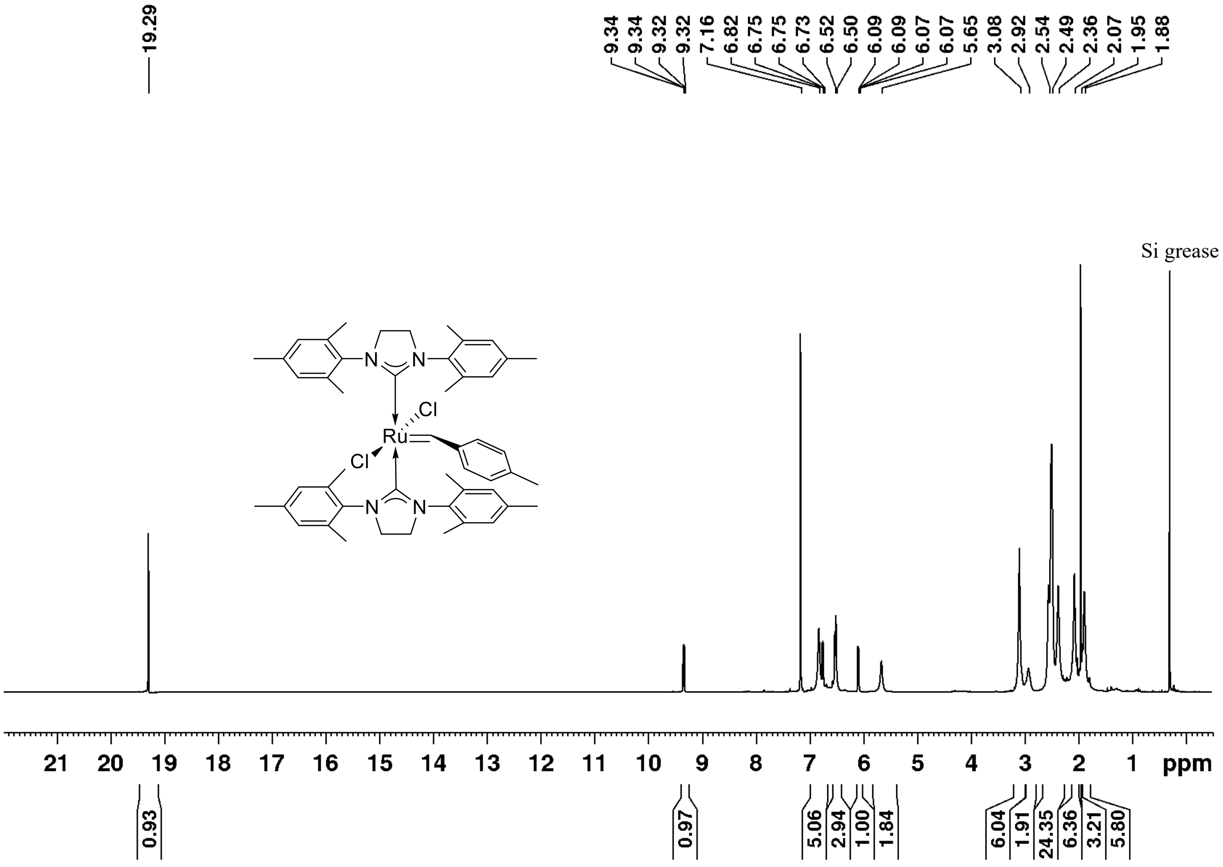

^{1}H NMR spectrum of complex **35** in C_6D_6 at 400 MHz

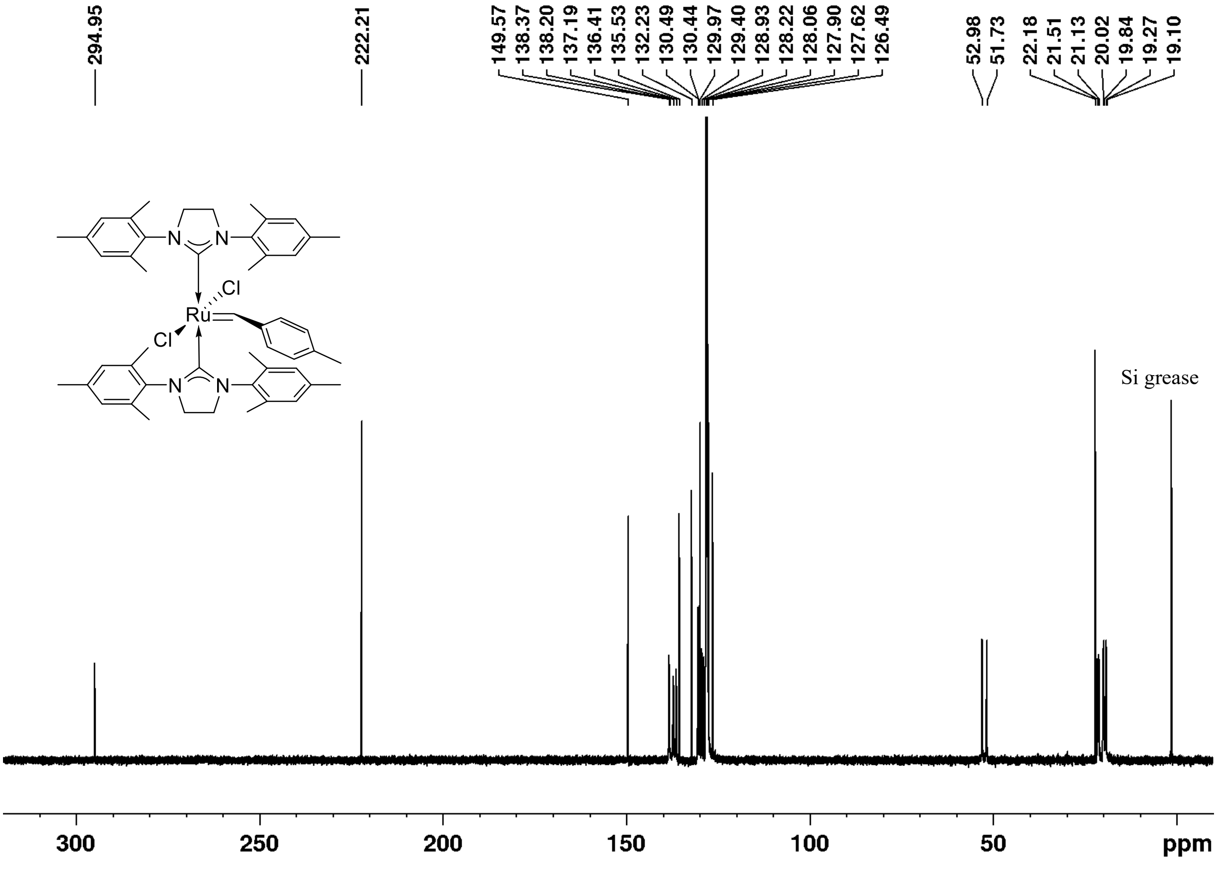

^{13}C{^{1}H} NMR spectrum of complex **35** in C_6D_6 at 151 MHz

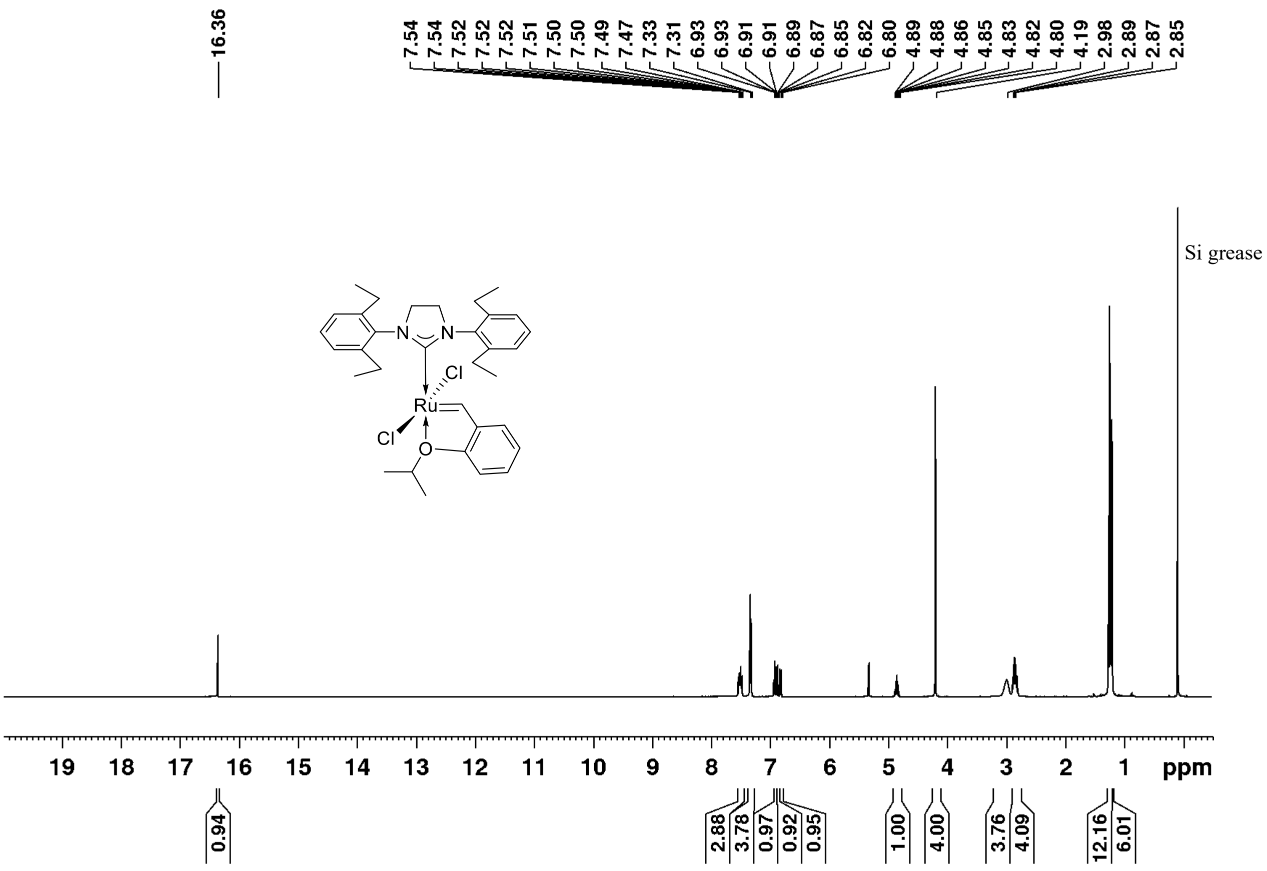

^{1}H NMR spectrum of complex **36** in CD_2Cl_2 at 400 MHz

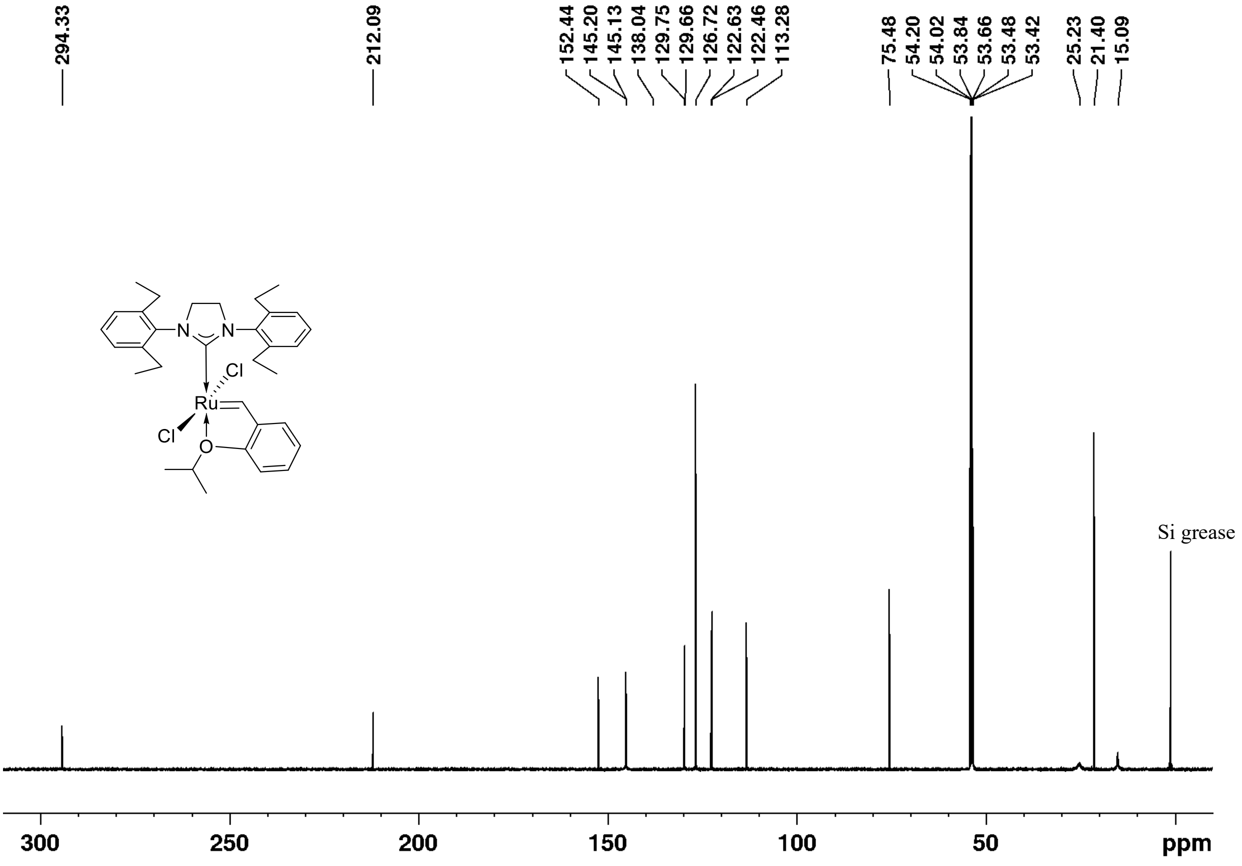

$^{13}C\{^1H\}$ NMR spectrum of complex **36** in CD_2Cl_2 at 151 MHz

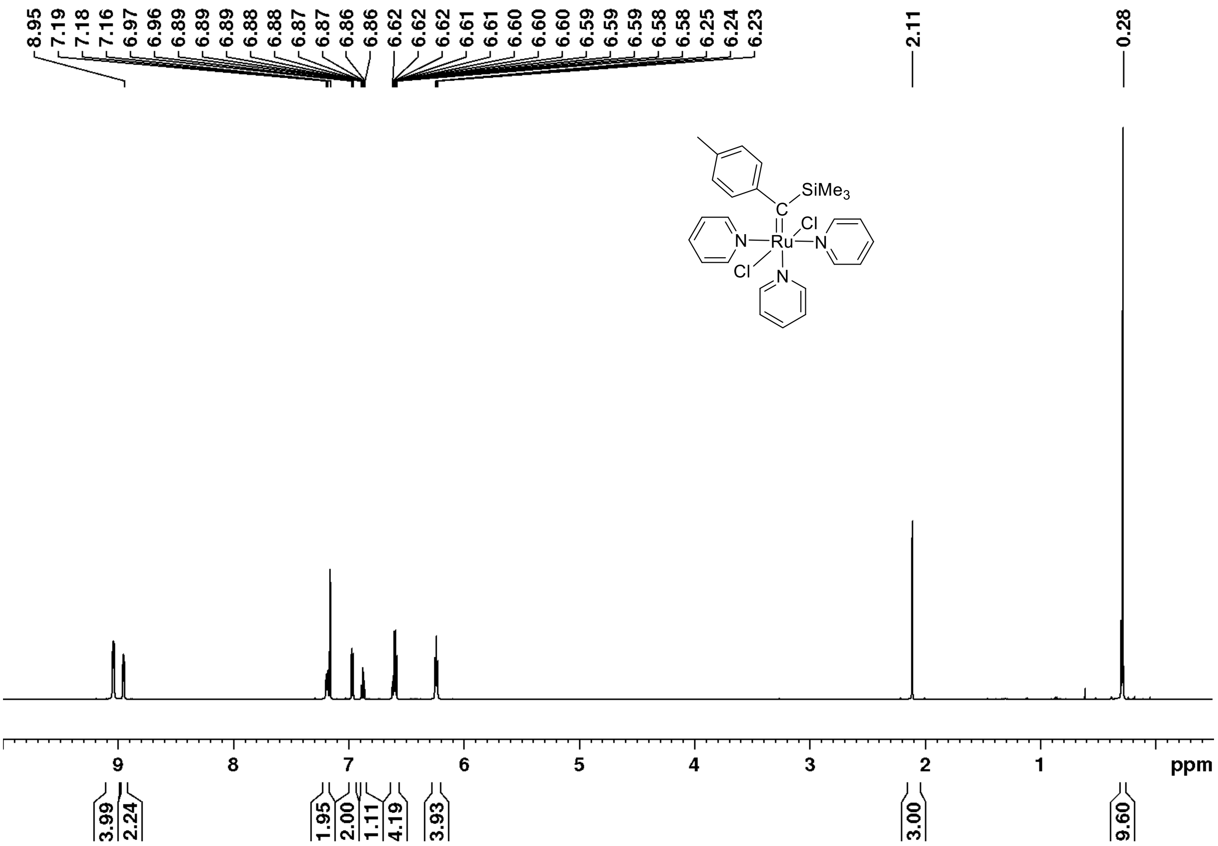

^{1}H NMR spectrum of complex **39** in C_6D_6 at 600 MHz at 298 K

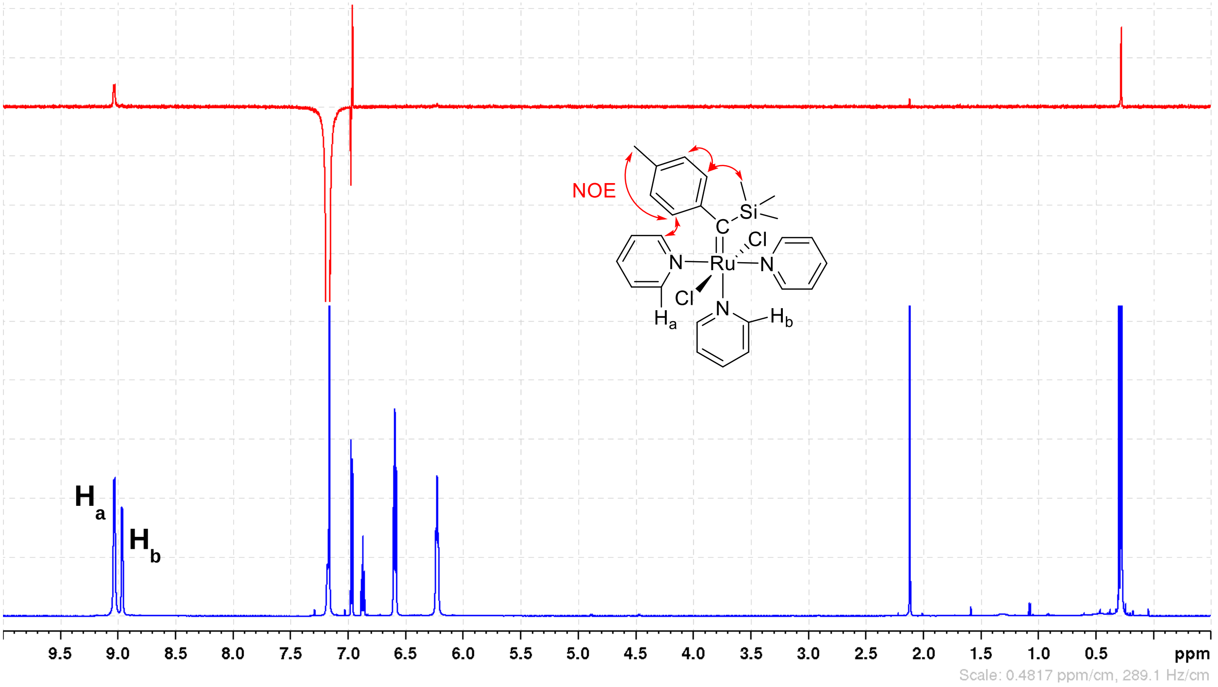

NOE spectrum of complex **39** in C_6D_6 at 600 MHz at 298 K, irradiating *ortho*-H atom of the *p*-tolyl group.

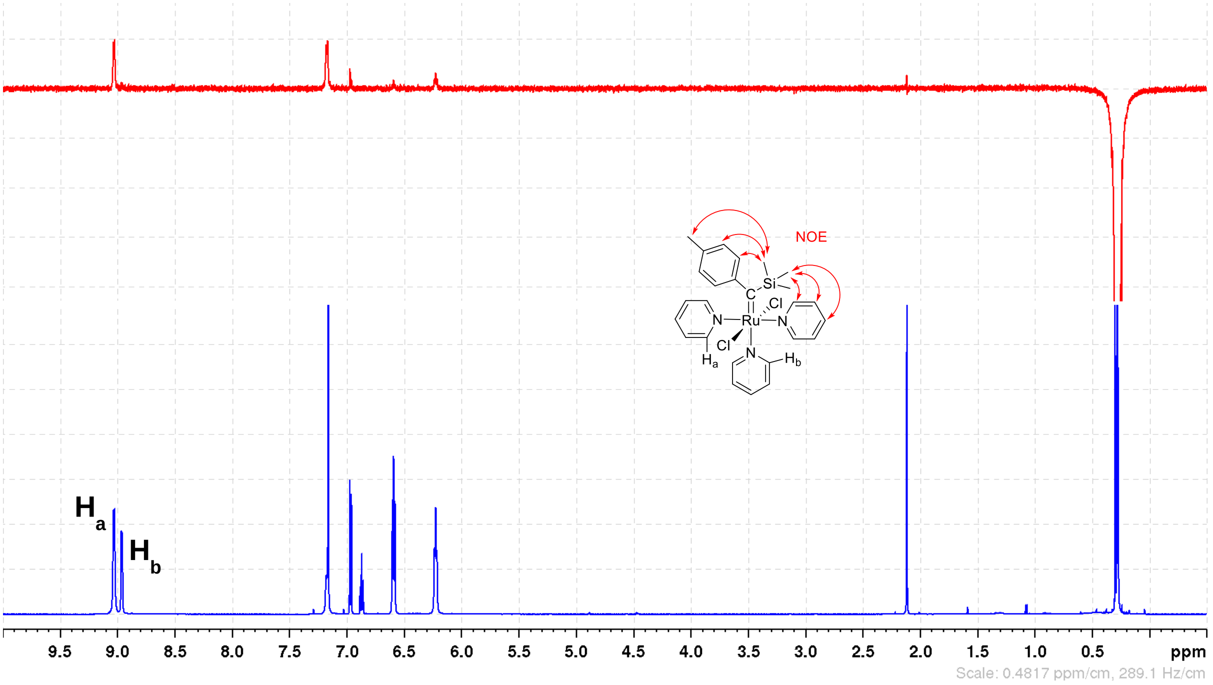

NOE spectrum of *trans*-Ru[=C(*p*-tolyl)TMS]$Cl_2(py)_3$ (**39**) in C_6D_6 at 600 MHz at 298 K, irradiating the TMS protons.

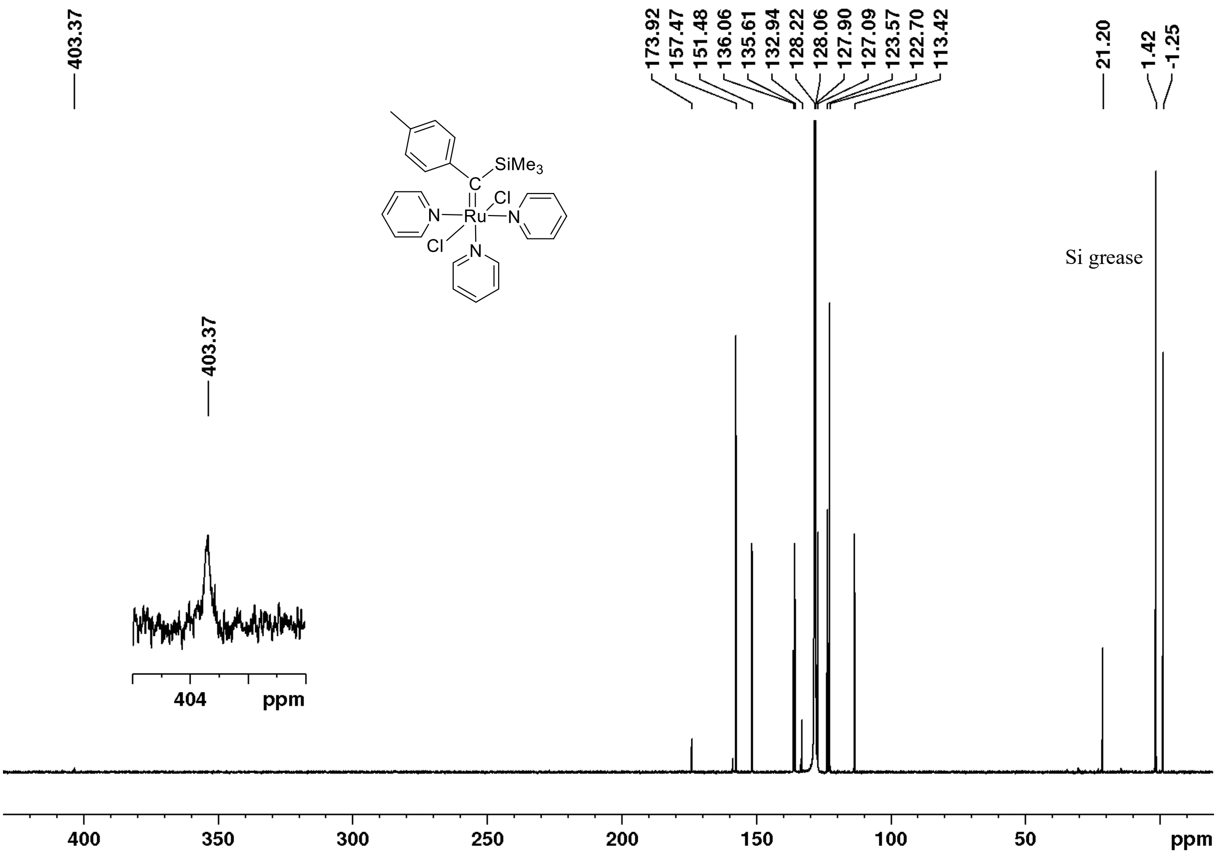

$^{13}C\{^1H\}$ NMR spectrum of complex **39** in C_6D_6 at 151 MHz at 298 K.

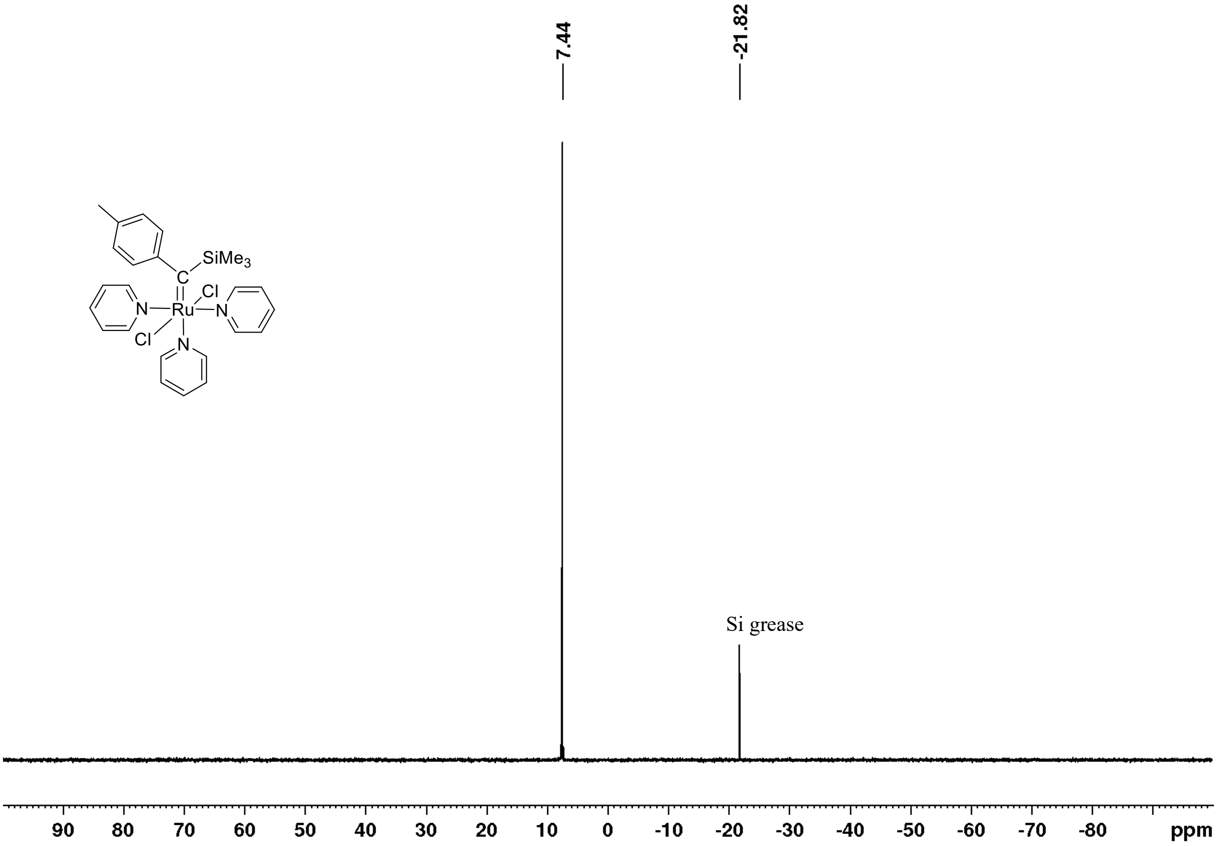

$^{29}Si\{^1H\}$ NMR spectrum of complex **39** in C_6D_6 at 119 MHz at 298 K

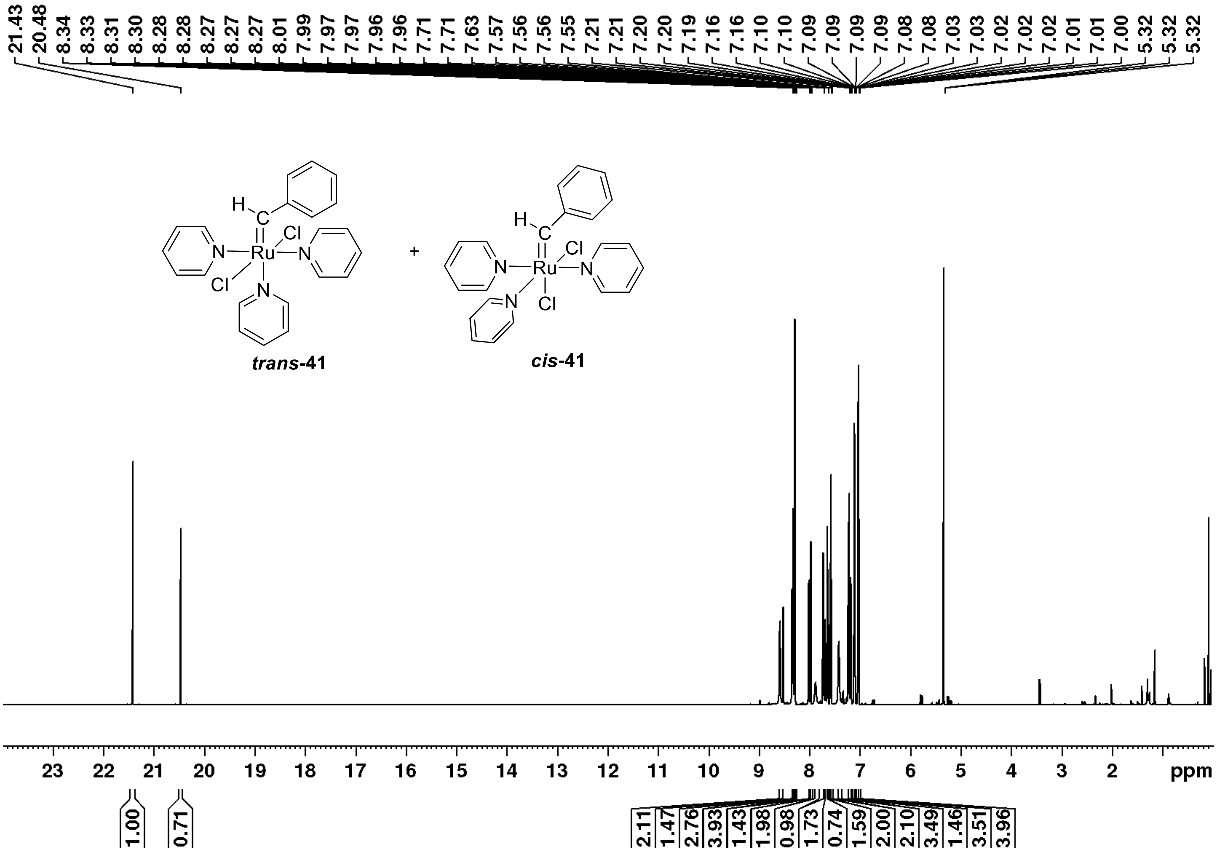

^{1}H NMR spectrum of complex **41** in CD_2Cl_2 at 600 MHz at 273 K

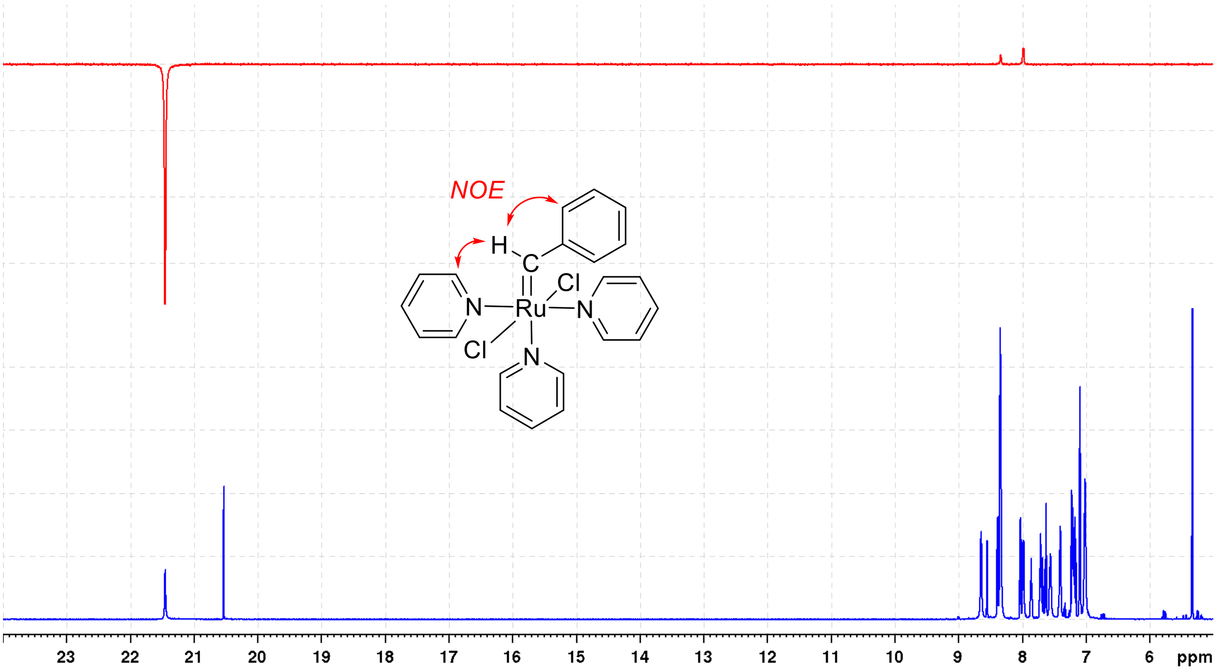

NOE spectrum of complex **41** in CD_2Cl_2 at 600 MHz at 298K, irradiating the carbene α-H atom of the *trans*-isomer

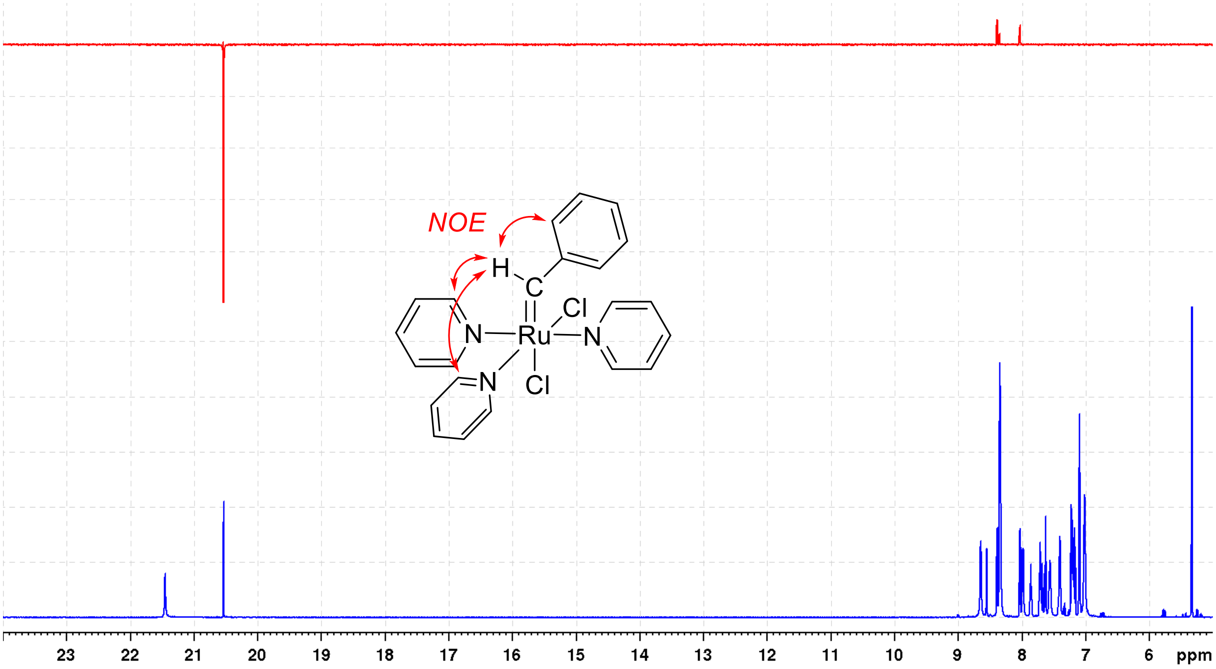

NOE spectrum of complex **41** in CD_2Cl_2 at 600 MHz at 298K, irradiating the carbene α-H atom of the *cis*-isomer

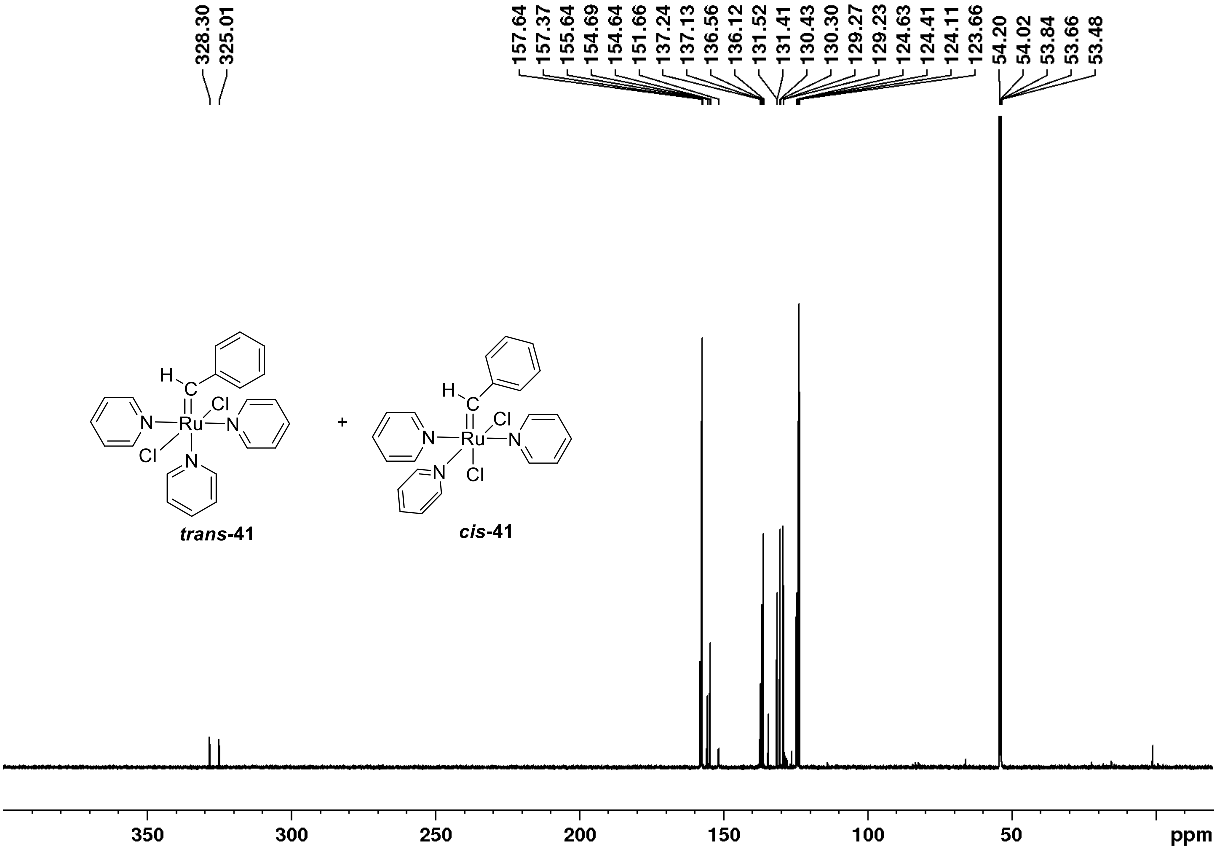

$^{13}C\{^1H\}$ NMR spectrum of complex **41** in CD_2Cl_2 at 151 MHz at 273 K

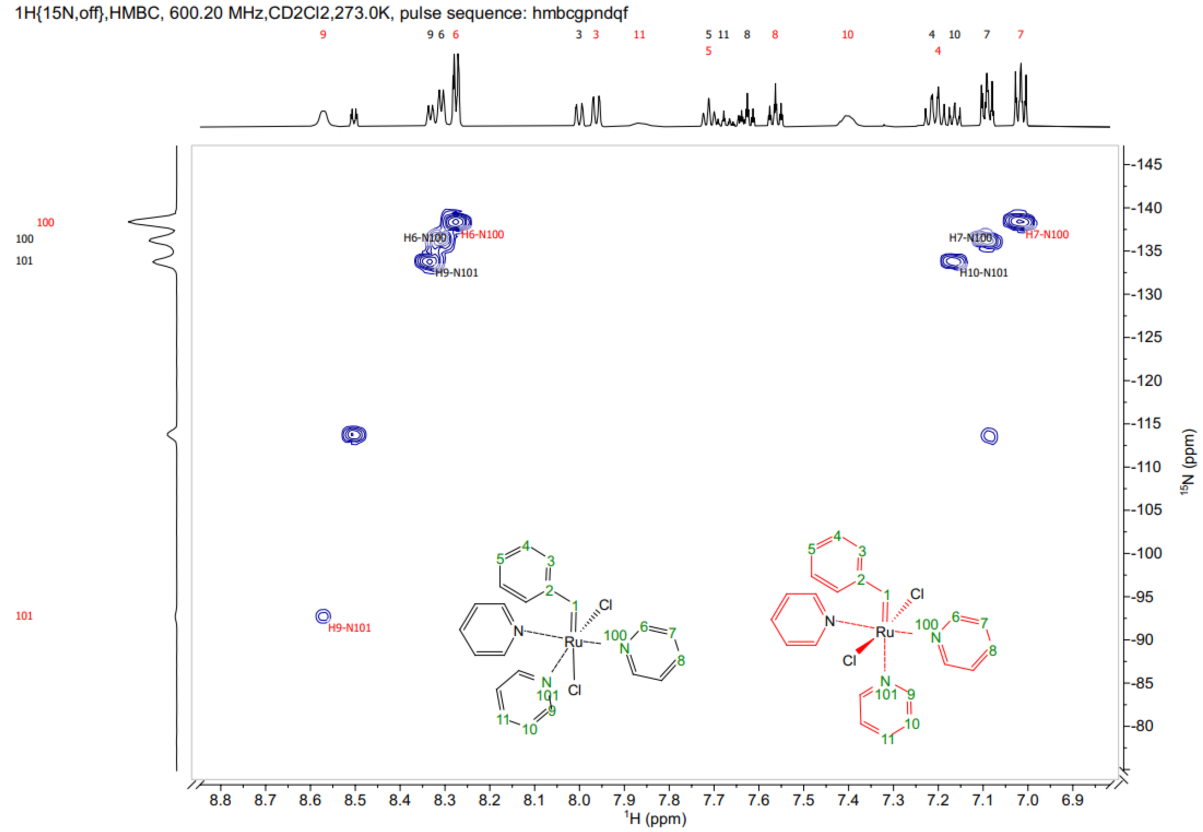

^{1}H-^{13}C HMBC spectrum of complex **41** in CD_2Cl_2

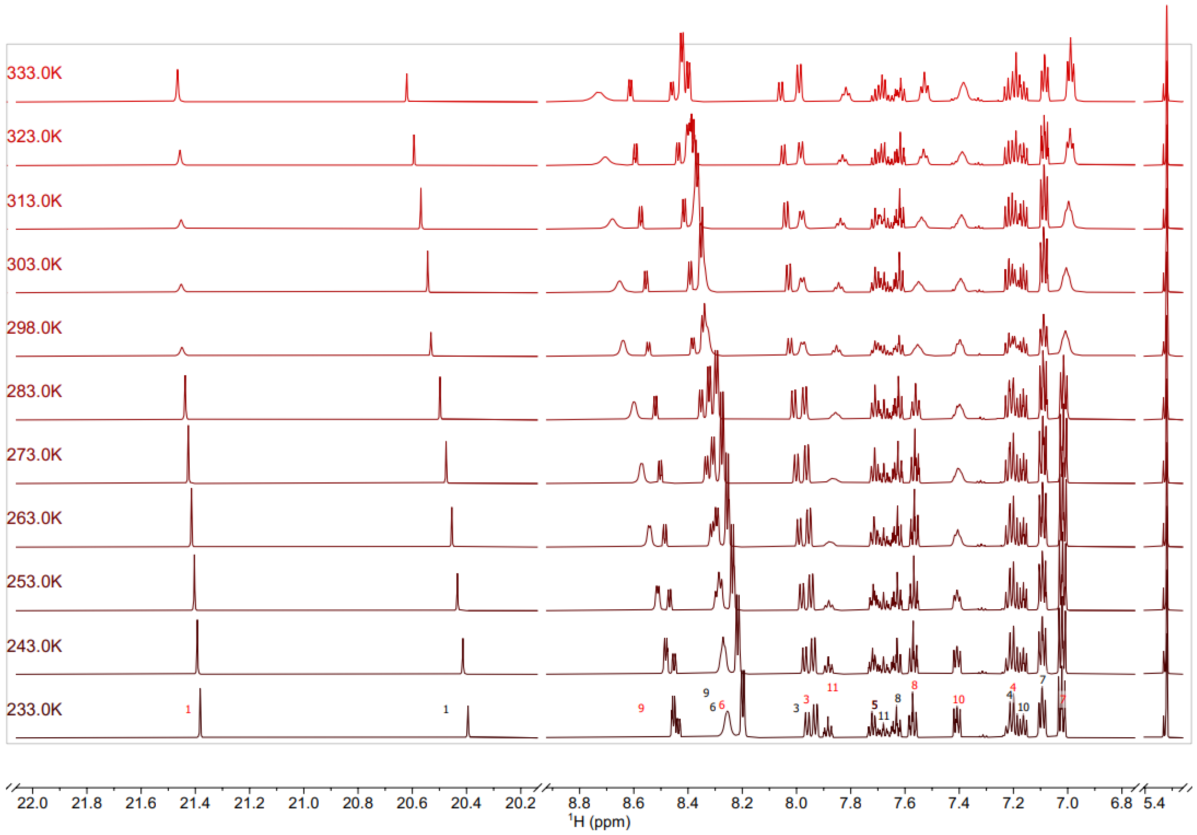

Variable temperature ^{1}H NMR spectra of the mixture of complexes *trans*-**41** (red numbers) and *cis*-**41** (black numbers) in CD_2Cl_2 at 600 MHz

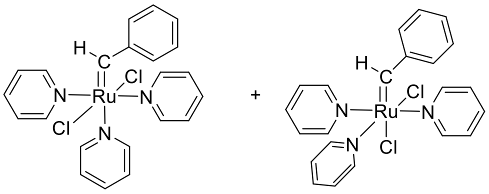

***trans*-41** + ***cis*-41**

Note: At room temperature, the signals corresponding to the carbene α-H-atom (H1), and the *trans*-pyridine protons (H9, H10 and H11) of complex *trans*-**41** are broadened, indicating a rapid pyridine exchange process at this temperature. At lower temperatures, these signals sharpen significantly, whereas resonances at other positions in the complexes (e.g. H6) become broadened, likely due to restricted rotation.

In contrast, the signals of *cis*-**41** remain sharp over the entire tested temperature range.

References

(1) A. M. Borys, *Organometallics* **2023,** *42*, 182-196.

(2) G. R. Fulmer, A. J. M. Miller, N. H. Sherden, H. E. Gottlieb, A. Nudelman, B. M. Stoltz, J. E. Bercaw, K. I. Goldberg, *Organometallics* **2010,** *29*, 2176-2179.

(3) A. J. Arduengo, R. Krafczyk, R. Schmutzler, H. A. Craig, J. R. Goerlich, W. J. Marshall, M. Unverzagt, *Tetrahedron* **1999**, *55*, 14523–14534.

(4) J. O. Krause, O. Nuyken, K. Wurst, M. R. Buchmeiser, *Chem. Eur. J.* **2004**, *10*, 777–784.

(5) R. Zhang, T. Yu, G. Dong, *Science* **2023**, *382*, 951–957.

(6) A. Michrowska, R. Bujok, S. Harutyunyan, V. Sashuk, G. Dolgonos, K. Grela*, J. Am. Chem. Soc.* **2004**, *126*, 9318–9325.

(7) V. Fasano, L. D. Curless, J. E. Radcliffe, M. J. Ingleson, *Angew. Chem., Int. Ed.* **2017**, *56*, 9202–9206.

(8) K. L. Tan, A. Vasudevan, R. G. Bergman, J. A. Ellman, A. J. Souers, *Org. Lett.* **2003**, *5*, 2131–2134.

(9) a) K. Yamamoto, S. Suzuki, J. Tsuji, *Tetrahedron Lett.* **1980,** *21*, 1653-1656. b) K. Yamamoto, A. Hayashi, S. Suzuki, J. Tsuji, *Organometallics* **1987,** *6*, 974-979.

(10) A. G. Brook, P. F. Jones, *Can. J. Chem.* **1969,** *47*, 4353-4358.

(11) S. R. Han, S. Kass, *J. Chem. Soc., Perkin Trans. 1* **1999**, 1553-1558.

(12) M. Cui, M. Leutzsch, A. A. Auer, A. Fürstner, *Angew. Chem. Int. Ed.* **2025**, *64*, e202519905

(13) S. R. Caskey, M. H. Stewart, Y. J. Ahn, M. J. A. Johnson, J. W. Kampf, *Organometallics* **2005,** *24*, 6074-6076.

(14) J. S. Kingsbury, J. P. A. Harrity, P. J. Bonitatebus Jr., A. H. Hoveyda, *J. Am. Chem. Soc.* **1999**, *121*, 791–799.

(15) R. J. Zachmann, A. Fürstner, *Chem. Eur. J.* **2021**, *27*, 7663–7666.

(16) W. A. Nugent, J. Feldman, J. C. Calabrese, *J. Am. Chem. Soc.* **1995**, *117*, 8992-8998.

(17) A. Fürstner, L. Ackermann, *Chem. Commun.* **1999**, 95–96.

(18) S.-H. Kim, W. J. Zuercher, N. B. Bowden, R. H. Grubbs, *J. Org. Chem.* **1996**, *61*, 1073-1081.

(19) A. Fürstner, K. Langemann, *J. Org. Chem.* **1996**, *61*, 3942-3943.

(20) W. H. Henderson, C. T. Check, N. Proust, J. P. Stambuli, *Org. Lett*. **2010**, 12, 824–827.

(21) J. A. Marshall, M. A. Wolf, E. M. Wallace, *J. Org. Chem.* **1997,** *62* (2), 367-371.

(22) A. Fürstner, O. R. Thiel, L. Ackermann, H.-J. Schanz, S. P. Nolan, *J. Org. Chem.* **2000**, *65*, 2204–2207.

(23) a) F. Neese, *WIREs Comput. Mol. Sci.* **2012**, *2*, 73–78; b) F. Neese, F. Wennmohs, U. Becker, C. Riplinger, *J. Chem. Phys.* **2020**, *152*, 224108; c) F. Neese, *WIREs Comput. Mol. Sci.* **2025**, *15*, e70019.

(24) a) A. D. Becke, *J. Chem. Phys.* **1993**, *98*, 1372–1377; b) F. Weigend, *Phys. Chem. Chem. Phys.* **2006**, *8*, 1057–1065; c) R. Izsák, F. Neese, *J. Chem. Phys.* **2011**, *135*, 144105. d) F. Weigend, R. Ahlrichs, *Phys. Chem. Chem. Phys.* **2005**, *7*, 3297–3305; e) C. Lee, W. Yang, R. G. Parr, *Phys. Rev. B* **1988**, *37*, 785–789; f) M. Garcia-Ratés, F. Neese, *J. Comput. Chem.* **2019**, *40*, 1816–1828; g) M. Garcia-Ratés, F. Neese, *J. Comput. Chem.* **2020**, *41*, 922–939; h) D. Andrae, U. Häußermann, M. Dolg, H. Stoll, H. Preuß, *Theor. Chim. Acta* **1990**, *77*, 123–141; i) E. Caldeweyher, C. Bannwarth, S. Grimme, *J. Chem. Phys.* **2017**, *147*, 034112.

(25) S. Grimme, *Chem. Eur. J.* **2012**, *18*, 9955–9964.
